# One-Pot Biocatalytic Synthesis of Primary, Secondary, and Tertiary Amines with Two Stereocenters from α,β-Unsaturated Ketones Using Alkyl-Ammonium Formate

**DOI:** 10.1021/acscatal.2c03052

**Published:** 2022-11-10

**Authors:** Tanja Knaus, Maria L. Corrado, Francesco G. Mutti

**Affiliations:** Van’t Hoff Institute for Molecular Sciences, HIMS-Biocat, University of Amsterdam, Science Park 904, 1098 XH Amsterdam, The Netherlands

**Keywords:** biocatalysis, reductive amination, α-chiral amines, biocatalytic cascades, imine reductases, reductive aminases, ene-reductases

## Abstract

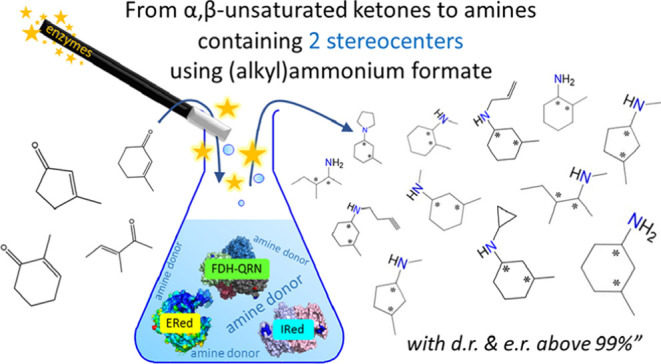

The efficient asymmetric catalytic synthesis of amines containing more than one stereogenic center is a current challenge. Here, we present a biocatalytic cascade that combines ene-reductases (EReds) with imine reductases/reductive aminases (IReds/RedAms) to enable the conversion of α,β-unsaturated ketones into primary, secondary, and tertiary amines containing two stereogenic centers in very high chemical purity (up to >99%), a diastereomeric ratio, and an enantiomeric ratio (up to >99.8:<0.2). Compared with previously reported strategies, our strategy could synthesize two, three, or even all four of the possible stereoisomers of the amine products while precluding the formation of side-products. Furthermore, ammonium or alkylammonium formate buffer could be used as the only additional reagent since it acted both as an amine donor and as a source of reducing equivalents. This was achieved through the implementation of an NADP-dependent formate dehydrogenase (FDH) for the in situ recycling of the NADPH coenzyme, thus leading to increased atom economy for this biocatalytic transformation. Finally, this dual-enzyme ERed/IRed cascade also exhibits a complementarity with the recently reported EneIRED enzymes for the synthesis of cyclic six-membered ring amines. The ERed/IRed method yielded trans-1,2 and cis-1,3 substituted cyclohexylamines in high optical purities, whereas the EneIRED method was reported to yield one cis-1,2 and one trans-1,3 enantiomer. As a proof of concept, when 3-methylcyclohex-2-en-1-one was converted into secondary and tertiary chiral amines with different amine donors, we could obtain all the four possible stereoisomer products. This result exemplifies the versatility of this method and its potential for future wider utilization in asymmetric synthesis by expanding the toolbox of currently available dehydrogenases via enzyme engineering and discovery.

## Introduction

The development of efficient and highly selective catalytic methods for the synthesis of α-chiral amines in optically pure form is of great importance for the pharmaceutical industry.^1,2^ Therefore, several organometallic-, organo-, and photoredox-catalysts possessing improved activity and selectivity have been developed during the past 2 decades for the synthesis of α-chiral amines starting from prochiral substrates.^3−16^ In this context, biocatalysis has made a tremendous contribution since an arsenal of enzymes from different families is now available for the synthesis of α-chiral amines in high optical purity;^17−21^ these enzyme families comprise hydrolases,^22−24^ ω-transaminases,^25−29^ ammonia lyases,^30,31^ amine oxidases,^19,32−35^ imine reductases/reductive aminases,^36−38^ amine dehydrogenases,^36,39^ engineered cytochromes P450,^40−42^ and Pictet-Spenglerases.^43−45^

Among these methods, enzymatic reductive amination is of great interest since it permits synthesizing enantiomerically pure primary, secondary, and tertiary amines through the reductive coupling of a prochiral ketone acceptor with an amine donor. This feature enables access to a wide structural diversity.^36−39,46−72^ As an alternative, the reductive amination of a non-prochiral substrate, such as an aldehyde, has been combined with the kinetic resolution of a racemic amine donor.^73^

However, many valuable biologically active compounds possess multiple stereocenters and, therefore, are more challenging to synthesize.^2,74,75^ In principle, these complex syntheses can be accomplished by developing enzymatic cascades.^76−81^ The few available methods deal with the synthesis of enantiomerically pure or enantioenriched 1,2-amino alcohols through one-pot cascade reactions combining a carboligase with a transaminase,^82^ an alcohol dehydrogenase with a transaminase,^83,84^ or an alcohol dehydrogenase with an amine dehydrogenase.^85^ An alternative strategy is the transamination of α-hydroxy ketones.^86^

The enzymatic stereoselective synthesis of β-alkyl/aryl- or γ-alkyl/aryl-substituted α-chiral amines containing at least two stereogenic centers has scarcely been investigated due to difficulties in finding compatible biocatalysts and the previous unavailability of reductive aminases and amine dehydrogenases. These structural motives, found already in some commercial pharmaceuticals (see Supporting Information, Figure S2), can be accessed through the asymmetric reduction of the alkene moiety of an α,β-unsaturated ketone using an ene-reductase, followed by the reductive amination of the carbonyl moiety. Ene-reductases (EReds) are flavin-dependent enzymes that catalyze the asymmetric reduction of activated alkenes, such as α,β-unsaturated aldehydes, ketones, and esters (also acids in particular cases), as well as cyano- and nitro-functionalized alkenes.^87−93^

Riva’s group and Bornscheuer’s group independently reported the combination of EReds with ω-transaminases (ωTAs) for the synthesis of diastereomerically enriched amines bearing two stereogenic centers.^94,95^ The former group performed the synthesis of two of the four possible stereoisomers of 3-methyl-4-phenylbutan-2-amine, 1-(chroman-3-yl)ethan-1-amine, and 3-methylcyclohexan-1-amine (**1c**) in excellent diastereomeric ratios (d.r. 94:6 to >99:<1).^94^ The latter group focused specifically on the synthesis of **1c** and obtained three of the four stereoisomers with moderate or high d.r. (86:14 to 98:2) by using two stereocomplementary EReds with one ωTA.^95,96^ In a recent publication, Paul’s group and Vergne-Vaxelaire’s group jointly reported the synthesis of α-chiral amines by combining EReds with native amine dehydrogenases (AmDHs). They obtained four structures of amine products containing two stereogenic centers, but only one (or two in one case) of the four possible stereoisomers was obtained with variable values of d.r. (65:35 to 99:1) and high e.r. (99:1).^97^

**Scheme 1:**
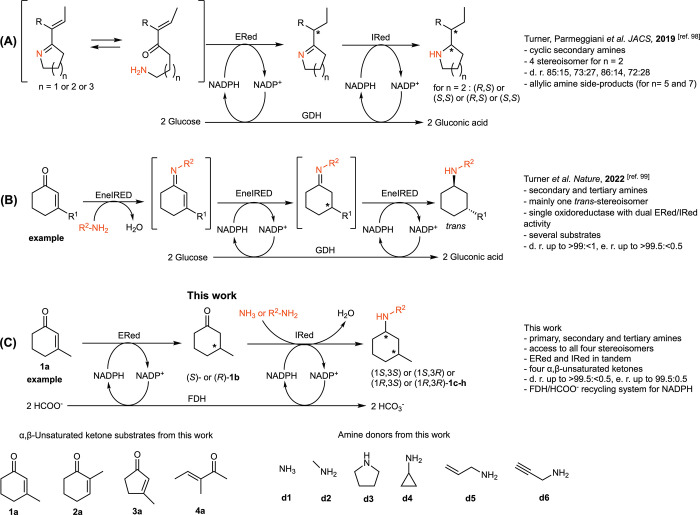
Highlights of Biocatalytic Strategies for the Asymmetric Synthesis of Primary, Secondary, and Tertiary Chiral Amines Bearing Two Stereogenic Centers in α,β or α,γ Positions

Notably, due to the inherent catalytic activity of ωTAs or AmDHs, all the studies mentioned above could give access only to primary α-chiral amines; furthermore, the possibility of synthesizing all the four possible stereoisomers remained elusive. Turner, Parmeggiani, and co-workers reported an important extension of these synthetic strategies by accomplishing the stereoselective synthesis of five-, six-, and seven-membered ring N-heterocycles containing two stereocenters (Scheme 1A).^98^ This was the first example of the synthesis of cyclic tertiary chiral amines by combining an ERed with an imine reductase (IRed). All four stereoisomers of one of the target amine products, namely 2-(*sec*-butyl)piperidine, were obtained in a diastereomerically enriched form (72:28 to 86:14). In another outstanding and recently published work, Turner’s group reported the synthesis of secondary and tertiary β-alkyl- and γ-alkyl-substituted α-chiral amines (Scheme 1B). Most notably, the reaction was catalyzed by a single oxidoreductase exhibiting both ERed and IRed activities. This family of enzymes, named EneIREDs, appears to give access to one of the four possible stereoisomers starting from a given ketone substrate and an amine donor.^99^ Although the synthesis with a single oxidoreductase possessing dual activity is of practical convenience, the use of two separated ERed and IRed still has the advantage of enabling the creation of modular cascades in which all the stereoisomers of an amine product are, in principle, accessible by combining enzymes with different stereoselectivities.

In this work, we developed a biocatalytic cascade for the asymmetric synthesis of primary, secondary, and tertiary amines bearing two stereocenters by studying the combination of EReds with IReds/RedAms with a selected panel of α,β-unsaturated ketones and amine donors (Scheme 1C). In the case of the reductive amination of 3-methylcyclohex-2-en-1-one (**1a**, Scheme 1C) with different amine donors for the synthesis of secondary and tertiary amine products, we obtained all the four possible stereoisomers with an excellent diastereomeric and enantiomeric ratio. Furthermore, we introduced the use of an NADP-dependent formate dehydrogenase for the in situ recycling of the reduced form of the nicotinamide adenine dinucleotide phosphate co-factor (NADPH). This enabled avoiding the addition of glucose as a sacrificial co-substrate because the ammonium or alkylammonium formate buffer was both the source of reducing equivalents and the amine donor.

## Results and Discussion

### Asymmetric Alkene Moiety Reduction of α,β-Unsaturated Ketones (**1–4a**) Using a Panel of Ene-Reductases (EReds)

We started our investigation by testing the asymmetric alkene moiety reduction of selected α,β-unsaturated ketones using a panel of ene-reductases (EReds)^100^ combined with a variant of the formate dehydrogenase from *Candida boidinii* (Cb-FDH-QRN),^101^ which is thus the so-called “couple-enzyme approach”. This initial phase of the work was important because the biocatalytic alkene reduction using an NADP-dependent FDH for the in situ recycling of NADPH has not been previously reported. In fact, the widely applied strategy for NADPH recycling in this biocatalytic reaction has always been the use of a glucose dehydrogenase (GDH), which requires the consumption of one equivalent of glucose as the sacrificial co-substrate.^87−91,93^ In contrast, the use of FDH/formate salt would result in a higher atom economy (see Supporting Information, Section 3). This is even more advantageous in the designed multi-enzyme cascade in which the asymmetric reduction of the alkene moiety is followed by asymmetric reductive amination, which ultimately requires ammonium or alkylammonium formate as the only additional reagent.

We first performed the alkene moiety reduction of 3-methylcyclohex-2-en-1-one (**1a**) by testing the whole panel of NADP-dependent EReds: PETNR,^102^ TOYE,^103^ OYE2,^104^ OYE3,^104^ XenA,^105,106^ XenB,^105,106^ LeOPR1,^107^ NerA,^108^ GluOx,^109^ YqjM,^110^ MR,^111^ and YqjM-v1^112^ in KPi buffer. The two NAD-dependent EReds (NerA and MR) were also tested for the same reaction in which NAD^+^ was recycled using the wild-type Cb-FDH.^113^ OYE2 and YqjM-v1 were the best-performing EReds in terms of conversion and stereoselectivity for the formation of (*S*) and (*R*)-**1b**, respectively (Table 1, entries 1 and 4; Supporting Information, Table S1). The obtained e.r. values agreed with the previously reported data using GDH/glucose as a recycling system.^112,114^ In a few cases, the formation of the phenol by-product **1b′** (Supporting Information, Section 6.1) was observed, and this is due to the promiscuous disproportionation activity of some EReds on **1a**, as described in the literature.^115^ Because the overall cascade reactions, comprising alkene reduction and carbonyl reductive amination, must run in a formate buffer of the amine donor, we tested the alkene reduction using OYE2 and YqjM-v1 in ammonium formate (HCOONH_4_) or methylammonium formate buffer (HCOONH_3_CH_3_) at a concentration of 1 M and a pH of 8.8. The change in the reaction buffer slightly affected the conversions, which decreased in the case of OYE2 and increased in the case of YqjM-v1. In contrast, variation in the enantiomeric ratios of (*S*) and (*R*)-**1b** was negligible (Table 1, entries 2,3 and 5–7). Significant phenol **1b′** formation was observed only for OYE2. In summary, these results demonstrated that both EReds and Cb-FDH-QRN are compatible with a formate salt buffer of the amine donor that is required for the subsequent reductive amination of the carbonyl moiety.

**Table 1:** Study on the Asymmetric Biocatalytic Reduction of the Alkene Moiety of α,β-Unsaturated Ketones (**1–4a**, 10 mM) Using a Broad Panel of Biochemically Characterized Ene-Reductases (EReds, 5–20 μM) and an NADP-Dependent FDH Variant (Cb-FDH-QRN, 5 μM) for the Recycling of the Catalytic NADPH Co-enzyme (0.25 mM Added as NADP^+^) in the Presence of HCOONa (30 mM)^a^

entry	substrate	ERed type	ERed conc. [μM]	*T* [°C]	buffer type	pH	conversion^b^**1–4b** [%]	side-product **1–2b′** [%]	e.r.^c^
1	**1a**	OYE2	5	30	KPi	8	97	n.d.	>99.8:<0.2 (*S*)^d^
2	**1a**	OYE2	5	30	HCOONH_4_	9	85	4	>99.8:<0.2 (*S*)^d^
3	**1a**	OYE2	5	30	HCOONH_3_CH_3_	9	84	4	>99.8:<0.2 (*S*)^d^
4	**1a**	YqjM-v1	5	30	KPi	8	89	<1	97:3 (*R*)
5	**1a**	YqjM-v1	5	30	HCOONH_4_	8.8	>99	<1	98:2 (*R*)
6	**1a**	YqjM-v1	5	30	HCOONH_3_CH_3_	8.8	>99	<1	98:2 (*R*)
7	**1a**	YqjM-v1	5	10	HCOONH_3_CH_3_	8.8	>99	n.d.	99:1 (*R*)
8	**2a**	TOYE	20	10	KPi	8	88	4	99:1 (*R*)
9	**2a**	TOYE	20	10	HCOONH_4_	8	98	2	94:6 (*R*)
10	**2a**	TOYE	20	10	HCOONH_3_CH_3_	8	>99	<1	97:3 (*R*)
11	**2a**	XenB	20	10	KPi	8	99	n.d.	97:3 (*R*)
12	**2a**	XenB	20	10	HCOONH_4_	8	>99	n.d.	93:7 (*R*)
13	**2a**	XenB	20	10	HCOONH_3_CH_3_	8	>99	n.d.	96:4 (*R*)
14	**3a**	PETNR	5	30	KPi	8	21	n.a.	99.7:0.3 (*S*)
15	**3a**	PETNR	20	30	HCOONH_4_	8	67	n.a.	>99.8:<0.2 (*S*)^d^
16	**3a**	PETNR	20	30	HCOONH_3_CH_3_	9	61	n.a.	99.8:0.2 (*S*)
17	**3a**	OYE2	5	30	KPi	8	40	n.a.	99.6:0.4 (*S*)
18	**3a**	OYE2	20	20	HCOONH_4_	8.8	78	n.a.	>99.8:<0.2 (*S*)^d^
19	**3a**	OYE2	20	20	HCOONH_3_CH_3_	8	68	n.a.	99.8:0.2 (*S*)
20	**3a**	YqjM-v1	10	30	HCOONH_4_	8.8	10	n.a.	77:23 (*R*)
21	**3a**	YqjM-v1	10	30	HCOONH_3_CH_3_	8.8	10	n.a.	76:24 (*R*)
22	**4a**	XenA	5	30	KPi	8	97	n.a.	99:1 (*S*)
23	**4a**	XenA	10	10	HCOONH_4_	8	99	n.a.	99:1 (S)
24	**4a**	XenA	10	10	HCOONH_3_CH_3_	8	>99	n.a.	99:1 (*S*)
25	**4a**	YqjM	5	30	KPi	8	97	n.a.	97:3 (*S*)
26	**4a**	YqjM-v1	5	30	KPi	8	99	n.a.	73:27 (*R*)
27	**4a**	YqjM-v1	10	30	HCOONH_4_	8.8	>99	n.a.	76:24 (*R*)
28	**4a**	YqjM-v1	10	30	HCOONH_3_CH_3_	8.8	>99	n.a.	76:24 (*R*)

a Two NAD-dependent EReds were also tested using NAD^+^ (0.25 mM) and WT Cb-FDH (5 μM). The reactions were conducted in HCOONH_4_ and in HCOONH_3_CH_3_ buffers (1 M, at various pH and *T* values) as these are the required reaction environments for the final intended cascade. Reactions in KPi buffer (50 mM, pH 8, 1 mL) were also performed to enable comparison and data analysis as this is the most applied buffer with EReds in the literature.

b Measured by GC–FID using an achiral column (DB-1701, 30 m, Agilent).

c Measured by GC–FID using a chiral column (Rt-bDEXsm, Restek for **1b**, **3b**, and **4b** and Rt-bDEXsa, Restek for **2b**). The enantiomeric ratio values were reported with one significant decimal digit if the value was 99.5:0.5 or higher; in the other cases, the value was rounded to the nearest integer number.

d e.r. reported as >99.8:<0.2 (equal to an e.e. of >99.6%) because the (*R*)-configured enantiomer was not observed (below the detection limit); therefore, the enantiomeric ratio was calculated and reported based on the detection limit of the GC (2 area units).

We conducted the same investigation on the next model substrate, namely 2-methylcyclohex-2-en-1-one (**2a**) in KPi buffer. This substrate is more challenging because the related saturated product **2b** is an α-substituted ketone, which is therefore prone to racemization due to keto–enol tautomerism.^100^ TOYE and XenB turned out to be the best enzymes in terms of the conversion and enantiomeric ratios at 10 °C (Table 1, entries 8 and 11; Supporting Information, Table S10). Notably, the reported values represent an improvement compared with published data, in which the highest reported e.r. was 93:7 with this set of EReds.^114,116^ Therefore, the biocatalytic reactions with TOYE and XenB were further investigated in HCOONH_4_ and in HCOONH_3_CH_3_ buffers at the same temperatures (10, 20, and 30 °C) and different pH values (8 and 8.8; see Supporting Information, Table S11). The change in the buffer composition resulted in an increased conversion into the product (*R*)-**2b** but a decrease in the e.r. (see Table 1, entries 9–10, 12–13). In general, (*R*)-**2b** was obtained in higher e.r. when the reaction was conducted at pH 8 rather than pH 8.8 (see Supporting Information, Table S11). In this work, we also proved that the variation in the ee values must be (at least mainly) attributed not to an inherent change in the stereoselectivity of the EReds under different reaction conditions but to the different rate of the spontaneous chemical racemization of **2b** (for details, see Supporting Information, Section 7.1 and Table S12). The racemization rate of **2b** depended partly on the temperature and pH, and it was lower at a lower temperature and neutral pH. However, we noticed that the type of buffer contributed more than the pH and temperature to the depletion of the e.e. The depletion of the enantiomeric excess was also greater in HCOONH_4_ than in HCOONH_3_CH_3_.

In a similar way, we investigated the reduction of 3-methylcyclopent-2-en-1-one (**3a**) in KPi buffer. In this case, PETNR and OYE2 were the best-performing EReds (Table 1, entries 14 and 17; see Supporting Information, Table S25, for the full dataset). Switching the buffer to HCOONH_4_ or HCOONH_3_CH_3_ did not affect the e.r., while the conversion was increased by increasing the ERed concentrations (Table 1, entries 15, 16, 18, 19; Supporting Information, Table S25). Finally, YqjM-v1 produced the opposite enantiomer in all the tested buffers with low conversion and moderate e.r. (see Table 1, entries 20 and 21).

(*E*)-3-methylpent-3-en-2-one (**4a**) was the last tested model of an α,β-unsaturated ketone substrate (see Supporting Information, Table S31). XenA and YqjM-v1 were the best-performing EReds for the synthesis of (*S*)-**4b** and (*R*)-**4b** in KPi buffer, respectively (Table 1, entries 22 and 25; Supporting Information, Table S31). The absolute configuration of (*S*)-**4b** was assigned by measuring the optical rotation of a sample obtained from a 61 mg–scale reaction catalyzed by wild-type YqjM (e.r. >97:<3 (*S*), 78% isolated yield due to the volatility of **4b**) and by comparing with literature data (see Supporting Information, Section 12.4.1).^117^ As in the case of **2b**, the obtained (*S*)-**4b** is an α-substituted saturated ketone and, therefore, prone to racemization in solution. In fact, the same effect observed for the racemization of (*R*)-**2b** was observed for the racemization of (*S*)-**4b**. Therefore, the optimized reaction conditions in HCOONH_4_ and HCOONH_3_CH_3_ buffers were found again at pH 8 and 10 °C (Table 1, entries 23 and 24; Supporting Information, Table S31). The saturated ketone **4b** was also obtained in quantitative conversions and in an (*R*)-configured enantioenriched form by using YqjM-v1 (see Table 1, entries 26–28).

### Asymmetric Reductive Amination of Saturated Ketones (**1–4b**) Using a Panel of Imine Reductases (IReds) and Amine Dehydrogenases (AmDHs) to Give Primary Amines (**1–4c**)

**Figure 1:**
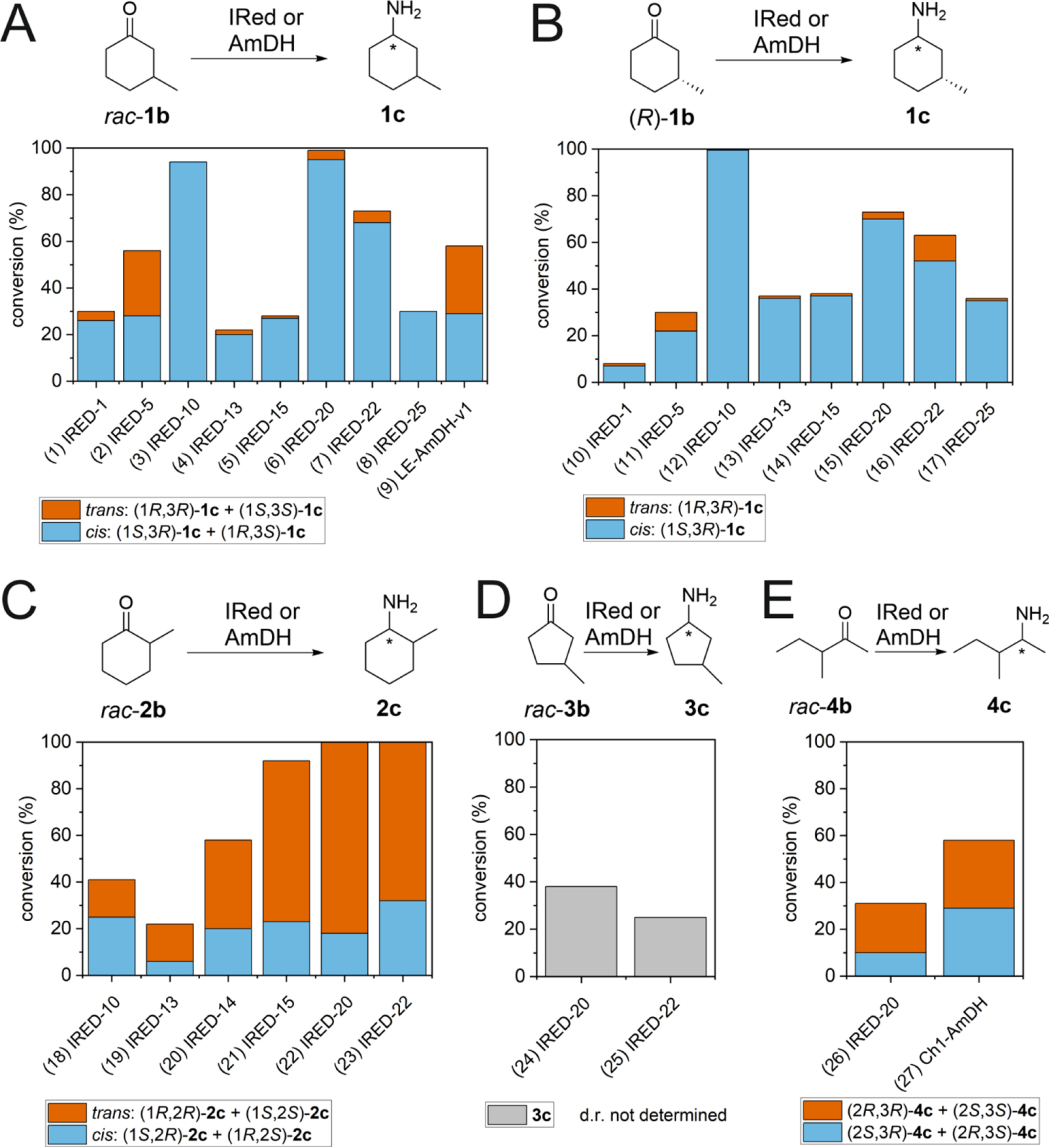
Study on the biocatalytic reductive amination of the saturated ketones (**1–4b**, 10 mM) using a broad panel of imine reductases (IReds, 20 μM) and selected amine dehydrogenases (AmDHs, 20 μM) with ammonia as an amine donor. The reactions were conducted in HCOONH_4_ buffer (1 M, pH 8.8) at a *T* of 30 °C for 24 h with a catalytic amount of either NADP^+^ or NAD^+^ (0.25 mM) and a suitable formate dehydrogenase (FDH 5 μM) for co-factor recycling.

**Scheme 2:**
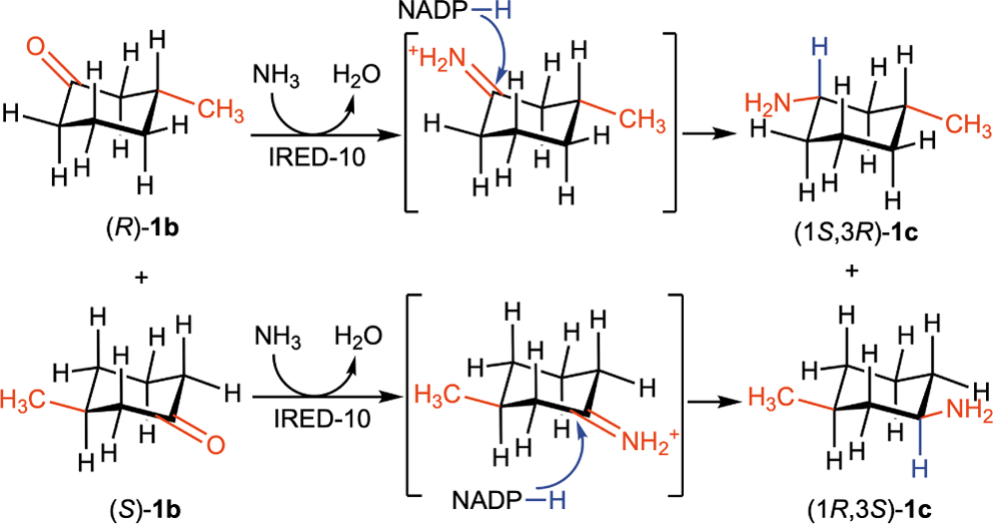
Stereochemical Behavior of IRED-10 in the Reduction of Racemic Ketone **1b**

A panel of 16 imine reductases (IReds)^46,48,118,119^ and three amine dehydrogenases (AmDHs)^59−62^ of known amino acid sequences was tested for the reductive amination of the saturated ketones **1–4b** (for a description of these enzymes, such as strains of origin, see Supporting Information, Section 4). Although most of these experiments were conducted using racemic ketones **1–4b**, we point out that reductive amination will eventually run on enantiomerically pure or enriched ketones **1–4b** in the final cascades. Therefore, it is more important that the IReds or AmDHs can install the additional stereogenic center by reducing the carbonyl moiety with high stereoselectivity than that they can distinguish the existing chirality of the substrate at the other carbon atom. We performed the biocatalytic reductive aminations of the ketone substrates in HCOONH_4_ buffer (1 M, pH 8.8) with an aminating enzyme at 30 °C for 24 h. As the 16 IReds were reported to be NADP dependent, NADP^+^ and Cb-FDH-QRN were added again for co-factor recycling. In the case of the AmDHs, namely Ch1-AmDH, Rh-PhAmDH, and LE-AmDH-v1, NAD^+^ and wild-type Cb-FDH were added because the three AmDHs prefer NADH as a co-factor. We initially hypothesized that the reductive amination of racemic 3-methylcyclohexan-1-one (*rac*-**1b**) would have led to a mixture of diastereomers of **1c** because the absolute configuration at the substrate’s β-carbon atom was supposed to not strongly influence the stereoselectivity of the hydride transfer from NADPH to the protonated imine intermediate in the active site of the enzymes. In contrast, the reaction with IRED-10, for example, yielded only a couple of cis enantiomers, namely (1*S*,3*R*)-**1c** and (1*R*,3*S*)-**1c**, with a total conversion of 94%, the remaining compound being unreacted *rac*-**1b** (see Figure 1A, entry 3, and Supporting Information, Tables S2 and S44, for the full dataset). This means that IRED-10 can efficiently aminate both enantiomers of **1b**, but the absolute configuration of the newly created stereogenic center (α-chiral carbon to the amine moiety) is controlled by the absolute configuration of the pre-existing stereogenic carbon atom of ketone substrate **1b**. Turner’s group has previously observed this type of “substrate-controlled” stereoselectivity in the reductive amination of 2,6-, 2,5-, and 2,4-disubstituted piperideine catalyzed by IReds.^120^ Also, in their case, the inherent selectivity of the IReds was overridden by the existing chirality at the methyl-substituted carbon atom. The authors postulated that the hydride attacks the cyclic imine anti-periplanar to the methyl substituent to avoid a steric clash. Accordingly, it appears in our study that substrate **1b** binds to the IRED-10’s active site in such a way that the hydride is also transferred from NADPH antiperiplanary to the methyl substituent (Scheme 2). Therefore, the amination of the enantiomer (*R*)-**1b** with IRED-10 proceeded toward the *Re*-face of the carbonyl moiety to give (1*S*,3*R*)-**1c**, while the amination of the (*S*)-**1b** proceeded toward the *Si*-face of the carbonyl moiety to give (1*R*,3*S*)-**1c**.

This finding was also supported by the reductive amination of enantiopure (*R*)-**1b** catalyzed by IRED-10, which afforded (1*S*,3*R*)-**1c** as the only stereoisomer in quantitative conversion (see Figure 1B, entry 12, and Supporting Information, Tables S3 and S44, for the full dataset). The other enzymes that performed well in terms of conversion for the amination of *rac*-**1b** were IRED-1, 5, 13, 15, 20, 22, and 25 (conversions 22–99%). In general, the formation of *cis*-configured isomers of **1c** was prevalent with most tested IReds, but varied amounts of trans-**1c** were also formed (Figure 1A, entries 1 and 3–8; see also Supporting Information, Table S2, for more data). In fact, only in three out of 19 cases was an equal ratio between the cis and trans diastereomers obtained, namely with IRED-5, LE-AmDH-v1, and IRED-30; the remaining compound was always unreacted *rac***-1b** (Figure 1A, entries 2 and 9, for the selected data; see also Supporting Information, Tables S2 and S44, for the full dataset). The amine dehydrogenase Ch1-AmDH was the only enzyme that yielded a higher concentration of the trans enantiomer couple (77:23 d.r.; see Supporting Information, Table S2, for the full dataset). When we performed the reductive amination starting from (*R*)-**1b**, the preferentially formed product with the active IReds was the expected (1*S*,3*R*)-**1c** (d.r. varying from 73:27 to >99.5:<0.5) (Figure 1B for the selected data; see Supporting Information, Table S3, for the full dataset). In conclusion, based on the results from Supporting Information Tables S2 and S3, IRED-1, 5, 10, 13, 15, 20, 22, and 25 were selected to test the overall cascade from **1a** to **1c** stereoisomers, which will be reported in the next section.

The reductive amination of racemic 2-methylcyclohexan-1-one (*rac*-**2b**) in HCOONH_4_ buffer was performed under the same reaction conditions as for the reductive amination of *rac*-**1b**. IRED-20 and IRED-22 led to quantitative conversion into **2c** with a diastereomeric ratio of 82:18 and 68:32, respectively (see Figure 1C, entries 22 and 23, and Supporting Information, Table S44). The other IReds that yielded a significant conversion (22–92%), namely IRED-10, 13, 14, and 15, also exhibited a non-equal diastereomeric ratio (see Supporting Information, Table S13, for the full dataset). Although the rationalization of the stereochemical outcome of the reaction for the synthesis of **2c** is more complicated than for the synthesis of **1c** due to the occurrence of keto–enol tautomerisms for **2b**, as previously discussed, most of the IReds produced mainly the trans-configured isomers [i.e., (1*S*,2*S*) and (1*R*,2*R*)-**2c**)], as depicted in Figure 1C (entries 19–23). This indicates that the hydride transfer from NADPH occurs prevalently syn-periplanar to the methyl substituent for 2-methyl-substituted cyclohexane. However, four IReds preferentially formed the cis-configured isomers of **2c**, of which IRED-10 yielded the highest conversion (Figure 1C, entry 18). Only Ch1-AmDH selectively yielded only *cis*-**2c** (see Supporting Information, Table S13, for the full dataset). The IReds reported in Figure 1C were selected to test the overall cascade from **2a** to **2c** stereoisomers.

Racemic 3-methylcyclopentan-1-one (*rac*-**3b**) turned out to be a challenging substrate for the reductive amination with ammonia as an amine donor since it was converted only by IRED-20 and 22 (Figure 1D, entries 24 and 25; see Supporting Information, Tables S26 and S44, for the full dataset). Therefore, these two IReds were selected to test the overall cascade from **3a** to **3c** stereoisomers. In contrast, 15 out of 16 IReds could aminate *rac*-**3b** when methylamine was used as the amine donor. This interesting finding is described in detail in the next section.

Racemic (*E*)-3-methylpent-3-en-2-one (*rac*-**4b**) was also aminated only by an amine dehydrogenase (Ch1-AmDH) and an imine reductase (IRED-20) when we used ammonia as an amine donor. Ch1-AmDH produced an equal mixture of **4c** diastereomers and IRED-20 afforded 68:32 d.r., while the remaining compound was always unreacted **4b** (Figure 1E, entries 26 and 27; see Supporting Information, Tables S32 and S44, for the full dataset). Therefore, these two enzymes were selected to test the overall cascade from **4a** to **4c** stereoisomers. Like *rac*-**3b**, *rac*-**4b** was better accepted by the IReds when methylamine was used as an amine donor (eight out of 16 enzymes) instead of ammonia. The analysis of these results is reported in the next section.

### Asymmetric Reductive Amination of Saturated Ketones (**1–4b**) Using a Panel of Imine Reductases (IReds) to Give Secondary Amines (**1–4d**)

**Figure 2:**
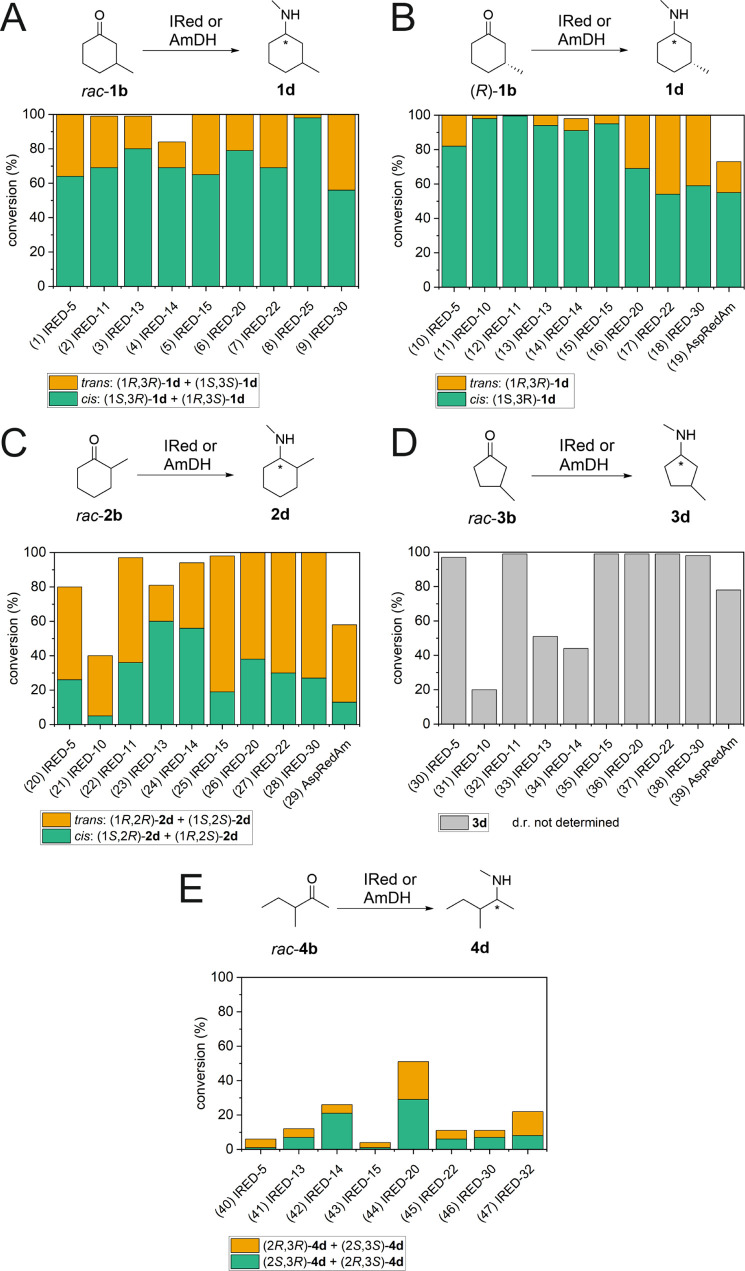
Study on the biocatalytic reductive amination of the saturated ketones (**1–4b**, 10 mM) using a broad panel of imine reductases (IReds, 20 μM) with methylamine as an amine donor. The reactions were conducted in HCOONH_3_CH_3_ buffer (1 M, pH 8.8) at a *T* of 30 °C for 24 h with a catalytic amount of either NADP^+^ or NAD^+^ (0.25 mM) and a suitable formate dehydrogenase (FDH 5 μM) for co-factor recycling.

The same panel of IReds and LE-AmDH-v1 was tested for the reductive amination of *rac*-**1b** with methylamine as an amine donor. The amine donor was again supplied as a component of the reaction buffer HCOONH_3_CH_3_ (1 M, pH 8.8). As observed for the reaction with ammonia as an amine donor, the absolute configuration of the β-carbon of **1b** influenced the stereoselectivity of the reductive amination. In particular, IRED-5, 11, 13, 15, 20, 22, 25, and 30 yielded quantitative conversion with variable ratios of cis/trans diastereomers. The formation of cis-**1d** prevailed over the formation of trans-**1d** in 14 out of 17 cases (Figure 2A, entries 1–9; see Supporting Information, Tables S4 and S45, for the full dataset). When we conducted the same reaction starting from (*R*)-**1b**, quantitative conversion was observed with most of the IReds (Figure 2B, entries 10–19; see Supporting Information, Tables S5 and S45, for the full dataset). However, only IRED-10 and IRED-11 yielded (1*S*,3*R*)-**1d** in high optical purity (98:2 d.r. or >99:<1 d.r; entries 11 and 12). The assignment of the absolute configuration of the enantiomers of **1d** was accomplished by following a strategy that was developed in this work and is described in the Supporting Information, Section 12.1.2 and Figure S5.

Based on the results from Supporting Information Tables S4 and S5, IRED-5, 10, 11, 13, 14, 15, 20, 22, 30, and AspRedAm were selected to test the overall cascade from **1a** to **1d** stereoisomers.

The reductive amination of *rac*-**2b** in HCOONH_3_CH_3_ was performed under the same reaction conditions as for the reductive amination of *rac*-**1b**. IRED-20, 22, and 30 yielded >99% conversion, while IRED-15 yielded 98% conversion (Figure 2C, entries 25–28). In all these cases, the trans-isomers (1*R*,2*R*, and 1*S*,2*S*) were prevalently formed, as previously observed for the amination of *rac*-**2b** with ammonia as an amine donor. IRED-5, 10, 11, 13, 14, and AspRedAm also yielded significant conversions (40–97%) (see Figure 2C and Supporting Information, Tables S14 and S45, for the full dataset). Notably, IRED-13 and 14 favored the formation of cis-**2d**, in contrast to all the other IReds (see Figure 2C, entries 23 and 24, and Supporting Information, Table S14, for a complete analysis). All the IReds depicted in Figure 2C were selected to test the overall cascade from **2a** to **2d** stereoisomers.

As briefly anticipated in the previous section, the reductive amination of *rac*-**3b** in HCOONH_3_CH_3_ was successful with most of the tested IReds, in contrast to the amination with ammonia as an amine donor. IRED-11, 15, 20, and 22 yielded quantitative conversion (Figure 2D, entries 32, 35, 36, and 37). IRED-5, 10, 13, 14, 30, and AspRedAm also yielded significant conversion (44–98%; see Figure 2D and Supporting Information, Tables S27 and S45, for the full dataset). Therefore, all these IReds were selected to test the overall cascade from **3a** to **3d** stereoisomers.

As anticipated in the previous section, the reductive amination of *rac*-**4b** in HCOONH_3_CH_3_ was also more successful than in HCOONH_4_ buffer. We observed conversion with eight IReds (4–51%), namely IRED-5, 13, 14, 15, 20, 22, 30, and 32, with again a non-equal diastereomeric ratio in all the cases (see Figure 2E and Supporting Information, Tables S33 and S45, for the full dataset). IRED-20 yielded the highest conversion (Figure 2E, entry 44). Either one of the two enantiomeric couples of **4d** was prevalently obtained depending on the IRed used. All the IReds except IRED-15 depicted in Figure 2E were selected to test the overall cascade from **4a** to **4d** stereoisomers.

### Biocatalytic Cascade Combining EReds and IReds for the Conversion of α,β-Unsaturated Ketones (**1–4a**) into Chiral Primary Amines (**1–4c**) Containing Two Stereogenic Centers

The cascade reaction from **1a** to give **1c** in high optical purity was first tested by combining OYE2, as one of the best-performing EReds for alkene moiety reduction (Table 1, entry 2), with the selected best-performing IReds for reductive amination (Figure 1A,B). We performed this one-pot concurrent cascade in the HCOONH_4_ buffer (1 M, pH 8.8) with **1a** at 30 °C for 24 h. The best combinations in terms of conversion and stereoselective outcome of the reaction were OYE2 with either IRED-10 or 22. Both combinations yielded (1*R*,3*S*)-**1c** in excellent conversions and stereoselectivities (Table 2, entries 1 and 2). For the full dataset with a detailed composition of the reaction mixtures, see Supporting Information, Tables S6 and S7. Notably, (1*R*,3*S*)-**1c** could previously be obtained at a maximum of 94:6 d.r. or 95:5 d.r. in two independently conducted studies using the more complex ω-transaminase-based amination system.^94,95^ Furthermore, the (1*R*,3*S*) stereoisomer of **1c** has not previously been obtained with any other cascade using dehydrogenase enzymes.^97^ Even the recently discovered EneIREDs were reported to produce a trans-configured stereoisomer (i.e., 1*R*,3*R*) for the reactions starting from 3-substituted cyclohexanone as substrates similar to **1a**.^99^ Therefore, the combinations of OYE2 with either IRED-10 or -22 are currently the only ones that can deliver (1*R*,3*S*)-**1c** (a cis-isomer) in excellent optical purity.

**Table 2:** Biocatalytic Cascades for the Conversion of α,β-Unsaturated Ketones (**1–4a**) Into Chiral Primary Amines (**1–4c**) Containing Two Stereogenic Centers by Combining Ene-Reductases (EReds) with Imine Reductases (IReds) or Amine Dehydrogenases (AmDHs)^a^

entry	substrate	ERed	IRed	product	conversion [%]^g^	d.r.^h^	e.r.
1	**1a**^b^	OYE2	IRED-10	(1*R*,3*S*)-**1c**	>99	97.0:3.0	99.8:0.2
2	**1a**^b^	OYE2	IRED-22	(1*R*,3*S*)-**1c**	>99	99.0:1.0	99.4:0.6
3	**1a**^c^	YqjM-v1	IRED-13	(1*S*,3*R*)-**1c**	97	98.5:1.5	96.8:3.2
4	**1a**^c^	YqjM-v1	IRED-15	(1*S*,3*R*)-**1c**	99	98.3:1.7	96.9:3.1
5	**1a**^c^	YqjM-v1	IRED-10	(1*S*,3*R*)-**1c**	>99	>99.9:<0.1	95.7:4.3
6	**1a**^c^	YqjM-v1	IRED-25	(1*S*,3*R*)-**1c**	>99	99.2:0.8	98.0:2.0
7	**2a**^d^	TOYE	IRED-22	(1*R*,3*R*)-**2c**	>99	89.2:10.8	98.0:2.0
8	**3a**^e^	OYE2	IRED-22	(1*R*,3*S*)-**3c**	44	95.0:5.0	>99:<1
9	**4a**^f^	XenA	Ch1-AmDH	(2*R*,3*S*)-**4c**	90	98.4:1.6	>99.8:<0.2
10	**4a**^f^	XenA	IRED-20	(2*S*,3*S*)-**4c**	51	79.6:20.4	97.8:2.2

a Reaction conditions varied depending on the substrates (see table footnotes and Supporting Information for details).

b **1a** (10 mM) was converted into (1*R*,3*S*)-**1c** using OYE2 (15 μM), IRed (40 μM), NADP^+^ (0.5 mM), and Cb-FDH-QRN (10 μM) in a one-pot concurrent two-step mode in HCOONH_4_ buffer (1 M, pH 8.8) at 30 °C for 24 h.

c **1a** (10 mM) was converted into (1*S*,3*R*)-**1c** as in (a) but with YqjM-v1 (20 μM) at 20 °C.

d **2a** (10 mM) was converted into (1*R*,3*R*)-**2c** in a one-pot sequential two-step mode; the first step was performed using TOYE (40 μM), Cb-FDH-QRN (10 μM), NADP^+^ (0.5 mM), and HCOONa (30 mM) in KPi buffer (pH 7, 50 mM) at 10 °C for 24 h to prevent the in situ racemization of the formed intermediate **2b**; the second step was performed upon addition of IRed (50 μM), Cb-FDH-QRN (10 μM), and NADP^+^ (0.5 mM) in HCOONH_4_ buffer (1 M, pH 7) at 20 °C for 24 h.

e **3a** (10 mM) was converted into (1*R*,3*S*)-**3c** using OYE2 (25 μM), IRed (50 μM), NADP^+^ (0.5 mM), and Cb-FDH-QRN (10 μM) in a one-pot concurrent two-step mode in HCOONH_4_ buffer (1 M, pH 8) at 30 °C for 24 h.

f **4a** (10 mM) was converted into (2*R*,3*S*)-**2c** or (2*S*,3*S*)-**2c** in a one-pot sequential two-step mode; the first step was performed using XenA (30 μM), Cb-FDH-QRN (5 μM), NADP^+^ (0.25 mM), and HCOONa (30 mM) in KPi buffer (pH 7.1, 50 mM) at 20 °C for 24 h to prevent the in situ racemization of the formed intermediate **2b**; the second step was performed upon addition of IRED-20 (50 μM) or Ch1-AmDH (100 μM), Cb-FDH-QRN or FDH (10 μM), and NADP^+^ or NAD^+^ (0.5 mM) in HCOONH_4_ buffer (1 M, pH 8.4) at 30 °C (IRED-20) or 50 °C (Ch1-AmDH) for 23 h.

g Measured by GC using an achiral column (DB-1701, 30 m, Agilent).

h Measured by GC using a chiral column (CP-Chirasil Dex-CB, Agilent; see Supporting Information, Section 13, for details).

Because the ene-reductase YqjM-v1 has the opposite stereoselectivity from that of OYE2 in the reduction of **1a** to **1b** (Table 1, entry 5), we investigated the cascades combining YqjM-v1 with the best-performing IReds for the reductive amination at 20 °C. The best combinations were YqjM-v1 combined with IRED-10, 13, 15, or 25 (Table 2, entries 3–6), thus leading to the quantitative conversion of (1*S*,3*R*)-**1c** (the other cis-isomer) with excellent d.r. and e.r. For the full dataset with a detailed composition of the reaction mixtures, see Supporting Information, Tables S6 and S7. (1*S*,3*R*)-**1c** is currently unattainable by combining EReds with ωTAs due to the lack of an ωTA possessing suitable stereoselectivity toward the saturated ketone intermediate.^94−96^ In a concomitant work, (1*S*,3*R*)-**1c** could be produced only at a maximum of 49% conversion using a native AmDH.^97^ In general, the scope of the ERed/IRed cascade for the conversion of **1a** to give access to both cis-isomers of **1c** in excellent d.r. and e.r. complements very well the documented trans-selectivity of the recently discovered EneIRED for 3-substituted cyclohexenones.^99^

The cascade for the conversion of **2a** into optically active **2c** turned out to be more challenging due to the previously described in situ racemization of the optically active intermediate **2b**, which can be minimized by reducing the temperature to 10 °C and changing the HCOONH_4_ buffer conditions (200 mM, pH 8; see Supporting Information, Tables S10 and S11, for details). As previously reported, the reduced temperature and buffer concentration led to a significant decrease in the catalytic activity of all the tested IReds (IRED-10, 13, 14, 15, 20, 22, and 25) in the cascade reaction with TOYE (see Supporting Information, Tables S15 and S16, for details). This resulted in a maximum of 32% total conversion with the trans-configured isomer (1*R*,2*R*)-**2c** as the major stereoisomer. Notably, the previously reported EneIRED enzymes favored the formation of a cis-isomer (i.e., (1*S*,2*R*)) in the case of the tested 2-substituted cyclohexenone substrate, thus again highlighting the complementarity of our ERed/IRed cascade approach in terms of stereoselectivity.^99^ However, aiming at improving the conversion, we decided to test the reaction from **2a** to **2c** in a one-pot two-stage cascade (i.e., a one-pot two-step separated in time). In the first stage of the one-pot cascade, TOYE, Cb-FDH-QRN, NADP^+^, HCOONa, and substrate **2a** were incubated in KPi buffer (1 mL, pH 7, 50 mM) at 10 °C for 24 h. In the next stage, HCOONH_4_ buffer (1 mL, 1 M, pH 7) containing IRed, Cb-FDH-QRN, and NADP^+^ was added directly to the previous solution and incubated at 20 °C for 24 h. The best combination in terms of conversion and stereoselectivities was TOYE with IRED-22 (Table 2, entry 7). For the full dataset with detailed procedures, see Supporting Information, Tables S17–S20.

For the cascade reaction from **3a** to **3c**, OYE2, rather than PETNR, was selected since the former ene-reductase yielded a higher conversion (see Table 1). The reaction was performed in a one-pot concurrent two-step mode; thus, the alkene reduction and reductive amination run simultaneously in this case. The combination of OYE2 with each of the IReds in HCOONH_4_ buffer (1 mL, 1 M, pH 8) led to measurable conversions only in two cases, namely OYE2 and IRED-20 or OYE2 and IRED-22 (Table 2, entry 8; Supporting Information, Table S28). All our efforts to analytically separate all the four possible stereoisomers of **3c** were unsuccessful. However, we could separate the two couples of enantiomers from each other, thus determining a d.r. of 95:5. We could then infer an e.r. value of >99:<1 based on the single alkene reduction step catalyzed by OYE2 (Table 1). The absolute configuration at the C-3 atom of **3c** was (*S*) according to the stereoselectivity of OYE2, while the configuration at the C-1 atom was assigned as (*R*) based on the determination of the stereoselectivity of IRED-22 in the cascade reaction with **3a** and methylamine (see the next section).

The last cascade from **4a** to **4c** again involved an α-substituted, α-chiral ketone intermediate (**4b**). Therefore, to minimize any possible in situ racemization of the **4b** intermediate along the cascade, we applied the one-pot sequential two-step approach. Thus, the alkene reduction was conducted at pH 7.1 in KPi buffer (50 mM, 1 mL) at 20 °C for 24 h. The lower temperature and the neutral pH precluded keto–enol tautomerization, thereby preserving the generated enantiomeric ratio in this step. XenA was used as the best ERed combined with Cb-FDH-QRN, NADP^+^, and HCOONa. To the same pot, HCOONH_4_ buffer (1 M, pH 8.4, 1 mL), an aminating enzyme, a co-factor (either NADP^+^ for IRED-20 or NAD^+^ for Ch1-AmDH), and a co-factor-recycling enzyme (either Cb-FDH-QRN for NADPH recycling or Cb-FDH for NADH recycling) were added, and the reaction was run for further 23 h. The full dataset and procedures are reported in Supporting Information, Tables S34 and S35. Notably, an increase in temperature from 30 to 50 °C was beneficial for the reaction with the thermostable Ch1-AmDH. In summary, starting from **4a**, both (2*R*,3*S*)-**4c** and (2*S*,3*S*)-**4c** were obtained with good to excellent conversion and stereoselectivity (Table 2, entries 9 and 10). Notably, the syntheses of (2*R*,3*S*)-**4c** and (2*S*,3*S*)-**4c** have never been achieved previously using either cascades with ωTAs or native AmDHs, or with EneIREDs.^47,94−97^

Finally, a semi-preparative scale synthesis of (1*R*,3*S*)-**1c** was accomplished from **1a** (30 mg) by combining OYE2 with IRED-22. The final product was obtained in 96% conversion, 70% yield, and high optical purity (99.5:0.5 e.r., 97:3 d.r.; Supporting Information, Section 12.1.4).

### Biocatalytic Cascade Combining EReds and IReds for the Conversion of α,β-Unsaturated Ketones (**1–4a**) into Chiral Secondary (i.e., *N*-Methyl) Amines (**1–4d**) Containing Two Stereogenic Centers

The synthesis of secondary amines starting from α,β-unsaturated ketones is not possible using EReds combined with either ωTA or AmDH as these enzymes can produce only primary amines.^25−29,36,37,39^ The formation of *N*-methyl secondary amines with an AmDH was documented in one of our publications, albeit with imperfect chemo- and stereo-selectivity and a constrained substrate scope.^121^ In contrast, IReds can effectively give access to secondary and even tertiary amines by reductive amination of ketones,^36−39,46−56^ a feature that we decided to explore in this work for the synthesis of secondary amines possessing two stereogenic centers. For this purpose, we first investigated the cascade reaction from **1a** to yield optically active **1d** by combining either OYE2 or YqjM-v1—as the best-performing EReds possessing opposite stereoselectivity for the reduction of **1a** to **1b** (Table 1, entries 1–7)—with each of the best-performing IReds for the reductive amination of ketone **1b** with methylamine as an amine donor (Figure 2A,B). The one-pot concurrent cascade was conducted in the HCOONH_3_CH_3_ buffer (1 M, pH 8.8) under the same conditions as described for the cascade from **1a** to **1c** in HCOONH_4_ buffer. Notably, (1*R*,3*S*)-**1d** was obtained in quantitative conversion and excellent stereoselectivity by combining OYE2 with AspRedAm as IRed (Table 3, entry 1). The combination of OYE2 with IRED-11 yielded the same product with a slightly lower conversion and d.r., albeit with the same e.r. (Table 3, entry 2). The other enantiomer product (1*S*,3*R*)-**1d** was also obtained in quantitative conversion and elevated stereoselectivity through the combination of YqjM-v1 with IRED-10 (Table 3, entry 3). The combination of YqjM-v1 with IRED-11 yielded the product with the same absolute configuration, again in quantitative yield, but a slightly lower d.r. and e.r. (Table 3, entry 4). For the full dataset, see Supporting Information, Tables S8 and S9. Notably, our ERed/IRed cascades afforded the synthesis of the two cis-configured stereoisomers of **1d** in excellent optical purity, which have not previously been synthesized. This result again highlights the complementarity between the ERed/IRed cascade and the reaction catalyzed by EneIRED, as the latter strategy always gives access to a trans-stereoisomer product (i.e., 1*R*,3*R*) in the conversion of 1,3-alkyl-substituted cyclohexenones to yield the related secondary amines.^99^

**Table 3:** Biocatalytic Cascades for the Conversion of α,β-Unsaturated Ketones (**1–4a**) Into Chiral Secondary (*N*-Methyl) Amines (**1–4d**) Containing Two Stereogenic Centers by Combining Ene-Reductases (EReds) with Imine Reductases (IReds)^a^

entry	substrate	ERed	IRed	product	conversion [%]^h^	d.r.^i^	e.r.
1	**1a**^b^	OYE2	AspRedAm	(1*R*,3*S*)-**1d**	>99	98.6:1.4	>99.9:<0.1
2	**1a**^b^	OYE2	IRED-11	(1*R*,3*S*)-**1d**	97	95.2:4.8	>99.9:<0.1
3	**1a**^c^	YqjM-v1	IRED-10	(1*S*,3*R*)-**1d**	>99	96.7:3.3	98.8:1.2
4	**1a**^c^	YqjM-v1	IRED-11	(1*S*,3*R*)-**1d**	>99	99.6:0.4	98.0:2.0
5	**2a**^d^	TOYE	IRED-5	(1*R*,3*R*)-**2d**	>99	96.9:3.1	98.5:1.5
6	**2a**^d^	TOYE	IRED-20	(1*R*,3*R*)-**2d**	>99	94.1:5.9	99.0:1.0
7	**2a**^d^	TOYE	IRED-30	(1*R*,3*R*)-**2d**	>99	94.1:5.9	98.6:1.4
8	**2a**^d^	TOYE	AspRedAm	(1*R*,3*R*)-**2d**	99	97.0:3.0	96.4:3.6
9	**2a**^d^	TOYE	IRED-11	(1*R*,3*R*)-**2d**	>99	96.2:3.8	98.8:1.2
10	**3a**^e^	OYE2	IRED-20	(1*R*,3*S*)-**3d**	99	98.9:1.1	>99:<1
11	**3a**^e^	OYE2	IRED-15	(1*S*,3*S*)-**3d**	56	96.0:4.0	>99:<1
12	**3a**^f^	OYE2	IRED-15	(1*S*,3*S*)-**3d**	>99	97.0:3.0	>99:<1
13	**4a**^g^	XenA	IRED-32	(2*S*,3*S*)-**4d**	47	99.4:0.6	>99.9:<0.1

a Reaction conditions varied depending on the substrates (see table footnotes and Supporting Information for details).

b **1a** (10 mM) was converted into (1*R*,3*S*)-**1d** using OYE2 (15 μM), IRed (40 μM), NADP^+^ (0.5 mM), and Cb-FDH-QRN (10 μM) in a one-pot concurrent two-step mode in HCOONH_3_CH_3_ buffer (1 M, pH 8.8) at 30 °C for 24 h.

c **1a** (10 mM) was converted into (1*S*,3*R*)-**1d** as in (a) but with YqjM-v1 (20 μM) at 20 °C.

d **2a** (10 mM) was converted into (1*R*,3*R*)-**2d** in a one-pot sequential two-step mode; the first step was performed using TOYE (40 μM), Cb-FDH-QRN (10 μM), NADP^+^ (0.5 mM), and HCOONa (30 mM) in KPi buffer (pH 7, 50 mM) at 10 °C for 24 h to prevent the in situ racemization of the formed intermediate **2b**; the second step was performed upon addition of IRed (50 μM), Cb-FDH-QRN (10 μM), and NADP^+^ (0.5 mM) in HCOONH_3_CH_3_ buffer (1 M, pH 7) at 20 °C for 24 h.

e **3a** (10 mM) was converted into (1*R*,3*S*)-**3d** or (1*S*,3*S*)-**3d** using OYE2 (25 μM), IRed (50 μM), NADP^+^ (0.5 mM), and Cb-FDH-QRN (10 μM) in a one-pot concurrent two-step mode in HCOONH_3_CH_3_ buffer (1 M, pH 8) at 30 °C for 24 h.

f **3a** (10 mM) was converted into (1*S*,3*S*)-**3d** in a one-pot sequential two-step mode; the first step was performed using OYE2 (25 μM), Cb-FDH-QRN (10 μM), and NADP^+^ (0.5 mM) in HCOONH_3_CH_3_ buffer (1 M, pH 8) at 30 °C for 24 h; next, IRED-15 (100 μM), Cb-FDH-QRN (10 μM), and NADP^+^ (0.5 mM) were added, and the reaction was incubated at 30 °C for 24 h.

g **4a** (10 mM) was converted into (2*S*,3*S*)-**4d** in a one-pot sequential two-step mode; the first step was performed using XenA (30 μM), Cb-FDH-QRN (5 μM), and NADP^+^ (0.5 mM) in HCOONH_3_CH_3_ buffer (1 M, pH 8) at 20 °C for 22 h; next, IRED-32 (50 μM), Cb-FDH-QRN (10 μM), and NADP^+^ (0.5 mM) were added, and the reaction was incubated at 30 °C for 26 h.

h Measured by GC using an achiral column (DB-1701, 30 m, Agilent).

i Measured by GC using a chiral column (CP-Chirasil Dex-CB, Agilent, for **1d** and **2d**; Hydrodex ß-TBDAc, Macherey-Nagel, for **4d**; see Supporting Information, Section 13, for details).

Based on the previously discussed results for the conversion of **2a** into **2c**, we again performed the biocatalytic cascade from **2a** to **2d** in a one-pot sequential two-step mode. The combination of TOYE with several IReds, namely IRED-5, 11, 20, 30, and AspRedAm, produced quantitative conversion giving (1*R*,2*R*)-**2d** as the main stereoisomer in excellent d.r. and e.r. (Table 3, entries 5–9). For the full dataset with detailed procedures, see Supporting Information, Tables S21–S24. A complementarity in the observed absolute configuration of the obtained product was again observed between the ERed/IRed cascade and the EneIRED enzyme. Our cascade yielded one trans-configured stereoisomer (i.e., (1*R*,2*R*)-**2d**), while EneIRED was reported in one case to yield a cis-configured 1,2 methyl-substituted secondary amine (i.e., the (1*S*,2*R*) configuration).^99^

As methylamine was generally better accepted than ammonia as an amine donor for the reductive amination of *rac*-**3b** with the IReds, the cascade from **3a** to **3d** with OYE2 led to successful conversions with more aminating enzymes, namely IRED-5, 10, 11, 13, 14, 15, 20, 22, 30, and AspRedAm (see Supporting Information, Table S29). In particular, the one-pot concurrent two-step reaction using OYE2 combined with IRED-20 led to the quantitative conversion of **3d** within 24 h. One major stereoisomer was detected with 99:1 d.r., while the e.r. was inferred to be >99:<1 from the previous alkene reduction step (Table 3, entry 10). Notably, the opposite diastereomer was obtained in 56% conversion and excellent stereoselectivity by combining OYE2 with IRED-15 (Table 3, entry 11). This latter conversion became quantitative by applying a one-pot two-step sequential protocol and increasing the concentration of IRED-15 (Table 3, entry 12; for details see Supporting Information, Table S30). In summary, the two cascades with OYE2 and IRED-20 or with OYE2 and IRED-15 led to two diastereomers with the same absolute configuration at the C-3 atom (*S*) and the opposite configuration at the C-1 atom. The absolute configuration of this latter carbon atom was determined by NOESY ^1^H NMR spectroscopic studies (see Supporting Information, Section 12.3.3, for an extended explanation), concluding that OYE2/IRED-20 and OYE2/IRED-15 led to (1*R*,3*S*)-**3d** (cis) and (1*S*,3*S*)-**3d** (trans), respectively.

The last cascade from **4a** to **4d** was performed following the one-pot sequential two-step approach to again minimize any possible in situ racemization of the α-substituted, α-chiral ketone intermediate **4b**. Although the control experiment for the reduction of the alkene moiety of **4a** catalyzed by XenA led to **4b** in 96:4 e.r., the overall cascade from **4a** to **4d** gave the final product with 89:11 e.r.; this signifies that partial racemization at the C-α to the carbonyl moiety occurred during reductive amination (for details, see Supporting Information, Table S36). Therefore, we investigated the single reduction of the alkene moiety catalyzed by XenA at pH 7 in either KPi or HCOOCH_3_NH_3_ buffer. The results showed that neither conversion nor stereoselectivity of the alkene reduction was influenced by a lower pH or a different buffer (for details, see Supporting Information, Table S37). The influence of the temperature was also negligible (see Supporting Information, Table S40). Several experiments with buffers at different pH values, varying the enzyme concentrations and testing both the concurrent and the sequential one-pot two-step approaches (see Supporting Information, Tables S38 and 39), led to the optimized conditions for this cascade. It was finally performed with a one-pot sequential two-step approach, both in the same HCOOCH_3_NH_3_ buffer (1 M, pH 8, 1 mL). In conclusion, **4a** was converted into (2*S*,3*S*)-**4d** in 47% conversion and excellent stereoselectivity (Table 3, entry 13).

Finally, in the context of NOESY experiments, semi-preparative scale syntheses of (1*R*,3*S*)-**3d** and (1*S*,3*S*)-**3d** were accomplished from **3a** (53–54 mg) by combining OYE2 with IRED-20 and 15, respectively. (1*R*,3*S*)-**3d** was obtained in 98% conversion, 99:1 d.r, and >99:<1 e.r., whereas (1*S*,3*S*)-**3d** was obtained in >99% conversion, 95:5 d.r., and >99:<1 e.r.

### Biocatalytic Cascade Combining EReds and IReds and Using Other Amine Donors for the Conversion of α,β-Unsaturated Ketones into Chiral Secondary and Tertiary Amines Containing Two Stereogenic Centers

Finally, we explored the synthetic potential of this one-pot cascade by testing ERed and IRed for the syntheses of chiral secondary and tertiary amines by using other amine donors, such as pyrrolidine (**d3**), cyclopropanamine (**d4**), allylamine (**d5**), and propargylamine (**d6**). All the four α,β-unsaturated ketones (**1–4a**) were tested as possible substrates for this cascade, and the reactions were performed following the one-pot sequential two-step approach. Therefore, the asymmetric reduction of the alkene moiety of substrates **1–4a** was conducted using the best-performing enzymes, as reported in Table 1, in KPi buffer (50 mM, pH 7.5, 1 mL) at 30 °C for 24 h. OYE2 and YqjM-v1 were used for the reactions with **1a**, whereas TOYE, OYE2, and XenA were used for the reactions with **2a**, **3a**, and **4a**, respectively. All these reactions yielded the respective products **1–4b** with conversions and enantiomeric excesses, as reported in Table 4. The reaction mixtures from the first step were directly used for the subsequent reductive amination step by directly adding a buffer solution containing each of the four amine donors (**d3–6**, 100 mM, pH 8.5–9.5, 1 mL) and each of the IReds from this study. The buffer with the amine donor and the IReds also contained NADP^+^, Cb-FDH-QRN, and HCOONa. In general, the final molar ratio between the substrate and the amine donor was 1 to 10. This ratio between the carbonyl compound acceptor and the amine donor is in the range of the catalytic activity reported for reductive aminase enzymes (see Supporting Information, Section 10, for details).^46^

**Table 4:** Biocatalytic Cascade for the Conversion of α,β-Unsaturated Ketones **1a** Into Chiral Secondary and Tertiary Amines (**1e–h**) Containing Two Stereogenic Centers by Combining Ene-Reductases (EReds) with Imine Reductases (IReds)^a^

entry	substrate	donor	ERed (step 1)	intermediate step 1	conversion [%]^b^	e.r.^c^	IRed (step 2)	product step 2^d^	conversion [%]^b^	d.r. [%]^e^
1	**1a**	**d3**	YqjM-v1	(*R*)-**1b**	>99	95:5	IRED-30	(1*X*,3*R*)-**1e**	30	97:3
2	**1a**	**d3**	YqjM-v1	(*R*)-**1b**	>99	95:5	IRED-5	(1*X*,3*R*)-**1e**	37	87:13
3	**1a**	**d3**	YqjM-v1	(*R*)-**1b**	>99	95:5	IRED-10	(1*Y*,3*R*)-**1e**	46	99.4:0.6
4	**1a**	**d3**	OYE2	(*S*)-**1b**	>99	>99:<1	IRED-32	(1*X*,3*S*)-**1e**	28	61:39
5	**1a**	**d3**	OYE2	(*S*)-**1b**	>99	>99:<1	IRED-14	(1*X*,3*S*)-**1e**	7	>99:<1
6	**1a**	**d4**	YqjM-v1	(*R*)-**1b**	>99	95:5	IRED-20	(1*X*,3*R*)-**1f**	60	92:8
7	**1a**	**d4**	YqjM-v1	(*R*)-**1b**	>99	95:5	IRED-10	(1*Y*,3*R*)-**1f**	81	98:2
8	**1a**	**d4**	OYE2	(*S*)-**1b**	>99	>99:<1	IRED-15	(1*X*,3*S*)-**1f**	63	98:2
9	**1a**	**d4**	OYE2	(*S*)-**1b**	>99	>99:<1	AspRedAm	(1*Y*,3*S*)-**1f**	84	99.9:0.1
10	**1a**	**d5**	YqjM-v1	(*R*)-**1b**	>99	95:5	IRED-30	(1*X*,3*R*)-**1g**	76	91:9
11	**1a**	**d5**	YqjM-v1	(*R*)-**1b**	>99	95:5	IRED-10	(1*Y*,3*R*)-**1g**	66	97:3
12	**1a**	**d5**	OYE2	(*S*)-**1b**	>99	>99:<1	IRED-15	(1*X*,3*S*)-**1g**	58	99.7:0.3
13	**1a**	**d5**	OYE2	(*S*)-**1b**	>99	>99:<1	AspRedAm	(1*Y*,3*S*)-**1g**	92	99.8:0.2
14	**1a**	**d6**	YqjM-v1	(*R*)-**1b**	>99	95:5	IRED-20	(1*X*,3*R*)-**1h**	32	91:9
15	**1a**	**d6**	YqjM-v1	(*R*)-**1b**	>99	95:5	IRED-30	(1*X*,3*R*)-**1h**	57	90:10
16	**1a**	**d6**	YqjM-v1	(*R*)-**1b**	>99	95:5	IRED-10	(1*Y*,3*R*)-**1h**	95	94:6
17	**1a**	**d6**	OYE2	(*S*)-**1b**	>99	>99:<1	Sp(S)-IRED	(1*X*,3*S*)-**1h**	22	99:1
18	**1a**	**d6**	OYE2	(*S*)-**1b**	>99	>99:<1	IRED-15	(1*X*,3*S*)-**1h**	53	98:2
19	**1a**	**d6**	OYE2	(*S*)-**1b**	>99	>99:<1	IRED-25	(1*X*,3*S*)-**1h**	31	99:1
20	**1a**	**d6**	OYE2	(*S*)-**1b**	>99	>99:<1	AspRedAm	(1*Y*,3*S*)-**1h**	82	99:1

a For experimental details, see Supporting Information, Section 10. Reaction conditions: in step 1, **1a** (10 mM), either OYE2 (15 μM) or YqjM-v1 (20 μM), NADP^+^ (0.5 mM), Cb-FDH-QRN (5 μM), and HCOONa (30 mM) in KPi buffer (50 mM, pH 7.5, 1 mL) at 30 °C for 24 h; in step 2, amine donors (**d3–6**, 100 mM, pH 8.5–9.5, 1 mL), IReds (40 μM), NADP^+^ (0.5 mM), Cb-FDH-QRN (5 μM), and HCOONa (30 mM).

b Measured by GC using an achiral column (DB-1701, 30 m, Agilent).

c Measured by GC using a chiral column (Rt-bDEXsm, Restek for **1a**, **3a**, and **4a**; Rt-bDEXsa, Restek for **2a**).

d The absolute configuration of C-1 was not determined; *X* and *Y* indicate the first and the second eluting diastereomer, respectively.

e Measured by GC–FID and GC–MS using an achiral column (DB-1701, 30 m, Agilent). The diastereomeric ratio values were reported with one significant decimal digit if the value was above 99:1; in the other cases, the value was rounded to the nearest integer number.

**Figure 3:**
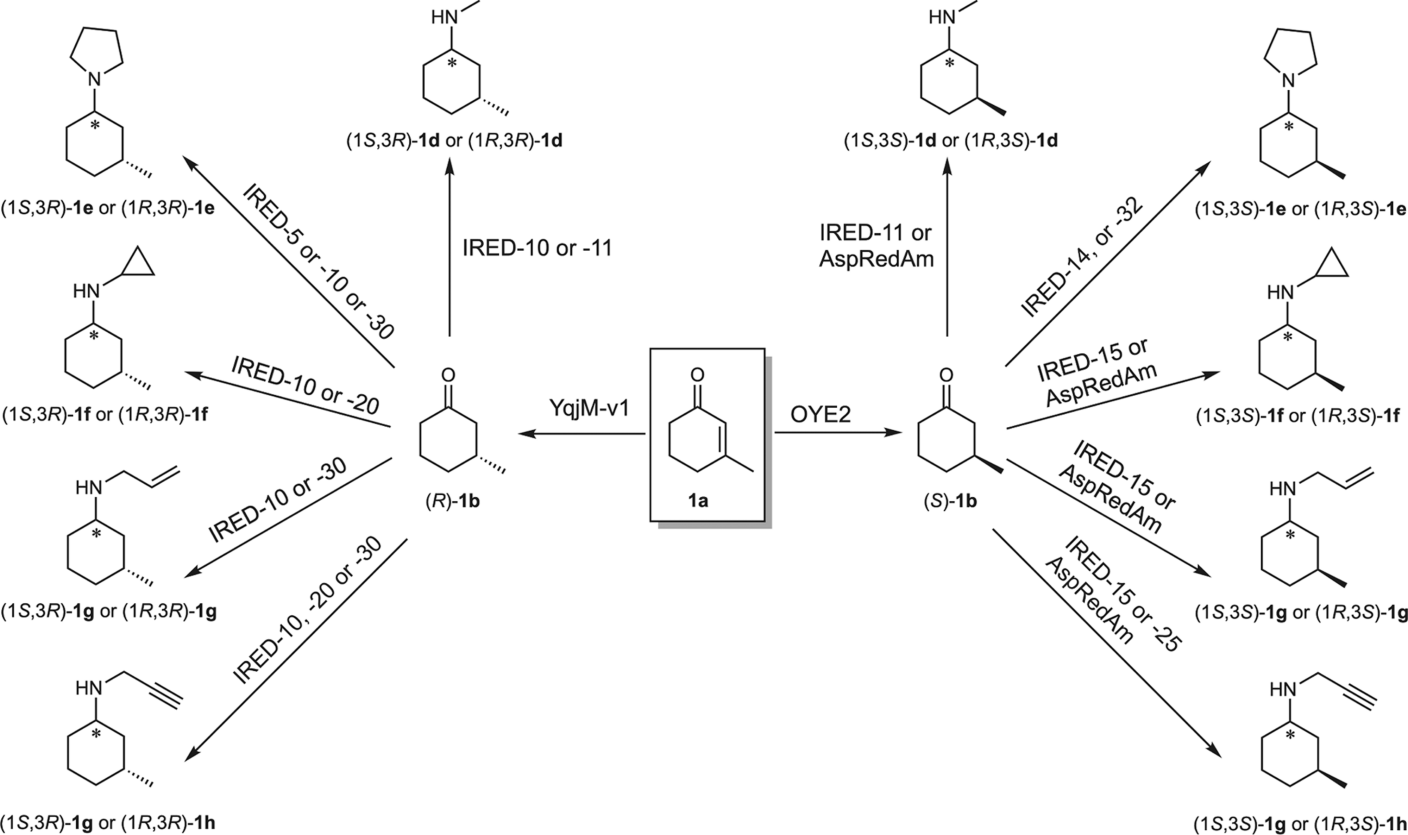
Illustration of the synthetic versatility of the dual-enzyme (ERed–IRed) approach reported in this study: synthesis of secondary and tertiary chiral amines possessing two stereogenic centers. All the possible combinations of the absolute configurations of the generated stereocenters were obtained.

**Figure 4:**
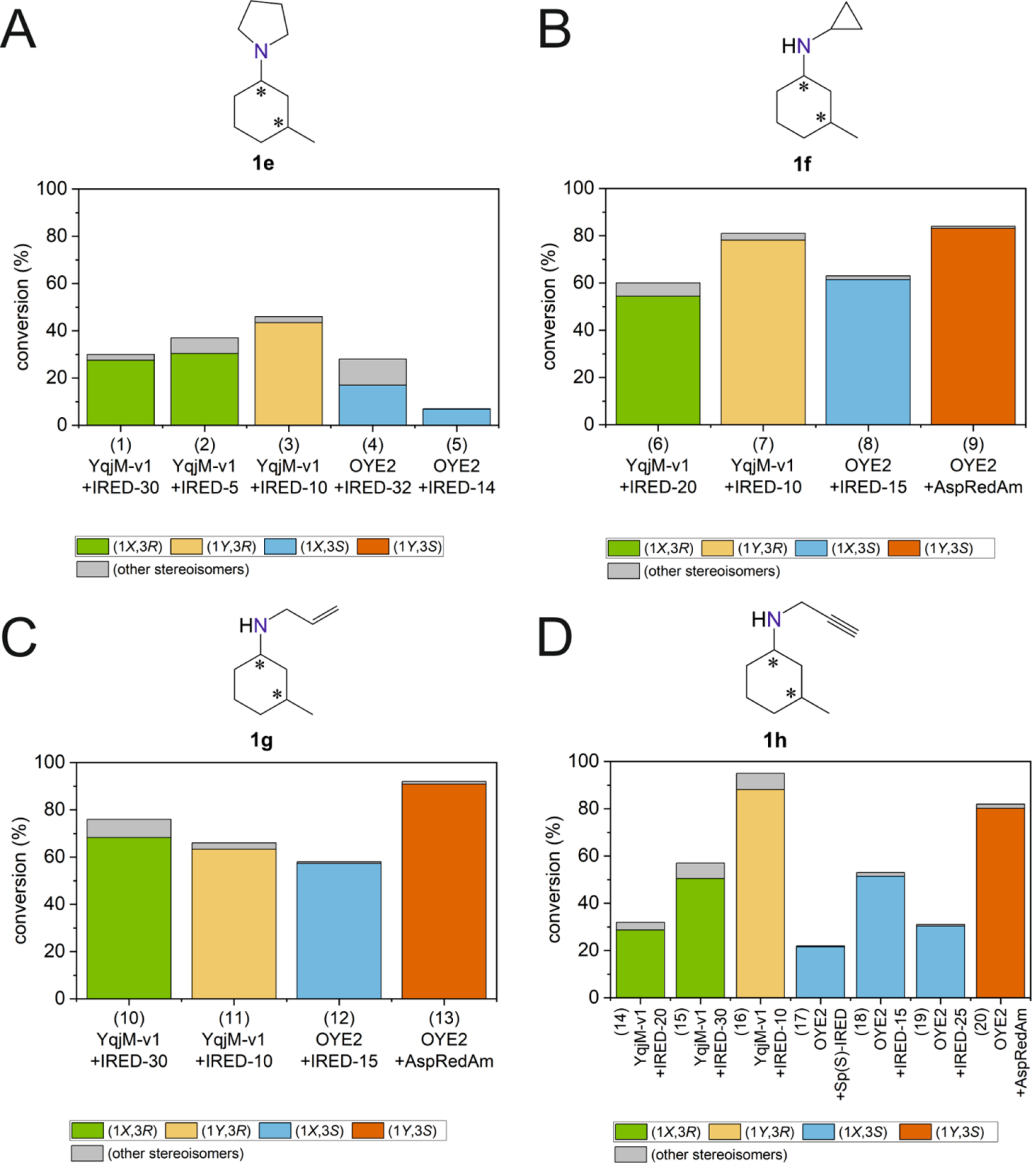
Biocatalytic cascades for the conversion of α,β-unsaturated ketones **1a** into chiral secondary and tertiary amines (**1e–h**) containing two stereogenic centers by combining ene-reductases (EReds) with imine reductases (IReds). Reactions with amine donors **d3** (A), **d4** (B), **d5** (C), and **d6** (D). For experimental details, see Supporting Information, Section 10. The absolute configuration of C-1 was not determined; *X* and *Y* indicate the first and the second eluting diastereomer, respectively.

Among the tested combinations, only the cascades starting from **1a** yielded the desired final amine products (Figure 3). Notably, we observed a typical trend regarding the acceptance of the intermediates (*R*)-**1b** or (*S*)-**1b** by the IReds. Regardless of the structure of the amine donor **d3–6**, (*R*)-**1b** was preferentially accepted by IRED-10, 20, and 30. Additionally, IRED-5 catalyzed the synthesis of 1-((3*R*)-3-methylcyclohexyl)pyrrolidine diastereomers (**1e**) with higher conversion, albeit with lower d.r. than IRED-30. In contrast, (*S*)-**1b** was preferentially accepted by IRED-15 and AspRedAm. The only exception was the synthesis of 1-((3*S*)-3-methylcyclohexyl)pyrrolidine diastereomers (**1e**), for which IRED-14 and 32 were the best-performing aminating enzymes. Considering the data for the reduction from **1a** to give (*R*)- or (*S*)-**1b** catalyzed by ERed and the data for the reductive amination catalyzed by IRed to give the final products **1e–h**, we observed that all the four possible stereoisomers were obtained in perfect or highly enriched optical purity in most of the cases. This fact exemplifies the stereochemical synthetic versatility of the dual-enzyme (ERed–IRed) approach because stereogenic centers with all of the different absolute configurations could be installed. This result has not previously been obtained for the synthesis of chiral amines bearing two stereogenic centers. For instance, the recently discovered EneIReds generally yield one of the trans-configured products starting from substrates such as **1a**.^99^Table 4 and Supporting Information Table S43 report the selected data and the full dataset for these one-pot sequential two-step cascades. Finally, Figure 4 depicts the composition of the product mixtures by highlighting the formation of the main stereoisomer versus all of the other stereoisomers.

## Conclusions

In this work, we have presented an enzymatic cascade that combined EReds with IReds/RedAms for the conversion of α,β-unsaturated ketones into primary, secondary, and tertiary amines containing two stereogenic centers in very high chemical purity, diastereomeric ratios, and enantiomeric ratios. It is noteworthy that the chemoselectivity of the reactions was also excellent when the cascades were run in a concurrent mode because the possible amino-alkene by-product was never observed. Compared with previously reported strategies, our strategy could access, in many cases, more or even all of the possible stereoisomers of the amine products while also avoiding the relevant formation of any side-product.^98^ This was possible due to the large pool of tested EReds and IReds and the thorough testing and optimization of the reaction conditions. Incidentally, the cascade reaction was also performed at a 30 mg scale for the synthesis of (1*R*,3*S*)-**1c** from **1a** (70% overall yield) and at a >50 mg scale for the synthesis of (1*S*,3*S*)-**3d** and (1*R*,3*S*)-**3d** from **3a** (98 to >99% conversion). Another important aspect was the utilization, for the first time in such reactions, of an NADP-dependent FDH for the in situ recycling of the NADPH co-factor. The FDH enables the atom efficiency of the reaction to be improved since the ammonium or alkylammonium buffer is also the source of the amine donor and reducing equivalents. Furthermore, FDH was found to be very stable under the required reaction conditions (e.g., high concentrations of ammonium or alkylammonium species and a wide range of pH values), which is not generally the case for the reported glucose dehydrogenases.^97^

Notably, our dual-enzyme ERed/IRed strategy exhibits a complementarity with the recently reported EneIRED enzymes for the synthesis of cyclic six-membered ring amines. While our ERed/IRed method afforded the synthesis of trans-1,2 and cis-1,3 substituted cyclohexylamines, the EneIRED method afforded the synthesis of one cis-1,2 and one trans-1,3 stereoisomer.^99^ Furthermore, as a proof of concept, we could obtain all four possible stereoisomers when 3-methylcyclohex-2-en-1-one (**1a**) was converted into secondary and tertiary chiral amines, thus highlighting the modularity and potential versatility of our approach. In general, this work highlights the potential of enzyme cascades for the highly selective synthesis of complex molecules possessing multiple stereogenic centers. Future research must focus on the engineering and discovery of more EReds and IReds possessing complementary stereoselectivity, improved stability, and a broader substrate scope. Such research efforts will elevate the impact of biocatalytic retrosynthesis on the sustainable synthesis of chiral organic molecules.^122−125^

## Supporting Information Available

Supporting Information contains the following: general information; procedures for enzyme expression and purification; general methods (with alkene moiety reductions, reductive aminations, and enzymatic synthesis of analytical reference compounds); experimental procedures and full datasets for biocatalytic reactions with substrates **1a,b**, substrates **2a,b**, substrates **3a,b**, and substrates **4a,b** with ammonia (**d1**) and methylamine (**d2**) as amine donors; experimental procedures and full datasets for biocatalytic reactions with amine donors **d3–6**; summary tables; chemical and chemo-enzymatic synthesis of reference compounds and scale-up enzymatic reductive amination, analytical methods, and representative GC chromatograms (PDF)
