# Supplementary material for: One-Pot Biocatalytic Synthesis of Primary, Secondary, and Tertiary Amines with Two Stereocenters from α,β-Unsaturated Ketones Using Alkyl-Ammonium Formate
Source: ACS Catal. 2022 Nov 10;12(23):14459–75. doi: 10.1021/acscatal.2c03052 (PMC9724091; doi:10.1021/acscatal.2c03052)
Supplement: Supplementary file 1 — cs2c03052_si_001.pdf [file cs2c03052_si_001.pdf]

Supporting Information for:

One-Pot Biocatalytic Synthesis of Primary, Secondary and Tertiary Amines with Two Stereocenters from α,β-Unsaturated Ketones using Alkylammonium Formate

Tanja Knaus[a]*, Maria Luisa Corrado[a] and Francesco G. Mutti[a]*

[a]*Van't Hoff Institute for Molecular Sciences, HIMS-Biocat, University of Amsterdam, Science Park 904, 1098 XH Amsterdam, (The Netherlands).*

* Corresponding authors: f.mutti@uva.nl; t.knaus@uva.nl;

Table of contents

1. Abbreviations 3
2. General information 3
3. Substrates and products used during this study, representative APIs, and comparative calculation of atom economy for the cascade. 3
4. Enzymes used in this study 6
5. Methods 7
6. Biocatalytic reactions performed for substrate 1a and 1b 9
   6.1. Substrate 1a - Stereoselective reduction of the double bond (ERED-Reaction) 9
   6.2. Substrate 1b - Amination 11
      *6.2.1. Substrate **1b** - Amination using ammonium formate/ammonia buffer 11*
      *6.2.2. Substrate **1b** - Amination using methylammonium formate/methylamine buffer 12*
   6.3. Substrate 1a – Cascade reaction 13
      *6.3.1. Substrate **1a** – Cascade reaction using ammonium formate/ammonia buffer 13*
      *6.3.1. Substrate **1a** – Cascade reaction using methylammonium formate/methylamine buffer 17*
7. Biocatalytic reactions performed for substrate 2a and 2b 20
   7.1. Substrate 2a - Stereoselective reduction of the double bond (ERED-Reaction) 20
   7.2. Substrate 2b - Amination 23
      *7.2.1. Substrate **2b** - Amination using ammonium formate/ammonia buffer 23*
      *7.2.2. Substrate **2b** - Amination using methylammonium formate/methylamine buffer 24*
   7.3. Substrate 2a – Cascade reaction 25
      *7.3.1. Substrate **2a** – Cascade reaction using ammonium formate/ammonia buffer 25*
      *7.3.2. Substrate **2a** – Cascade reaction using methylammonium formate/methylamine 28*
8. Biocatalytic reactions performed for substrate 3a and 3b 30
   8.1. Substrate 3a - Stereoselective reduction of the double bond (ERed-reaction) 30
   8.2. Substrate 3b - Amination 32
      *8.2.1. Substrate **3b** - Amination using ammonium formate/ammonia buffer 32*
      *8.2.2. Substrate **3b** - Amination using methylammonium formate/methylamine buffer 32*
   8.3. Substrate 3a – Cascade reaction 33
      *8.3.1. Substrate **3a** – Cascade reaction using ammonium formate/ammonia buffer 33*
      *8.3.2. Substrate **3a** – Cascade reaction using methylammonium formate/methylamine buffer 34*
9. Biocatalytic reactions performed for substrate 4a 39
   9.1. Substrate 4a - Stereoselective reduction of the double bond (ERed-reaction) 39
   9.1. Substrate 4b - Amination 41
      *9.1.1. Substrate **4b** - Amination using ammonium formate/ammonia buffer 41*
      *9.1.1. Substrate **4b** - Amination using methylammonium formate/methylamine buffer 41*

9.1. Substrate 4a – Cascade reaction 42
*9.1.1. Substrate **4a** – Cascade reaction using ammonium formate/ammonia buffer ... 42*
*9.1.2. Substrate **4a** – Cascade reaction using methylammonium formate/methylamine buffer ... 44*
10. Biocatalytic reactions using further amine donor ... 50
11. Summary of results as depicted in Figures 1 and 2, main manuscript. ... 55
12. Synthesis of reference compounds ... 57
12.1. Substrates 1a-d 57
*12.1.1. References for **1c** using ωTAs on analytical scale ... 57*
12.1.2. Chemo-enzymatic strategy for the assignment of the absolute configuration of α-chiral secondary amines 59
*12.1.3. Chemical synthesis of rac-**1d** ... 59*
*12.1.4. References for **1d** – chemo-enzymatic synthesis ... 62*
12.1.5. Representative NMRs of final product: ... 70
12.2. Substrates 2a-d 73
*12.2.1. References for **2c** using ωTAs on analytical scale ... 73*
*12.2.2. Chemical synthesis of rac-**2d** ... 76*
12.3. Substrate 3a-d 78
*12.3.1. References for **3c** with ωTAs on analytical scale ... 78*
*12.3.2. Biocatalytic reaction to synthesize enantiopure **3d** on semi-preparative scale by combining ERed and IRed. 81*
*12.3.3. NMR measurements of enantiopure **3d** produced by biocatalytic reaction on semi-preparative scale ... 83*
12.4. Substrate 4a-d 93
*12.4.1. Reference for **4b** - Identification of the enantiomers using optical rotation ... 93*
*12.4.2. References for **4c** with ωTAs on analytical scale ... 95*
*12.4.3. References for **4c** with ωTAs on semi-preparative scale ... 97*
*12.4.4. References for **4d** - chemo-enzymatic synthesis ... 99*
13. Analytical methods ... 101
13.1. Achiral GC-FID measurement 101
13.2. Chiral GC-FID measurements for the separation of the saturated intermediates 1b-4b 102
13.1. Chiral GC measurements for the separation of the diastereomers 103
14. Representative GC-chromatograms ... 104
14.1. Representative achiral GC-chromatograms 104
14.2. Representative chiral GC-FID chromatograms for the best performing reactions 106
14.2.1. Chiral primary amines ... 106
14.2.2. Chiral secondary (N-methyl)amines ... 109
14.3. GC-FID chromatograms for substrate 3 111
*14.3.1. Cascade reaction combining **3a** with OYE2 and IRED22 (ammonium formate buffer) ... 111*
*14.3.1. Cascade reaction combining **3a** with OYE2 and IRED20 and IRED15 (methylammonium formate buffer) . 112*
15. Literature ... 115

1. Abbreviations

ERed ene reductase

IRed imine reductase

RedAm Reductive aminase

AmDH amine dehydrogenase

FDH formate dehydrogenase

n.d. not detected

n.m. not measured

DCM dichloromethane

2. General information

3-methyl-2-cyclohexen-1-one (**1a**, >98% purity), (*R*)-(+)-3-methylcyclohexanone ((*R*)-**1b**, 98%), 2-methyl-2-cyclohexen-1-one (**2a**, >90%), 2-methylcyclohexanone (**2b**, 99%), 3-methyl-2-cyclopentenone (**3a**, 97%), 3-methylcyclopentanone (**3b**, 99%), (*R*)-(+)-3-methylcyclopentanone ((*R*)-**3b**, 99%) and 3-methyl-2-pentanone (**4b**, 99%), ammonium hydroxide solution (**d1**, 32%), methylamine solution (**d2**, 40%), pyrrolidine (**d3**, 99%), cyclopropylamine (**d4**, 98%) allylamine (**d5**, 98%) propargylamine (**d6**, 98%) were purchased from Merck, whereas 3-methylcyclohexanone (**1b**, >97%), 3-methylcyclohexylamine (**1c**, >98%), 2-methylcyclohexylamine (**2c**, >95%), 3-methyl-3-penten-2-one (**4a**, >95%) from TCI chemicals. $NADP^+$ and NAD^+ were purchased from Melford Biolaboratories (Chelsworth, Ipswich, UK).

3. Substrates and products used during this study, representative APIs, and comparative calculation of atom economy for the cascade.

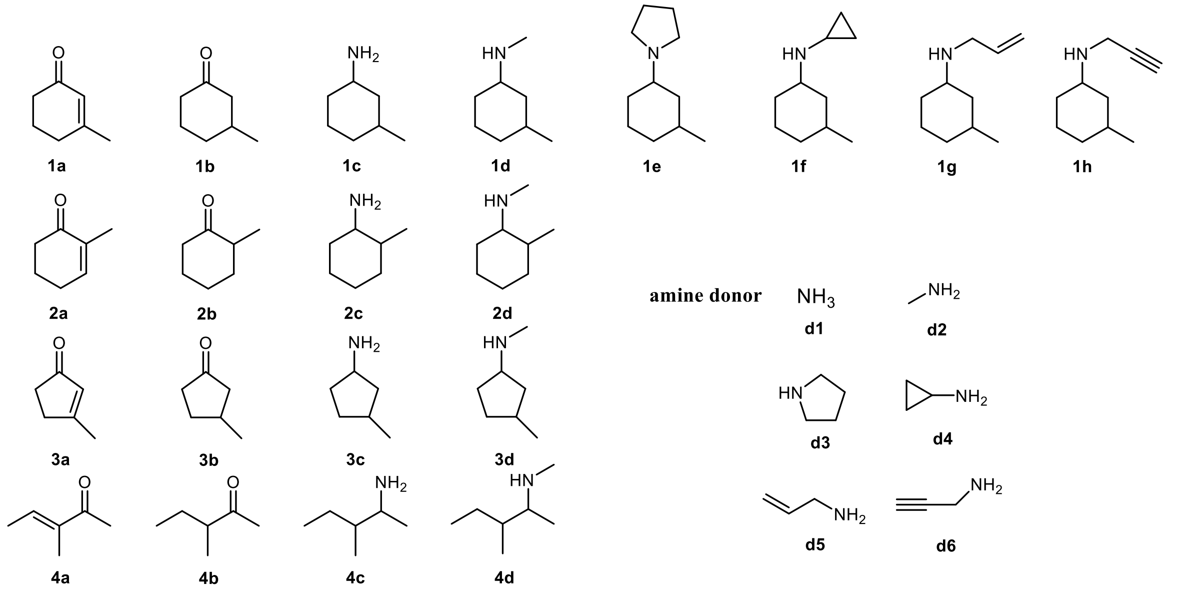

Figure S1. Substrates, intermediates and products used in this study.

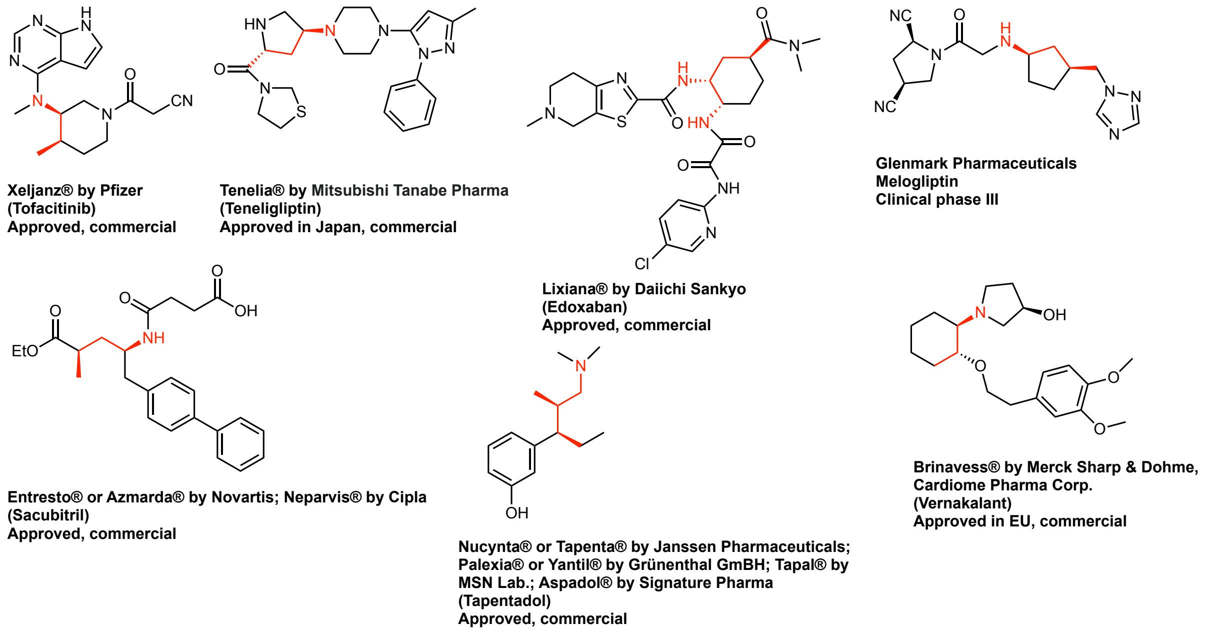

Figure S2. Selection of APIs containing multiple stereogenic centres that can be installed starting from prochiral precursors by combining the chemical reactivities of ene-reductases (EReds) with imine reductases (IReds) or reductive aminases (RedAms) or amine dehydrogenases (AmDHs). Compounds (1), (2) and (3) were already listed in the "top-200 pharmaceuticals for global revenues in 2020" (compiled by Njardarson's goup at University of Arizona, USA).

Comparative calculation of atom economy

The atom economy or atom efficiency is calculated by dividing the molecular weight of the product by the sum total of the molecular weight of all substances formed in the stoichiometric equation for the reaction involved. [1] The atom efficiency calculation assumes a yield of 100% and exactly stoichiometric amounts and disregards substances which do not appear in the stoichiometric equation.

For this comparative calculation, we have considered the systems that recycle NADPH using GDH/glucose (e.g., Scheme 1B, main paper) and FDH/formate (e.g., Scheme 1C, main paper).

a) GDH/glucose system

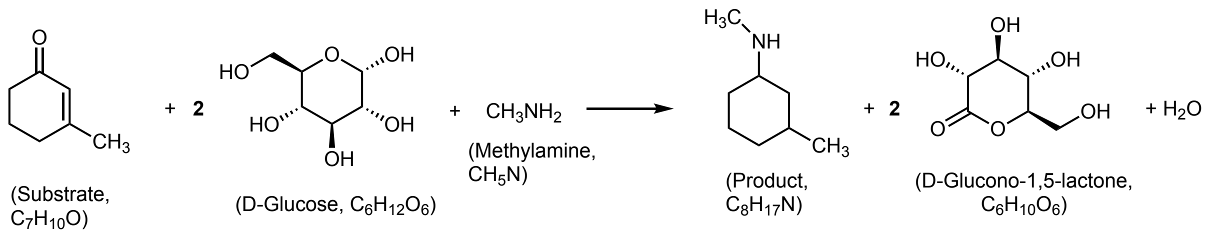

	MW (g mol⁻¹)	Stoichiometry	Total
Product	127.23	1	127.23
D-glucono-1,5-lactone	178.14	2	356.28
Water	18.02	1	18.02

Atom Economy: [127.23 / (127.23 + 356.28 + 18.02)] * 100 = 25%

b) FDH/formate system

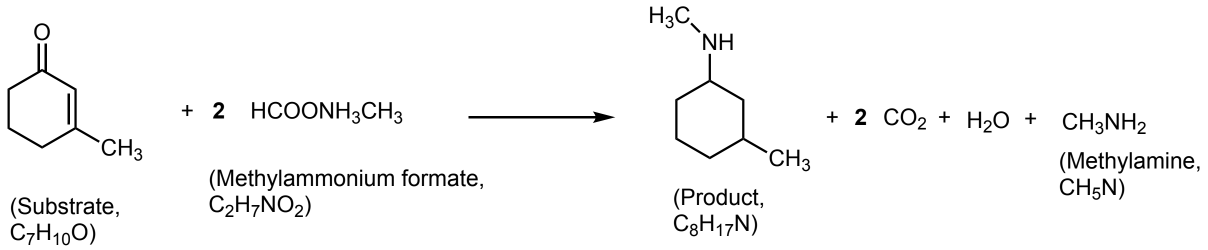

	MW (g mol-1)	Stoichiometry	Total
Product	127.23	1	127.23
Carbon dioxide	44.01	2	88.02
Water	18.02	2	36.04
Methylamine	31.06	1	31.06

Atom Economy: [127.23 / (127.23 + 88.02 + 36.04 + 31.06)] * 100 = 45%

4. Enzymes used in this study

(1) Ene reductases: PETNR (*Enterobacter cloacae*), TOYE (*Thermoanaerobacter pseudethanolicus*), OYE2 (*Saccharomyces cerevisiae*), OYE3 (*Saccharomyces cerevisiae*), XenA (*Pseudomonas putida*), XenB (*Pseudomonas fluorescens*), LeOPR1 (*Lycopersicon esculentum*), NerA (*Agrobacterium radiobacter*), GluOx (*Gluconobacter oxydans*), YqjM (*Bacillus subtilis*), MR (*Pseudomonas putida*), YqjM-v1 (variant originated from the ER from *Bacillus subtilis*)

The ene reductases have been expressed and purified as described as in our previous publication. [2] All proteins were stored as concentrated stocks in KPi buffer (50 mM, pH 7.0). YqjM-v1 is the Cys26Asp Ile69Thr variant as described in the paper by Skalden et. al. [3]. It was expressed and purified in the same way as the wild-type enzyme.

(2) Imine reductases: Sp(S)-IRED (*Streptomyces* sp. GF3546) [4], IRED1 (*Streptomyces* sp. Mg1), IRED5 (Cupriavidus sp.), IRED13 (*Saccharopolyspora erythraea* NRRL 2338), IRED14 (*Nocardia cyriacigeorgica* GUH-2), IRED15 (*Actinomadura rifamycini*), IRED20 (*Streptomyces tsukubaensis*), IRED21 (*Paenibacillus ehimensis*), IRED22 (*Streptomyces sp*. CNH287), IRED25 (*Paenibacillus sp*. HGF5), IRED30 (*Nitratireductor pacificus*), IRED32 (*Aeromonas veronii*), IRED7 (*Streptomyces aurantiacus*), IRED10 (*Mycobacterium smegmatis*), IRED11 (*Verrucosispora maris*). With the exception of Sp(S)-IRED, the information for the other imine reductases can be found in the publications by Wetzl et. al. [5] and [6]

Expression of the IREDs: For recombinant expression, 800 mL of LB medium supplemented with the appropriate antibiotic (50 µg mL^{-1} kanamycin) were inoculated with 15 mL of an overnight culture harboring the desired vector with genes for the expression of the enzymes. *E. coli* BL21 DE3 was used as host organism. Cells were grown at 37 °C until an OD600 of 0.6 to 1 was reached and expression of protein was induced by the addition of IPTG (final concentration 0.5 mM). Protein expression was carried out overnight (25 °C) and after harvesting of the cells (4 °C, 4500 rpm, 10 min), the remaining cell pellet was frozen.

Purification of the IREDs by Nickel affinity chromatography: The His_6-tagged proteins were resuspended in lysis buffer (50 mM KH_2PO_4, 300 mM NaCl, 10 mM imidazole, pH 8.0) prior to cell disruption, and protein purification was performed by Ni-NTA affinity chromatography using pre-packed Ni-NTA HisTrap HP columns (GE Healthcare), previously equilibrated with lysis buffer. After loading of the filtered lysate, the column was washed with sufficient amounts of wash buffer (50 mM KH_2PO_4, 300 mM NaCl, 25 mM imidazole, pH 8.0), and bound protein was recovered with elution buffer (50 mM KH_2PO_4, 300 mM NaCl, 200 mM imidazole, pH 8.0). Purity was analyzed by SDS-PAGE and fractions showing >95% purity were combined and dialyzed overnight against KPi buffer (50 mM, pH 8 for Sp(S)-IRED, IRED13, IRED15, IRED20, IRED22, IRED7, IRED10, IRED11 and IRED25 or pH7 for the remaining IREDs) containing $MgCl_2$ (1 mM). The enzyme solutions were concentrated and their concentration was determined spectrophotometrically based on their extinction coefficient at 280 nm.

(3) Reductive aminases and amine dehydrogenases: AspRedAm (*Aspergillus oryzae*), Ch1-AmDH (chimeric amine dehydrogenase generated through domain shuffling of Bb-PhAmDH variant and L-AmDH variant, the latter originated from the L-leucine dehydrogenase from *Bacillus stearothermophilus*.), Rh-PhAmDH (amine dehydrogenase variant originated from the L-phenylalanine dehydrogenase from *Rhodoccoccus species*) and LE-AmDH-v1 (variant of NADH dependent ε-deaminating L-lysine dehydrogenase from *Geobacillus sterothermophilus*).

The AmDHs were expressed and purified as previously described. [7] [8] [9] [10]

(4) Formate dehydrogenases NADH dependent (Cb-FDH) and NADPH dependent (FDH-QRN)

Cb-FDH (*Candida boidinii*) was expressed and purified as previously described. [9] FDH-QRN (variant of the formate dehydrogenase from *Candida boidinii*) [11] was expressed and purified in the same way as Cb-FDH. The gene encoding for the protein was subcloned in pET15b between NdeI and XhoI, therefore it contains ampicillin instead of kanamycin resistance.

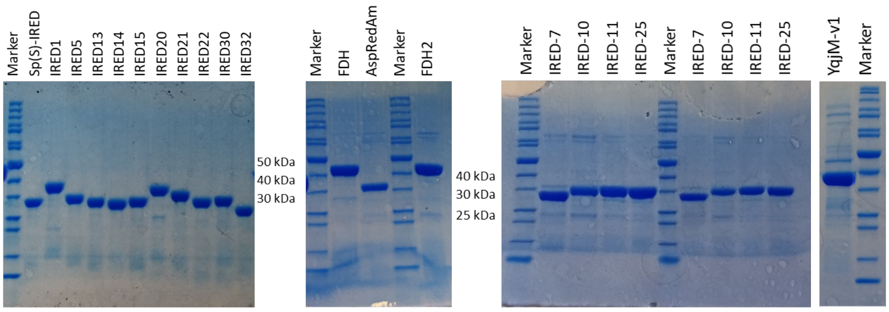

Figure S3. SDS-Page of the purified proteins. Marker: PageRuler Unstained Protein Ladder (Thermo Scientific)

Below there is a summary of the enzymes used in this study and their primary literature references.

enzyme name	organism	reference
PETNR	*Enterobacter cloacae*	12
TOYE	*Thermoanaerobacter pseudethanolicus*	13
OYE2	*Saccharomyces cerevisiae*	14
OYE3	*Saccharomyces cerevisiae*	14
XenA	*Pseudomonas putida*	15
XenB	*Pseudomonas fluorescens*	15
LeOPR1	*Lycopersicon esculentum*	16
NerA	*Agrobacterium radiobacter*	17
GluOx	*Gluconobacter oxydans*	18
YqjM	*Bacillus subtilis*	19
MR	*Pseudomonas putida*	20
YqjM-v1	variant originated from the ER from *Bacillus subtilis*	3
Sp(S)-IRED	*Streptomyces* sp. GF3546	4
IRED1	*Streptomyces* sp. Mg1	5
IRED5	*Cupriavidus* sp.	5
IRED13	*Saccharopolyspora erythraea* NRRL 2338	5
IRED14	*Nocardia cyriacigeorgica* GUH-2	5
IRED15	*Actinomadura rifamycini*	5
IRED20	*Streptomyces tsukubaensis*	5
IRED21	*Paenibacillus ehimensis*	5
IRED22	*Streptomyces sp*. CNH287	5
IRED25	*Paenibacillus sp*. HGF5	6
IRED30	*Nitratireductor pacificus*	6
IRED32	*Aeromonas veronii*	6
IRED7	*Streptomyces aurantiacus*	5
IRED10	*Mycobacterium smegmatis*	5
IRED11	*Verrucosispora maris*	5
AspRedAm	*Aspergillus oryzae*	7
Ch1-AmDH	chimeric amine dehydrogenase generated through domain shuffling of Bb-PhAmDH variant and L-AmDH variant, the latter originated from the L-leucine dehydrogenase from *Bacillus stearothermophilus*	21
Rh-PhAmDH	amine dehydrogenase variant originated from the L-phenylalanine dehydrogenase from *Rhodococcus species*	22
LE-AmDH-v1	variant of NADH dependent ε-deaminating L-lysine dehydrogenase from *Geobacillus sterothermophilus*	10
Cb-FDH	*Candida boidinii*	23
FDH-QRN	variant of the formate dehydrogenase from *Candida boidinii*	11

5. Methods

__Preparation of solutions for the amine donor:__ **(a) ammonium formate buffer ($HCOONH_4/NH_3$) 1 M**: ammonium formate (NH_4HCO_2, Merck, 63.06 g/mol, 99% purity; final concentration 0.945 M) was dissolved in H_2O. Then, ammonium hydroxide solution (32%, 8.1 M, Merck; final concentration 0.055 M) was added and the solution was filled up with H_2O to the desired volume. The pH was 8.8 and adjusted if necessary with formic acid. **(b) methylammonium formate buffer ($HCOOCH_3NH_3/CH_3NH_2$) 1M**: methylamine solution (40%, 11.5 M) was diluted with H_2O to 1 M and the pH adjusted with formic acid. **(c) amine donor d3-d6**: 100 mM of the amine donor **d3**-**d6** were dissolved in KPi buffer (200 mM, pH 7.5) and the pH corrected with phosphoric acid.

__General__: In the biocatalytic reactions on analytical scale, the substrate was always added as a stock solution in DMSO (1 M). The concentrations of the reagents in the biocatalytic reactions varied and are listed for every single experiment in the corresponding paragraph.

<u>ERed reactions</u>: the biocatalytic reactions contained buffer, ERed, FDH-QRN, $NADP^+$ (or Cb-FDH combined with NAD^+), sodium formate and substrate. The reactions were incubated on a horizontal shaker before the organic compounds were extracted.

<u>Aminating reaction</u>: the biocatalytic reaction consisted of buffer, aminating enzyme, FDH-QRN, $NADP^+$ and substrate. The reactions were incubated on a horizontal shaker before the organic compounds were extracted.

<u>Work up of the biocatalytic reactions</u>: The biocatalytic reactions were basified with KOH (10 M, 100 µL to 1 mL reaction, only when the amine was formed), extracted with MTBE (2 x 500 µL), dried over $MgSO_4$ and the reaction products were verified by GC-MS.

<u>Chiral GC measurements</u>: (i) Derivatization of the amines with DMAP/acetic anhydride: to 250 µL organic phase (substrate concentration 10 mM), DMAP (5 µL, 50 mg/mL in acetic anhydride) were added. The vials were incubated for 30 min at 30 °C. H_2O was added (125 µL) and the mixture was incubated again (30 min, 30 °C). After centrifugation, the organic phase was dried over $MgSO_4$. (ii) Derivatization with benzoic anhydride/DMAP: benzoic anhydride (57.5 mg) and DMAP (15.3 mg) were dissolved in THF (250 µL). 20 µL were added into the biocatalytic reactions. They were incubated at 30 °C for 30 min. Then, $KHCO_3$ was added (1 mL of 10% solution) and incubated at 30 °C for further 30 min. After separation of the two layers, the organic phase was dried over $MgSO_4$.

<u>Synthesis of enantiopure reference compounds with ωTAs</u>: The synthesis of the primary amine reference compounds was done with the already studied ω-TAs ((*S*)-selective: Vf-ωTA from *Vibrio fluvialis* and Cv-ωTA from *Chromobacterium violaceum.* (*R*)-selective: As(R)-ωTA from *Arthrobacter* sp., As(R)mut11-ωTA variant of the former, At-ωTA from *Aspergillus terreus* and Hn-ωTA from *Hyphomonas neptunium*) as described elsewhere. [24] [25] [26]

Two different reaction set-ups were performed: (a, c) using the saturated racemic intermediate in combination with the ωTA and (b, d) using the unsaturated starting material in combination with an ERED and an ωTA.

<u>(a) saturated intermediate with ωTA</u>: A 1 mL reaction volume (in 2 mL Eppendorf tubes) consisted of buffer (KPi, 50 mM, pH 7.0) substrate (10 mM, added as 1 M stock in DMSO), ωTA (20 mg/mL, lyophilized powder), PLP (1 mM), alanine (50 mM, 5 equiv., L-Ala for *S*-selective and D-Ala for *R*-selective ωTAs), LDH (1 mg/mL), Cb-FDH (10 µM) and NAD^+ (0.5 mM). The biocatalytic reactions were run at 30 °C for 24 h in an orbital shaker and stopped by the addition of KOH (10 M, 100 µL); the reaction products were extracted with MTBE (2 x 500 µL) and dried over $MgSO_4$.

<u>(b) unsaturated starting material with ERed and ωTA in two sequential steps</u>: (i) 1 mL reaction volume (in 2 mL Eppendorf tubes): Buffer (KPi, 50 mM, pH 7.0), substrate (10 mM, added as 1 M stock in DMSO), ERed (20 µM, OYE2 for **1a**, TOYE for **2a**, PETNR for **3a**, XenA for **4a**), NAD^+ (0.25 mM), Cb-FDH (10 µM), HCOONa (30 mM). The reactions were run for 24 h at 30 °C. (ii) total reaction volume is 1.5 mL: addition of ωTA (20 mg lyophilized powder), PLP (1 mM), alanine (50 mM, 5 equiv., L-Ala for *S*-selective and D-Ala for *R*-selective ωTAs), LDH (1 mg/mL), Cb-FDH (10 µM), NAD^+ (0.5 mM). The transamination reactions were run at 30 °C for 24 h, then basified with KOH (10 M, 100 µL); the reactions were split in two Eppendorf tubes, and each tube was extracted with MTBE (1 x 500 µL). Finally, the organic layers were combined and dried over $MgSO_4$.

<u>(c) saturated intermediate with As(R)mut11-ωTA</u>: A 1 mL reaction volume (in 2 mL Eppendorf tubes) consisted of 2-propylamine (1 M, pH 11), PLP (0.5 mM), As(R)mut11-ωTA (20 mg/mL, as lyophilized cells) and substrate (10 mM, added as 1 M stock in DMSO). The reactions were run at 45 °C for 24 h, and the reaction products were extracted with MTBE (2 x 500 µL) and dried over $MgSO_4$.

<u>(d) unsaturated starting material with ERED and As(R)mut11-ωTA in two sequential steps</u>: (i) 1 mL reaction volume (in 2 mL Eppendorf tubes): Buffer (KPi, 50 mM, pH 7.0), substrate (10 mM, added as 1 M stock in DMSO), ERed (20 µM, OYE2 for **1a**, TOYE for **2a**, PETNR for **3a**, XenA for **4a**), NAD^+ (0.25 mM), Cb-FDH (10 µM), HCOONa (30 mM). The reactions were run for 24 h at 30 °C. (ii) total reaction volume is 1.5 mL: addition of 2-propylamine (1.5 M, pH 11), As(R)mut11-ωTA (20 mg), PLP (0.5 mM). The reactions were run at 45 °C for 24, then extracted with MTBE (2 x 500 µL) and dried over $MgSO_4$.

6. Biocatalytic reactions performed for substrate 1a and 1b

6.1. Substrate 1a - Stereoselective reduction of the double bond (ERED-Reaction)

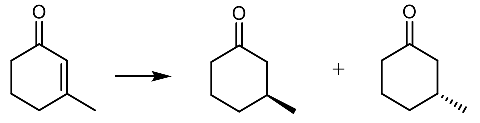

1a (*S*)-**1b** (*R*)-**1b**

Reaction conditions: 1 mL total volume in 2 mL Eppendorf tubes. Buffer KPi (50 mM, pH 8.0) or ammonium formate/ammonia buffer (1 M, pH see Table S1) or methylammonium/methylamine buffer (1 M, pH see Table S1), $NADP^+$ (0.25 mM), sodium formate (30 mM, only for KPi buffer), FDH-QRN (5 µM), ERed (5 µM or 10 µM[(a)], or 20 µM[(b)]), substrate **1a** (10 mM), T= 10–30 °C, time= 20 h. NerA and MR were also tested with NAD^+ (0.25 mM) in combination with Cb-FDH (5 µM).

Table S1. Conversion and *e.r.* for **1a** using EREDs.

enzyme	T (°C)	buffer	pH	**1b**	**1a**	phenol deriv (**1b'**) [d]	*e.r.* **1b**
Blank	30	KPi	8	n.d.	>99	n.d.	n.m.
TOYE	30	KPi	8	1	96	3	n.m.
OYE2	30	KPi	8	97	3	n.d.	>99.8:<0.2 (S)
OYE2[(a),[c]]	30	$HCOONH_4/NH_3$	9	85	11	4	>99.8:<0.2 (S)
OYE2[(a),[c]]	30	$HCOOCH_3NH_3/CH_3NH_2$	9	84	12	4	>99.8:<0.2 (S)
OYE3	30	KPi	8	23	77	n.d.	>99.5:<0.5 (S)
XenA	30	KPi	8	1	86	13	n.m.
XenB	30	KPi	8	33	67	n.d.	>99.5:<0.5 (S)
LeOPR1	30	KPi	8	2	98	n.d.	n.m.
NerA	30	KPi	8	4	84	12	n.m.
NerA (NAD^+)	30	KPi	8	11	82	7	n.m.
GluOx	30	KPi	8	44	56	n.d.	>99.5:<0.5 (S)
YqjM	30	KPi	8	1	96	3	n.m.
MR	30	KPi	8	n.d.	>99	n.d.	n.m.
MR (NAD^+)	30	KPi	8	1	98	1	n.m.
YqjM-v1	30	KPi	8	89	11	<1	97.1:2.9 (*R*)
YqjM-v1[(a)]	30	$HCOONH_4/NH_3$	8.8	>99	n.d.	<1	98.0:2.0 (*R*)
YqjM-v1[(b)]	30	$HCOONH_4/NH_3$	8.8	>99	n.d.	n.d.	97.6:2.4 (*R*)
YqjM-v1[(b)]	20	$HCOONH_4/NH_3$	8.8	>99	n.d.	n.d.	98.0:2.0 (*R*)
YqjM-v1[(b)]	10	$HCOONH_4/NH_3$	8.8	>99	n.d.	n.d.	98.2:1.8 (*R*)
YqjM-v1[(a)]	30	$HCOOCH_3NH_3/CH_3NH_2$	8.8	>99	n.d.	<1	97.7:2.3 (*R*)
YqjM-v1[(a)]	30	$HCOOCH_3NH_3/CH_3NH_2$	8.8	>99	n.d.	n.d.	97.9:2.1 (*R*)
YqjM-v1[(b)]	20	$HCOOCH_3NH_3/CH_3NH_2$	8.8	>99	n.d.	n.d.	98.1:1.9 (*R*)
YqjM-v1[(b)]	10	$HCOOCH_3NH_3/CH_3NH_2$	8.8	>99	n.d.	b.d.	98.3:1.7 (*R*)

[c] In the case of OYE2, the second enantiomer was never observed. The enantiomeric ratio was calculated based on the detection limit (2 area units) of the GC.

[d] Promiscuous disproportionation activity of some EReds with some substrates:

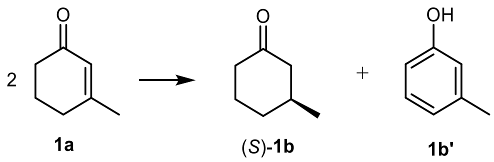

1a (*S*)-**1b** **1b'**

Chiral GC-FID measurements for the separation of the two enantiomers of **1b**

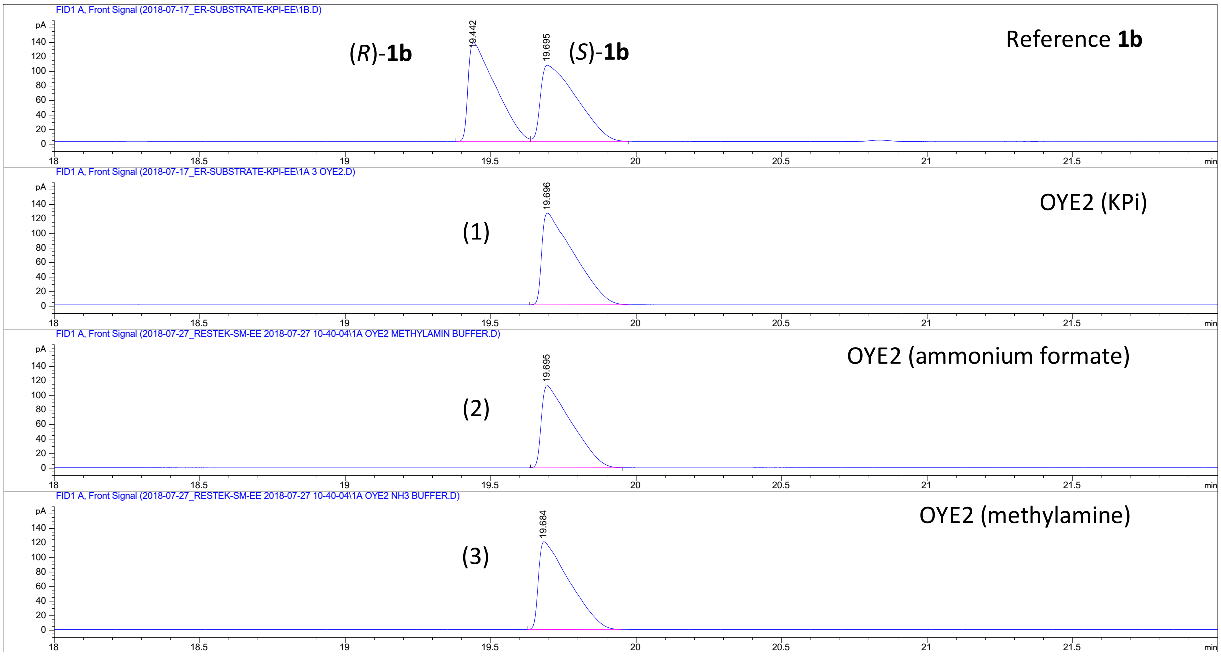

6.2. Substrate 1b - Amination

*6.2.1. Substrate **1b** - Amination using ammonium formate/ammonia buffer*

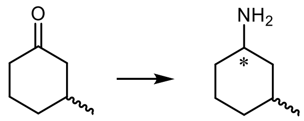

rac-**1b** **1c**

Reaction conditions: 1 mL total volume in 2 mL Eppendorf tubes. Buffer ammonium formate/ammonia (1 M, pH 8.8), $NADP^+$ (0.25 mM; note: for Ch1-AmDH and Rh-PhAmDH, NAD^+ was used), FDH-QRN (5 µM; note: for Ch1-AmDH and Rh-PhAmDH, wild-type Cb-FDH was used), aminating enzyme (20 µM), substrate (10 mM), T= 30 °C, time= 24 h.

Table S2. Amination (%) of *rac*-**1b** using IReds, AspRedAm and AmDHs with ammonium formate/ammonia buffer (GC measurement with achiral column).

enzyme	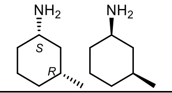	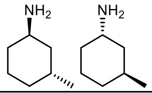	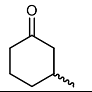
	(1*S*,3*R*)-**1c** + (1*R*,3*S*)-**1c**	(1*R*,3*R*)-**1c** + (1*S*,3*S*)-**1c**	*rac*-**1b**
Ch1-AmDH	6	20	74
Rh-PhAmDH	n.d.	n.d.	>99
Sp(S)-IRED	n.d.	n.d.	>99
IRED-1	26	4	70
IRED-5	28	28	44
IRED-13	20	2	78
IRED-14	18	2	80
IRED-15	27	1	72
IRED-20	95	4	1
IRED-21	n.d.	n.d.	>99
IRED-22	68	5	27
IRED-30	5	5	90
IRED-32	1	2	97
AspRedAm	11	1	88
IRED-7	n.d.	n.d.	>99
IRED-10	94	n.d.	6
IRED-11	11	n.d.	89
IRED-25	30	n.d.	70
LE-AmDH-v1	29	29	42

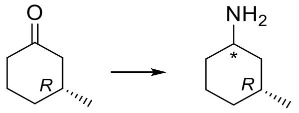

(*R*)-**1b** (1*R*,3*R*)-**1c** or (1*S*,3*R*)-**1c**

Reaction conditions: 1 mL total volume in 2 mL Eppendorf tubes. Buffer ammonium formate/ammonia (1 M, pH 8.8), $NADP^+$ (0.25 mM), FDH-QRN (5 µM), aminating enzyme (20 µM), substrate (10 mM), T= 30 °C, time= 24 h.

Table S3. Amination (%) of (*R*)-**1b** using IReds and AspRedAm with ammonium formate/ammonia buffer (GC measurement with achiral column).

enzyme	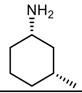	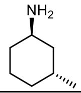	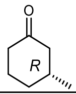
	(1*S*,3*R*)-**1c**	(1*R*,3*R*)-**1c**	(*R*)-**1b**
Sp(S)-IRED	n.d.	n.d.	>99
IRED-1	7	1	93
IRED-5	22	8	70
IRED-13	36	1	64
IRED-14	12	1	87
IRED-15	37	1	62
IRED-20	70	3	27
IRED-21	n.d.	n.d.	>99
IRED-22	52	11	37
IRED-30	1	<1	98
IRED-32	n.d.	n.d.	>99
AspRedAm	1	<1	99
IRED-7	2	n.d.	98
IRED-10	>99	n.d.	n.d.
IRED-11	13	<1	87
IRED-25	35	<1	65

*6.2.2. Substrate **1b** - Amination using methylammonium formate/methylamine buffer*

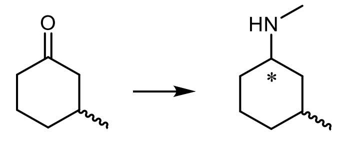

rac-**1b** **1d**

Reaction conditions: 1 mL total volume in 2 mL Eppendorf tubes. Buffer methylammonium formate/methylamine (1 M, pH 8.8), $NADP^+$ (0.25 mM), FDH-QRN (5 µM), aminating enzyme (20 µM), substrate (10 mM), T= 30 °C, time= 24 h.

Table S4. Amination (%) of *rac*-**1b** using IReds and AspRedAm with methylammonium formate/methylamine (GC measurement with achiral column).

enzyme	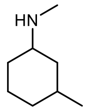	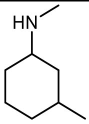	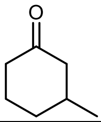	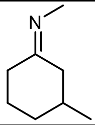
	(1*R*,3*R*)-**1d** + (1*S*,3*S*)-**1d**	(1*S*,3*R*)-**1d** + (1*R*,3*S*)-**1d**	*rac*-**1b**	imine
Sp(S)-IRED	5	12	70	13
IRED-1	2	2	80	16
IRED-5	36	64	n.d.	n.d.
IRED-13	19	80	1	n.d.
IRED-14	15	69	14	2
IRED-15	35	65	n.d.	n.d.
IRED-20	21	79	n.d.	n.d.
IRED-21	4	7	76	13
IRED-22	31	69	<1	n.d.
IRED-30	44	56	<1	n.d.
IRED-32	19	5	64	12
AspRedAm	5	64	26	5
IRED-7	2	4	94	n.d.
IRED-11	30	69	1	n.d.
IRED-25	2	98	n.d.	n.d.
IRED-30	5	9	86	n.d.
LE-AmDH-v1	7	6	87	n.d.

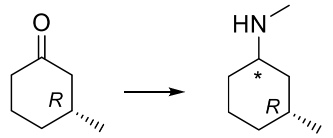

(*R*)-**1b** (1*R*,3*R*)-**1d** or (1*S*,3*R*)-**1d**

<u>Reaction conditions</u>: 1 mL total volume in 2 mL Eppendorf tubes. Buffer methylammonium formate/methylamine (1 M, pH 8.8), $NADP^+$ (0.25 mM), FDH-QRN (5 µM), aminating enzyme (20 µM), substrate (10 mM), T= 30 °C, time= 24 h.

Table S5. Amination (%) of (*R*)-**1b** using IReds and AspRedAm with methylamine/formic acid buffer (GC measurement with achiral column).

enzyme	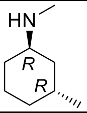	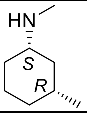	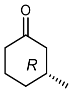	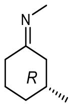
	(1*R*,3*R*)-**1d**	(1*S*,3*R*)-**1d**	**1b**	imine
Sp(S)-IRED	3	12	81	4
IRED-1	2	21	73	4
IRED-5	18	82	n.d.	n.d.
IRED-13	6	94	n.d.	n.d.
IRED-14	7	91	2	n.d.
IRED-15	5	95	n.d.	n.d.
IRED-20	31	69	n.d.	n.d.
IRED-21	4	1	90	5
IRED-22	46	54	n.d.	n.d.
IRED-30	41	59	n.d.	n.d.
IRED-32	6	2	86	6
AspRedAm	18	55	26	1
IRED-7	1	7	86	6
IRED-10	2	98	n.d.	n.d.
IRED-11	<1	>99	n.d.	n.d.
IRED-25	1	6	88	5

6.3. *Substrate 1a – Cascade reaction*

6.3.1. *Substrate **1a** – Cascade reaction using ammonium formate/ammonia buffer*

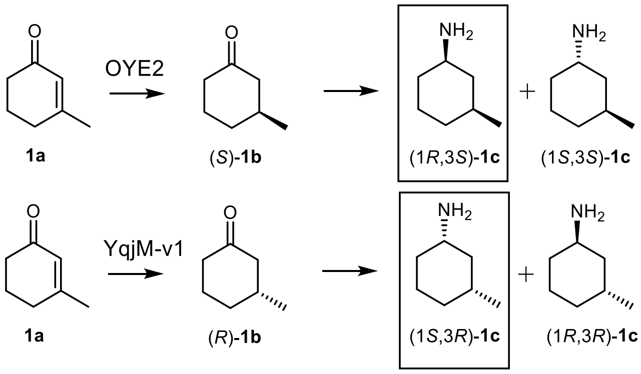

<u>Reaction conditions</u>: 1 mL total volume in 2 mL Eppendorf tubes. Buffer ammonium formate/ammonia (1 M, pH 8.8), $NADP^+$ (0.5 mM), FDH-QRN (10 µM), ERed (15 µM for OYE2 or 20 µM for YqjM-v1), aminating enzyme (40 µM), substrate **1a** (10 mM), T= 30 °C for OYE2 and 20 °C for YqjM-v1, time= 24 h.

Control reaction for alkene reduction (without addition of aminating enzyme): the enantiomeric ratio (*e.r.*) of the OYE2-catalyzed reaction was perfect (the second enantiomer was not observed at all; see chromatogram on next page). The *e.r.* of the YqjM-v1-catalyzed reaction was determined to be 98:2.

Chiral GC-FID chromatogram of the OYE2-catalyzed control reaction (i.e., from **1a** to (*S*)-**1b**).

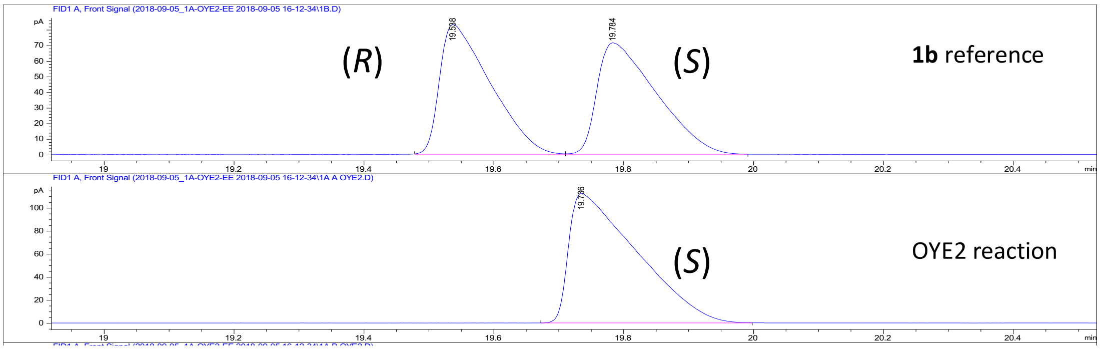

Table S6. Conversion (%) for the biocatalytic cascade of **1a** to **1c** combining OYE2 and IReds in one pot (GC measurement with achiral column).

substrate	ERED	IRED	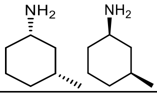	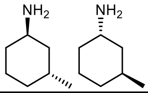	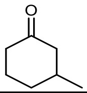	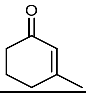
			(1*S*,3*R*)-**1c** + (1*R*,3*S*)-**1c**	(1*R*,3*R*)-**1c** + (1*S*,3*S*)-**1c**	**1b** [%]	**1a** [%]
**1a**	OYE2	-	-	-	>99	n.d.
**1a**	OYE2	IRED-1	n.d.	n.d.	>99	n.d.
**1a**	OYE2	IRED-5	27	71	2	n.d.
**1a**	OYE2	IRED-13	15	9	76	n.d.
**1a**	OYE2	IRED-15[a]	73	7	5	n.d.
**1a**	OYE2	IRED-20	94	6	n.d.	n.d.
**1a**	OYE2	IRED-22	98	2	n.d.	n.d.
**1a**	OYE2	IRED-22	97	3	n.d.	n.d.
**1a**	OYE2	IRED-22	97	3	n.d.	n.d.
**1a**	OYE2	IRED-10	96	4	n.d.	n.d.
**1a**	OYE2	IRED-25	5	<1	95	n.d.
**1a**	YqjM-v1				>99	n.d.
**1a**	YqjM-v1	IRED-13	95	2	3	n.d.
**1a**	YqjM-v1	IRED-15	96	3	1	n.d.
**1a**	YqjM-v1	IRED-10	99.8[b]	0.2	-	n.d.
**1a**	YqjM-v1	IRED-25	98	1	0.9	n.d.

[a] 15% of an unidentified side product was observed. [b] The percentage values were reported with one significant decimal digit if the value was 99.5% or higher.

Table S7. Diastereomeric and enantiomeric composition (%) for **1c** (GC measurement with a chiral column) after derivatization to the acetamide.

substrate	ERED	IRED	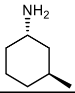	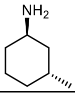	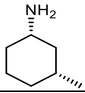	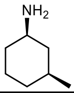
			(1*S*,3*S*)-**1c**	(1*R*,3*R*)-**1c**	(1*S*,3*R*)-**1c**	(1*R*,3*S*)-**1c**
**1a**	OYE2	IRED-5	71	n.d.	n.d.	29
**1a**	OYE2	IRED-13	32	n.d.	n.d.	69
**1a**	OYE2	IRED-15	6	n.d.	n.d.	94
**1a**	OYE2	IRED-20	4	n.d.	n.d.	96
**1a**	OYE2	IRED-22	0.7	n.d.	1.0	98.4
**1a**	OYE2	IRED-22	1.0	n.d.	0.6	98.4
**1a**	OYE2	IRED-22	0.8	n.d.	0.8	98.4
**1a**	OYE2	IRED-10	3.0	n.d.	0.2	96.8
**1a**	YqjM-v1	IRED-13	Tiny traces	1.1	95.4	3.2
**1a**	YqjM-v1	IRED-15	n.d.	1.7	95.4	3.0
**1a**	YqjM-v1	IRED-10	n.d.	n.d.	95.8	4.3
**1a**	YqjM-v1	IRED-25	n.d.	0.4	97.6	2.0

Separation of the **1c** isomers (IRED-10 and IRED-22 give the (1*S*,3*R*) enantiomer product starting with the (*R*)-configured ketone (*R*)-**1b**): [6]

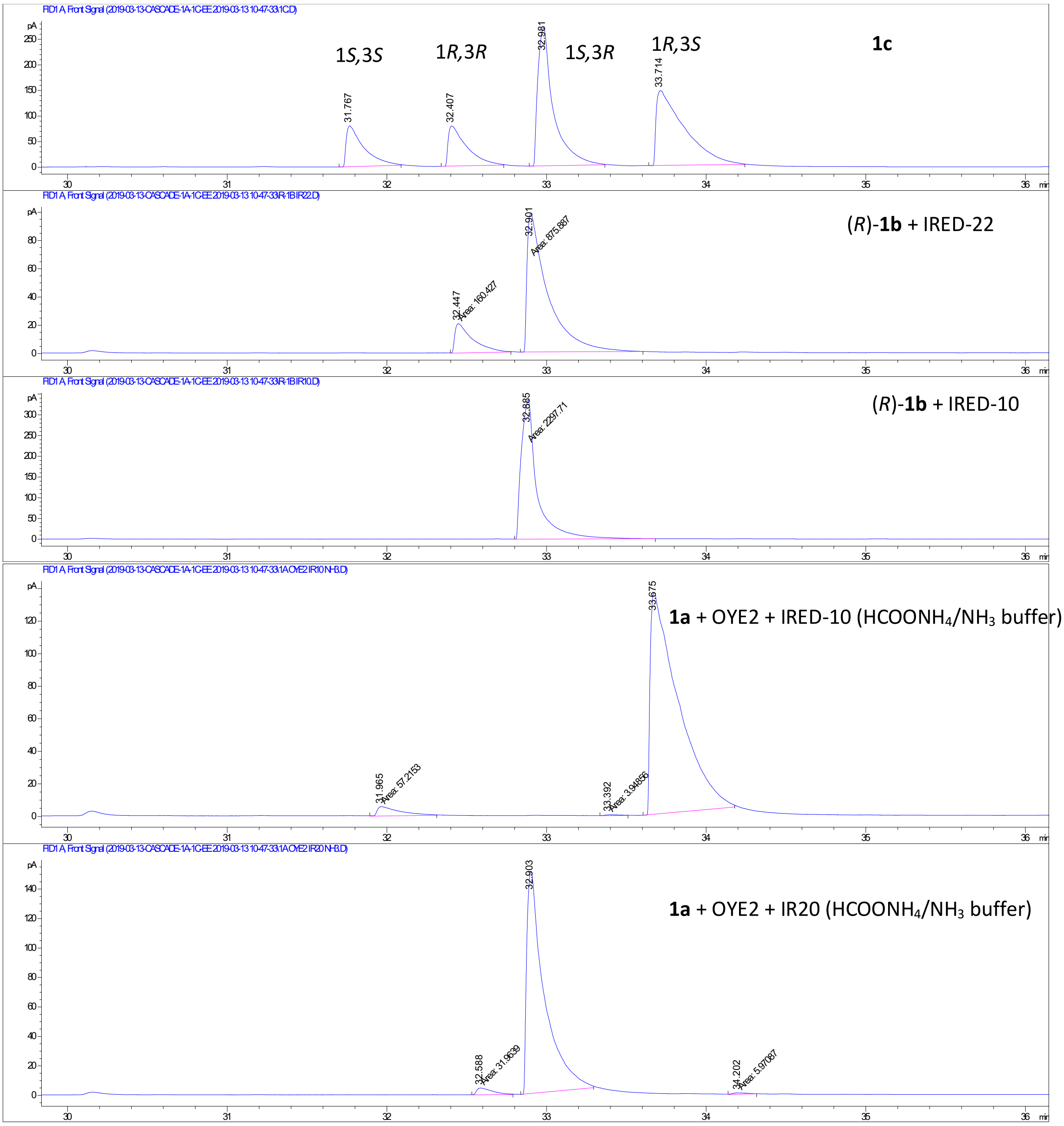

GC-FID chromatograms with a chiral column of **1a**+OYE2+IRED-22 (ammonium formate/ammonium buffer.

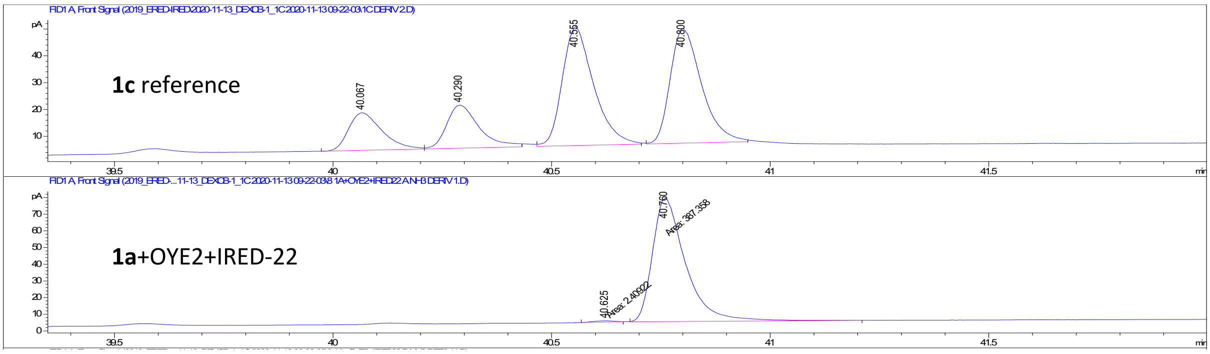

Chiral GC chromatograms of **1a**+YqjM+IREDs (ammonium formate/ammonium buffer.

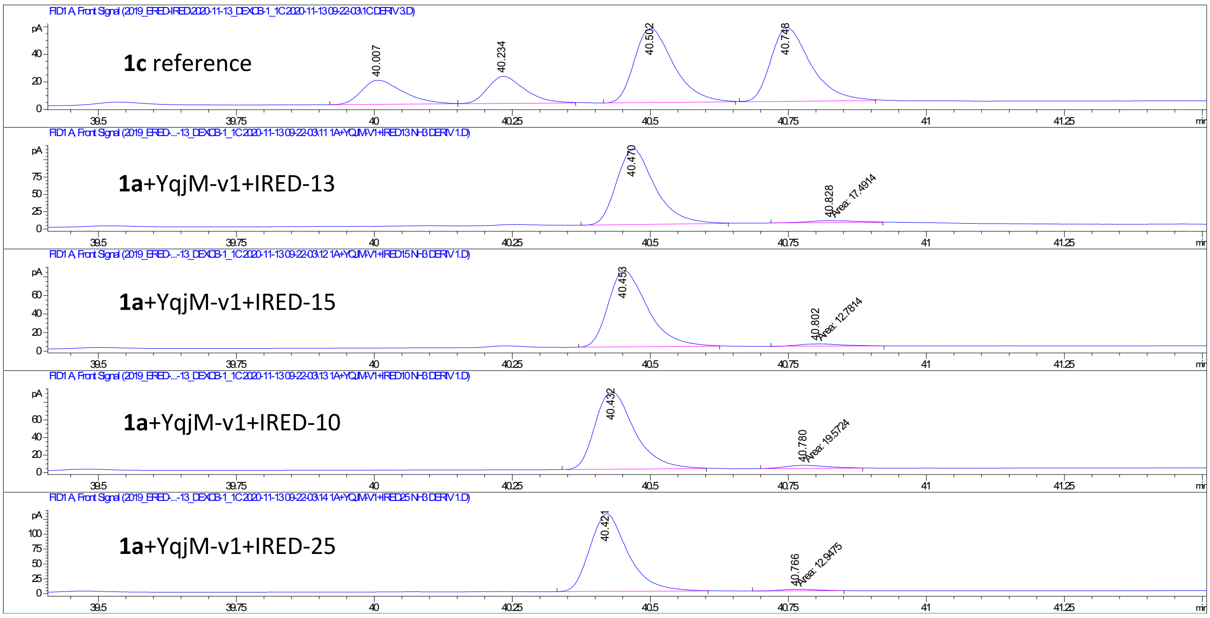

*6.3.1. Substrate **1a** – Cascade reaction using methylammonium formate/methylamine buffer*

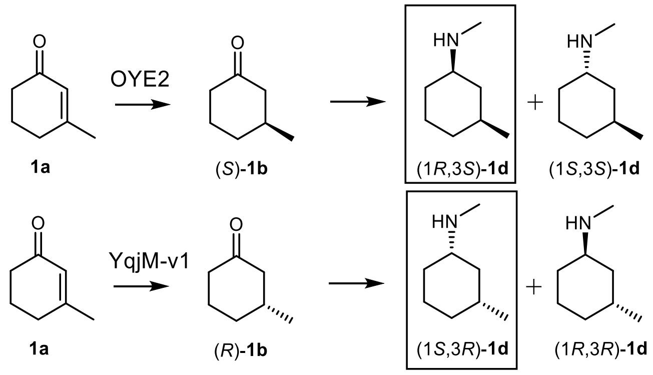

Reaction conditions: 1 mL total volume in 2 mL Eppendorf tubes. Buffer ammonium formate/ammonia (1 M, pH 8.8), NADP+ (0.5 mM), FDH-QRN (10 µM), ERed (15 µM for OYE2 or 20 µM for YqjM-v1), aminating enzyme (40 µM), substrate **1a** (10 mM), T= 30 °C for OYE2 and 20 °C for YqjM-v1, time= 24 h.

Control reaction for alkene reduction (without addition of aminating enzyme): the enantiomeric ratio (*e.r.*) of the OYE2-catalyzed reaction was perfect (the second enantiomer was not observed at all). The *e.r.* of the YqjM-v1-catalyzed reaction was determined to be 98%.

Table S8. Conversion (%) for the biocatalytic cascade of **1a** to **1d** combining OYE2 and aminating enzymes in one pot (GC measurement with an achiral column).

substrate	ERED	aminating enzyme	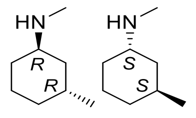	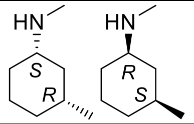	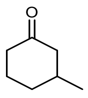	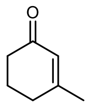
			(1*R*,3*R*)-**1d** + (1*S*,3*S*)-**1d**	(1*S*,3*R*)-**1d** + (1*R*,3*S*)-**1d**	**1b**	**1a**
**1a**	OYE2	-	-	-	92[a]	n.d.
**1a**	OYE2	IRED-5	50	50	n.d.	n.d.
**1a**	OYE2	IRED-13	32	65	3	n.d.
**1a**	OYE2	IRED-14	30	63	7	n.d.
**1a**	OYE2	IRED-15	63	37	n.d.	n.d.
**1a**	OYE2	IRED-20	10	90	n.d.	n.d.
**1a**	OYE2	IRED-22	15	85	n.d.	n.d.
**1a**	OYE2	IRED-30	46	54	n.d.	n.d.
**1a**	OYE2	AspRedAm	2	98	0.4	n.d.
**1a**	OYE2	IRED-11	5	92	n.d.[a]	n.d.
**1a**	YqjM-v1				91[a]	n.d.
**1a**	YqjM-v1	IRED-10	4	96	n.d.	n.d.
**1a**	YqjM-v1	IRED-11	0.5	98	0.2	n.d.

[a] imine peak of adduct between **1b** and ammonia: 8% for OYE2, 3% for OYE2+IRED11, 9% for YqjM-v1

Note: The peak annotation of the four amine peaks in the chiral GC measurement was done after upscaling of the enzymatic reactions using ammonium formate/ammonia buffer and subsequent chemical methylation as described in section 12.1.4.

Table S9. Diastereomeric and enantiomeric composition (%) for **1d** (chiral GC Measurement) after derivatization to the acetamide.[a]

substrate	ERED	aminating enzyme	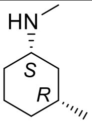	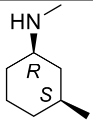	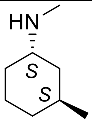	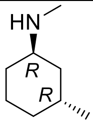
			(1*S*,3*R*)-**1d**	(1*R*,3*S*)-**1d**	(1*S*,3*S*)-**1d**	(1*R*,3*R*)-**1d**
**1a**	OYE2	IRED-5	n.d.	51	49	<1
**1a**	OYE2	IRED-13	n.d.	67	32	<1
**1a**	OYE2	IRED-14	n.d.	69	31	n.d.
**1a**	OYE2	IRED-15	n.d.	39	61	n.d.
**1a**	OYE2	IRED-20	n.d.	90	10	<1
**1a**	OYE2	IRED-22	n.d.	85	14	<1
**1a**	OYE2	IRED-30	n.d.	55	45	<1
**1a**	OYE2	AspRedAm	n.d.	98.6	1.4	n.d.
**1a**	OYE2	IRED-11	n.d.	95.2	4.8	n.d.
**1a**	YqjM-v1	IRED-10	95.6	1.2	1.4	1.8
**1a**	YqjM-v1	IRED-11	97.6	1.9	0.1	0.3

[a] Compositions values were reported with one significant decimal digit in selected cases where high selectivity was found.

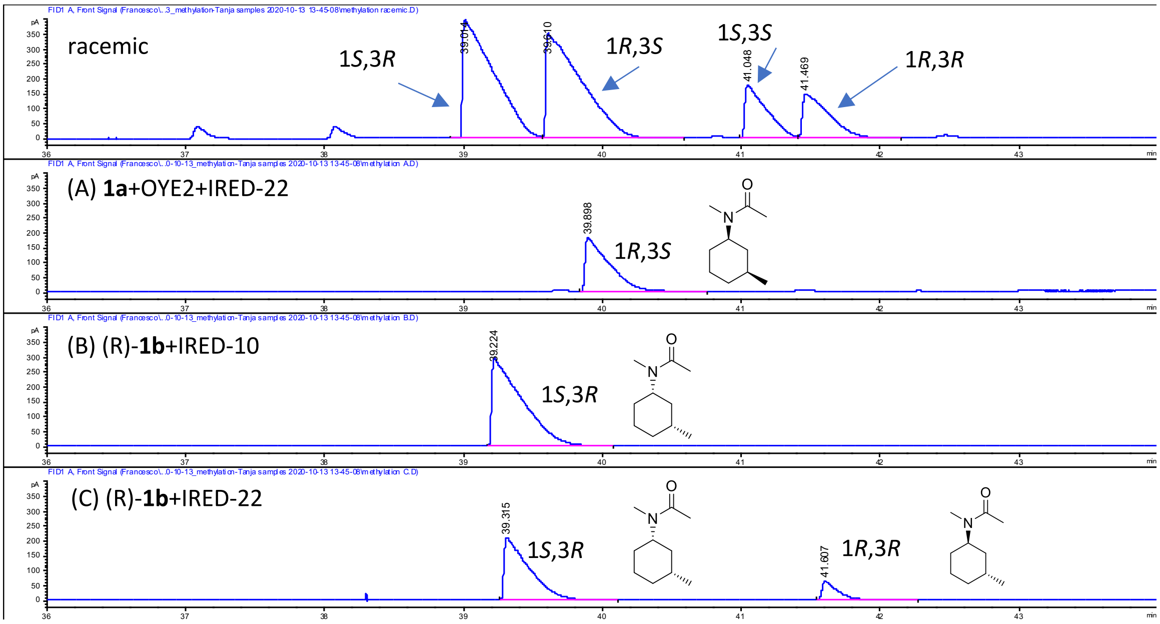

GC chromatograms Dex-CB-2 method amid

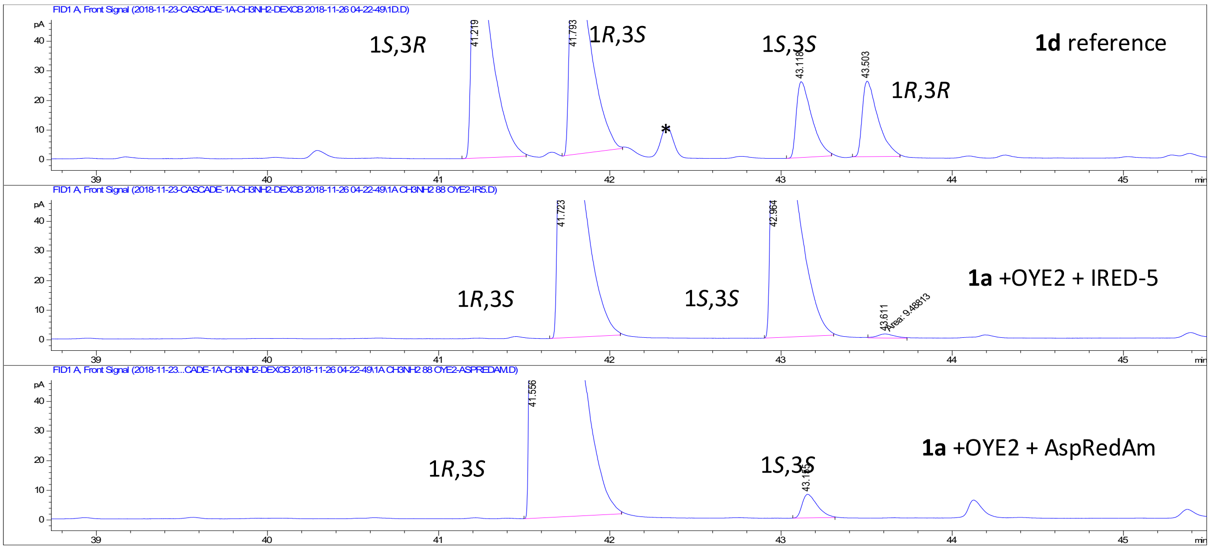

GC-FID chromatograms with a chiral column of **1a**+OYEs+aminating enzymes (methylammonium formate/methylamine buffer.

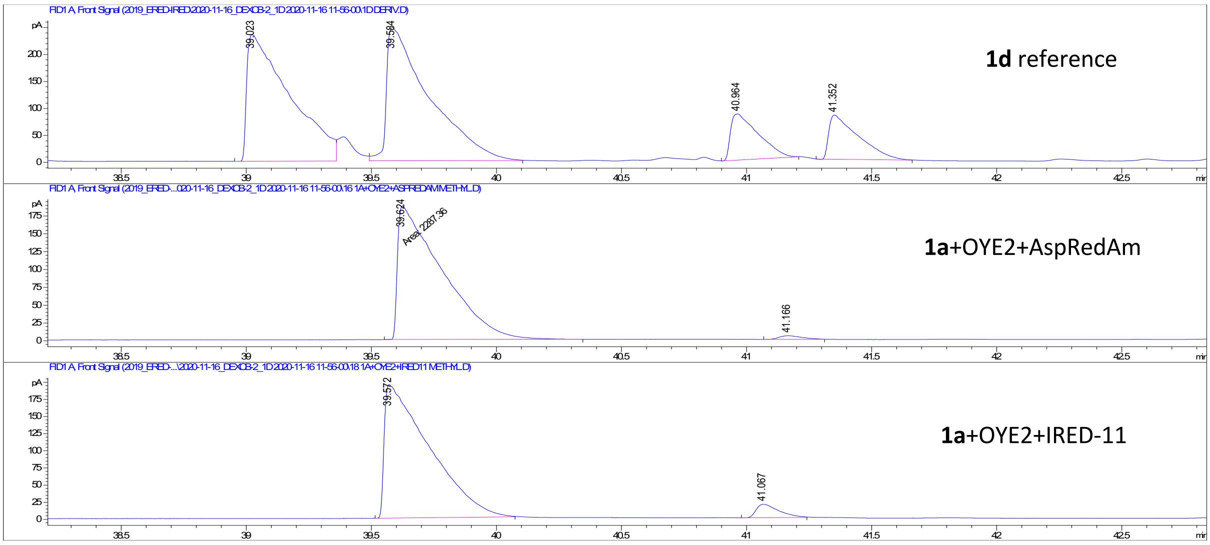

Chiral GC-FID chromatograms of 1a+YqjM-v1+aminating enzymes (methylamine/formic acid buffer.

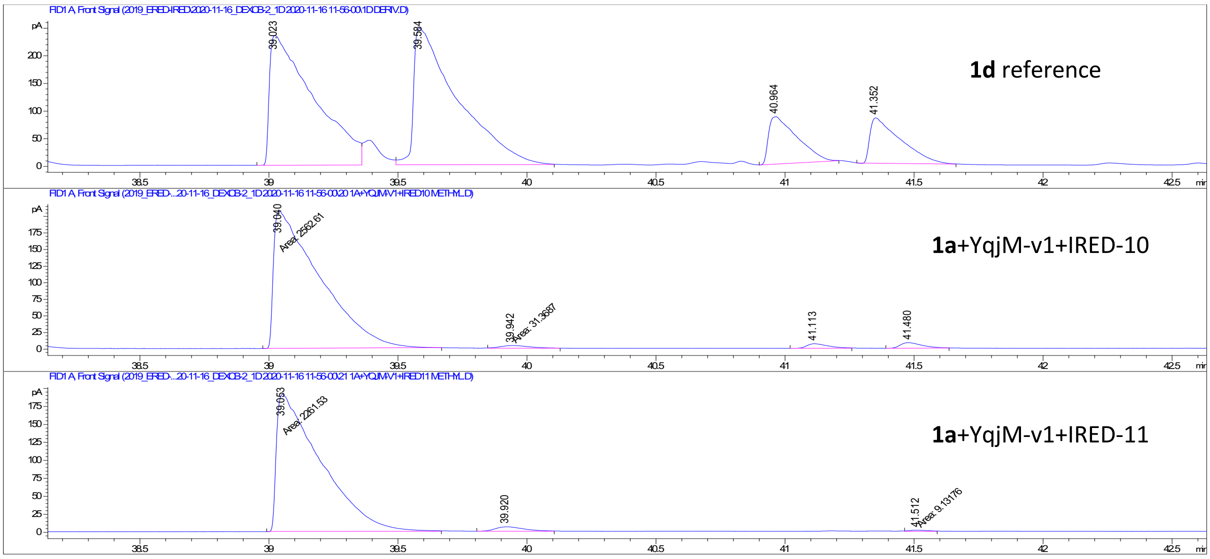

7. Biocatalytic reactions performed for substrate 2a and 2b

7.1. *Substrate 2a - Stereoselective reduction of the double bond (ERED-Reaction)*

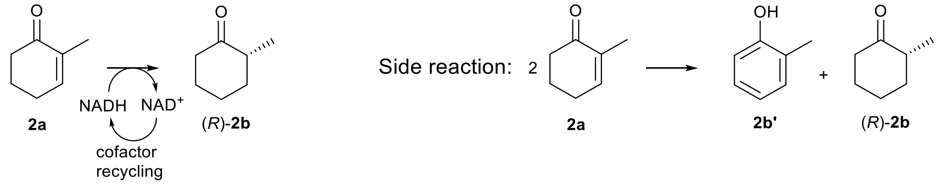

Reaction conditions: 1 mL total volume in 2 mL Eppendorf tubes. Buffer KPi (50 mM, pH 8.0) or ammonium formate/ammonia buffer (1M, pH 8.8) or methylammonium formate/methylamine buffer (1M, pH 8.8), $NADP^+$ (0.25 mM), sodium formate (30 mM, only for KPi buffer), FDH-QRN (5 µM), ERed (5–20 µM), substrate **2a** (10 mM), T=10–30 °C, time= 20 h. NerA and MR were also tested with NAD^+ (0.25 mM) in combination with Cb-FDH (5 µM).

According to the publications by Hall *et al* [27, 28], OYE2 and OYE3 give 87% and 85% (*R*)-**2b**, and LeOPR1 and YqjM give 75% and 84% (*R*)-**2b**, respectively, whereby the stereoselectivity of YqjM could get increased to 93% by cofactor change.

Table S10. Conversion and *e.r.* for **2a** using EReds.

enzyme	ERed (µM)	T (°C)	buffer	pH	**2b**	**2a**	phenol deriv. (**2b'**)	*e.r.* (*R*)-**2b**
Blank	-	30	KPi 50 mM	8	n.d.	>99	n.d.	n.d.
PETNR	5	30	KPi 50 mM	8	94	6	n.d.	93:7
TOYE	5	30	KPi 50 mM	8	87	10	3	95:5
TOYE	10	20	KPi 50 mM	8	88	9	3	97:3
TOYE	20	10	KPi 50 mM	8	88	8	4	98:2
OYE2	5	30	KPi 50 mM	8	96	2	2	94:6
OYE2	10	20	KPi 50 mM	8	97	1	2	96:4
OYE2	20	10	KPi 50 mM	8	98	n.d.	2	96:4
OYE3	5	30	KPi 50 mM	8	>99	<1	n.d.	94:6
OYE3	10	20	KPi 50 mM	8	>99	1	n.d.	96:4
OYE3	20	10	KPi 50 mM	8	>99	n.d.	n.d.	95:5
XenA	5	30	KPi 50 mM	8	96	n.d.	4	93:7
XenB	5	30	KPi 50 mM	8	>99	n.d.	<1	94:6
XenB	10	20	KPi 50 mM	8	>99	n.d.	n.d.	96:4
XenB	20	10	KPi 50 mM	8	99	1	n.d.	97:3
LeOPR1	5	30	KPi 50 mM	8	95	5	<1	93:7
NerA	5	30	KPi 50 mM	8	19	80	1	90:10
NerA NAD	5	30	KPi 50 mM	8	75	25	n.d.	92:8
GluOx	5	30	KPi 50 mM	8	>99	<1	n.d.	93:7
YqjM	10	20	KPi 50 mM	8	95	2	3	95:5
YqjM	5	30	KPi 50 mM	8	96	1	3	95:5
YqjM	20	40	KPi 50 mM	8	44	49	7	65:35
MR	5	30	KPi 50 mM	8	1	99	n.d.	n.d.
MR NAD	5	30	KPi 50 mM	8	69	31	n.d.	79:11
YqjM-v1	5	30	KPi	8	94	<1	6	55:45
YqjM-v1	10	30	$HCOONH_4/NH_3$	8.8	96	n.d.	4	52:48
YqjM-v1	10	30	$HCOOCH_3NH_3/CH_3NH_2$	8.8	96	n.d.	4	56:44

Comment: The conversions are all high using the EReds; however, the *e.r.* values were moderately high at 30 °C. The *e.r.* values could significantly be improved by reducing the temperature to 20 °C (*e.r.*: TOYE 97:3 *e.r.* instead of 95:5 *e.r.*; OYE2 96:4 *e.r.* instead of 94:6 *e.r.*; OYE3 96:4 *e.r.* instead of 94:6 *e.r.*). Reducing the temperature even further to 10 °C improved the *e.r.* even more. Specifically, for TOYE an *e.r.* of 98:2 could be reached. At lower temperatures, we also had to use a higher amount of enzyme. When we performed the biotransformation with YqjM at 30 °C, the highest *e.r.* of 95:5 was reached. Decreasing or increasing the temperature to 20 °C or 40 °C reduced the *e.r.* to 95:5 and 65:35, respectively.

Chiral GC-FID chromatogram for the separation of the **2b** enantiomers

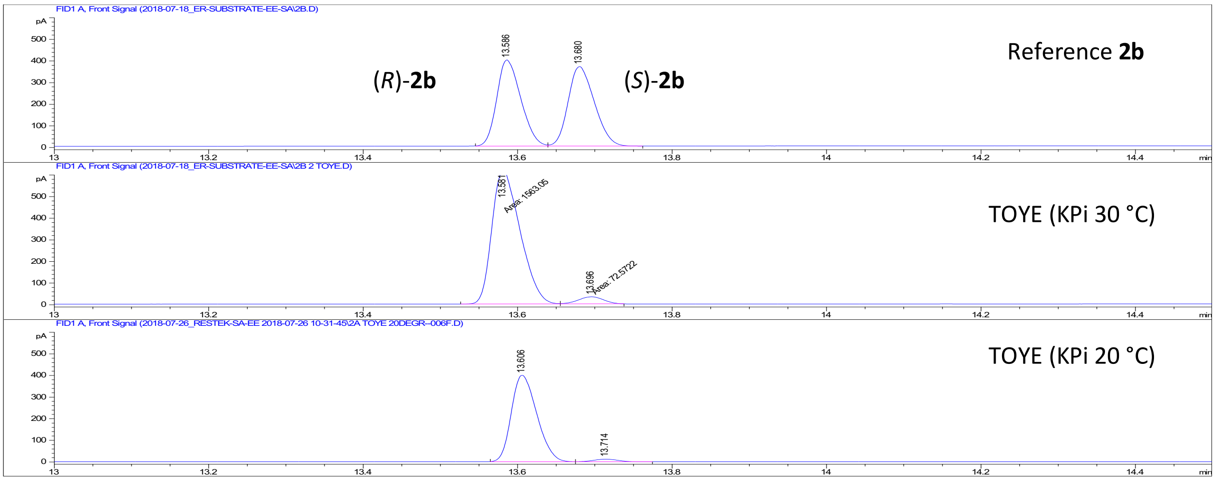

As TOYE and XenA are the most promising candidates for the stereoselective reduction of **2a**, they were tested with ammonium formate/ammonia and methylammonium formate/methylamine buffer as well. The results are shown in the Table S11 (The results for the KPi buffer are also displayed for comparison and are taken from the previous experiment):

<u>Reaction conditions</u>: 1 mL total volume in 2 mL Eppendorf tubes. Buffer ammonium formate/ammonia buffer (1M, pH 8.8 or pH 8) or methylamine/formic acid buffer (1M, pH 8.8 or 8), $NADP^+$ (0.25 mM), FDH-QRN (5 µM), ERED (20 µM), substrate **2a** (10 mM), T=10-30 °C, t=20 h.

Table S11. Conversion and *e.r.* for **2a** using TOYE and XenB at different buffers and temperatures.

enzyme	ERed (µM)	T (°C)	buffer	pH	**2b**	**2a**	phenol deriv.	*e.r.* (*R*)-**2b**
TOYE	5	30	KPi 50 mM	8	87	10	3	95:5
TOYE	10	20	KPi 50 mM	8	88	9	3	97:3
TOYE	20	10	KPi 50 mM	8	88	8	4	98:2
TOYE	20	30	$HCOONH_4/NH_3$	8.8	47	49	4	56:44
TOYE	20	20	$HCOONH_4/NH_3$	8.8	90	7	3	71:29
TOYE	20	10	$HCOONH_4/NH_3$	8.8	95	2	3	86:14
TOYE	20	30	$HCOONH_4/NH_3$	8	99	<1	1	79:21
TOYE	20	20	$HCOONH_4/NH_3$	8	99	n.d.	1	88:12
TOYE	20	10	$HCOONH_4/NH_3$	8	98	<1	2	94:6
TOYE	20	30	$HCOOCH_3NH_3/CH_3NH_2$	8.8	51	44	5	60:40
TOYE	20	20	$HCOOCH_3NH_3/CH_3NH_2$	8.8	71	25	4	77:23
TOYE	20	10	$HCOOCH_3NH_3/CH_3NH_2$	8.8	83	14	3	91:9
TOYE	20	30	$HCOOCH_3NH_3/CH_3NH_2$	8	99	<1	1	89:11
TOYE	20	20	$HCOOCH_3NH_3/CH_3NH_2$	8	>99	n.d.	<1	94:6
TOYE	20	10	$HCOOCH_3NH_3/CH_3NH_2$	8	>99	n.d.	<1	97:3
XenB	5	30	KPi 50 mM	8	>99	n.d.	<1	94:6
XenB	10	20	KPi 50 mM	8	>99	n.d.	n.d.	96:4
XenB	20	10	KPi 50 mM	8	99	1	n.d.	97:3
XenB	20	30	$HCOONH_4/NH_3$	8.8	70	30	n.d.	56:44
XenB	20	20	$HCOONH_4/NH_3$	8.8	97	3	n.d.	70:30
XenB	20	10	$HCOONH_4/NH_3$	8.8	>99	n.d.	n.d.	85:15
XenB	20	30	$HCOONH_4/NH_3$	8	69	31	n.d.	78:22
XenB	20	20	$HCOONH_4/NH_3$	8	>99	<1	n.d.	88:12
XenB	20	10	$HCOONH_4/NH_3$	8	>99	n.d.	n.d.	92:8
XenB	20	30	$HCOOCH_3NH_3/CH_3NH_2$	8.8	36	64	n.d.	55:45
XenB	20	20	$HCOOCH_3NH_3/CH_3NH_2$	8.8	1	98	1	n.d.
XenB	20	10	$HCOOCH_3NH_3/CH_3NH_2$	8.8	>99	n.d.	n.d.	94:6
XenB	20	30	$HCOOCH_3NH_3/CH_3NH_2$	8	61	39	n.d.	88:12
XenB	20	20	$HCOOCH_3NH_3/CH_3NH_2$	8	86	14	n.d.	95:5
XenB	20	10	$HCOOCH_3NH_3/CH_3NH_2$	8	>99	n.d.	n.d.	96:4

Comments: (1) For the reduction of **2a** with TOYE, the e.r. depended strongly on the buffer and pH that was used for the biotransformation. At 10 °C using KPi buffer an e.r. of 98:2 could be obtained, which decreased to 97:3 when using $HCOONH_3CH_3/CH_3NH_2$ buffer and even to 94:6 when using $HCOONH_4/NH_3$ buffer.

(2) For the reduction of **2a** with XenB, the results for XenB are similar to TOYE. The e.r. depended strongly on the buffer and pH that was used for the biotransformation. At 10 °C using KPi buffer an *e.r.* of 96:4 could be obtained, which remained statistically constant to 92:8 when using $HCOONH_3CH_3/CH_3NH_2$ buffer and decreased to 93:7 when using $HCOONH_4/NH_3$ buffer.

(3) During the reactions, we noticed that significant racemization of the (*R*)-**2b** substrate occurs. Therefore, the racemization was tested as below:

Racemization test for (*R*)-**2b** (produced in the reaction of **2a** catalyzed by TOYE):

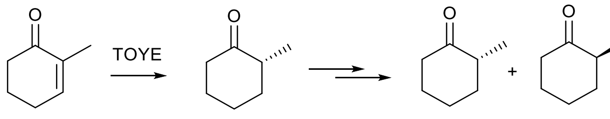

For this reaction, the pH of the buffer for the ERed reaction was decreased from 8 to 7 and the concentrations of $NADP^+$ and FDH-QRN were doubled.

Reaction conditions: (i) ERed-reaction: 1 mL total volume in 2 mL Eppendorf tubes. Buffer KPi (50 mM, pH 7.1), $NADP^+$ (0.5 mM), FDH-QRN (10 µM), sodium formate (30 mM), TOYE (40 µM), substrate **2a** (10 mM). The biocatalytic reaction was run at 10 °C for 23 h. (ii) In a second step, buffer (1 mL, 1 M, either ammonium formate/ammonia pH 6.8, 7.7 and 8.4 or methylammonium formate/methylamine pH 7.0, 8.0 or 8.4) was added and further incubated for 24 h at different temperatures.

Table S12. Conversion and *e.r.* for **2a** to **2b** using TOYE, and subsequent incubation of the produced intermediate at different temperatures in different buffer media varying in their pHs.

	pH	T (°C)	**2b**	*e.r.* (*R*)-**2b**
TOYE1 reaction			93	99:1
TOYE2 reaction			93	99:1
subsequent incubation in different buffer and temperatures				
KPi	7.1		94	94:6
$HCOONH_4/NH_3$	6.8	20 °C	>99	95:5
$HCOONH_4/NH_3$	7.7	20 °C	>99	95:5
$HCOONH_4/NH_3$	8.4	20 °C	>99	90:10
$HCOONH_4/NH_3$	6.8	25 °C	>99	94:6
$HCOONH_4/NH_3$	7.7	25 °C	>99	93:7
$HCOONH_4/NH_3$	8.4	25 °C	>99	86:14
$HCOONH_4/NH_3$	6.8	30 °C	>99	92:8
$HCOONH_4/NH_3$	7.7	30 °C	>99	90:10
$HCOONH_4/NH_3$	8.4	30 °C	>99	81:19
$HCOOCH_3NH_3/CH_3NH_2$	7.0	20 °C	>99	96:4
$HCOOCH_3NH_3/CH_3NH_2$	8.0	20 °C	>99	96:4
$HCOOCH_3NH_3/CH_3NH_2$	8.8	20 °C	>99	96:4
$HCOOCH_3NH_3/CH_3NH_2$	7.0	25 °C	>99	95:5
$HCOOCH_3NH_3/CH_3NH_2$	8.0	25 °C	>99	95:5
$HCOOCH_3NH_3/CH_3NH_2$	8.8	25 °C	>99	94:6
$HCOOCH_3NH_3/CH_3NH_2$	7.0	30 °C	>99	94:6
$HCOOCH_3NH_3/CH_3NH_2$	8.0	30 °C	>99	94:6
$HCOOCH_3NH_3/CH_3NH_2$	8.8	30 °C	>99	93:7

Comment: The racemization of **2b** depends on the temperature and on the pH value, but even more on the type of buffer during incubation. There is also a racemization of **2b** when incubated at 30 °C in KPi buffer. In general, racemization of substrate in methylammonium formate/methylamine buffer is slower than in the ammonium formate/ammonia buffer.

7.2. Substrate 2b - Amination

7.2.1. Substrate **2b** - Amination using ammonium formate/ammonia buffer

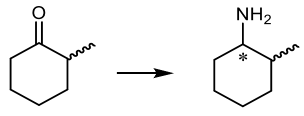

rac-**2b** **2c**

Reaction conditions: 1 mL total volume in 2 mL Eppendorf tubes. Buffer ammonium formate/ammonia (1 M, pH 8.8), $NADP^+$ (0.25 mM; note: for Ch1-AmDH and Rh-PhAmDH, NAD^+ was used), FDH-QRN (5 µM; note: for Ch1-AmDH and Rh-PhAmDH, wild-type Cb-FDH was used), aminating enzyme (20 µM), substrate (10 mM), T= 30 °C or 10 °C, time= 24 h.

Table S13. Amination (%) of *rac*-**2b** using IReds, AspRedAm and AmDHs with ammonium formate/ammonia buffer (GC measurement with achiral column).

enzyme	T (°C)	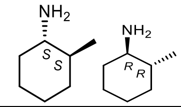	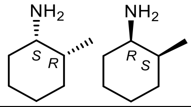	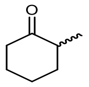
		(1*S*,2*S*)-**2c** + (1*R*,2*R*)-**2c**	(1*S*,2*R*)-**2c** + (1*R*,2*S*)-**2c**	*rac*-**2b**
Ch1-AmDH	30	n.d.	9	91
Rh-PhAmDH	30	n.d.	n.d.	>99
Sp(S)-IRED	30	3	4	93
IRED-1	30	n.d.	1	99
IRED-5	30	4	9	87
IRED-13	30	16	6	78
IRED-13	10	5	1	93
IRED-14	30	38	20	42
IRED-14	10	12	7	81
IRED-15	30	69	23	8
IRED-15	10	10	3	87
IRED-20	30	82	19	n.d.
IRED-20	10	42	8	50
IRED-21	30	1	1	98
IRED-22	30	68	32	n.d.
IRED-22	10	29	12	59
IRED-30	30	11	10	79
IRED-32	30	4	<1	96
AspRedAm	30	2	5	93
IRED-7	30	n.d.	n.d.	>99
IRED-10	30	16	25	59
IRED-10	10	13	21	66
IRED-11	30	n.d.	n.d.	>99
IRED-25	30	n.d.	n.d.	>99
LE-AmDH-v1	30	<1	5	95

7.2.2. *Substrate **2b** - Amination using methylammonium formate/methylamine buffer*

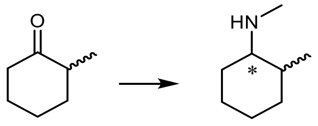

rac-**2b** **2d**

Reaction conditions: 1 mL total volume in 2 mL Eppendorf tubes. Buffer methylammonium formate/methylamine (1 M, pH 8.8), $NADP^+$ (0.25 mM), FDH-QRN (5 µM), aminating enzyme (20 µM), substrate (10 mM), T= 30 °C, time= 24 h.

Table S14. Amination (%) of *rac*-**2b** using IReds and AspRedAm with methylammonium formate/methylamine buffer (GC measurement with an achiral column).

enzyme	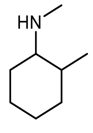	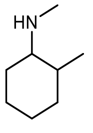	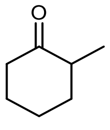	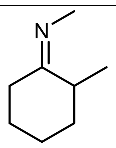
	cis-**2d**	*trans*-**2d**	*rac*-**2b**	
Sp(S)-IRED	1	4	91	4
IRED-1	n.d.	2	94	4
IRED-5	26	54	20	n.d.
IRED-13	60	21	9	n.d.
IRED-14	56	38	6	n.d.
IRED-15	19	79	2	n.d.
IRED-20	38	62	n.d.	n.d.
IRED-21	1	5	89	5
IRED-22	30	70	n.d.	n.d.
IRED-30	27	73	n.d.	n.d.
IRED-32	1	14	81	4
AspRedAm	13	45	40	2
IRED-7	n.d.	n.d.	>99	n.d.
IRED-10	5	35	60	n.d.
IRED-11	36	61	3	n.d.
IRED-25	n.d.	<1	>99	n.d.
LE-AmDH-v1	n.d.	n.d.	>99	n.d.

7.3. Substrate 2a – Cascade reaction

*7.3.1. Substrate **2a** – Cascade reaction using ammonium formate/ammonia buffer*

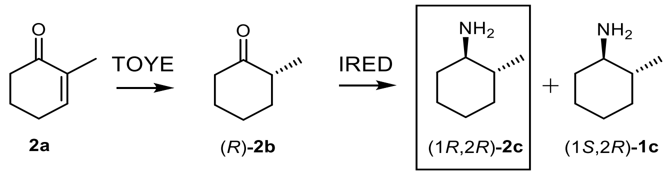

The reaction was performed at 10 °C and with a lower molarity of buffer to increase the *e.r.* of the ERed-catalyzed reaction through reduction of the racemization rate of **2b** (see paragraph 12.2.1, Reference of the four diastereomers produced with the ωTAs). We thought that combining the TOYE reaction with the IRed reaction would have led to a rapid amination of the in situ produced saturated intermediate **2b**, thus avoiding its racemization.

Reaction conditions: 1 mL total volume in 2 mL Eppendorf tubes. buffer ammonium formate/ammonia (200 mM, pH 8), $NADP^+$ (0.5 mM), FDH-QRN (10 µM), TOYE (20 µM), aminating enzyme (40 µM), substrate **2a** (10 mM), T= 10 °C, time= 20 h. All samples were put in the fridge for 30 min prior to the addition of substrate.

Table S15. Conversion (%) for the biocatalytic cascade of **2a** to **2c** combining TOYE and IReds in one pot (GC measurement with an achiral column).

substrate	ERed	IRed	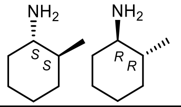	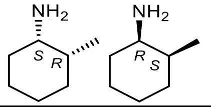	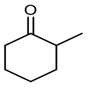	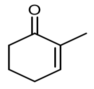
			(1*S*,2*S*)-**2c** + (1*R*,2*R*)-**2c**	(1*S*,2*R*)-**2c** + (1*R*,2*S*)-**2c**	**2b** [%]	**2a** [%]
**2a**	TOYE[(a)]				>99	n.d.
**2a**	TOYE	IRED-13	n.d.	n.d.	>99	n.d.
**2a**	TOYE	IRED-14	8	2	90	n.d.
**2a**	TOYE	IRED-15	4	2	94	n.d.
**2a**	TOYE	IRED-20	26	6	68	n.d.
**2a**	TOYE	IRED-22	25	2	73	n.d.
**2a**	TOYE	IRED-10	10	12	78	n.d.

[(a)] *e.r.*= 85:15

Table S16. Diastereomeric and enantiomeric composition (%) for **2c** (GC measurement with a chiral column) after derivatization to the acetamide, and *e.r.* of the remaining (unreacted) intermediate **2b**.

substrate	ERed	IRed	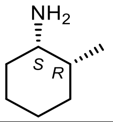	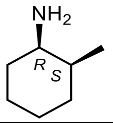	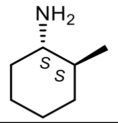	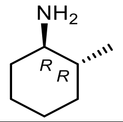	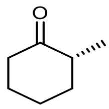
			(1*S*,2*R*)-**2c**	(1*R*,2*S*)-**2c**	(1*S*,2*S*)-**2c**	(1*R*,2*R*)-**2c**	*e.r.* (*R*)-**2b**
**2a**	TOYE	-					84:16
**2a**	TOYE	IRED-13					83:17
**2a**	TOYE	IRED-14	14.4	2.1	2.5	81.1	85:15
**2a**	TOYE	IRED-15	41.3	Tiny traces	3.1	55.6	83:17
**2a**	TOYE	IRED-20	18.0	0.5	2.3	79.2	83:17
**2a**	TOYE	IRED-22	6.8	1.1	0.7	91.4	82:18
**2a**	TOYE	IRED-10	52.3	1.6	0.6	46.5	83:17

In a second attempt, the cascade reaction was performed in two steps. Step 1: for the TOYE reaction, KPi buffer was used. The reaction was performed at 10 °C. This was supposed to give the highest possible *e.r.* value (see 7.1, comparison of different buffer and temperatures for the TOYE reaction). Step 2: addition of IRed and the necessary ammonium formate/ammonia buffer at 30 °C.

<u>Reaction conditions for the cascade reaction performed in two sequential steps</u>. (i) ERed-reaction in 1 mL reaction volume (in 2 mL Eppendorf tubes): buffer (KPi, 50 mM, pH 8.0), TOYE (30 µM), $NADP^+$ (0.25 mM), FDH-QRN (5 µM), sodium formate (30 mM), substrate (10 mM). The TOYE samples were put in the fridge for 30 min prior to the addition of substrate and then, the reactions were run for 24 h at 10 °C. (ii) Amination with IReds, total reaction volume is 2 mL. 1 mL of following reaction composition was added: buffer ammonium formate/ammonia (1 M, pH 8.8), IRed (50 µM), $NADP^+$ (0.25 mM), FDH-QRN (5 µM). The reactions were run for further 23 h at 30 °C. Note: the final concentration of the reactant in the second step is half.

Table S17. Conversion (%) for the biocatalytic cascade of **2a** to **2c** combining TOYE and IREDs in two sequential steps (GC measurement with an achiral column).

substrate	ERed	IRed	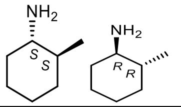	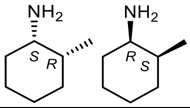	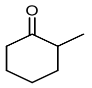	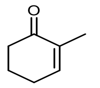
			(1*S*,2*S*)-**2c** + (1*R*,2*R*)-**2c**	(1*S*,2*R*)-**2c** + (1*R*,2*S*)-**2c**	**2b**	**2a**
**2a**	TOYE[(a)]				93	7
**2a**	TOYE	IRED-14	73	15	12	n.d.
**2a**	TOYE	IRED-15	59	41	n.d.	n.d.
**2a**	TOYE	IRED-20	77	23	n.d.	n.d.
**2a**	TOYE	IRED-22	86	14	n.d.	n.d.
**72a**	TOYE	IRED-10	45	55	n.d.	n.d.

[(a)] *e.r.*= 99:1

Table S18. Diastereomeric and enantiomeric composition (%) for **2c** (GC measurement with a chiral column) after derivatization to the acetamide.

substrate	ERed	IRed	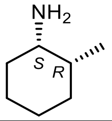	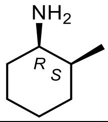	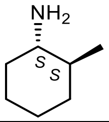	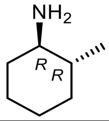
			(1*S*,2*R*)-**2c**	(1*R*,2*S*)-**2c**	(1*S*,2*S*)-**2c**	(1*R*,2*R*)-**2c**
**2a**	TOYE	IRED-14	12	6	7	75
**2a**	TOYE	IRED-15	40	1	7	52
**2a**	TOYE	IRED-20	22	2	5	71
**2a**	TOYE	IRED-22	10	4	3	83
**2a**	TOYE	IRED-10	48	6	2	44

Comment: The *e.r.* of the TOYE-catalyzed reaction was determined to be 99:1 for the (*R*)-configured enantiomer, but the conversion was 93%.

The test of racemization in solution for **2b** was described in section 7.1. This test showed that there is an influence of the temperature, the pH value, as well as type of buffer on the racemization rate of the saturated intermediate. Therefore, the pH of the two buffers used for the one-pot two-step cascade reaction was lowered to pH 7. (i) TOYE reaction was performed using KPi buffer at pH 7 at 10 °C, followed by the (ii) IRed reaction using ammonium formate/ammonia buffer at pH 7. As IReds do not work well at 10 °C, 20 °C reaction temperature was selected. As IRED-14 and IRED-22 were the most promising candidates, only these two IReds were used further.

<u>Reaction conditions for the cascade reaction performed in two sequential steps</u>: (i) TOYE-reaction 1 mL reaction volume (in 2 mL Eppendorf tubes): buffer (KPi, 50 mM, pH 7.0), TOYE (40 µM), $NADP^+$ (0.5 mM), FDH-QRN (10 µM), sodium formate (30 mM) and substrate (10 mM). The TOYE samples were put in the fridge for 30 min prior to the addition of substrate and then, the reactions were run for 24 h at 10 °C. (ii) Amination with IReds, total reaction volume is 2 mL. 1 mL of following reaction composition was added: buffer ammonium formate/ammonia (1 M, pH 7), IRed (50 µM), $NADP^+$ (0.5 mM), FDH-QRN (10 µM). The reactions were run for further 24 h at 20 °C. Note: the final concentration of the reactant in the second step is half.

Table S19. Conversion (%) for the biocatalytic cascade of **2a** to **2c** combining TOYE and IReds in two sequential steps at pH 7 (GC measurement with an achiral column).

substrate	ERed	IRed	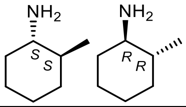	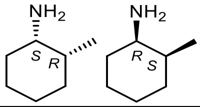	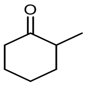	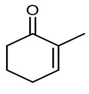
			(1*S*,2*S*)-**2c** + (1*R*,2*R*)-**2c**	(1*S*,2*R*)-**2c** + (1*R*,2*S*)-**2c**	**2b**	**2a**
**2a**	TOYE[(a)]	-	-	-	94	6
**2a**	TOYE	IRED-14	36	6	58	n.d.
**2a**	TOYE	IRED-22	89	10	1	n.d.

[(a)] *e.r.*=99:1

Table S20. Diastereomeric and enantiomeric composition (%) for **2c** (GC measurement with a chiral column) after derivatization to the acetamide.

substrate	ERed	IRed	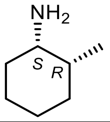	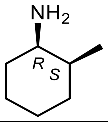	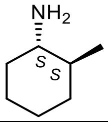	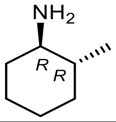
			(1*S*,2*R*)-**2c**	(1*R*,2*S*)-**2c**	(1*S*,2*S*)-**2c**	(1*R*,2*R*)-**2c**
**2a**	TOYE	IRED-14	11.1	3.1	4.0	81.8
**2a**	TOYE	IRED-22	8.3	2.3	1.8	87.7

7.3.2. Substrate **2a** – Cascade reaction using methylammonium formate/methylamine

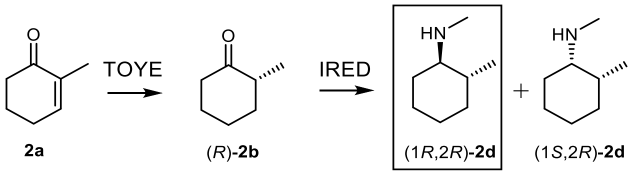

Like the second attempt for the reaction with ammonium formate/ammonia buffer, the cascade reaction using methylammonium formate/methylamine buffer was performed in two sequential steps:

Reaction conditions for the cascade reaction performed in two sequential steps: (i) ERED-reaction 1 mL reaction volume (in 2 mL Eppendorf tubes): buffer (KPi, 50 mM, pH 8.0), TOYE (30 µM), $NADP^+$ (0.25 mM), FDH-QRN (5 µM), sodium formate (30 mM) and substrate **2a** (10 mM). The TOYE samples were put in the fridge for 30 min prior to the addition of substrate and then, the reactions were run for 24 h at 10 °C. (ii) Amination with IReds, total reaction volume is 2 mL. 1 mL of following reaction composition was added: buffer methylammonium formate/methylamine (1 M, pH 8.8), IRed (50 µM), $NADP^+$ (0.25 mM), FDH-QRN (5 µM). The reactions were run for further 23 h at 30 °C. Note: the final concentration of the reactant in the second step is half.

Table S21. Conversion (%) for the biocatalytic cascade of **2a** to **2d** combining TOYE and aminating enzymes in two sequential steps (GC measurement with an achiral column).

substrate	ERed	aminating enzyme	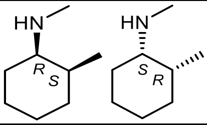	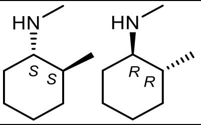	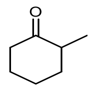	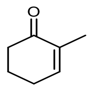
			(1*R*,2*S*)-**2d** + (1*S*,2*R*)-**2d**	(1*R*,2*R*)-**2d** + (1*S*,2*S*)-**2d**	**2b**	**2a**
**2a**	TOYE[(a)]	-			93	7
**2a**	TOYE	IRED-5	3	97	n.d.	n.d.
**2a**	TOYE	IRED-13	11	89	n.d.	n.d.
**2a**	TOYE	IRED-14	19	80	1	n.d.
**2a**	TOYE	IRED-15	53	38	9	n.d.
**2a**	TOYE	IRED-20	5	95	<1	n.d.
**2a**	TOYE	IRED-22	21	79	n.d.	n.d.
**2a**	TOYE	IRED-30	5	95	n.d.	n.d.
**2a**	TOYE	AspRedAm	2	93	5	n.d.
**2a**	TOYE	IRED-10	14	85	1	n.d.
**2a**	TOYE	IRED-11	4	96	n.d.	n.d.

[(a)] *e.r.*=99:1

Table S22. Diastereomeric and enantiomeric composition (%) for **2d** (GC measurement with a chiral column) after derivatization to the acetamide.

substrate	ERed	IRed	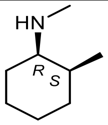 (1*R*,2S)-**2d**	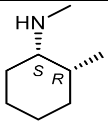 (1*S*,2*R*)-**2d**	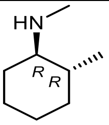 (1*R*,2*R*)-**2d**	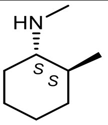 (1*S*,2S)-**2d**
**2a**	TOYE	IRED-5	2	<1	96	1
**2a**	TOYE	IRED-13	4	8	88	-
**2a**	TOYE	IRED-14	4	16	79	1
**2a**	TOYE	IRED-15	n.d.	59	35	6
**2a**	TOYE	IRED-20	3	2	94	<1
**2a**	TOYE	IRED-22	2	20	77	2
**2a**	TOYE	IRED-30	2	3	94	1
**2a**	TOYE	AspRedAm	2	<1	94	4
**2a**	TOYE	IRED-10	<1	14	81	4
**2a**	TOYE	IRED-11	3	1	95	1

After the racemization test performed for **2b** (paragraph 7.1), new parameter for the cascade reaction were set wherein the pH of the buffers used for the one-pot two-step cascade reaction was lowered to pH 7. (i) TOYE reaction using KPi buffer at pH 7 at 10 °C, followed by the (ii) IRed reaction using ammonium formate/ammonia buffer at pH 7 and 20 °C.

<u>Reaction conditions for the cascade reaction performed in two sequential-steps</u>: (i) TOYE-reaction in 1 mL reaction volume (in 2 mL Eppendorf tubes): buffer (KPi, 50 mM, pH 7.0), TOYE (40 µM), $NADP^+$ (0.5 mM), FDH-QRN (10 µM), sodium formate (30 mM). substrate (10 mM). The TOYE samples were put in the fridge for 30 min prior to the addition of substrate and then, the reactions were run for 24 h at 10 °C. (ii) Amination with IReds, total reaction volume is 2 mL. 1 mL of following reaction composition was added: buffer methylammonium formate/methylamine (1 M, pH 7), IRed (50 µM), $NADP^+$ (0.5 mM), FDH-QRN (10 µM). The reactions were run for further 24 h at 20 °C. Note: the final concentration of the reactant in the second step is half.

Table S23. Conversion (%) for the biocatalytic cascade of **2a** to **2d** combining TOYE and aminating enzymes in two sequential steps at pH 7 (GC measurement with an achiral column).

substrate	ERed	aminating enzyme	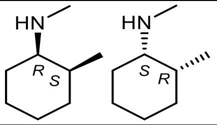	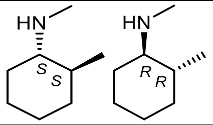	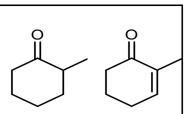	
			(1*R*,2*S*)-**2d** + (1*S*,2*R*)-**2d**	(1*R*,2*R*)-**2d** + (1*S*,2*S*)-**2d**	**2b**	**2a**
**2a**	TOYE[(a)]	-	-	-	94	6
**2a**	TOYE	IRED-5	3	93	4	n.d.
**2a**	TOYE	IRED-20	6	93	1	n.d.
**2a**	TOYE	IRED-30	6	93	1	n.d.
**2a**	TOYE	AspRedAm	3	66	31	n.d.
**2a**	TOYE	IRED-11	4	95	1	n.d.

[(a)] *e.r.*=99:1

Table S24. Diastereomeric and enantiomeric composition [%] for **2d** (GC measurement with a chiral column) after derivatization to the acetamide.

substrate	ERed	aminating enzyme	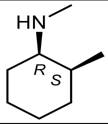	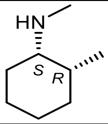	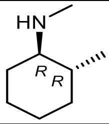	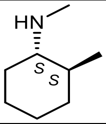
			(1*R*,2*S*)-**2d**	(1*S*,2*R*)-**2d**	(1*R*,2*R*)-**2d**	(1*S*,2*S*)-**2d**
**2a**	TOYE	IRED-5	2.5	0.6	96.1	0.8
**2a**	TOYE	IRED-20	3.0	3.6	92.9	0.5
**2a**	TOYE	IRED-30	2.5	4.0	92.7	0.8
**2a**	TOYE	AspRedAm	3.0	0.8	93.7	2.5
**2a**	TOYE	IRED-11	2.9	1.0	95.9	0.2

8. Biocatalytic reactions performed for substrate 3a and 3b

8.1. Substrate 3a - Stereoselective reduction of the double bond (ERed-reaction)

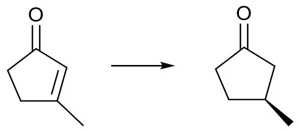

Reaction conditions: 1 mL total volume in 2 mL Eppendorf tubes. Buffer KPi (50 mM, pH 8.0) or ammonium formate/ammonia buffer (1 M) or methylammonium formate/methylamine buffer (1 M), $NADP^+$ (0.25 mM), sodium formate (30 mM, only for KPi buffer), FDH-QRN (5 µM), ERed (5-20 µM), substrate **1a** (10 mM), T= 10–30 °C, time= 20 h. NerA and MR were also tested with NAD^+ (0.25 mM) in combination with Cb-FDH (5 µM).

According to the publication by Hall *et al* [27], OYE2 and OYE3 give *e.r.* >99.5:<0.5 (*S*)-**3b.**

Table S25. Conversion and *e.r.* for **3a** using EReds under different reaction conditions.

enzyme	ERed (µM)	T (°C)	buffer	pH	**3b**	**3a**	*e.r.* **3b**[a]
Blank	-	30	KPi 50 mM	8	n.d.	>99	n.m.
PETNR	5	30	KPi 50 mM	8	21	79	99.7:0.3 (*S*)
PETNR	20	30	$HCOONH_4/NH_3$	8.8	67	33	99.8:0.2 (*S*)
PETNR	20	30	$HCOONH_4/NH_3$	8.8	59	41	>99.8:<0.2
PETNR	20	20	$HCOONH_4/NH_3$	8.8	22	78	>99.8:<0.2
PETNR	20	30	$HCOONH_4/NH_3$	8	67	33	>99.8:<0.2
PETNR	20	20	$HCOONH_4/NH_3$	8	30	69	>99.8:<0.2
PETNR	20	30	$HCOOCH_3NH_3/CH_3NH_2$	9	61	39	99.8:0.2 (*S*)
PETNR	20	30	$HCOOCH_3NH_3/CH_3NH_2$	8.8	61	39	99.8:0.2
PETNR	20	30	$HCOOCH_3NH_3/CH_3NH_2$	8.8	37	63	99.8:0.2
PETNR	20	30	$HCOOCH_3NH_3/CH_3NH_2$	8	21	79	99.8:0.2
PETNR	20	20	$HCOOCH_3NH_3/CH_3NH_2$	8	18	82	99.8:0.2
TOYE	5	30	KPi 50 mM	8	<1	>99	n.m
OYE2	5	30	KPi 50 mM	8	40	60	99.6:0.4 (*S*)
OYE2	20	30	$HCOONH_4/NH_3$	8.8	88	12	99.6:0.4 (*S*)
OYE2	20	20	$HCOONH_4/NH_3$	8.8	78	22	>99.8:<0.2
OYE2	20	30	$HCOONH_4/NH_3$	8	76	24	99.6:0.4
OYE2	20	20	$HCOONH_4/NH_3$	8	71	29	>99.8:<0.2
OYE2	20	30	$HCOOCH_3NH_3/CH_3NH_2$	8.8	12	82	96:4 (*S*)
OYE2	20	20	$HCOOCH_3NH_3/CH_3NH_2$	8.8	4	97	96:4
OYE2	20	30	$HCOOCH_3NH_3/CH_3NH_2$	8	76	24	99.6:0.4
OYE2	20	20	$HCOOCH_3NH_3/CH_3NH_2$	8	68	32	99.8:0.2
OYE3	5	30	KPi 50 mM	8	6	94	n.m
XenA	5	30	KPi 50 mM	8	<1	>99	n.m
XenB	5	30	KPi 50 mM	8	13	87	99:1
LeOPR1	5	30	KPi 50 mM	8	1	99	n.m
NerA	5	30	KPi 50 mM	8	1	99	n.m
NerA NAD	5	30	KPi 50 mM	8	4	96	n.m
GluOx	5	30	KPi 50 mM	8	23	77	98:2
YqjM	5	30	KPi 50 mM	8	1	99	n.m
MR	5	30	KPi 50 mM	8	n.d.	>99	n.m
MR NAD	5	30	KPi 50 mM	8	n.d.	>99	n.m
YqjM-v1	5	30	KPi 50 mM	8	6	94	73:27 (*R*)
YqjM-v1	10	30	$HCOONH_4/NH_3$	8.8	10	90	76:24 (*R*)
YqjM-v1	10	30	$HCOOCH_3NH_3/CH_3NH_2$	8.8	10	90	75:25 (*R*)

[a] The enantiomeric ratio values were reported with one significant decimal digit if the value was 99.5:0.5 or higher. The enantiomeric ratio was calculated based on the detection limit (2 area units) of the GC.

Chiral GC-FID chromatograms for the separation of the **3b** enantiomers

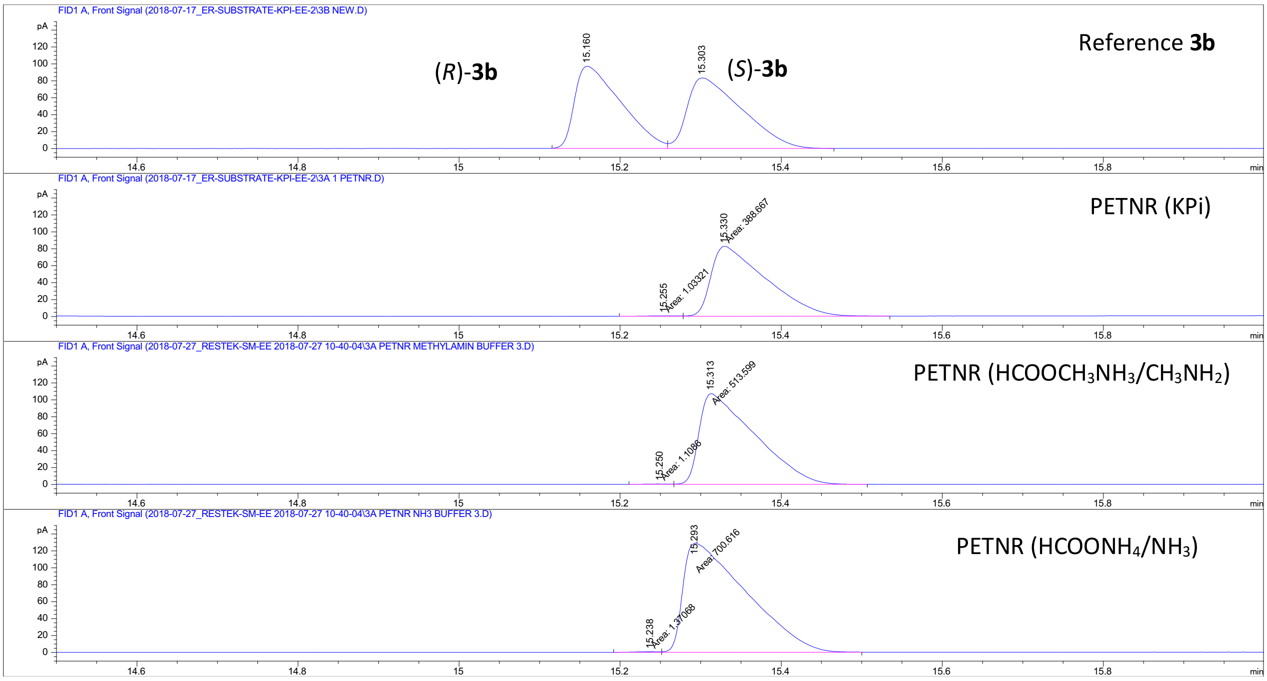

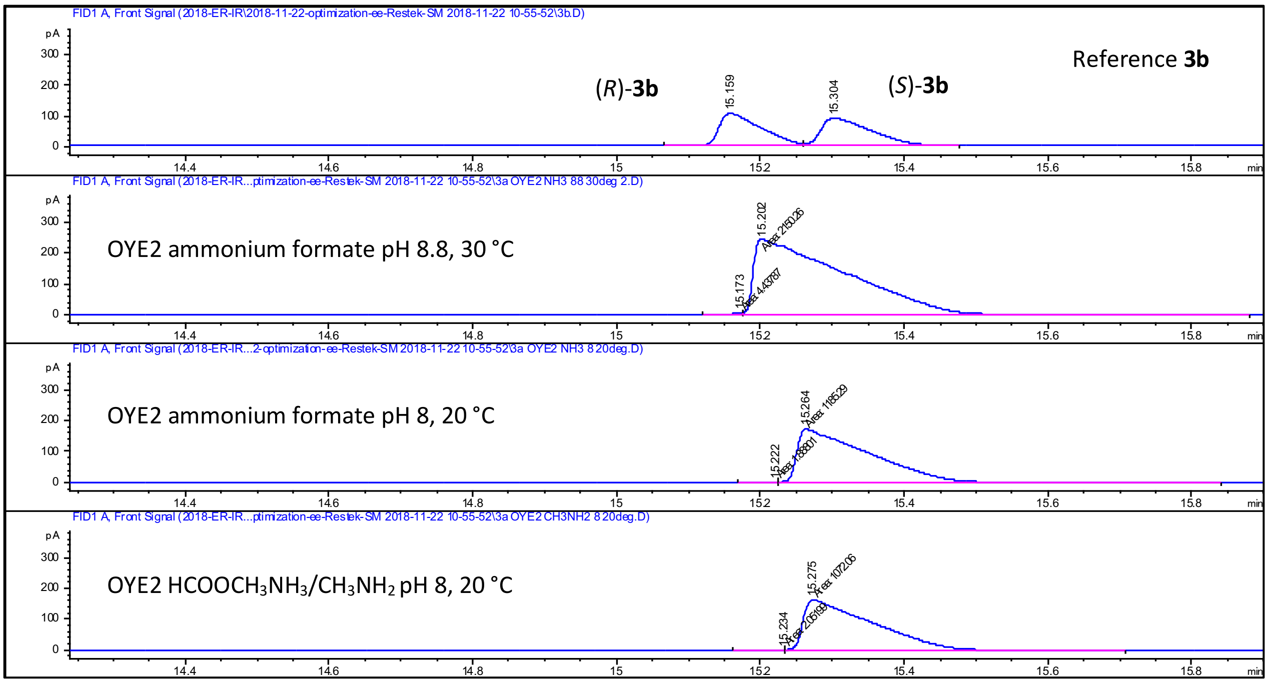

8.2. Substrate 3b - Amination

*8.2.1. Substrate **3b** - Amination using ammonium formate/ammonia buffer*

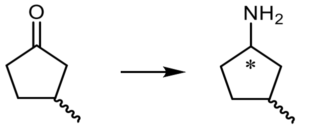

rac-**3b** **3c**

Reaction conditions: 1 mL total volume in 2 mL Eppendorf tubes. Buffer ammonium formate/ammonia (1 M, pH 8.8), $NADP^+$ (0.25 mM; in the case of Ch1-AmDH and Rh-PhAmDH, NAD^+ was used), FDH-QRN (5 µM; in the case of Ch1-AmDH and Rh-PhAmDH, Cb-FDH was used), aminating enzyme (20 µM), substrate (10 mM), T= 30 °C, time= 24 h.

Table S26. Amination (%) of *rac*-**3b** using IReds, AspRedAm and AmDHs in ammonium formate/ammonia buffer (GC measurement with an achiral column).

Enzyme	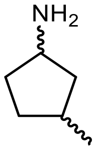	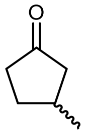
	**3c**	**3b**
Ch1-AmDH	n.d.	>99
Rh-PhAmDH	n.d.	>99
Sp(S)-IRED	n.d.	>99
IRED-1	n.d.	>99
IRED-5	n.d.	>99
IRED-13	n.d.	>99
IRED-14	n.d.	>99
IRED-15	n.d.	>99
IRED-20	38	62
IRED-21	n.d.	>99
IRED-22	25	75
IRED-30	n.d.	>99
IRED-32	n.d.	>99
AspRedAm	n.d.	>99
IRED-7*	n.d.	>99
IRED-10*	n.d.	>99
IRED-11*	n.d.	>99
IRED-25*	n.d.	>99
LE-AmDH-v1*	n.d.	>99

*8.2.2. Substrate **3b** - Amination using methylammonium formate/methylamine buffer*

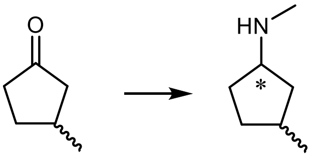

rac-**3b** **3d**

Reaction conditions: 1 mL total volume in 2 mL Eppendorf tubes. Buffer methylammonium formate/methylamine (1 M, pH 8.8), $NADP^+$ (0.25 mM), FDH-QRN (5 µM), aminating enzyme (20 µM), substrate (10 mM), T= 30 °C, time= 24 h.

Table S27. Amination (%) of *rac*-**3b** using IREDs and AspRedAm in methylammonium formate/methylamine buffer (achiral GC measurement).

Enzyme	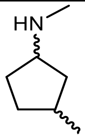	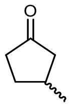	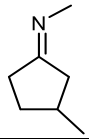
	**3d**	*rac*-**3b**	
Sp(S)-IRED	28	35	37
IRED-1	11	41	48
IRED-5	95	2	3
IRED-13	51	23	26
IRED-14	44	26	30
IRED-15	>99	<1	n.d.
IRED-20	>99	n.d.	n.d.
IRED-21	5	47	48
IRED-22	>99	n.d.	n.d.
IRED-30	98	1	1
IRED-32	6	44	50
AspRedAm	78	10	12
IRED-7	3	47	50
IRED-10	20	39	41
IRED-11	>99	n.d.	n.d.
IRED-25	n.d.	49	51
LE-AmDH-v1	n.d.	55	45

8.3. Substrate 3a – Cascade reaction

*8.3.1. Substrate **3a** – Cascade reaction using ammonium formate/ammonia buffer*

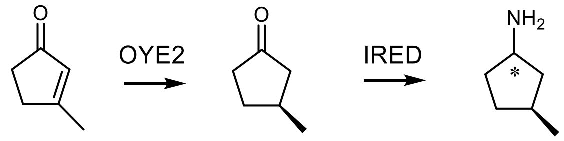

3a (*S*)-**3b** **3c**

Reaction conditions: 1 mL total volume in 2 mL Eppendorf tubes. Buffer ammonium formate/ammonia (1 M, pH 8), $NADP^+$ (0.5 mM), FDH-QRN (10 µM), OYE2 (25 µM), aminating enzyme (50 µM), substrate **3a** (10 mM), T= 30 °C, time= 22 h. Derivatization with benzoic anhydride/DMAP: benzoic anhydride (57.5 mg) and DMAP (15.3 mg) were dissolved in THF (250 µL). 20 µL were added into the biocatalytic reactions. They were incubated at 30 °C for 30 min. Then, $KHCO_3$ was added (1 mL of 10% solution) and incubated at 30 °C for further 30 min. After separation of the two layers, the organic phase was dried over anhydrous $MgSO_4$.

Full conversion and an *e.r.* of 99.7:0.3 for the OYE2 blank reaction were measured.

Table S28. Conversion and d.r. for the biocatalytic cascade of **3a** to **3c** combining OYE2 and IREDs in one pot (conversion was measured as non-derivatized sample, the d.e after derivatization with benzoic anhydride/DMAP).

			conversion (non-derivatized sample)			d.r. (derivatized sample with benzoic anhydride)	
substrate	ERed	IRed	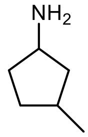	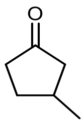	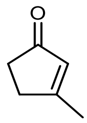	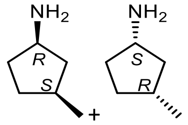	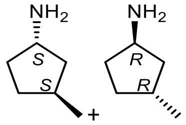
			**3c**	**3b**	**3a**	(1*R*,3*S*)-**3c**+(1*S*,3*R*)-**3c**	(1*S*,3*S*)-**3c**+(1*R*,3*R*)-**3c**
**3a**	OYE2	Sp(S)-IRED	n.d.	99	1	n.m.	n.m.
**3a**	OYE2	IRED-1	n.d.	99	1	n.m.	n.m.
**3a**	OYE2	IRED-5	n.d.	99	1	n.m.	n.m.
**3a**	OYE2	IRED-13	n.d.	99	1	n.m.	n.m.
**3a**	OYE2	IRED-14	n.d.	99	1	n.m.	n.m.
**3a**	OYE2	IRED-15	n.d.	99	1	n.m.	n.m.
**3a**	OYE2	IRED-20	11	88	1	86	14
**3a**	OYE2	IRED-20[(a)]	18	82	n.d.	86	14
**3a**	OYE2	IRED-21	n.d.	99	1	n.m.	n.m.
**3a**	OYE2	IRED-22	31	68	1	95	5
**3a**	OYE2	IRED-22[(a)]	44	56	n.d.	95	5
**3a**	OYE2	IRED-30	2	97	1	n.m.	n.m.
**3a**	OYE2	IRED-32	n.d.	99	1	n.m.	n.m.
**3a**	OYE2	IRED-7	n.d.	99	1	n.m.	n.m.
**3a**	OYE2	IRED-10	1	98	1	n.m.	n.m.
**3a**	OYE2	IRED-11	n.d.	99	1	n.m.	n.m.
**3a**	OYE2	IRED-25	n.d.	99	1	n.m.	n.m.

[(a)]IRED= 100 µM, FDH-QRN= 20 µM, $NADP^+$= 1 mM

*8.3.2. <u>Substrate **3a** – Cascade reaction using methylammonium formate/methylamine buffer</u>*

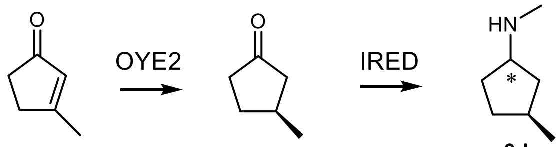

3a (*S*)-**3b** **3d**

<u>Reaction conditions one pot</u>: 1 mL total volume in 2 mL Eppendorf tubes. Buffer methylammonium formate/methylamine (1 M, pH 8), $NADP^+$ (0.5 mM), FDH-QRN (10 µM), OYE2 (25 µM), aminating enzyme (50 µM), substrate **3a** (10 mM), T= 30 °C, t= 24 h. Derivatization with benzoic anhydride/DMAP: benzoic anhydride (57.5 mg) and DMAP (15.3 mg) were dissolved in THF (250 µL). 20 µL were added into the biocatalytic reactions. They were incubated at 30 °C for 30 min. Then, $KHCO_3$ was added (1 mL of 10% solution) and incubated at 30 °C for further 30 min. After separation of the two layers, the organic phase was dried over anhydrous $MgSO_4$.

Full conversion and an *e.r.* of 99.6:0.4 for the OYE2 blank reaction were measured.

Table S29. Conversion and d.r. for the biocatalytic cascade of **3a** to **3d** combining OYE2 and IReds (conversion was measured as non-derivatized sample, the d.e after derivatization with benzoic anhydride/DMAP).

			conversion (non-derivatized sample)				d.r. (derivatized sample)	
substrate	ERed	IRed	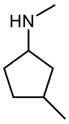	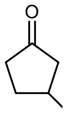	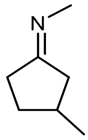	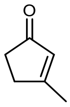	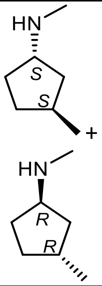	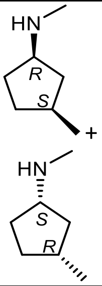
			**3d**	**3b**	**Imine of unreacted 3b and CH_3NH_3**	**3a**	(1*S*,3*S*)-**3d**+ (1*R*,3*R*)-**3d**	(1*R*,3*S*)-**3d**+ (1*S*,3*R*)-**3d**
**3a**	OYE2	IRED-5	93	4	3	n.d.	66.6	33.4
**3a**	OYE2	IRED-13	92	4	4	n.d.	73.3	26.7
**3a**	OYE2	IRED-14	61	20	19	n.d.	8.0	92.0
**3a**	OYE2	IRED-15	56	21	23	n.d.	96.2	3.8
**3a**	OYE2	IRED-20	99	1	n.d.	n.d.	1.1	98.9
**3a**	OYE2	IRED-22	99	1	n.d.	n.d.	2.2	97.8
**3a**	OYE2	IRED-30	73	14	13	n.d.	6.0	94.0
**3a**	OYE2	AspRedAm	74	13	13	n.d.	0.9	99.1
**3a**	OYE2	IRED-10	62	19	19	n.d.	92.0	8.0
**3a**	OYE2	IRED-11	99	1	n.d.	n.d.	19.3	80.7

GC-MS of sample **3a**+OYE2+IRED-5 was measured. Peak 1 and 2 were proven to be **3d** (m/z 113) and **3b** (m/z 98), whereas peak 3 gave a MS spectrum with parent peak having m/z 111. Further experiments demonstrated that this additional compound is not originated from any other enzymatic reaction (see next page); therefore, this peak is not related to the conceivable formation of unsaturated amine product. In contrast, it is the imine that is generated from the unreacted intermediate **3b** and methylamine upon extraction of the reaction mixture in an organic solvent.

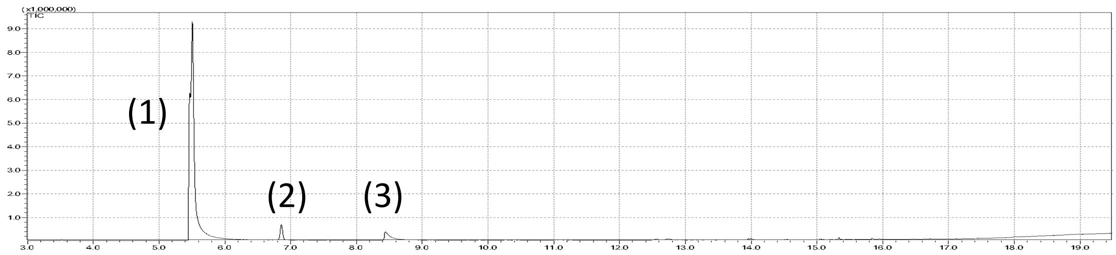

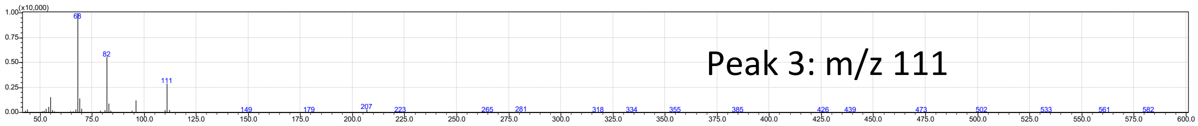

GC-FID chromatograms of the derivatized samples (with benzoic anhydride/DMAP, the mass of the derivatized samples was also confirmed by GC-MS)

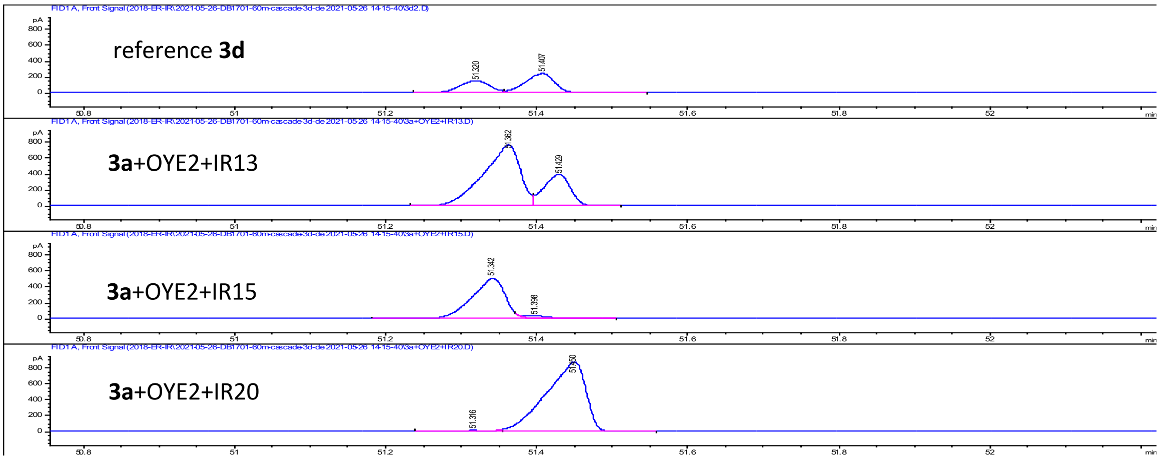

Experiment to elucidate the origin of the additionally observed compound

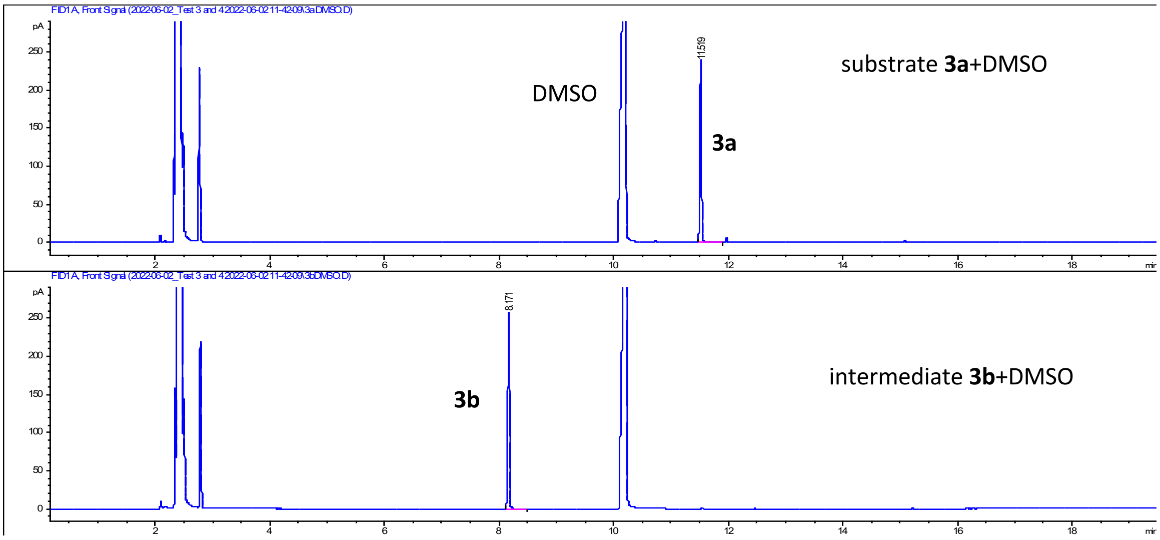

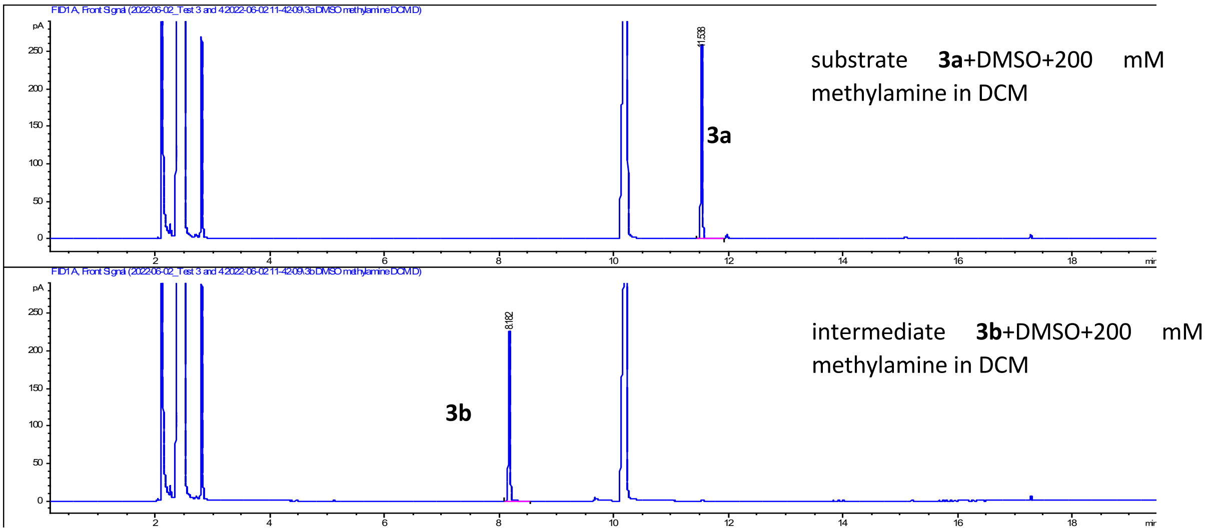

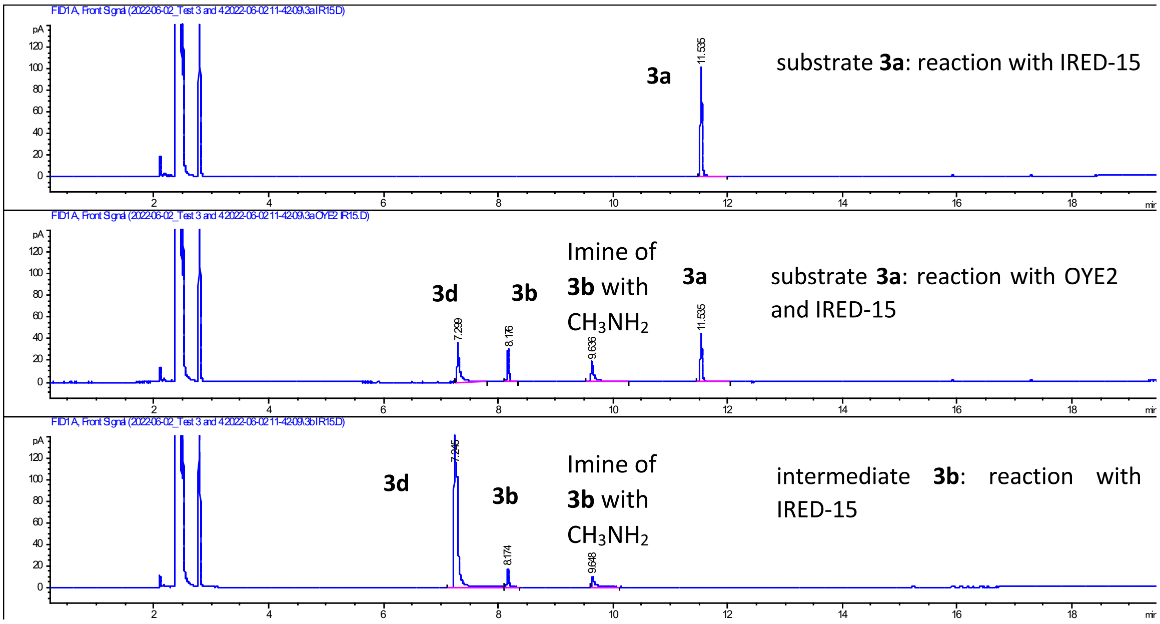

GC-MS analysis of reaction of **3a** with OYE2 and IRED-15

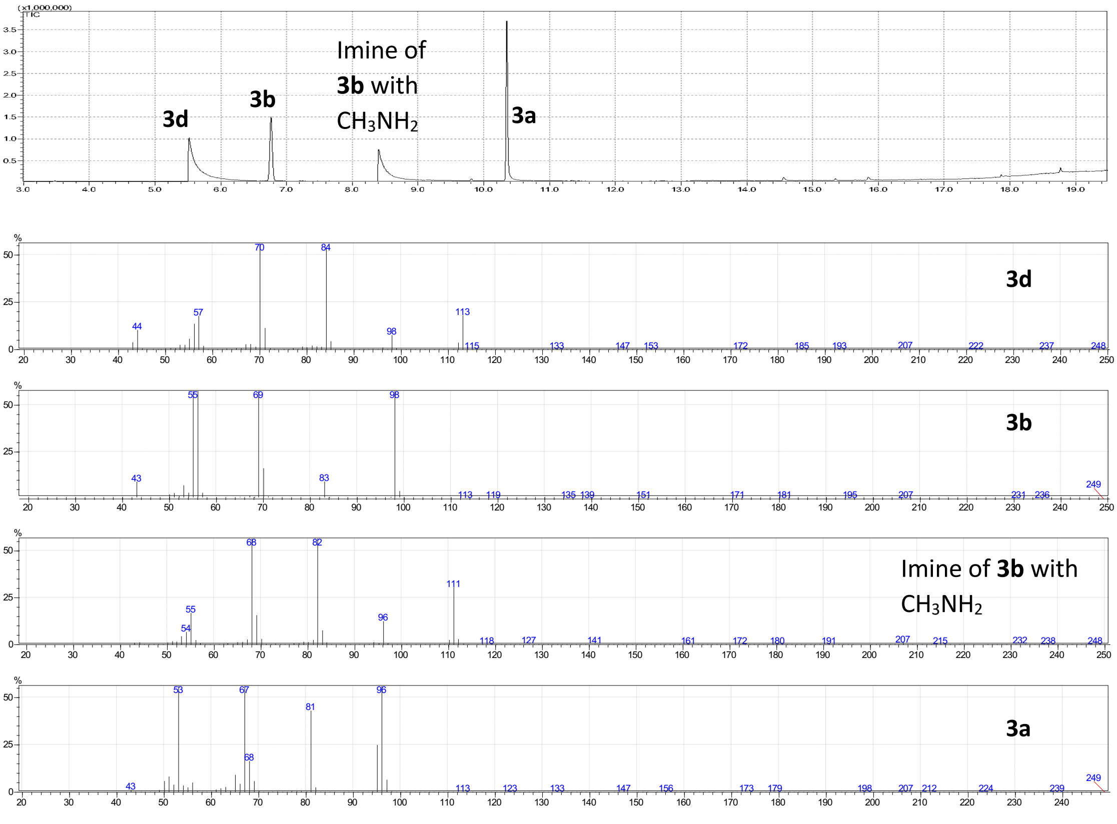

GC-MS analysis of reaction of **3b** with IRED-15

The new peak (peak 3) is the same as in the reaction of **3a** with OYE2 and IRED-15

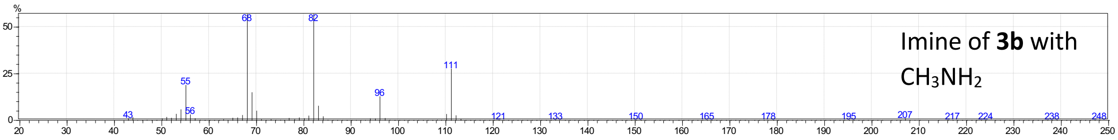

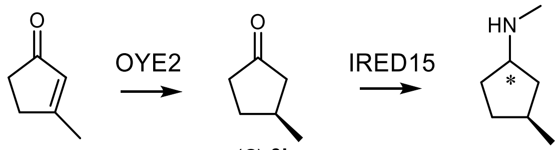

3a (*S*)-**3b** **3d** two sequential steps

<u>Reaction conditions two sequential steps</u>: (i) 1 mL total volume in 2 mL Eppendorf tubes. Buffer methylammonium formate/methylamine (1 M, pH 8), $NADP^+$ (0.5 mM), FDH-QRN (10 µM), OYE2 (25 µM), substrate **3a** (10 mM), T= 30 °C, time= 24 h. (ii) afterwards IRED-15 (50 µM and 100 µM), FDH-QRN (10 µM) and $NADP^+$ (0.5 mM) were added. T= 30 °C, time= 24 h. Derivatization with benzoic anhydride/DMAP: benzoic anhydride (57.5 mg) and DMAP (15.3 mg) were dissolved in THF (250 µL). 20 µL were added into the biocatalytic reactions. They were incubated at 30 °C for 30 min. Then, $KHCO_3$ was added (1 mL of 10% solution) and incubated at 30 °C for further 30 min. After separation of the two layers, the organic phase was dried over anhydrous $MgSO_4$.

Full conversion and an *e.r.* of 99.6:0.4 for the OYE2 blank reaction were measured.

Table S30. Conversion and d.r. for the biocatalytic cascade of **3a** to **3d** combining OYE2 and IRED-15 in two sequential steps (conversion was measured as non-derivatized sample, the d.r. after derivatization with benzoic anhydride/DMAP).

				conversion (non-derivatized sample)				d.r. (derivatized sample)	
substrate	ERed	IRed	IRed (µM)	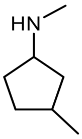	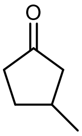	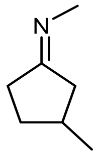	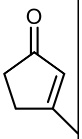		
				**3d**	**3b**	**Imine of unreacted 3b and CH_3NH_3**	**3a**		
**3a**	OYE2	IRED-15	50	94	6	n.d.	n.d.	96.6	3.4
**3a**	OYE2	IRED-15	100	>99	<1	n.d.	n.d.	96.9	3.1

9. Biocatalytic reactions performed for substrate 4a

9.1. Substrate 4a - Stereoselective reduction of the double bond (ERed-reaction)

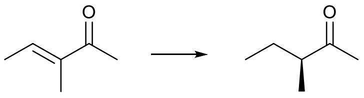

4a (S)-4b

Reaction conditions: 1 mL total volume in 2 mL Eppendorf tubes. Buffer KPi (50 mM, pH 8.0) or ammonium formate/ammonia buffer (1M) or methylammonium formate/methylamine buffer (1 M), $NADP^+$ (0.25 mM), sodium formate (30 mM, only for KPi buffer), FDH-QRN (5 µM), ERed (5-20 µM), substrate **1a** (10 mM), T= 10, 20 or 30 °C, time= 22 h. NerA and MR were also tested with NAD^+ (0.25 mM) in combination with Cb-FDH (5 µM).

Table S31. Conversion and *e.r.* for **4a** using EReds.

ERed	ERed (µM)	T (°C)	buffer	pH	**4b**	**4a**	side product (most likely alcohol)	*e.r.* **3b**[a]
Blank		30	KPi	8	n.d.	99	1	n.m.
PETNR	5	30	KPi	8	97	<1	3	57:43 (*S*)
TOYE	5	30	KPi	8	97	3	n.m.	95:5 (*S*)
OYE2	5	30	KPi	8	95	2	3	84:16 (*S*)
OYE3	5	30	KPi	8	97	n.d.	3	94:6 (*S*)
XenA	5	30	KPi	8	97	2	<1	98.7:1.3 (*S*)
XenA	10	20	KPi	8	98	2	n.d.	99.0:1.0 (*S*)
XenA	10	10	KPi	8	97	3	n.d.	99.1:0.9 (*S*)
XenA	20	10	KPi	7	98	2	n.d.	99.1:0.9 (*S*)
XenA	10	30	$HCOONH_4/NH_3$	9	>99	n.d.	n.d.	91:9 (*S*)
XenA	10	20	$HCOONH_4/NH_3$	8.8	>99	n.d.	n.d.	96.7:3.3 (*S*)
XenA	10	10	$HCOONH_4/NH_3$	8.8	>99	n.d.	n.d.	98.2:1.8 (*S*)
XenA	10	20	$HCOONH_4/NH_3$	8	>99	n.d.	n.d.	98.5:1.5 (*S*)
XenA	10	10	$HCOONH_4/NH_3$	8	99	1	n.d.	98.9:1.1 (*S*)
XenA	10	30	$HCOOCH_3NH_3/CH_3NH_2$	9	>99	n.d.	n.d.	96.2:3.8 (*S*)
XenA	10	20	$HCOOCH_3NH_3/CH_3NH_2$	8.8	>99	n.d.	n.d.	98.1:1.9 (*S*)
XenA	10	10	$HCOOCH_3NH_3/CH_3NH_2$	8.8	>99	n.d.	n.d.	98.7:1.3 (*S*)
XenA	10	20	$HCOOCH_3NH_3/CH_3NH_2$	8	>99	n.d.	n.d.	98.9:1.1 (*S*)
XenA	10	10	$HCOOCH_3NH_3/CH_3NH_2$	8	>99	n.d.	n.d.	99.1:0.9 (*S*)
XenB	5	30	KPi	8	97	<1	3	76:24 (*S*)
LeOPR1	5	30	KPi	8	97	2	<1	58:42 (*S*)
NerA	5	30	KPi	8	61	37	2	59:41 (*S*)
NerA NAD	5	30	KPi	8	92	5	3	59:41 (*S*)
GluOx	5	30	KPi	8	97	<1	3	65:35 (*S*)
YqjM	5	30	KPi	8	97	3	<1	97:3 (*S*)
YqjM	10	30	KPi	8	98	2	n.d.	97:3 (*S*)
YqjM	10	40	KPi	8	98	2	n.d.	96:4 (*S*)
MR	5	30	KPi	8	3	96	1	n.m.
MR NAD	5	30	KPi	8	68	31	1	50:50 (*S*)
YqjM-v1	5	30	KPi	8	99	1	n.d.	73:27 (*R*)
YqjM-v1	10	30	NH_3	8.8	>99	n.d.	n.d.	76:24 (*R*)
YqjM-v1	10	30	$HCOOCH_3NH_3/CH_3NH_2$	8.8	>99	n.d.	n.d.	76:24 (*R*)

[a] The enantiomeric ratio values were reported with one significant decimal digit in selected cases where a high selectivity was found. The enantiomeric ratio was calculated based on the detection limit (2 area units) of the GC.

The configuration of **4b** was determined by measuring the optical rotation (paragraph 12.4.1)

Chiral GC-FID chromatograms for the separation of the **4b** enantiomers

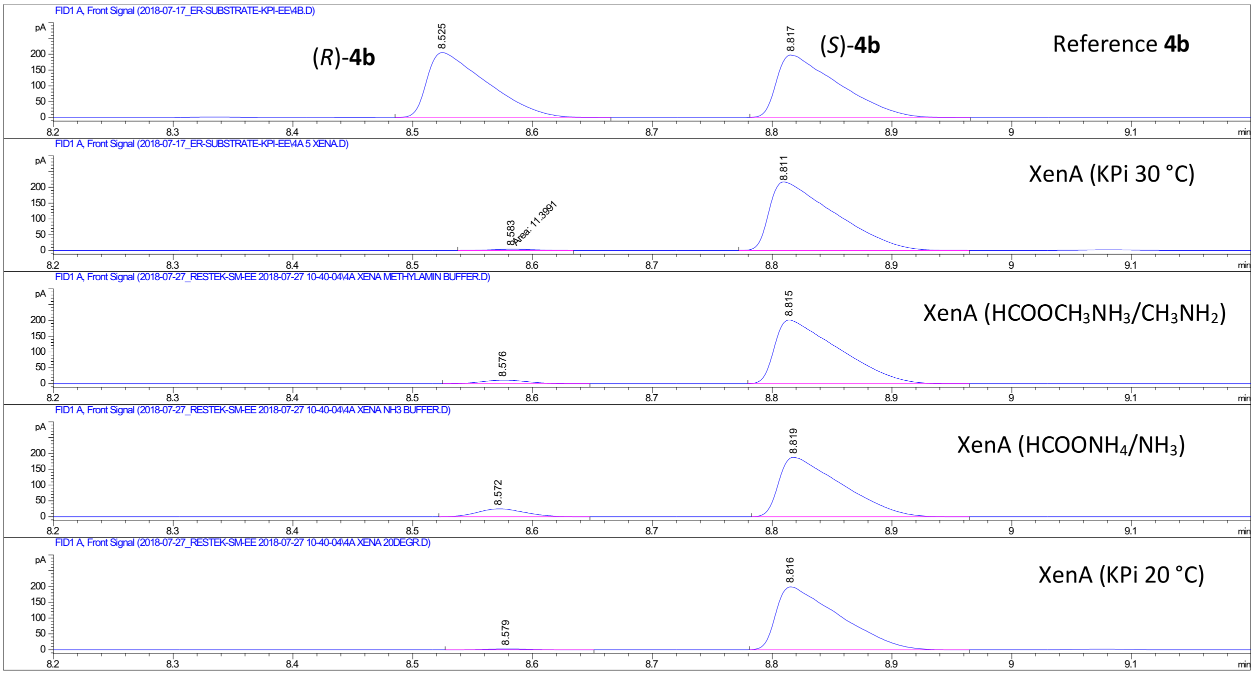

9.1. Substrate 4b - Amination

9.1.1. Substrate **4b** - Amination using ammonium formate/ammonia buffer

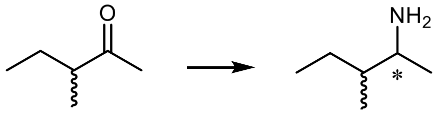

rac-**4b** **4c**

Reaction conditions: 1 mL total volume in 2 mL Eppendorf tubes. Buffer ammonium formate/ammonia (1 M, pH 8.8), $NADP^+$ (0.25 mM, in the case of Ch1-AmDH and Rh-PhAmDH, NAD^+ was used), FDH-QRN (5 µM, in the case of Ch1-AmDH and Rh-PhAmDH, Cb-FDH was used), aminating enzyme (20 µM), substrate *rac*-**4b** (10 mM), T= 30 °C, time= 24 h.

Table S32. Amination (%) of *rac*-**4b** using IReds, AspRedAm and AmDHs in ammonium formate/ammonia buffer (achiral GC measurement).

enzyme	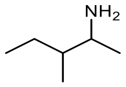	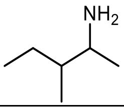	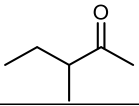
	**4c**	**4c**	rac-**4b**
Ch1-AmDH	29	29	42
Rh-PhAmDH	n.d.	n.d.	>99
Sp(S)-IRED	n.d.	n.d.	>99
IRED-1	n.d.	n.d.	>99
IRED-5	n.d.	n.d.	>99
IRED-13	n.d.	n.d.	>99
IRED-14	1	4	95
IRED-15	4	5	91
IRED-20	21	10	69
IRED-21	n.d.	n.d.	>99
IRED-22	6	8	86
IRED-30	n.d.	n.d.	>99
IRED-32	<1	n.d.	>99
AspRedAm	n.d.	n.d.	>99
IRED-7	n.d.	n.d.	>99
IRED-10	n.d.	n.d.	>99
IRED-11	n.d.	n.d.	>99
IRED-25	n.d.	n.d.	>99
LE-AmDH-v1	<1	n.d.	>99

9.1.1. Substrate **4b** - Amination using methylammonium formate/methylamine buffer

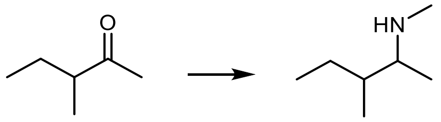

rac-**4b** **4d**

Reaction conditions: 1 mL total volume in 2 mL Eppendorf tubes. Buffer methylammonium formate/methylamine (1 M, pH 8.8), $NADP^+$ (0.25 mM), FDH-QRN (5 µM), aminating enzyme (20 µM), substrate *rac*-**4b** (10 mM), T= 30 °C, time= 24 h.

Table S33. Amination (%) of *rac*-**4b** using IReds and AspRedAm with methylammonium formate/methylamine buffer (GC measurement with an achiral column).

enzyme	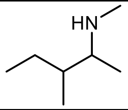	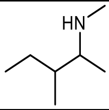	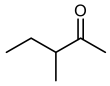	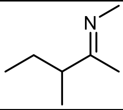
	**4d**	**4d**	rac-**4b**	
Sp(S)-IRED	n.d.	n.d.	87	13
IRED-1	n.d.	n.d.	86	14
IRED-5	5	1	81	13
IRED-13	5	7	77	12
IRED-14	5	21	64	10
IRED-15	3	1	83	13
IRED-20	22	29	42	7
IRED-21	n.d.	n.d.	86	14
IRED-22	5	6	77	13
IRED-30	4	7	77	12
IRED-32	14	8	68	11
AspRedAm	n.d.	n.d.	86	14
IRED-7	n.d.	n.d.	88	12
IRED-10	n.d.	n.d.	87	13
IRED-11	3	n.d.	84	13
IRED-25	n.d.	n.d.	87	13
LE-AmDH-v1	n.d.	n.d.	87	13

n.d. = not detected (below the detection limit)

9.1. Substrate 4a – Cascade reaction

*9.1.1. Substrate **4a** – Cascade reaction using ammonium formate/ammonia buffer*

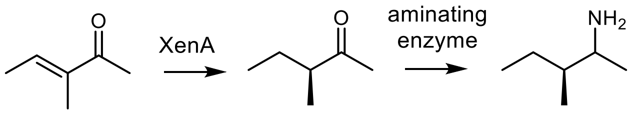

4a (*S*)-**4b** **4d**

To keep the *e.r.* of the saturated intermediate high, the reaction was performed in two sequential steps.

Reaction conditions for the cascade reaction performed in two sequential steps: (i) ERed-reaction in 1 mL reaction volume (in 2 mL Eppendorf tubes). Buffer (KPi, 50 mM, pH 7.1), XenA (30 µM), $NADP^+$ (0.25 mM), FDH-QRN (5 µM), sodium formate (30 mM). substrate **4a** (10 mM). The reactions were incubated for 24 h at 20 °C under horizontal shaking. (ii) Amination, total reaction volume is 2 mL. 1 mL of following reaction composition was added: buffer ammonium formate/ammonia (1 M, pH 8.4), aminating enzyme (50 or 100 µM), $NADP^+$ for IRED20 or NAD^+ for Ch1-AmDH (0.5 mM), FDH-QRN for IRED20 or Cb-FDH for Ch1-AmDH (10 µM). The reactions were run for further 23 h at 30, 40 or 50 °C. Note: the final concentration of the reactant in the second step was half.

For the XenA reaction, 98% conversion and an *e.r.* of 99:1 for (*S*)-**4b** were determined.

Table S34. Conversion (%) for the biocatalytic cascade of **4a** to **4c** combining XenA and aminating enzymes in two sequential steps (GC-FID measurement with an achiral column).

	ERed	IRed or AmDH	µM	T (°C)	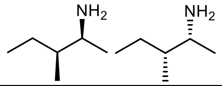	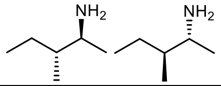	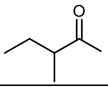	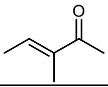
					(2*S*,3*S*)-**4c** + (2*R*,3*R*)-**4c**	(2*S*,3*R*)-**4c** + (2*R*,3*S*)-**4c**	**4b**	**4a**
**4a**	XenA	IRED-20	50	30	40	11	49	<1
**4a**	XenA	Ch1-AmDH	50	30	<1	49	50	n.d.
**4a**	XenA	Ch1-AmDH	100	30	<1	51	48	n.d.
**4a**	XenA	Ch1-AmDH	100	40	<1	72	27	n.d.
**4a**	XenA	Ch1-AmDH	100	50	>1	90	9	n.d.

Table S35. Diastereomeric and enantiomeric composition (%) for **4c** (GC-FID measurement with a chiral column) after derivatization to the acetamide.

substrate	ERed	IRed or AmDH	µM	T (°C)	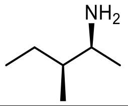	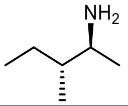	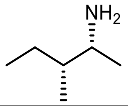	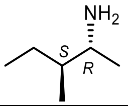
					(2*S*,3*S*)-**4c**	(2*S*,3*R*)-**4c**	(2*R*,3*R*)-**4c**	(2*R*,3*S*)-**4c**
**4a**	XenA	IRED-20	50	30	78.2	0.5	1.8	19.5
**4a**	XenA	Ch1-AmDH	50	30	n.d.	n.d.	1.4	98.6
**4a**	XenA	Ch1-AmDH	100	30	n.d.	n.d.	1.3	98.7
**4a**	XenA	Ch1-AmDH	100	40	n.d.	n.d.	1.3	98.7
**4a**	XenA	Ch1-AmDH	100	50	n.d.	n.d.	1.7	98.3

GC-FID chromatograms (chiral) of the cascade reaction using XenA in combination with Ch1-AmDH.

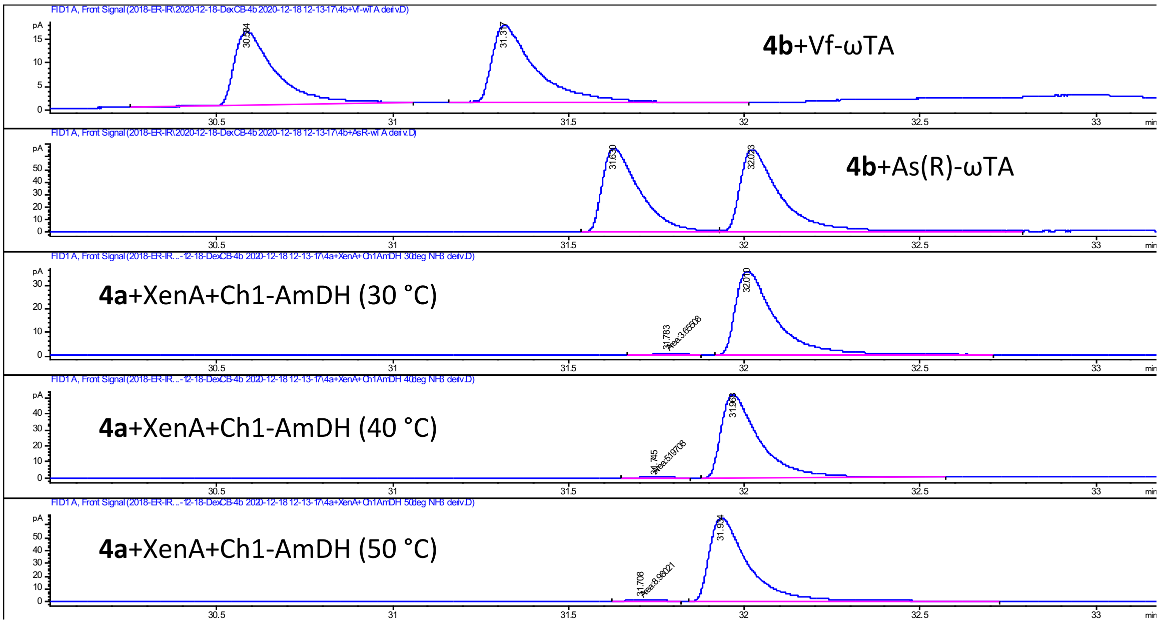

9.1.2. *Substrate **4a** – Cascade reaction using methylammonium formate/methylamine buffer*

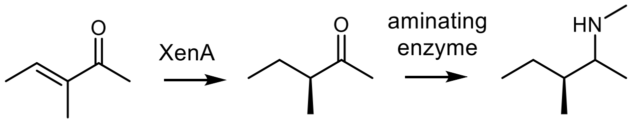

4a (*S*)-**4b** **4d**

(A) Test reaction

(1) Reaction conditions for the cascade reaction performed in two-steps: (i) ERed-reaction in 1 mL reaction volume (in 2 mL Eppendorf tubes). Buffer (methylammonium formate/methylamine, 1 M, pH 8), XenA (20 µM), $NADP^+$ (0.25 mM), FDH-QRN (5 µM), sodium formate (30 mM) and substrate **4a** (10 mM). The reactions were incubated for 23 h at 20 °C under horizontal shaking. (ii) Amination, total reaction volume is 2 mL. 1 mL of following reaction composition was added: buffer (methylamine/formic acid, 1 M, pH 8), aminating enzyme (50 µM), $NADP^+$ (0.5 mM), FDH-QRN (10 µM). The reactions were run for further 23 h at 30 °C. Note: the final concentration of the reactants in the second step is half.

98% conversion and an *e.r.* of 96:4 for the XenA blank reaction were measured. However, after the amination step the *e.r.* of the remaining intermediate dropped down to 89:11.

Table S36. Conversion (%) for the biocatalytic cascade of **4a** to **4d** combining XenA and IREDs in two sequential steps (GC-FID measurement with an achiral column) as well as the *e.r.* of the remaining intermediate measured with an achiral GC column.

substrate	ERED	IRED	achiral GC-FID 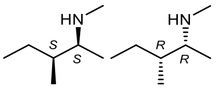	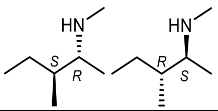	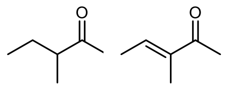		chiral GC-FID
			(2*S*,3*S*)-**4d** + (2*R*,3*R*)-**4d**	(2*S*,3*R*)-**4d** + (2*R*,3*S*)-**4d**	**4b**	**4a**	e.r. (*S*)-**4b**
**4a**	XenA	IRED5	40	<1	59	n.d.	89:11
**4a**	XenA	IRED13	50	2	48	n.d.	90:10
**4a**	XenA	IRED14	16	3	81	n.d.	90:10
**4a**	XenA	IRED20	38	9	53	n.d.	89:11
**4a**	XenA	IRED22	12	41	47	n.d.	89:11
**4a**	XenA	IRED30	38	9	53	n.d.	89:11
**4a**	XenA	IRED32	40	<1	59	n.d.	89:11

(B) Variation of reaction conditions using most promising IReds from (A)

(1) Reaction conditions for the cascade reaction performed in one-step: 1 mL reaction volume (in 2 mL Eppendorf tubes). Buffer (methylammonium formate/methylamine, 1 M, pH 7 or pH 8), XenA (12 µM), $NADP^+$ (0.5 mM), FDH-QRN (10 µM), IRed (30 µM), substrate **4a** (10 mM). The reactions were incubated for 23 h at 30 °C under horizontal shaking.

(2) Reaction conditions for the cascade reaction performed in two sequential steps: (i) ERed-reaction 1 mL reaction volume (in 2 mL Eppendorf tubes). Buffer (KPi, 50 mM, pH 7), XenA (12 µM), $NADP^+$ (0.25 mM), FDH-QRN (5 µM), sodium formate (30 mM) and substrate **4a** (10 mM). The reactions were incubated for 23 h at 20 °C under horizontal shaking. (ii) Amination, total reaction volume is 2 mL. 1 mL of following reaction composition was added: buffer (methylammonium formate/methylamine, 1 M, pH 8), aminating enzyme (50 µM), $NADP^+$ (0.5 mM), FDH-QRN (10 µM). The reactions were run for further 23 h at 30 °C. Note: the final concentration of the reactants in the second step was half.

Table S37. Conversion and *e.r.* for the XenA blank reactions under different reaction conditions.

Substrate	enzyme	buffer	pH	4b	*e.r.* (*S*)-4b
**4a**	XenA	KPi	7	98	98.9:1.1
**4a**	XenA	$HCOONH_4/NH_3$	7	>99	98.6:1.4
**4a**	XenA	$HCOONH_4/NH_3$	8	>99	98.6:1.4

Table S38. Conversion (%) for the biocatalytic cascade of **4a** to **4d** combining XenA and IRess (GC-FID measurement with an achiral column).

substrate	ERed	IRed	type of cascade	pH	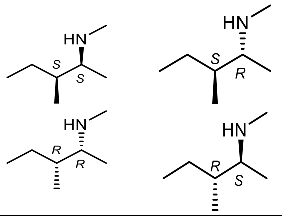		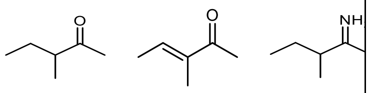		
					(2*S*,3*S*)-**4d** + (2*R*,3*R*)-**4d**	(2*S*,3*R*)-**4d** + (2*R*,3*S*)-**4d**	**4b**	**4a**	**Imine of 4b with CH_3NH_2**
	**parallel pH 7**								
**4a**	XenA	IRED-5	concurrent	7	29	1	64	n.d.	6
**4a**	XenA	IRED-5	concurrent	8	15	<1	73	n.d.	11
**4a**	XenA	IRED-5	sequential	7	26	<1	68	n.d.	5
**4a**	XenA	IRED-5	sequential	8	26	<1	68	n.d.	5
**4a**	XenA	IRED-13	concurrent	7	25	1	65	n.d.	9
**4a**	XenA	IRED-13	concurrent	8	22	1	67	n.d.	10
**4a**	XenA	IRED-13	sequential	7	33	2	61	n.d.	4
**4a**	XenA	IRED-13	sequential	8	33	2	61	n.d.	4
**4a**	XenA	IRED-32	concurrent	7	26	<1	64	n.d.	9
**4a**	XenA	IRED-32	concurrent	8	28	<1	62	n.d.	9
**4a**	XenA	IRED-32	sequential	7	29	<1	65	n.d.	5
**4a**	XenA	IRED-32	sequential	8	29	1	63	n.d.	5

As in the case of the biocatalytic reactions with substrates **3a**, a new peak was observed for the reaction with **4a**. This was proven to be again the imine of unreacted intermediate **4b** and methylamine that is spontaneously formed upon extraction in an organic solvent (see below).

Experiment to elucidate the origin of the additionally observed compound

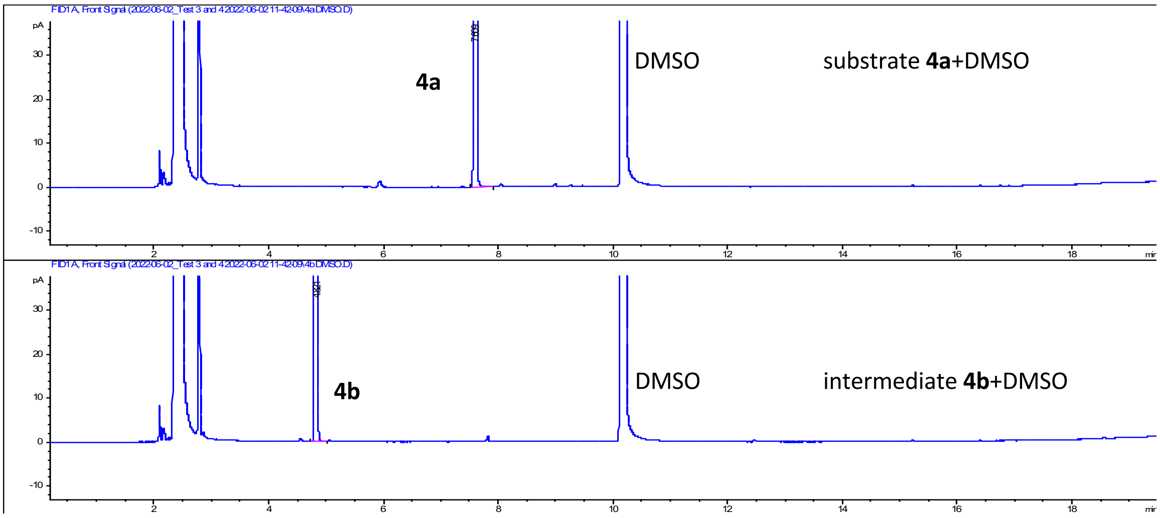

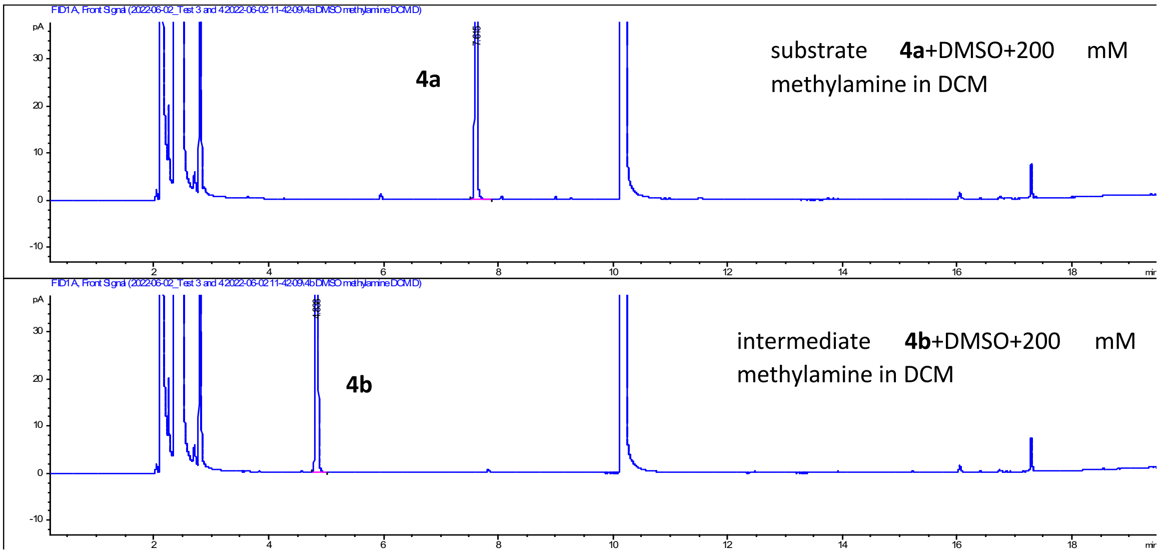

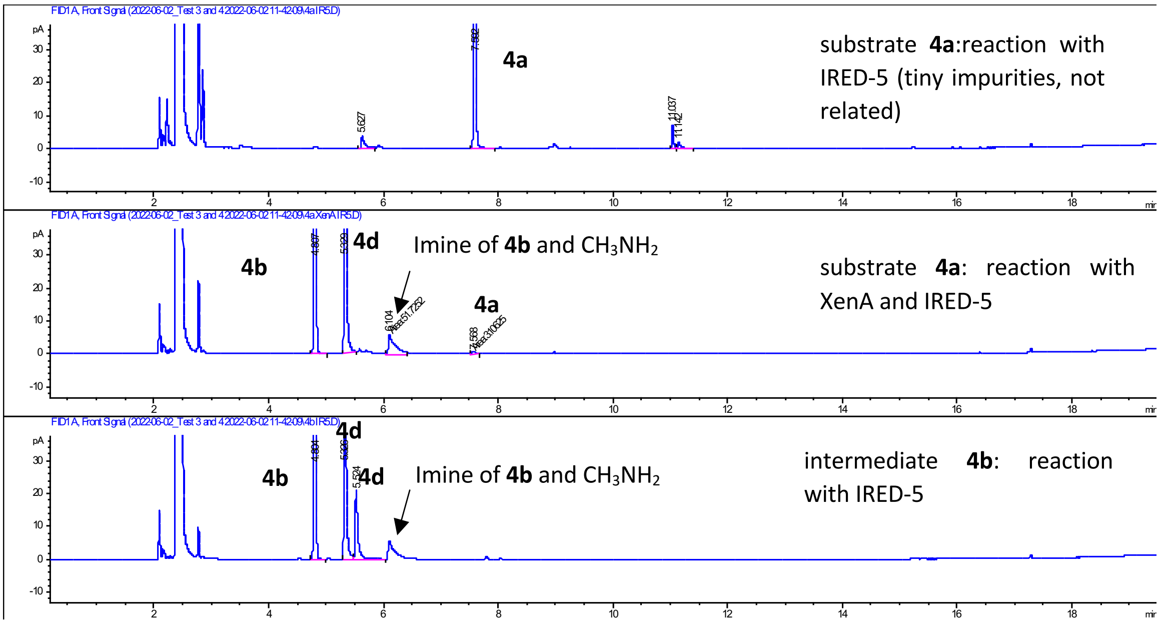

GC-MS analysis of reaction of **4a** with XenA and IRED-5

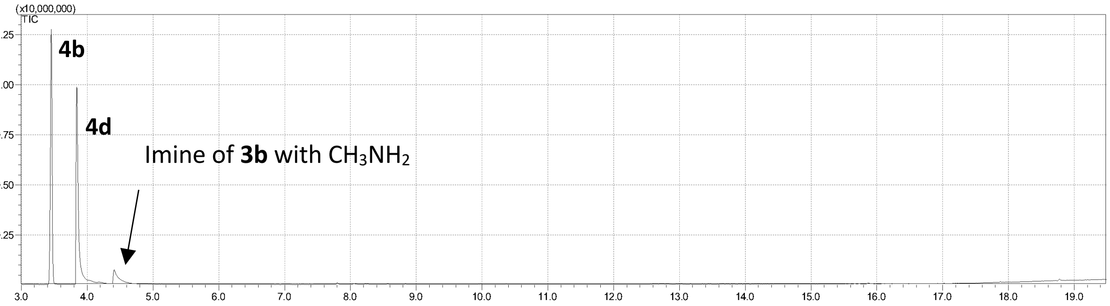

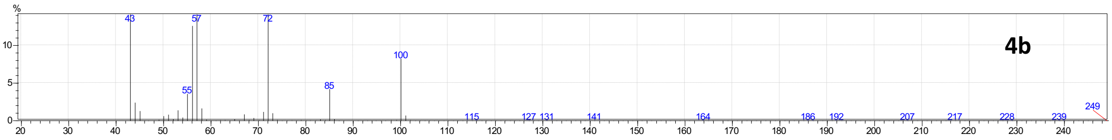

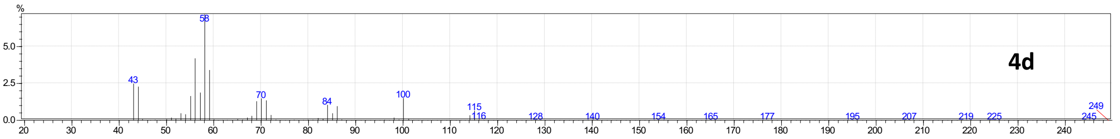

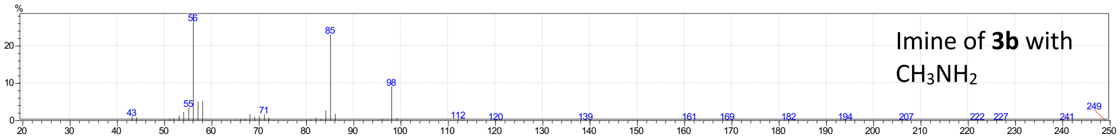

GC-MS analysis of reaction of **4b** with IRED-5

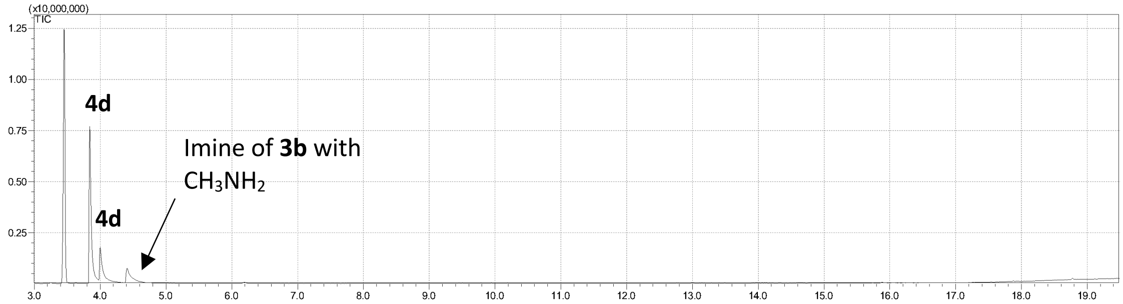

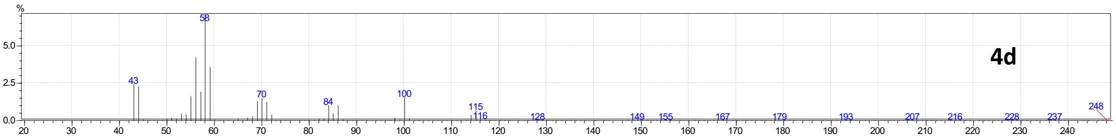

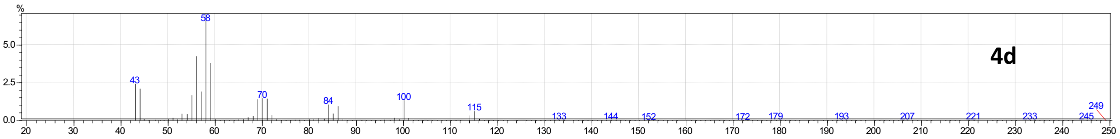

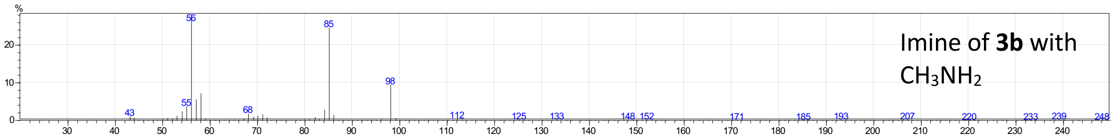

Table S39. Diastereomeric and enantiomeric composition (%) for **4c** (GC-FID measurement with a chiral column) after derivatization to the acetamide.

substrate	ERed	IRED	type of cascade	pH	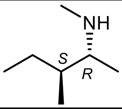	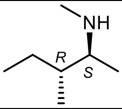	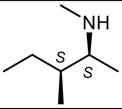	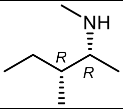
					(2*S*,3*R*)-**4d**	(2*R*,3*S*)-**4d**	(2*S*,3*S*)-**4d**	(2*R*,3*R*)-**4d**
**4a**	XenA	IRED-5	concurrent	7	0.5	0.6	98.9	n.d.
**4a**	XenA	IRED-5	concurrent	8	n.d.	0.4	99.6	n.d.
**4a**	XenA	IRED-5	sequential	7	n.d.	0.7	99.3	n.d.
**4a**	XenA	IRED-5	sequential	8	n.d.	0.8	99.2	n.d.
**4a**	XenA	IRED-13	concurrent	7	1.0	2.3	96.7	n.d.
**4a**	XenA	IRED-13	concurrent	8	0.9	2.3	96.8	n.d.
**4a**	XenA	IRED-13	sequential	7	1.0	3.1	95.9	n.d.
**4a**	XenA	IRED-13	sequential	8	0.6	2.3	97.1	n.d.
**4a**	XenA	IRED-32	concurrent	7	0.3	n.d.	99.7	n.d.
**4a**	XenA	IRED-32	concurrent	8	0.5	n.d.	99.5	n.d.
**4a**	XenA	IRED-32	sequential	7	0.6	n.d.	99.4	n.d.
**4a**	XenA	IRED-32	sequential	8	0.6	n.d.	99.4	n.d.

(C) Improvement of conversion

<u>(2) Reaction conditions for the cascade reaction performed in two sequential steps</u>: (i) ERed-reaction in 1 mL reaction volume (in 2 mL Eppendorf tubes). Buffer (KPi, 50 mM, pH 7 or methylammonium formate/methylamine, 1 M, pH 8), XenA (30 µM), $NADP^+$ (0.25 mM), FDH-QRN (5 µM), sodium formate (30 mM) and substrate **4a** (10 mM). The reactions were incubated for 22 h at 20 or 30°C under horizontal shaking. (iia) Amination, total reaction volume is 2 mL. 1 mL of following reaction composition was added to the XenA reactions that were performed in KPi buffer: buffer (methylammonium formate/methylamine, 1 M, pH 8), aminating enzyme (50 µM or 100 µM), $NADP^+$ (0.5 mM), FDH-QRN (10 µM). (iib) For the amination to the XenA reactions that were performed in methylammonium formate/methylamine buffer, only IRED (50 µM), $NADP^+$ (0.5 mM) and FDH-QRN (10 mM) were added. (iic) For the amination to the XenA reactions that were performed in methylammonium formate/methylamine buffer, 1 mL of following reaction composition was added: buffer (methylammonium formate/methylamine, 1 M, pH 8), aminating enzyme (50 µM), $NADP^+$ (0.5 mM), FDH-QRN (10 µM). The reactions were run for further 26 h at 30°C.

Table S40. Conversion and *e.r.* for the XenA blank reactions under different reaction conditions.

substrate	enzyme	buffer	pH	T (°C)	**4b**	*e.r.* (*S*)-**4b**
**4a**	XenA	KPi	7	20	98	99.1:0.9
**4a**	XenA	KPi	7	30	98	98.7:1.3
**4a**	XenA	$HCOONH_4/NH_3$	8	20	>99	99.1:0.9
**4a**	XenA	$HCOONH_4/NH_3$	8	30	>99	98.6:1.4

Table S41. Conversion (%) for the biocatalytic cascade of **4a** to **4d** combining XenA and IREDs (GC-FID measurement with an achiral column).

substrate	T for XenA reaction (°C)	IRed	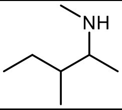	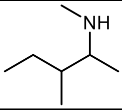	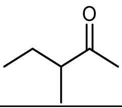	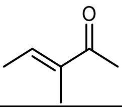	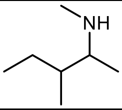
			**4d**-peak1	**4d**-peak2	**4b**	**4a**	**Imine of 4b with CH_3NH_2**
**XenA KPi pH 7.0 50 µM IRED**							
**4a**	20 °C	IRED-5	32	<1	62	<1	5
**4a**	30 °C	IRED-5	33	<1	62	<1	5
**XenA KPi pH 7.0 50 µM IRED**							
**4a**	20 °C	IRED-32	38	1	56	<1	5
**4a**	30 °C	IRED-32	38	1	58	<1	3
**XenA KPi pH 7.0 100 µM IRED**							
**4a**	20 °C	IRED-5	39	<1	56	<1	4
**4a**	30 °C	IRED-32	45	1	51	<1	3
**XenA methylamine pH 8.0 1mL**							
**4a**	20 °C	IRED-5	39	<1	53	<1	7
**4a**	30 °C	IRED-5	37	<1	55	<1	7
**XenA methylamine pH 8.0 1mL**							
**4a**	20 °C	IRED-32	47	<1	46	<1	6
**4a**	30 °C	IRED-32	43	<1	50	<1	7
**XenA methylamine pH 8.0 2mL**							
**4a**	20 °C	IRED-5	55	<1	39	<1	5
**4a**	30 °C	IRED-32	48	<1	44	<1	6

Table S42. Diastereomeric and enantiomeric composition (%) for **4c** (GC-FID measurement with a chiral column) after derivatization to the acetamide.

substrate	T for XenA reaction (°C)	IRed	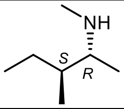	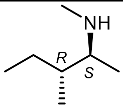	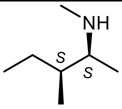	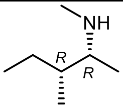
			(2*S*,3*R*)-**4d**	(2*R*,3*S*)-**4d**	(2*S*,3*S*)-**4d**	(2*R*,3*R*)-**4d**
**XenA KPi pH 7.0 50 µM IRED**						
**4a**	20 °C	IRED-5	0.2	0.8	99.0	Tiny traces
**4a**	30 °C	IRED-5	0.3	0.8	98.7	0.2
**XenA KPi pH 7.0 50 µM IRED**						
**4a**	20 °C	IRED-32	0.7	n.d.	99.3	n.d.
**4a**	30 °C	IRED-32	1.0	n.d.	99.0	n.d.
**XenA KPi pH 7.0 100 µM IRED**						
**4a**	20 °C	IRED-5	0.3	0.8	99.0	Tiny trace
**4a**	30 °C	IRED-32	1.0	n.d.	99.0	n.d.
**XenA methylamine pH 8.0 1mL**						
**4a**	20 °C	IRED-5	0.2	0.7	99.1	Tiny trace
**4a**	30 °C	IRED-5	0.4	0.9	98.4	0.3
**XenA methylamine pH 8.0 1mL**						
**4a**	20 °C	IRED-32	0.6	n.d.	99.4	n.d.
**4a**	30 °C	IRED-32	0.9	n.d.	99.1	n.d.
**XenA methylamine pH 8.0 2mL**						
**4a**	20 °C	IRED-5	0.3	1.0	98.5	0.2
**4a**	30 °C	IRED-32	1.0	n.d.	99.0	n.d.

Chiral GC-FID chromatograms of the IRED-32 reactions

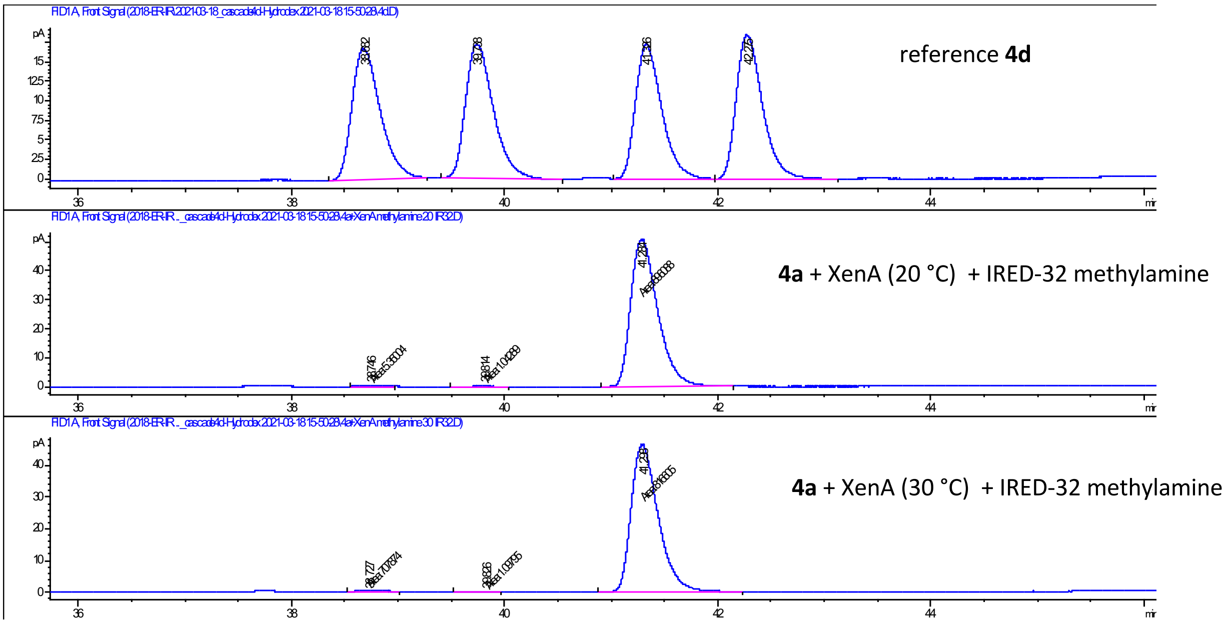

10. Biocatalytic reactions using further amine donor

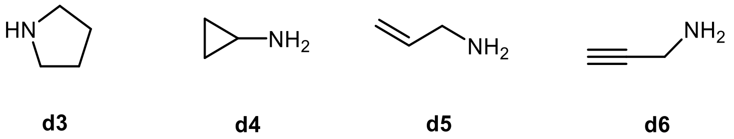

Figure S4. Additional amine donor used.

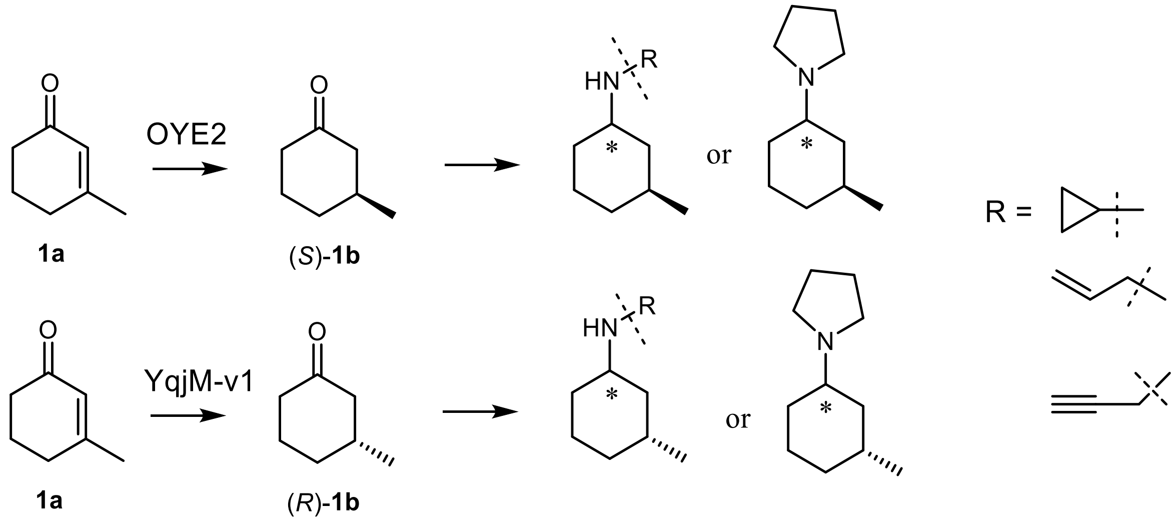

Figure S5. Reaction scheme for the biocatalytic reaction using substrate **1a** in a two sequential step reaction combining OYE2 with aminating enzymes with different amine donor

<u>**Biocatalytic reactions were run in two sequential steps:**</u>

(i) ERed-reaction 1 mL reaction volume (in 2 mL Eppendorf tubes)

<u>substrate 1</u>: buffer (KPi, 50 mM, pH 7.5), OYE2 (15 µM) or YqjM-v1 (20 µM), $NADP^+$ (0.5 mM), FDH-QRN (5 µM), sodium formate (30 mM) and substrate **1a** (10 mM). The reactions were run for 24 h at 30 °C. Conversion for OYE2 was >99% (*e.r.* >99.5:<0.5 (*S*)-**1b**) and >99% for YqjM-v1 (*e.r.* >97:<3 (*R*)-**1b**).

<u>substrate 2</u>: buffer (KPi, 50 mM, pH 7), TOYE (40 µM), $NADP^+$ (0.5 mM), FDH-QRN (10 µM), sodium formate (30 mM) and substrate **2a** (10 mM). The reactions were run for 24 h at 10 °C. Conversion for TOYE 93% (*e.r.* >99:<1 (*R*)-**2b**).

<u>substrate 3</u>: buffer (KPi, 50 mM, pH 7), OYE2 (30 µM), $NADP^+$ (0.5 mM), FDH-QRN (10 µM), sodium formate (30 mM) and substrate **3a** (10 mM). The reactions were run for 24 h at 30 °C. Conversion for OYE2 59% (*e.r.* >99:<1 (*S*)-**3b**).

<u>substrate 4</u>: buffer (KPi, 50 mM, pH 7), XenA (15 µM), $NADP^+$ (0.5 mM), FDH-QRN (5 µM), sodium formate (30 mM) and substrate **4a** (10 mM). The reactions were run for 24 h at 20 °C. Conversion for XenA 98% (*e.r.* >99:<1 (*S*)-**4b**).

(ii) Amination, total reaction volume is 2 mL:

1 mL of following reaction composition was added to the ERED reaction: buffer containing amine donor **d3-6** (100 mM, pH 8.5-8.9), IRed (40 µM), $NADP^+$ (0.25 mM), FDH-QRN (5 µM) and sodium formate (30 mM). The reactions were run for further 23 h at 30 °C. Note: the final concentration of the reactants in the second step was half. Buffer preparation: 100 mM of the amine donor were dissolved in 200 mM KPi buffer (200 mM, pH 8). The pH was then adjusted to 8.5-8.7 with phosphoric acid. Note: the final concentration of the reactants in the second step was half.

The yields for the produced amines between ketone **1a** and amine donors **d3-6** are summarized in the subsequent tables. The formation of the amines was verified via GC-MS. For the other ketones **2-4a**, no conversion to the amine products were detected.

Table S43. Conversion (%) for the biocatalytic cascade of **1a** combining OYE2 and aminating enzymes with the different amine donor (GC measurement with an achiral column).

amine donor	aminating enzyme	ERed	amine peak 1	amine peak 2	ERed	amine peak 1	amine peak 2
**d3**	Sp(S)-IRED	OYE2	n.d.	n.d.	YqjM-v1	n.d.	n.d.
**d3**	IRED1	OYE2	2	n.d.	YqjM-v1	1	0.2
**d3**	IRED5	OYE2	10	11	YqjM-v1	32	5
**d3**	IRED13	OYE2	n.d.	n.d.	YqjM-v1	2	5
**d3**	IRED14	OYE2	7	n.d.	YqjM-v1	6	22
**d3**	IRED15	OYE2	2	n.d.	YqjM-v1	2	0.3
**d3**	IRED20	OYE2	n.d.	n.d.	YqjM-v1	1	n.d.
**d3**	IRED21	OYE2	n.d.	n.d.	YqjM-v1	0.5	n.d.
**d3**	IRED22	OYE2	n.d.	n.d.	YqjM-v1	1	n.d.
**d3**	IRED30	OYE2	n.d.	n.d.	YqjM-v1	29	1
**d3**	IRED32	OYE2	17	11	YqjM-v1	14	4
**d3**	IRED7	OYE2	n.d.	n.d.	YqjM-v1	2	n.d.
**d3**	IRED10	OYE2	1	3	YqjM-v1	0.3	46
**d3**	IRED11	OYE2	n.d.	2	YqjM-v1	3	0.2
**d3**	IRED25	OYE2	6	n.d.	YqjM-v1	8	0.2
**d3**	AspRedAm	OYE2	n.d.	2	YqjM-v1	0.4	4

amine donor	aminating enzyme	ERed	amine peak 1	amine peak 2	ERED	amine peak 1	amine peak 2
**d4**	Sp(S)-IRED	OYE2	9	3	YqjM-v1	9	8
**d4**	IRED1	OYE2	46	4	YqjM-v1	8	17
**d4**	IRED5	OYE2	23	49	YqjM-v1	10	83
**d4**	IRED13	OYE2	43	19	YqjM-v1	8	27
**d4**	IRED14	OYE2	27	29	YqjM-v1	15	68
**d4**	IRED15	OYE2	62	1	YqjM-v1	24	26
**d4**	IRED20	OYE2	13	21	YqjM-v1	55	5
**d4**	IRED21	OYE2	8	28	YqjM-v1	21	24
**d4**	IRED22	OYE2	8	10	YqjM-v1	34	17
**d4**	IRED30	OYE2	8	70	YqjM-v1	16	24
**d4**	IRED32	OYE2	12	45	YqjM-v1	45	11
**d4**	IRED7	OYE2	25	1	YqjM-v1	4	18
**d4**	IRED10	OYE2	38	13	YqjM-v1	2	79
**d4**	IRED11	OYE2	8	71	YqjM-v1	9	85
**d4**	IRED25	OYE2	50	1	YqjM-v1	8	35
**d4**	AspRedAm	OYE2	0.1	84	YqjM-v1	25	56

amine donor	aminating enzyme	ERed	amine peak 1	amine peak 2	ERed	amine peak 1	amine peak 2
**d5**	Sp(S)-IRED	OYE2	11	n.d.	YqjM-v1	4	6
**d5**	IRED1	OYE2	45	1	YqjM-v1	6	38
**d5**	IRED5	OYE2	53	10	YqjM-v1	37	41
**d5**	IRED13	OYE2	9	4	YqjM-v1	7	21
**d5**	IRED14	OYE2	13	38	YqjM-v1	15	64
**d5**	IRED15	OYE2	58	0.2	YqjM-v1	13	32
**d5**	IRED20	OYE2	2	18	YqjM-v1	35	3
**d5**	IRED21	OYE2	7	22	YqjM-v1	13	16
**d5**	IRED22	OYE2	2	5	YqjM-v1	12	3
**d5**	IRED30	OYE2	7	67	YqjM-v1	69	7
**d5**	IRED32	OYE2	3	49	YqjM-v1	17	4
**d5**	IRED7	OYE2	16	1	YqjM-v1	8	11
**d5**	IRED10	OYE2	43	10	YqjM-v1	2	64
**d5**	IRED11	OYE2	24	46	YqjM-v1	20	62

**d5**	IRED25	OYE2	52	1	YqjM-v1	5	44
**d5**	AspRedAm	OYE2	0.2	92	YqjM-v1	27	67

amine donor	aminating enzyme	ERed	amine peak 1	amine peak 2	ERed	amine peak 1	amine peak 2
**d6**	Sp(S)-IRED	OYE2	22	0.2	YqjM-v1	4	5
**d6**	IRED1	OYE2	58	2	YqjM-v1	10	53
**d6**	IRED5	OYE2	32	6	YqjM-v1	27	20
**d6**	IRED13	OYE2	13	5	YqjM-v1	6	24
**d6**	IRED14	OYE2	10	27	YqjM-v1	12	51
**d6**	IRED15	OYE2	52	1	YqjM-v1	6	20
**d6**	IRED20	OYE2	2	16	YqjM-v1	29	3
**d6**	IRED21	OYE2	4	11	YqjM-v1	7	16
**d6**	IRED22	OYE2	2	5	YqjM-v1	13	2
**d6**	IRED30	OYE2	10	61	YqjM-v1	51	6
**d6**	IRED32	OYE2	10	25	YqjM-v1	9	3
**d6**	IRED7	OYE2	12	0.4	YqjM-v1	6	13
**d6**	IRED10	OYE2	46	19	YqjM-v1	6	89
**d6**	IRED11	OYE2	14	43	YqjM-v1	15	50
**d6**	IRED25	OYE2	31	0.4	YqjM-v1	4	20
**d6**	AspRedAm	OYE2	1	81	YqjM-v1	9	79

Representative GC-MS chromatograms for substrate 1 with the different amine donor.

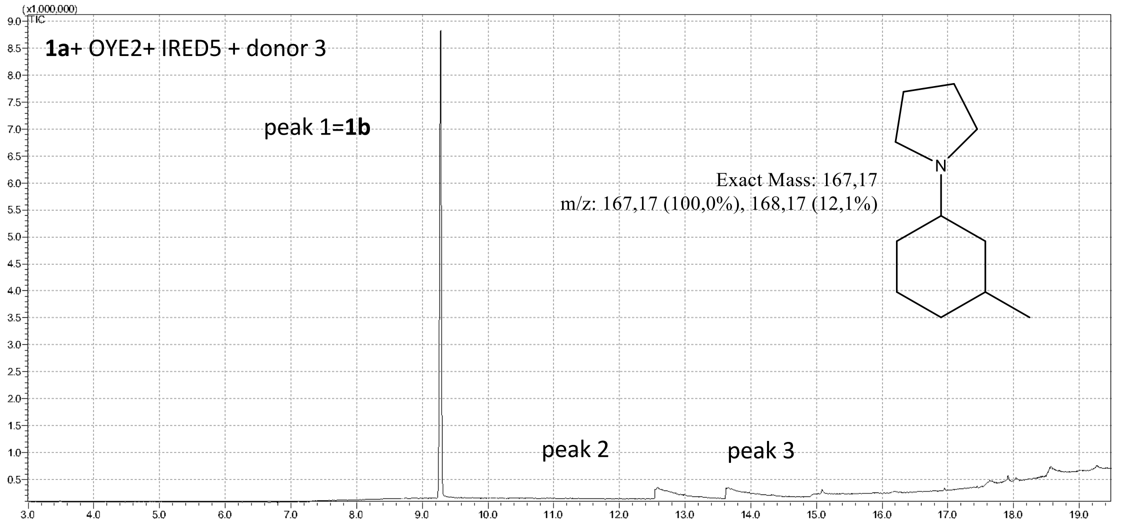

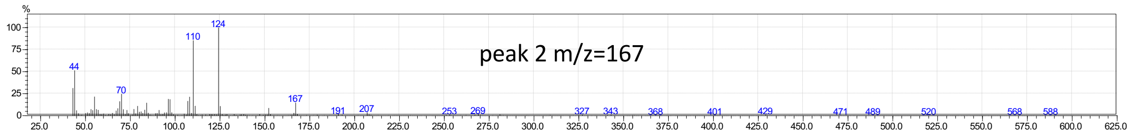

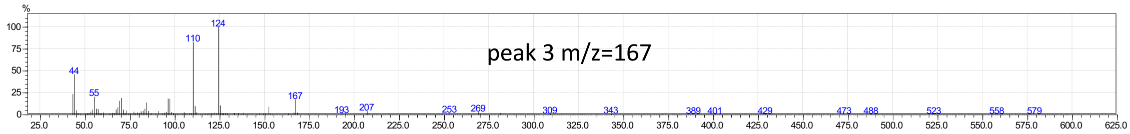

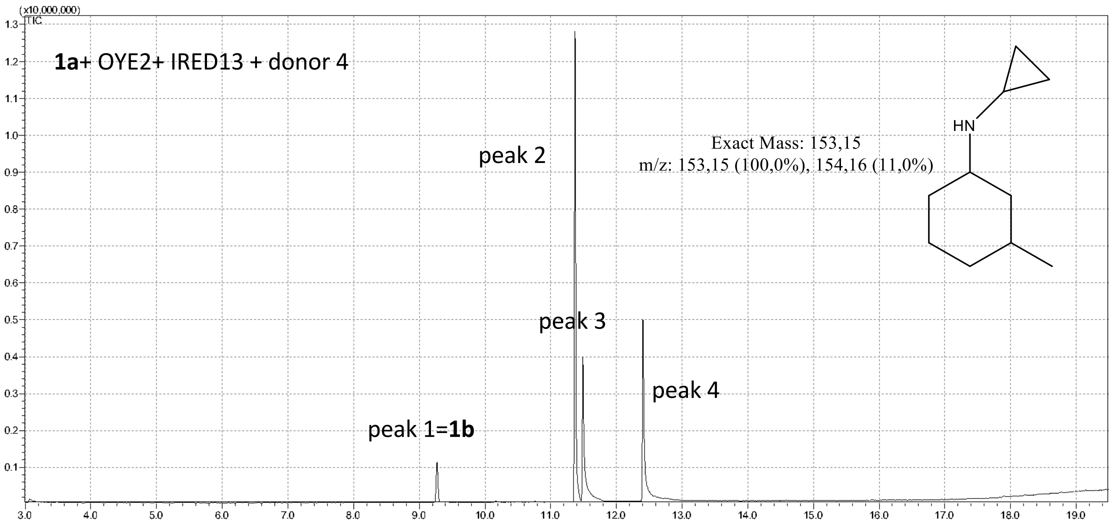

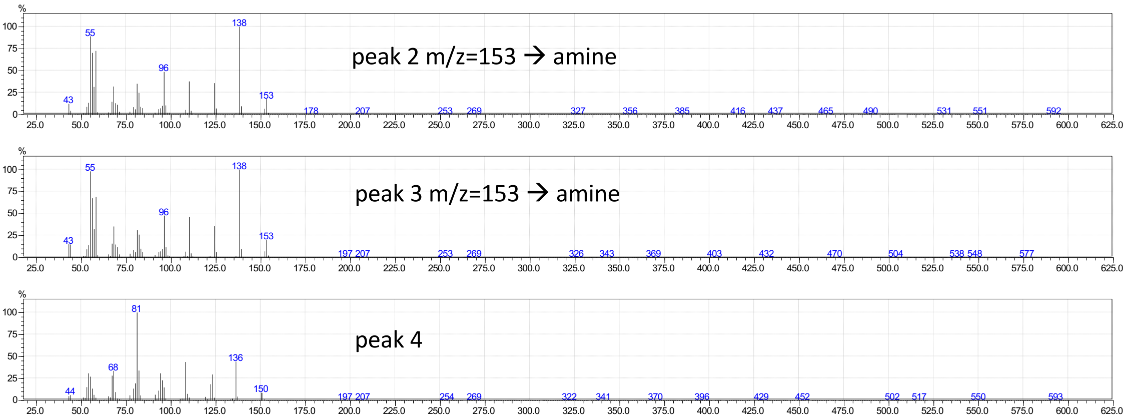

Peak 4 is the MS of the imine that is in equilibrium with ketone **1a** in the reaction mixture.

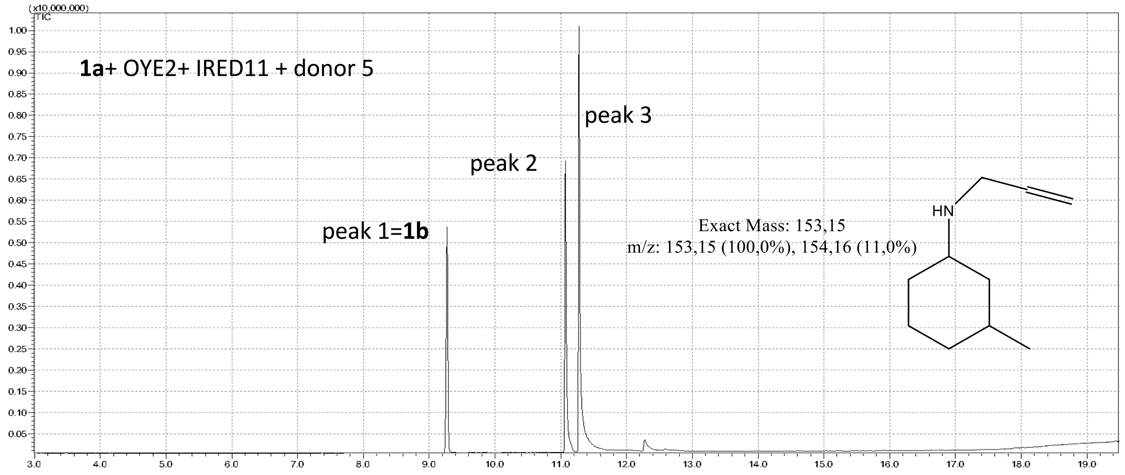
1a+ OYE2+ IRED11 + donor 5
peak 1=1b
peak 2
peak 3
Exact Mass: 153,15
m/z: 153,15 (100,0%), 154,16 (11,0%)
HN

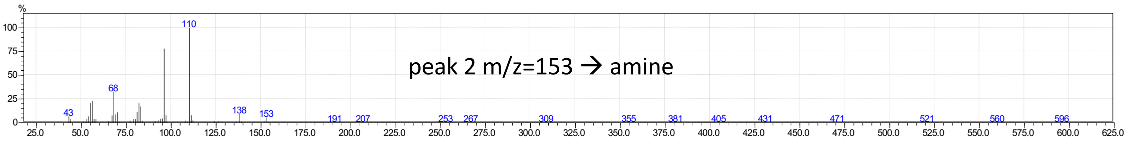
peak 2 m/z=153 → amine

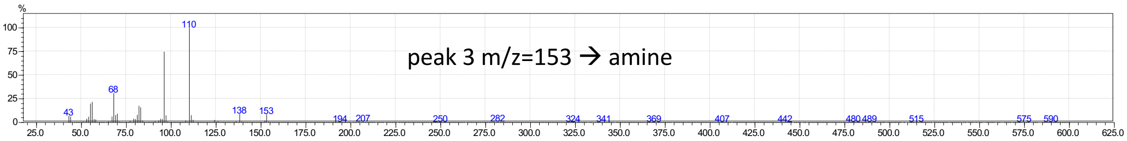
peak 3 m/z=153 → amine

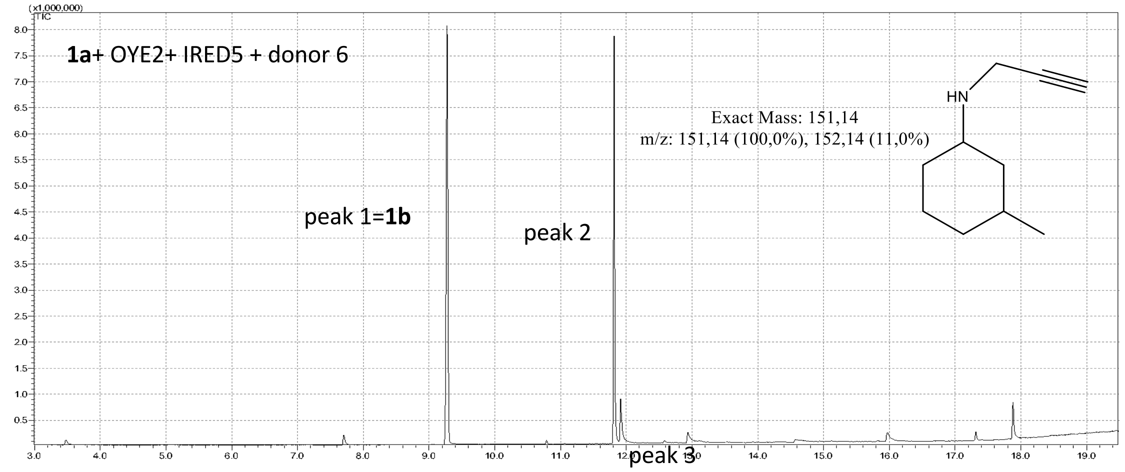
1a+ OYE2+ IRED5 + donor 6
peak 1=1b
peak 2
peak 3
Exact Mass: 151,14
m/z: 151,14 (100,0%), 152,14 (11,0%)
HN

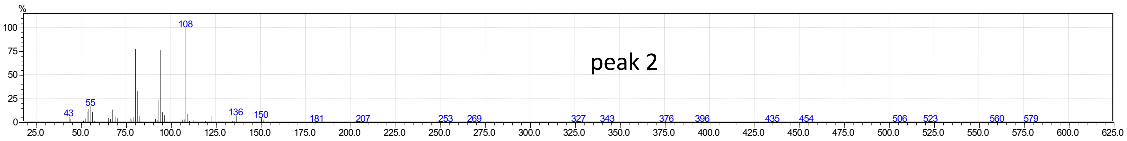
peak 2

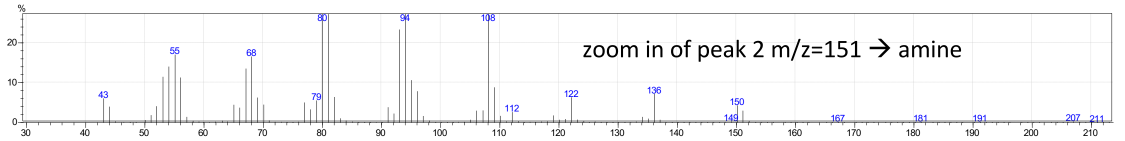
zoom in of peak 2 m/z=151 → amine

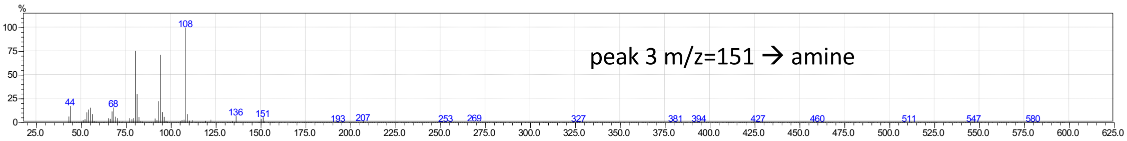
peak 3 m/z=151 → amine

11. Summary of results as depicted in Figures 1 and 2, main manuscript.

Table S44. Summary amination (%) of **1-4b** using IREDs, AspRedAm and AmDHs in ammonium formate/ammonia buffer (achiral GC measurement).

entry	substrate	IRed type	total conversion[a] **1-4b** (%)	conversion ratio of diastereomers (%,%)
1	*rac*-**1b**	IRED-1	30	26:4[b]
2	*rac*-**1b**	IRED-5	56	28:28[b]
3	*rac*-**1b**	IRED-10	94	94:0[b]
4	*rac*-**1b**	IRED-13	22	20:2[b]
5	*rac*-**1b**	IRED-15	28	27:1[b]
6	*rac*-**1b**	IRED-20	99	95:4[b]
7	*rac*-**1b**	IRED-22	73	68:5[b]
8	*rac*-**1b**	IRED-25	30	30:0[b]
9	*rac*-**1b**	LE-AmDH-v1	58	29:29[b]
10	(*R*)-**1b**	IRED-1	8	7:1[c]
11	(*R*)-**1b**	IRED-5	30	22:8[c]
12	(*R*)-**1b**	IRED-10	>99	>99.5:<0.5[c]
13	(*R*)-**1b**	IRED-13	37	36:1[c]
14	(*R*)-**1b**	IRED-15	38	37:1[c]
15	(*R*)-**1b**	IRED-20	73	70:3[c]
16	(*R*)-**1b**	IRED-22	63	52:11[c]
17	(*R*)-**1b**	IRED-25	35	35:<1[c]
18	*rac*-**2b**	IRED-10	41	16:25[d]
19	*rac*-**2b**	IRED-13	22	16:6[d]
20	*rac*-**2b**	IRED-14	58	38:20[d]
21	*rac*-**2b**	IRED-15	92	69:23[d]
22	*rac*-**2b**	IRED-20	>99	82:18[d]
23	rac-**2b**	IRED-22	>99	68:32[d]
24	*rac*-**3b**	IRED-20	38	n.d.
25	*rac*-**3b**	IRED-22	25	n.d.
26	*rac*-**4b**	IRED-20	31	21:10[e]
27	*rac*-**4b**	Ch1-AmDH	58	29:29[e]

[a] measured by achiral GC (DB1701, 30m, Agilent); [b] Absolute configuration of each pair of diastereomers: first pair (1*S*, 3*R*)-**1c** + (1*R*, 3*S*)-**1c**; second pair (1*R*, 3*R*)-**1c** + (1*S*, 3*S*)-**1c**; [c] Absolute configuration of each diastereomers: first diastereomer (1*S*, 3*R*)-**1c**; second diastereomer (1*R*, 3*R*)-**1c**; [d] Absolute configuration of each pair of diastereomers: first pair (1*S*, 2*S*)-**2c** + (1*R*, 2*R*)-**2c**; second pair (1*S*, 2*R*)-**2c** + (1*R*, 2*S*)-**2c**; [d] Absolute configuration of each pair of diastereomers: first pair (2*S*, 3*S*)-**4c** + (2*R*, 3*R*)-**4c**; second pair (2*S*, 3*R*)-**4c** + (2*R*, 3*S*)-**4c**;

Table S45. Summary amination (%) of **1-4b** using IREDs and AspRedAm in methylamine/methylammonum formate buffer (achiral GC measurement).

entry	substrate	IRed type	total conversion[a] **1-4b** (%)	conversion ratio of diastereomers (%,%)
1	*rac*-**1b**	IRED-5	>99	36:64[b]
2	*rac*-**1b**	IRED-11	99	30:69[b]
3	*rac*-**1b**	IRED-13	99	19:80[b]
4	*rac*-**1b**	IRED-14	84	15:69[b]
5	*rac*-**1b**	IRED-15	>99	35:65[b]
6	*rac*-**1b**	IRED-20	>99	21:79[b]
7	*rac*-**1b**	IRED-22	>99	31:69[b]
8	*rac*-**1b**	IRED-25	>99	2:98[b]
9	*rac*-**1b**	IRED-30	>99	44:56[b]
10	(*R*)-**1b**	IRED-5	>99	18:82[c]
11	(*R*)-**1b**	IRED-10	>99	2:98[c]
12	(*R*)-**1b**	IRED-11	>99	<0.5:>99.5[c]
13	(*R*)-**1b**	IRED-13	>99	6:94[c]
14	(*R*)-**1b**	IRED-14	98	7:91[c]
15	(*R*)-**1b**	IRED-15	>99	5:95[c]
16	(*R*)-**1b**	IRED-20	>99	31:69[c]
17	(*R*)-**1b**	IRED-22	>99	46:54[c]
18	(*R*)-**1b**	IRED-30	>99	41:59[c]
19	(*R*)-**1b**	AspRedAm	73	18:55[c]
20	*rac*-**2b**	IRED-5	80	26:54[d]
21	*rac*-**2b**	IRED-10	40	5:35[d]
22	*rac*-**2b**	IRED-11	97	36:61[d]
23	*rac*-**2b**	IRED-13	81	60:21[d]
24	*rac*-**2b**	IRED-14	64	56:38[d]
25	*rac*-**2b**	IRED-15	98	19:79[d]
26	*rac*-**2b**	IRED-20	>99	38:62[d]
27	*rac*-**2b**	IRED-22	>99	30:70[d]
28	*rac*-**2b**	IRED-30	>99	27:73[d]
29	*rac*-**2b**	AspRedAm	58	13:45[d]
30	*rac*-**3b**	IRED-5	97	n.d.
31	*rac*-**3b**	IRED-10	>20	n.d.
32	*rac*-**3b**	IRED-11	>99	n.d.
33	*rac*-**3b**	IRED-13	51	n.d.
34	*rac*-**3b**	IRED-14	44	n.d.
35	*rac*-**3b**	IRED-15	>99	n.d.
36	*rac*-**3b**	IRED-20	>99	n.d.
37	*rac*-**3b**	IRED-22	>99	n.d.
38	*rac*-**3b**	IRED-30	98	n.d.
39	*rac*-**3b**	AspRedAm	78	n.d.
40	*rac*-**4b**	IRED-5	6	5:1[e]
41	*rac*-**4b**	IRED-13	12	5:7[e]
42	*rac*-**4b**	IRED-14	26	5:21[e]
43	*rac*-**4b**	IRED-15	4	3:1[e]
44	*rac*-**4b**	IRED-20	51	22:29[e]
45	*rac*-**4b**	IRED-22	11	5:6[e]
46	*rac*-**4b**	IRED-30	11	4:7[e]
47	*rac*-**4b**	IRED-32	22	14:8[e]

[a] measured by achiral GC (DB1701, 30m, Agilent); [b] Absolute configuration of each pair of diastereomers: first pair (1*R*, 3*R*)-**1d** + (1*S*, 3*S*)-**1d**; second pair (1*S*, 3*R*)-**1d** + (1*R*, 3*S*)-**1d**; [c] Absolute configuration of each diastereomers: first diastereomer (1*R*, 3*R*)-**1d**; second diastereomer (1*S*, 3*R*)-**1d**; [d] Absolute configuration of each pair of diastereomers: first pair (1*R*, 2*S*)-**2d** + (1*S*, 2*R*)-**2d**; second pair (1*R*, 2*R*)-**2d** + (1*S*, 2*S*)-**2d**; [e] Absolute configuration of each pair of diastereomers: first pair (2*S*, 3*S*)-**4d** + (2*R*, 3*R*)-**4d**; second pair (2*S*, 3*R*)-**4d** + (2*R*, 3*S*)-**4d**;

12. Synthesis of reference compounds

12.1. Substrates 1a-d

*12.1.1. References for **1c** using ωTAs on analytical scale*

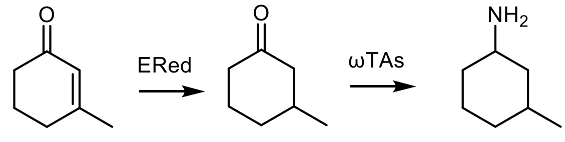

1a **1b** **1c**

Table S46. Conversion (%) for substrate **1a** or (*R*)-**1b** using ωTAs or an ERed in combination with ωTAs for obtaining the four stereoisomers of the primary amine **1c**. (GC measurement with an achiral column).

substrate	ERed	ωTA	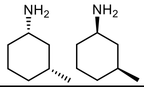	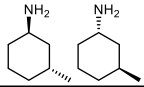	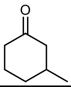	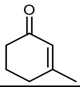	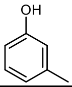
			(1*S*,3*R*)-**1c** + (1*R*,3*S*)-**1c**	(1*R*,3*R*)-**1c** + (1*S*,3*S*)-**1c**	**1b**	**1a**	
**1a**	OYE2	-			**86**	8	6
**1b**	-	Vf-ωTA (S)	60	27	13	n.d.	n.d.
**1b**	-	As(R)-ωTA (R)	22	38	40	n.d.	n.d.
**1b**	-	Cv-ωTA (S)	17	64	19	n.d.	n.d.
(*R*)-**1b**	-	Vf-ωTA (S)	37	34	29	n.d.	n.d.
(*R*)-**1b**	-	As(R)-ωTA (R)	n.d.	80	20	n.d.	n.d.
(*R*)-**1b**	-	Cv-ωTA (S)	32	53	15	n.d.	n.d.
**1a**	OYE2	Vf-ωTA (S)	54	45	0.9	0.4	n.d.
**1a**	OYE2	As(R)-ωTA (R)	46	1	51	2	n.d.
**1a**	OYE2	Cv-ωTA (S)	1	74	23	2	n.d.

Table S47. Diastereomeric and enantiomeric composition (%) for **1c** (GC measurement with a chiral column) after derivatization to the acetamide.

substrate	ERed	ωTA	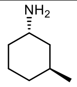 (1*S*,3*S*)-**1c**	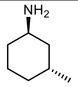 (1*R*,3*R*)-**1c**	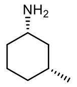 (1*S*,3*R*)-**1c**	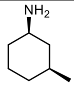 (1*R*,3*S*)-**1c**
**1b**	-	Vf-ωTA (S)	6	23	22	49
**1b**	-	As(R)-ωTA (R)	n.d.	61	n.d.	39
**1b**	-	Cv-ωTA (S)	46	32	21	1
(*R*)-**1b**	-	Vf-ωTA (S)	not possible	46	54	not possible
(*R*)-**1b**	-	As(R)-ωTA (R)	not possible	>99	n.d.	not possible
(*R*)-**1b**	-	Cv-ωTA (S)	not possible	60	40	not possible
**1a**	OYE2	Vf-ωTA (S)	42	not possible	not possible	58
**1a**	OYE2	As(R)-ωTA (R)	n.d.	not possible	not possible	>99
**1a**	OYE2	Cv-ωTA (S)	98	not possible	not possible	2

note: The OYE2 blank reaction yields an *e.r.* >99.8:<0.2 ((*S*)-enantiomer)

According to the reaction performed with As(R)-ωTA, the configuration for the amine center could be identified. The peak annotation is also according with the enzymes' selectivity for **1b** as reported in the literature. [29] [3]

GC-FID chromatograms (chiral GC) for the separation of the **1c** isomers from ωTA reactions:

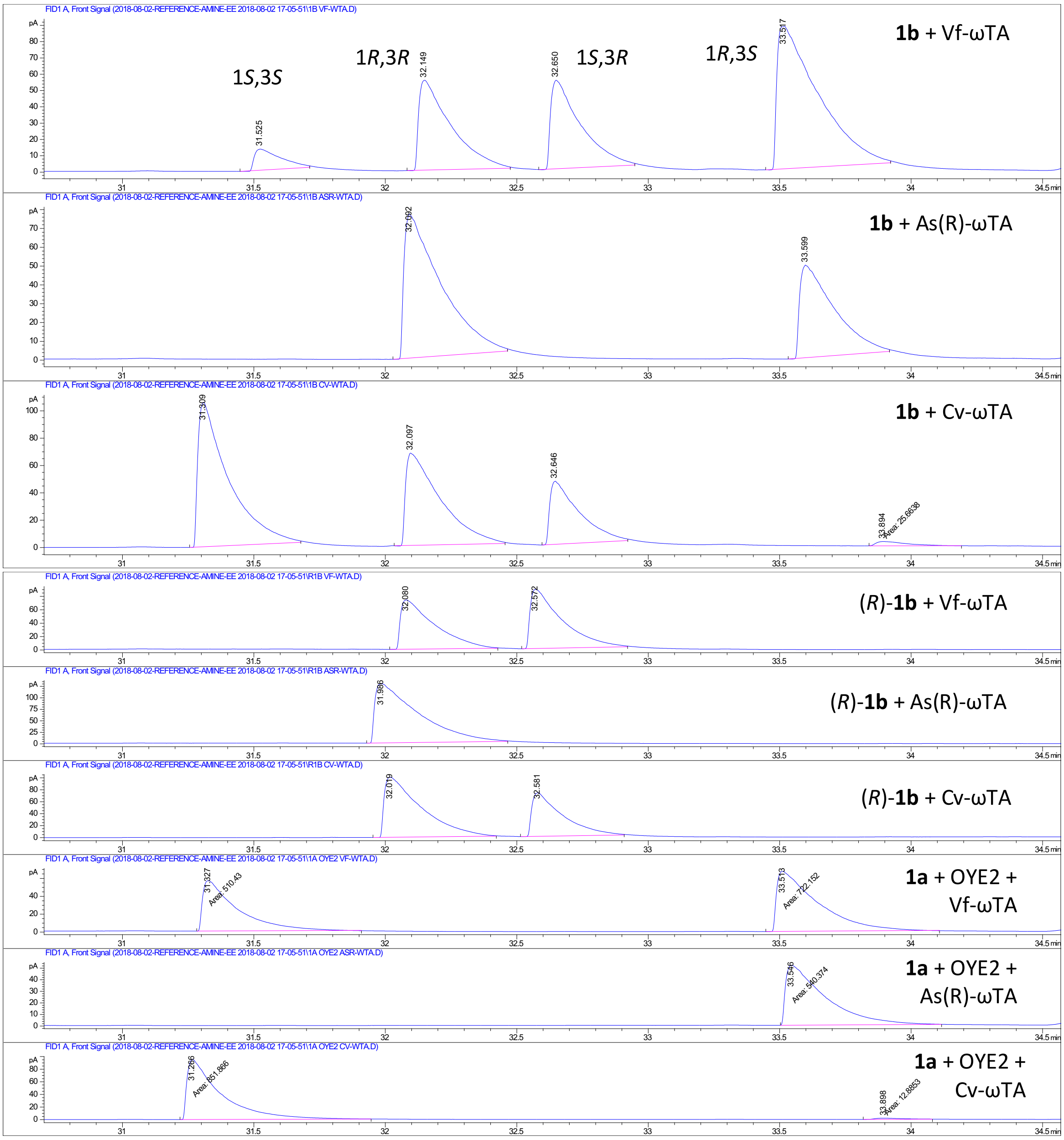

12.1.2. Chemo-enzymatic strategy for the assignment of the absolute configuration of α-chiral secondary amines

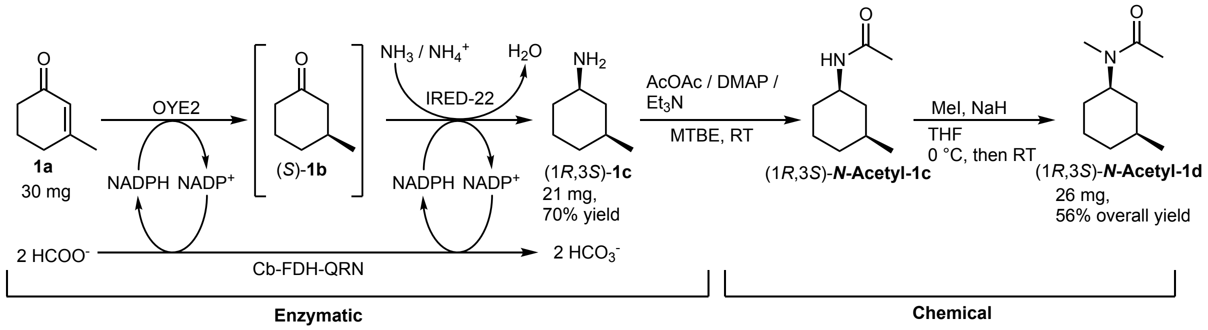

Figure S6. Chemo-enzymatic strategy for the assignment of the absolute configuration of α-chiral secondary amines, exemplified for the case of **1d**. Stereoisomers of *N*-acetylated **1d** as the final product can be separated by GC with a chiral column. The method can be extended beyond *N*-methyl secondary amines by using different alkyl iodide reagents in the last step.

For example, (1*R*, 3*S*)-**1c** (97:3 d.r., 99.5:0.5 e.r.) was synthesized in 96% conversion and 70% analytical yield (measured by GC, ca. 21 mg) from **1a** (30 mg) by alkene reduction catalyzed by OYE2 and subsequent reductive amination catalyzed by IRED-22. Next, (1*R*, 3*S*)-**1c** was subjected to subsequent chemical acetylation using acetic anhydride, DMAP and Et_3N in MTBE, and *N*-methylation using methyl-iodide with NaH in THF to afford *N*-acetylated (1*R*, 3*S*)-**1d** (26 mg, 56% overall yield from **1a**), which served as reference standard for chiral GC determination. We followed the same chemo-enzymatic strategy for the synthesis of the other *N*-acetylated stereoisomers of **1d** by starting from commercially available (*R*)-**1b** (for details and experimental procedures, see SI section 12.1.3). Racemic (non-acetylated) **1d** was also synthesized as analytical standard from commercially available *rac*-**1c** via reaction with ethyl-chloroformate and followed by reduction with $LiAlH_4$ (see SI section 12.1.2).

12.1.3. Chemical synthesis of *rac*-*1d*

Step 1: carbamate formation: The synthesis of the carbamate compound **6** was performed by reacting the amine **1c** (4.505 mmol, 510 mg, 0.6 mL) with ethylchloroformate **5** (11.263 mmol, 1.219 g, 1.1 mL) in the presence of pyridine (2.934 g, 3 mL) as catalyst in a mixture of EtOH/H_2O (22.5/12 mL v/v). The ratio between pyridine/EtOH/H_2O was 8:32:60. The reaction was complete after stirring overnight at room temperature leading to the formation of compound **6** as a mixture of diastereomers according to GC-MS analysis. The isolated product was obtained in 73% yield (610 mg) as a clear yellow liquid and used in the next step without any further purification.

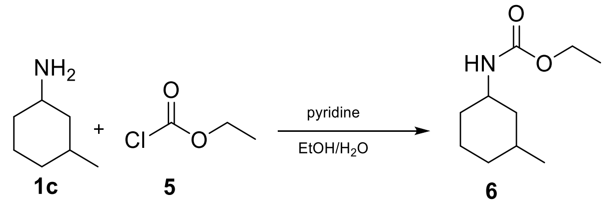

Step 2: carbamate reduction: The crude product **6** (2.699 mmol, 500 mg) was directly used in the reduction to the corresponding methylated amine **1d** by $LiAlH_4$ (2 M stock in THF; 6.747 mmol, 256e mg, 2.5 eq., 6.8 mL) in THF (18 mL). The reaction was complete after 3.5 h leading to the formation of compound **1d** as a mixture of diastereomers according to GC-MS analysis. The isolated product was obtained in 45% yield (153.4 mg) as a yellow liquid. The final product **1d** was also analyzed by ^{1}H-NMR (a complex spectrum due to the presence of stereoisomers).

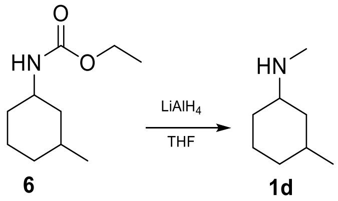

GC-MS analysis of isolated carbamate **6** (achiral column Agilent, DB-1701, 30m x 250 µm x 0.25 µm):

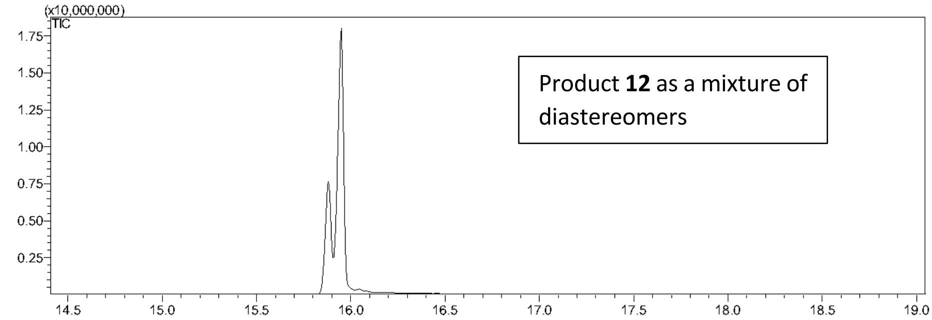

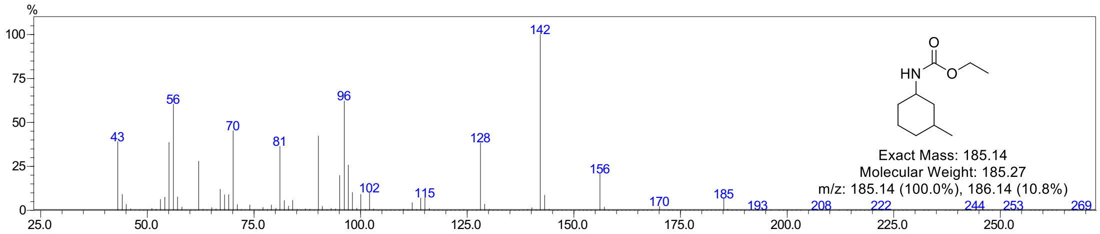

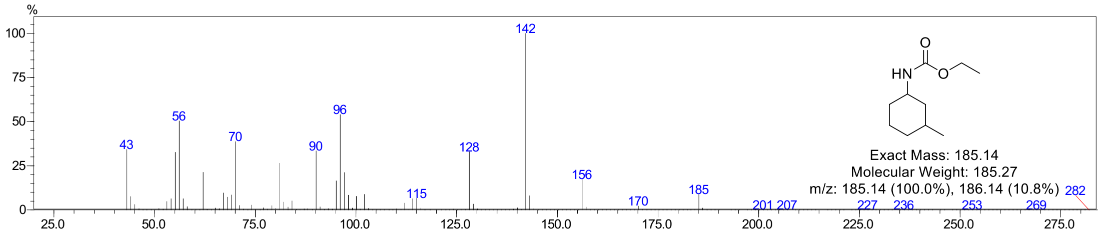

GC-MS analysis isolated compound **1d** (achiral column Agilent, DB-1701, 30m x 250 µm x 0.25 µm)::

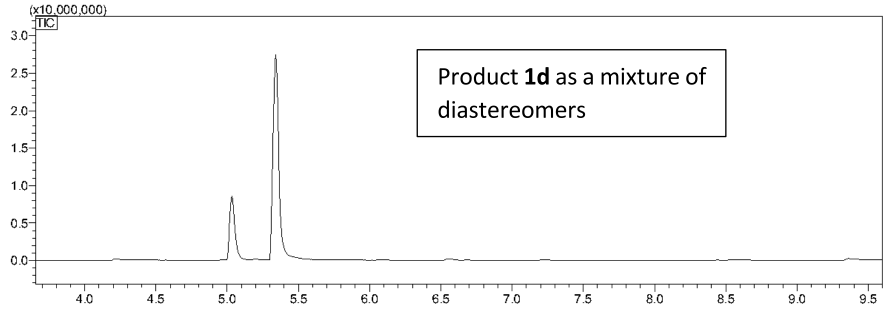

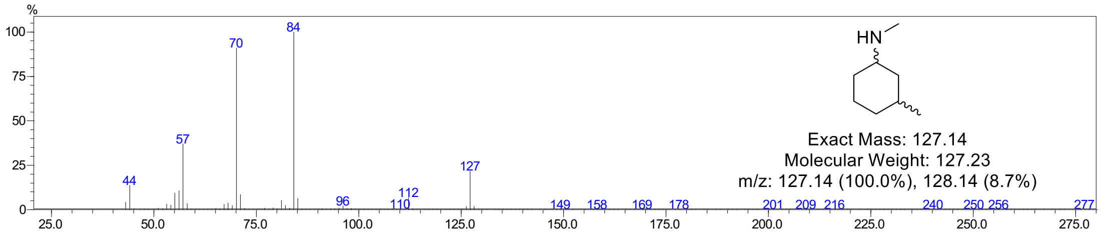

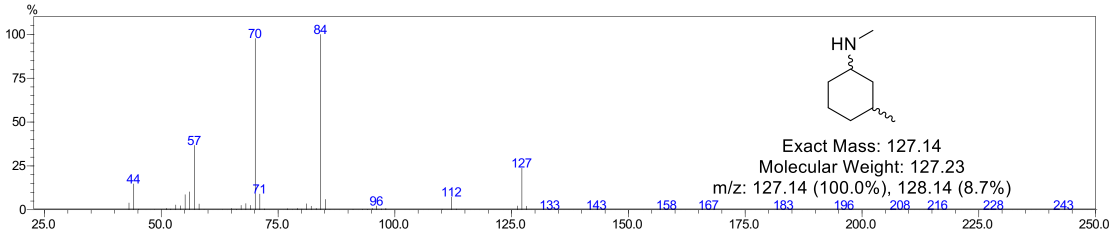

^{1}H-NMR isolated compound **1d** (solvent $CDCl_3$):

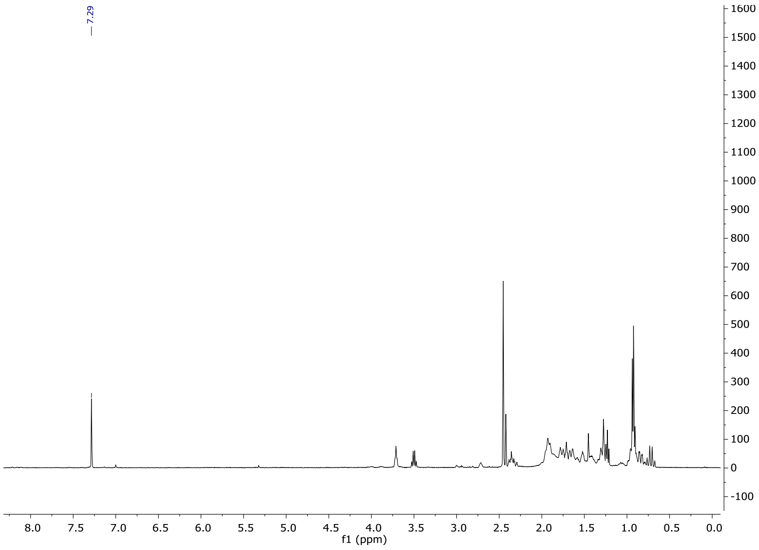

12.1.4. *References for **1d** – chemo-enzymatic synthesis*

To identify the configurations of the primary amines **1d**, first the enantiopure free amines **1c** were prepared on semi-preparative scale (30-50 mg substrate), followed by subsequent chemical manipulation.

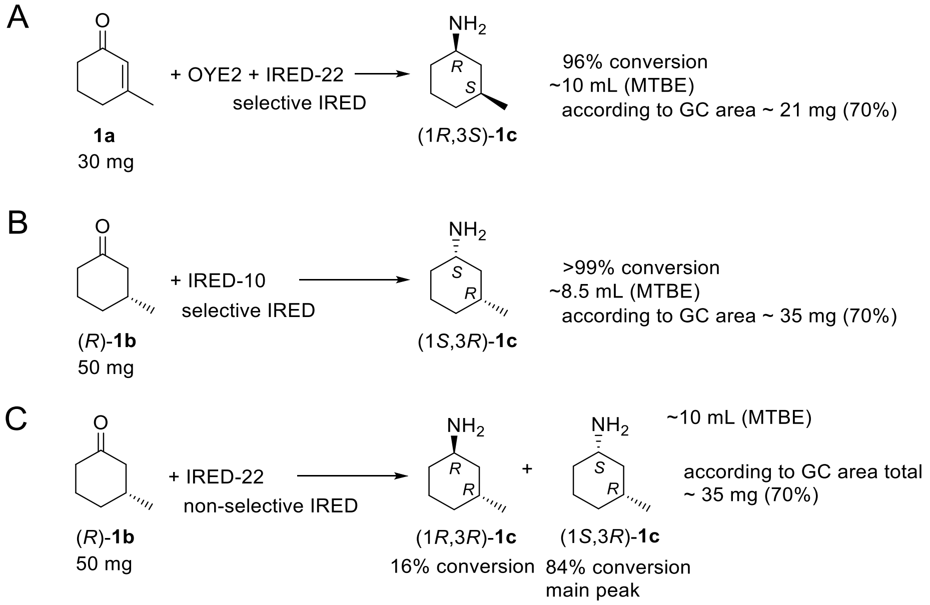

(1) Biocatalytic reactions

Reaction (A). In a 100 mL Erlenmeyer flask, a total volume of 27 mL consisted of: buffer (ammonium formate/ammonia, 24.8 mL, 1 M, pH 8), OYE2 (13 µM, 546 µL of 643 µM stock), IRED-22 (30 µM, 832 µL of 974 µM stock), FDH-QRN (10 µM, 227 µL of 974 µM stock), $NADP^+$ (0.5 mM, 540 µL of 25 mM stock) and substrate **1a** (30 mg, 10 mM, 30.9 µL, 110.15 g/mol, 0.971 mg/mL).

Reaction (B). In a 100 mL Erlenmeyer flask, a total volume of 41 mL consisted of: buffer (ammonium formate/ammonia, 39.5 mL, 1 M, pH 8), IRED-10 (20 µM, 781 µL of 1050 µM stock), FDH-QRN (5 µM, 210 µL of 974 µM stock), $NADP^+$ (0.25 mM, 410 µL of 25 mM stock) and substrate (*R*)-**1b** (50 mg, 10 mM, 55 µL, 110.15 g/mol).

Reaction (C). In a 100 mL Erlenmeyer flask, a total volume of 41 mL consisted of: buffer (ammonium formate/ammonia, 38.6 mL, 1 M, pH 8), IRED-22 (40 µM, 1684 µL of 974 µM stock), FDH-QRN (5 µM, 210 µL of 974 µM stock), $NADP^+$ (0.25 mM, 410 µL of 25 mM stock) and substrate (*R*)-**1b** (50 mg, 10 mM, 55 µL, 110.15 g/mol).

The reactions were run at 30 °C for 24 h. A 50 µL sample was extracted after basifying and the conversion measured by GC using an achiral column. The samples were derivatized and measured by GC using a chiral column..

Table S48. Conversion (%) for the semi-preparative reactions (GC measurement with an achiral column) on an analytical aliquot of the reaction mixture

	**1c** (%)		**1b** (%)	**1a** (%)
(A)	96	n.d.	4	<1
(B)	>99	n.d.	n.d.	n.d.
(C)	84	16	n.d.	n.d.

Table S49. Diastereomeric and enantiomeric composition (%) for the semi-preparative reactions (GC measurement with a chiral column) after derivatization to the acetamide of an analytical aliquot of the reaction mixture. Peak annotation in agreement with section 12.1.1.

	(1*S*,3*S*)-**1c** (%)	(1*R*,3*R*)-**1c** (%)	(1*S*,3*R*)-**1c** (%)	(1*R*,3*S*)-**1c** (%)
(A)	1.1	2.0	0.5	96.4
(B)	n.d.	1.5	98.2	0.3
(C)	n.d.	17.8	81.9	0.3

Chiral GC chromatograms of the biocatalytic reactions run on semi-preparative scale (ammonium formate/ammonia buffer.

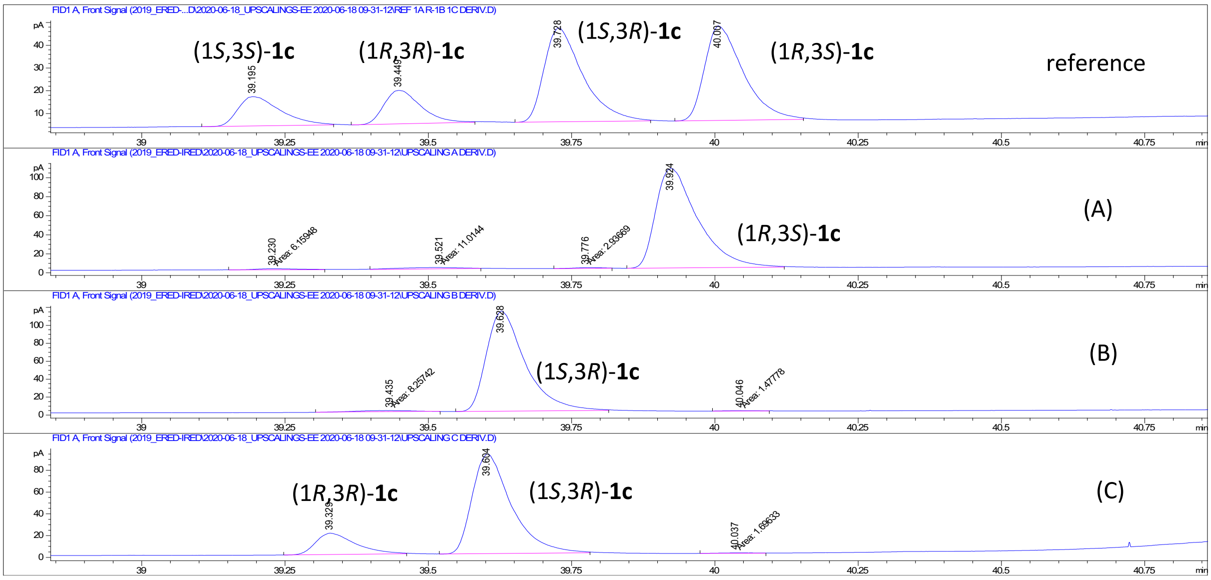

Each of the whole reaction mixtures was basified with KOH (10 M, 3 mL for A and 4 mL for B and C), transferred in 2 x 50 mL falcon tubes, and the organic compounds were extracted with MTBE (2 x 10 mL per falcon tube for reaction A and 2 x 15 mL per falcon tube for reaction B and C). Centrifugation between the extraction steps was done for 10 min (4500 rpm). The organic phases were dried over $MgSO_4$ and collected in glass vials.

Due to the volatility of the amine products **1c**, the MTBE was not evaporated with rotavapor. In contrast, the solvent was left to partly evaporate under the fume hood until reaching a volume of ca. 10 mL. However, this step might lead to also loss of some **1c** due to partial concomitant evaporation with MTBE; therefore, this step must be done with great care. The **1c** product in the remaining MTBE was stored at -20 °C until the next chemical synthesis steps.

<u>(2) Acetylation of optically active 1c from step (1) (reactions A, B and C) and of commercially available *rac*-1c (reaction D)</u>

Reaction A: The acetylation was carried out directly in the glass vials. To the dissolved compound (1*R*,3*S*)-**1c** (ca. 21 mg in ca. 10 mL MTBE, 0.186 mmol, 113.20 g/mol, ca. 19 mM) obtained from the biocatalytic reaction, the acetylation reagents have been added in following order: DMAP (10 mol%, 2.4 mg, 122.17 g/mol, 0.019 mmol), acetic anhydride (1 mL, 15.6 mmol, 102.1 g/mol) and triethylamine (ca. 5 eq., 130 µL, 0.93 mmol, 101.19 g/mol). The mixture was stirred at room temperature for 3 h. After that, a small sample was taken, diluted to ~4-5 mg/mL with MTBE and the progress of the reaction was checked via GC-MS (chromatograms and MS are reported in the next pages). **Work-up:** The reaction was treated with aqueous HCl (1 N, 10 mL) and the obtained mixture was stirred vigorously for ca. 15 min at RT. After the separation of the two phases, the organic phase was then treated with a saturated solution of K_2CO_3 (10 mL) and stirred for ca. 15 min at RT. After separation of the two phases, the organic phase was dried over anhydrous $MgSO_4$ and the solvent was evaporated under reduced pressure. A white solid was obtained (53.2 mg) and was directly used for the next step (3).

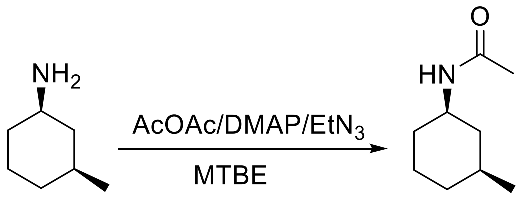

(1*R*,3*S*)-**1c** (1*R*,3*S*)-*N*-acetyl-**1c**

Reaction B: The acetylation was carried out in the same way as for reaction A using following reagents: (1*S*,3*R*)-**1c** (ca. 35 mg in ca. 8.5 mL MTBE, 0.309 mmol, 113.20 g/mol, ca. 36 mM), DMAP (10 mol%, 3.8 mg, 122.17 g/mol, 0.031 mmol), acetic anhydride (1 mL, 15.6 mmol, 102.1 g/mol) and triethylamine (ca. 5 eq., 215 µL, 1.55 mmol, 101.19 g/mol). After work-up, a white solid was obtained (49.4 mg) and was directly used for the next step (3).

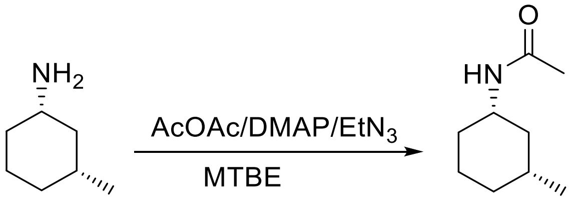

(1*S*,3*R*)-**1c** (1*S*,3*R*)-*N*-acetyl-**1c**

Reaction C: The acetylation was carried out in the same way as for reaction A using following reagents: (1*R*,3*R*)-**1c**+(1*S*,3*R*)-**1c** (ca. 35 mg in ca. 10 mL MTBE, 0.309 mmol, 113.20 g/mol, ca. 31 mM), DMAP (10 mol%, 3.8 mg, 122.17 g/mol, 0.031 mmol), acetic anhydride (1 mL, 15.6 mmol, 102.1 g/mol) and triethylamine (ca. 5 eq., 215 µL, 1.55 mmol, 101.19 g/mol). After work-up, a white solid was obtained (67.3 mg) and was directly used for the next step (3).

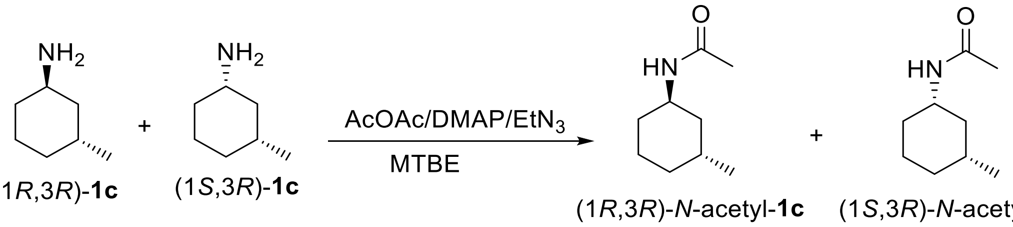

(1*R*,3*R*)-**1c** (1*S*,3*R*)-**1c** (1*R*,3*R*)-*N*-acetyl-**1c** (1*S*,3*R*)-*N*-acetyl-**1c**

Reaction D: The acetylation was carried out in the same way as for reaction A using following reagents: *rac*-**1c** (155 mg, 1.37 mmol, 113.20 g/mol), DMAP (10 mol%, 16.5 mg, 122.17 g/mol, 0.135 mmol), acetic anhydride (1.5 mL, 15.9 mmol, 102.1 g/mol) and triethylamine (ca. 5 eq., 920 µL, 6.62 mmol, 101.19 g/mol) in 10 mL of MTBE. After work-up, a white solid was obtained (173 mg, 1.12 mmol, 81% isolated yield) and was directly used for the next step (3).

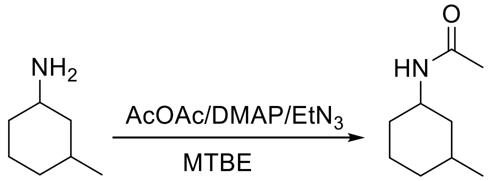

rac-**1c** *rac*-*N*-acetyl-**1c**

GC-MS chromatogram of Reaction A (monitor of progress of reaction):

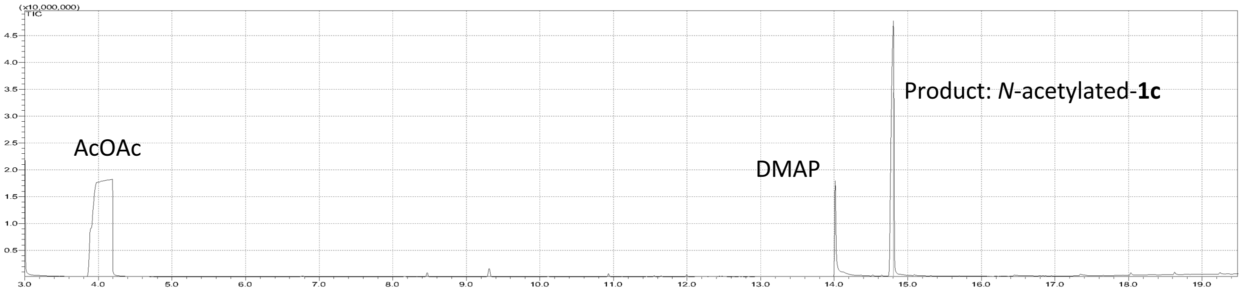

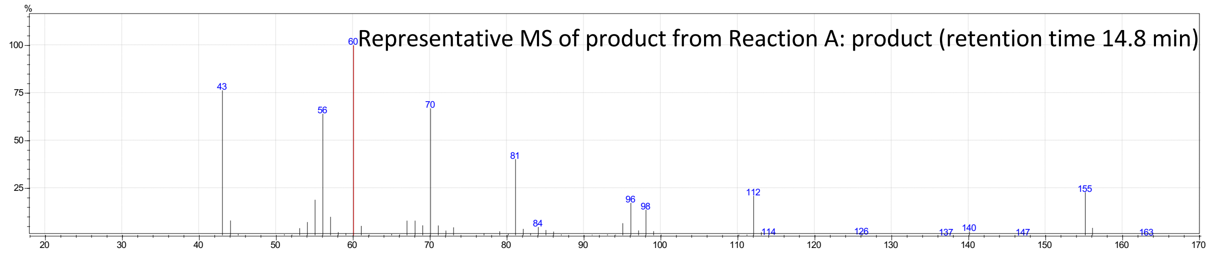

GC-MS chromatogram of Reaction B (monitor of progress of reaction):

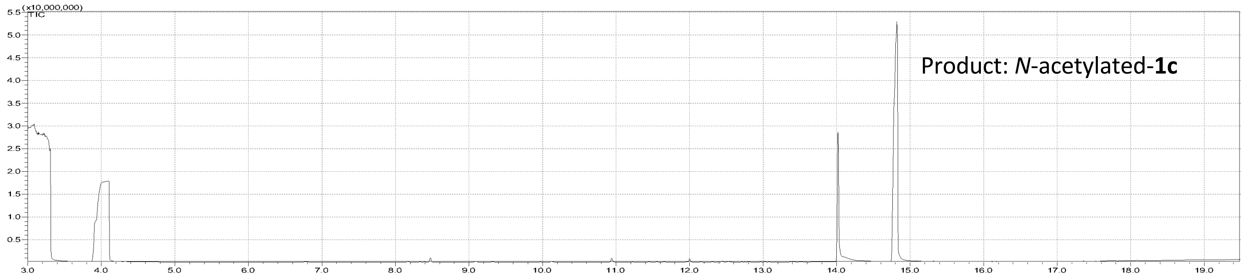

GC-MS chromatogram of Reaction C (monitor of progress of reaction):

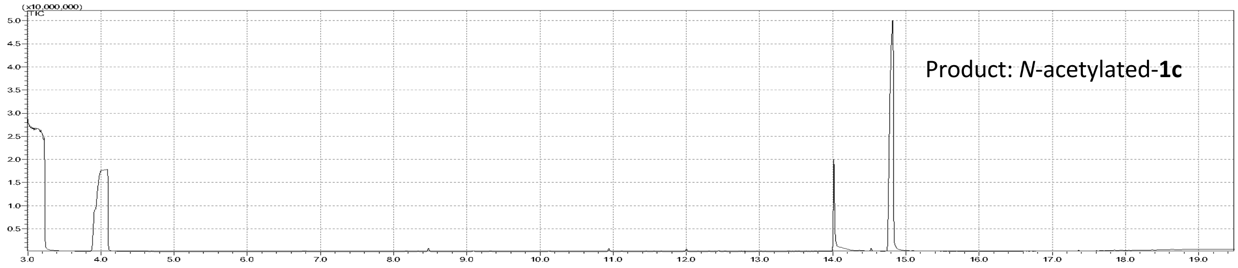

GC-MS chromatogram of Reaction D (monitor of progress of reaction):

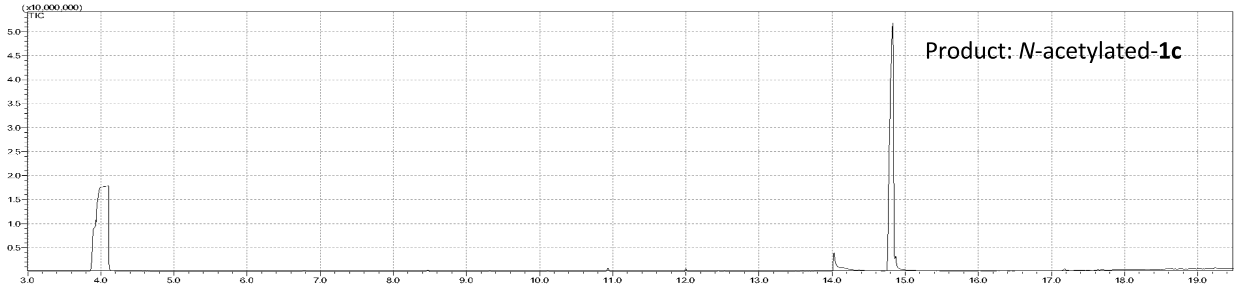

Representative GC-MS chromatogram of Reactions A-D after purification work-up:

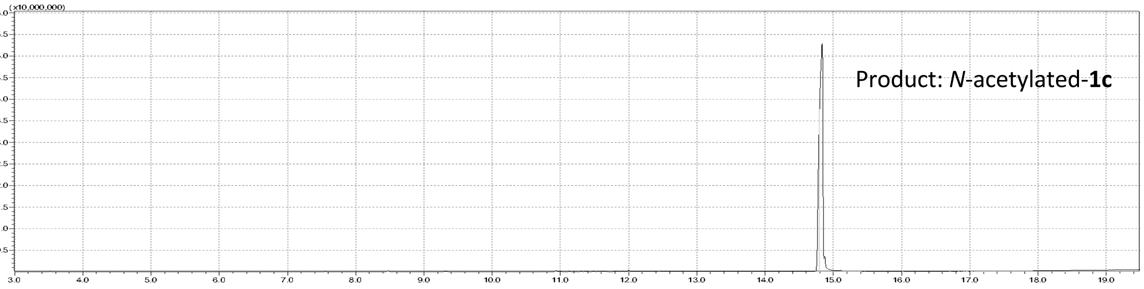

(3) Methylation of acetylated optically active 1c and racemic 1c from step (2)

Reaction A: The chemical reactions were performed under dinitrogen atmosphere in a Schlenk tube. Sodium hydride (4 eq., 1.37 mmol, 33 mg; added NaH as 60% oil dispersion, 55 mg) was washed with dried *n*-hexane to remove the oil. After that, the Schlenk tube was cooled down to 0 °C with an acetone/dinitrogen bath and dried THF (4 mL) was added. Bubbling was observed due to hydrogen formation. Under stirring, the (1*R*,3*S*)-configured *N*-acetylated **1c** compound from previous reaction step (53.2 mg, 0.343 mmol, 155.13 g/mol) was added and the reaction was performed for 2 h at 0 °C. Then, the acetone/ice bath was removed, MeI (3 eq., 65 µL, 1.029 mmol, 141.9 g/mol) was added and the chemical reaction was run overnight at room temperature. The next day, 100 µL were taken, centrifuged, diluted with CH_2Cl_2 (100 µL) and analyzed via GC-MS. Full conversion was observed. Then, on ice, K_2CO_3 (10 mL, 20% solution) was stepwise added (smoking was observed); the organic compound was extracted with CH_2Cl_2 (2 x 10 mL), dried over anhydrous $MgSO_4$ and the solvent was removed under reduced pressure. 25.8 mg as yellow oil (0.1525 mmol, 169.15 g/mol).

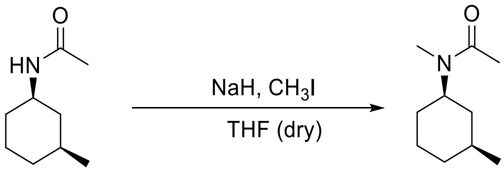

(1*R*,3*S*)-*N*-acetyl-**1c** (1*R*,3*S*)-*N*-acetyl-**1d**

GC-MS chromatogram: 15.1 min - only 1 main peak, almost no impurities

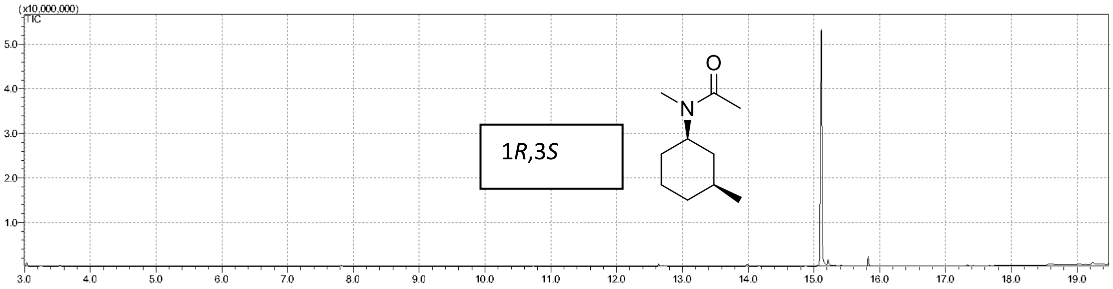

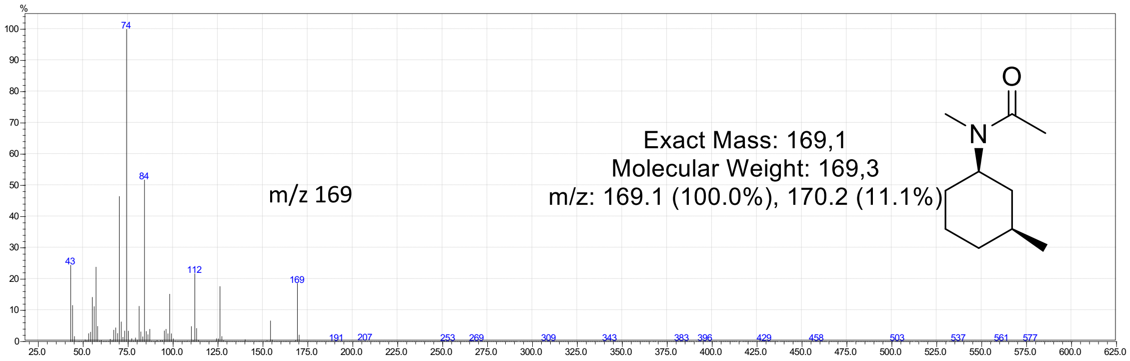

Reaction B: The methylation was performed in the same way as for reaction A using following reagents: sodium hydride (4 eq., 1.27 mmol, 31 mg, added NaH as 60% oil dispersion, 51 mg), dried THF (4 mL), (1*S*,3*R*) configured *N*-acetylated compound from previous reaction step (49.4 mg, 0.318 mmol, 155.13 g/mol) and MeI (3 eq., 60 µL, 0.954 mmol,141.9 g/mol). GC-MS analysis showed full conversion. After work-up, 35.9 mg as slightly yellowish oil (0.212 mmol, 169.15 g/mol) were obtained.

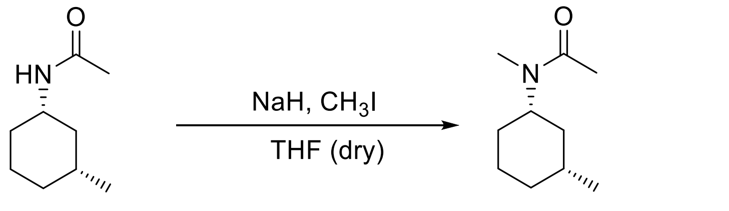

(1*S*,3*R*)-*N*-acetyl-**1c** (1*S*,3*R*)-*N*-acetyl-**1d**

GC-MS chromatogram: 15.1 min; m/z: 169; only 1 main peak, almost no impurities

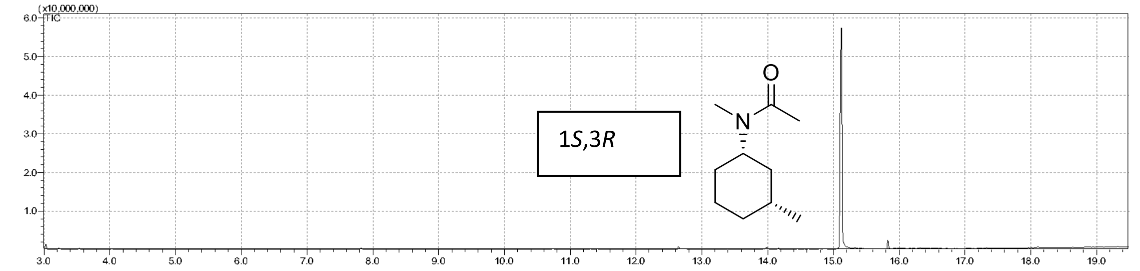

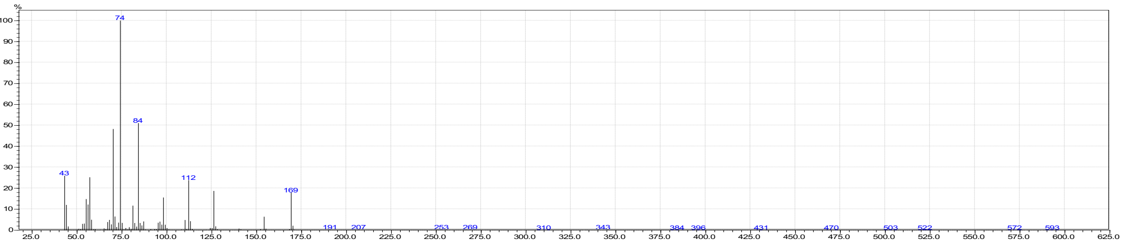

Reaction C: The methylation was performed in the same way as for reaction A using following reagents: sodium hydride (4 eq., 1.73 mmol, 41 mg, added NaH as 60% oil dispersion, 77 mg), dried THF (4 mL), (1*R*,3*R*)+(1*S*,3*R*) configured *N*-acetylated compound from previous reaction step (67.3 mg, 0.434 mmol, 155.13 g/mol) and MeI (3 eq., 81 µL, 1.302 mmol, 141.9 g/mol). GC-MS analysis showed full conversion. After work-up, 33.8 mg as slightly yellowish oil (0.20 mmol, 169.15 g/mol) were obtained.

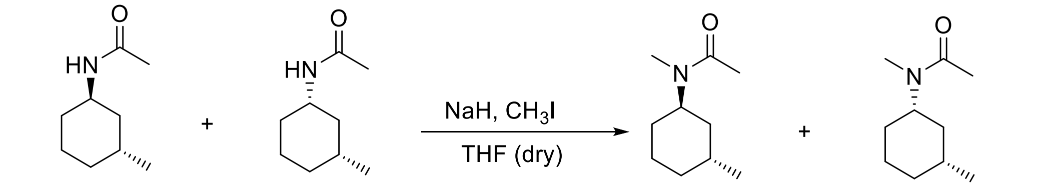

(1*R*,3*R*)-*N*-acetyl-**1c** (1*R*,3*S*)-*N*-acetyl-**1c** (1*R*,3*R*)-*N*-acetyl-**1d** (1*R*,3*S*)-*N*-acetyl-**1d**

GC-MS chromatogram; 2 peaks at 15.1 and 15.2 min, m/z of both: 169

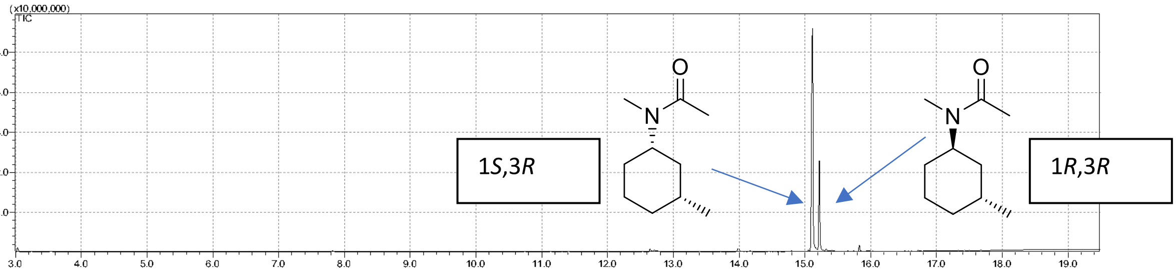

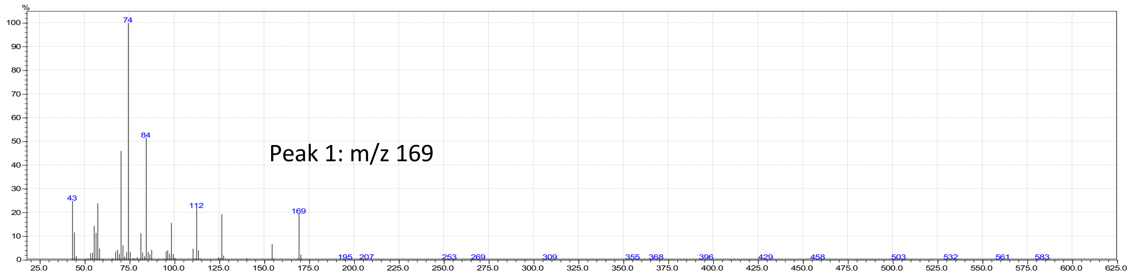

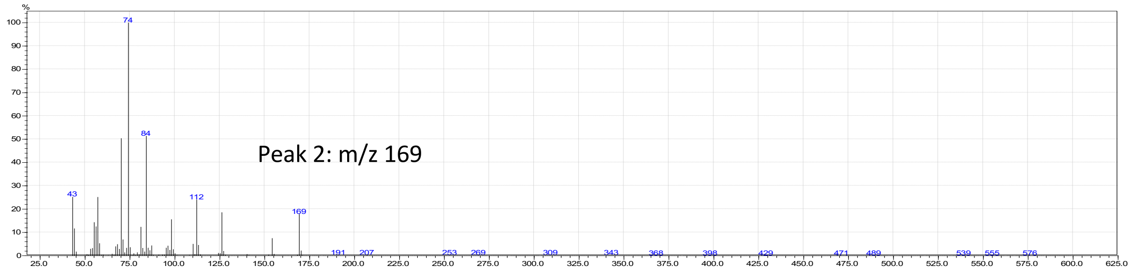

Reaction D: The methylation was performed in the same way as for reaction A using following reagents: sodium hydride (4 eq., 2.5 mmol, 62 mg, added NaH as 60% oil dispersion, 103 mg), dried THF (10 mL), racemic configured *N*-acetylated compound from previous reaction step (99.7 mg, 0.643 mmol, 155.13 g/mol) and MeI (2 eq., 80 µL, 1.290 mmol, 141.9 g/mol). GC-MS analysis showed high conversion into the desired product, but a tiny bit of starting material remained as only 2 equivalents of MeI were added during the reaction. However, this was enough to fulfil our goal of obtaining racemic reference compound. After work-up, 33.8 mg as slightly yellowish oil (0.20 mmol, 169.15 g/mol) were obtained.

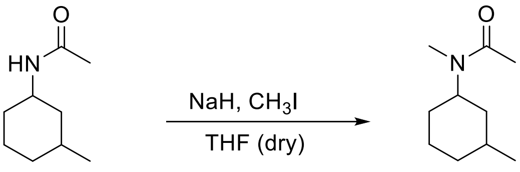

rac-*N*-acetyl-**1c** *rac*-*N*-acetyl-**1d**

GC-MS chromatogram; 2 product peaks at 15.1 and 15.2 min, m/z of both: 169

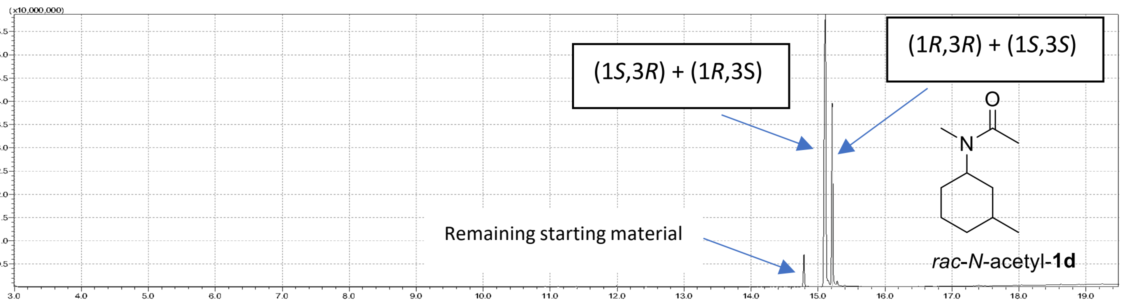

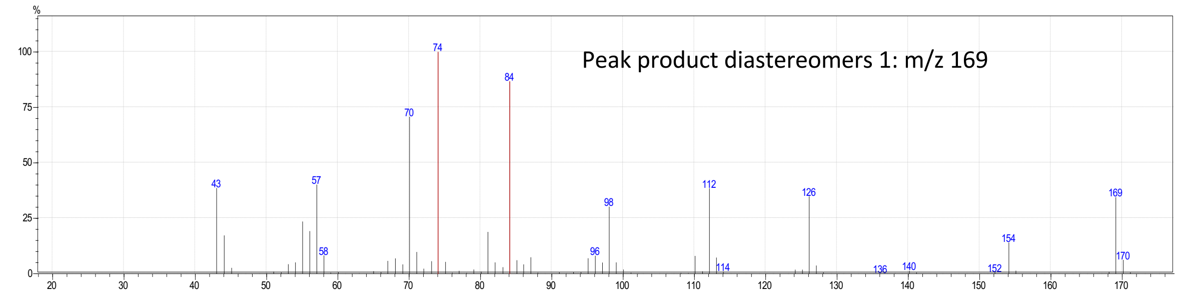

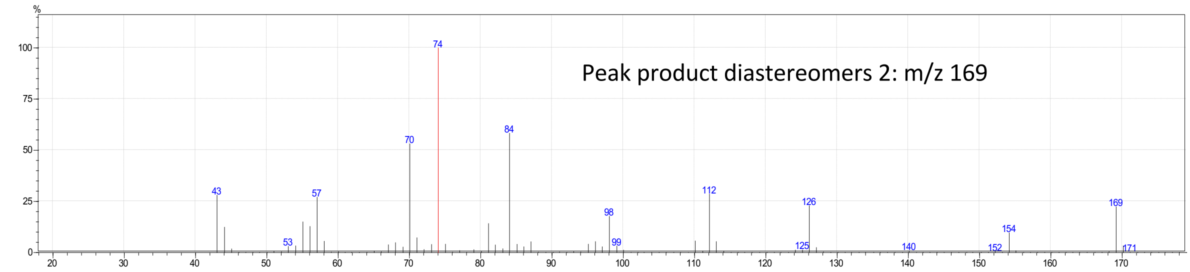

Table S50. Diastereomeric and enantiomeric composition for the semi-preparative reactions (chiral GC measurement) after chemical methylation.

	(1*S*,3*R*)-**1d** (%)	(1*R*,3S)-**1d** (%)	(1*S*,3*S*)-**1d** (%)	(1*R*,3*R*)-**1d** (%)
(A) finished	n.d.	99	n.d.	1
(B) finished	>99	n.d.	n.d.	n.d.
(C) finished	84	n.d.	n.d.	16

GC chromatograms of reference compounds with chiral column (CP-Chirasil (Dex-CB, CP7503) 25m x 0.32 mm x 0.25 µm, Agilent; analytical method: Dex-CB-2 method amid; see SI section 13 for details).

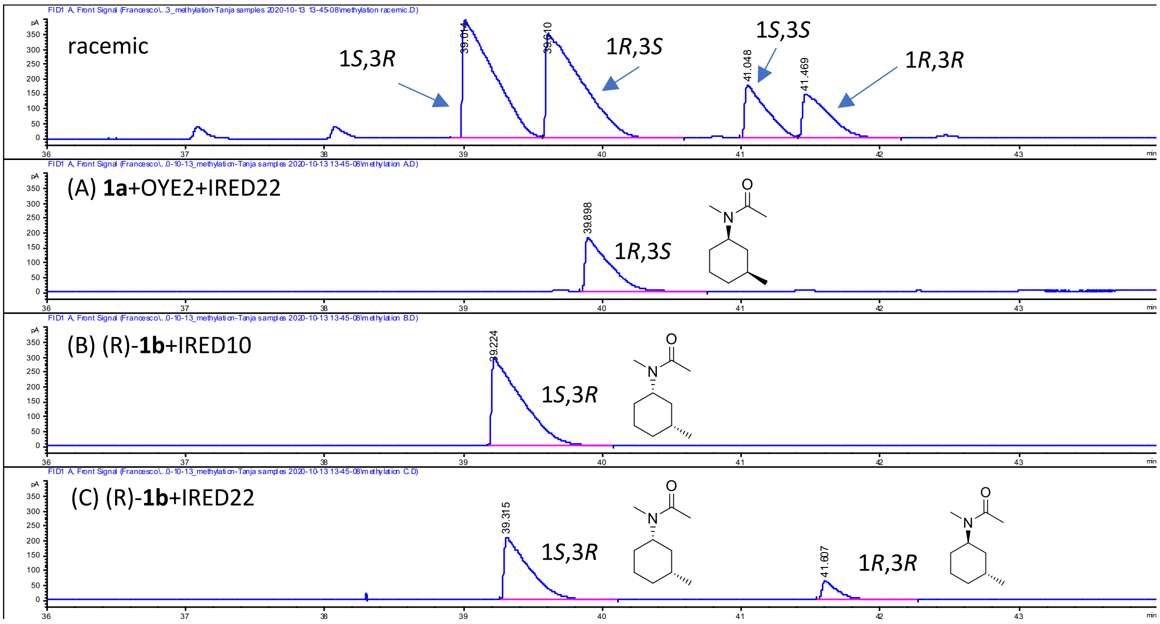

12.1.5. <u>*Representative NMRs of final product:*</u>

<u>Reaction A</u>

¹H-NMR (solvent $CDCl_3$)

Comment: It is clearly visible that some peaks are split in two. The reason for that is the possibility to have two conformations for the acetyl group of the amide that are not converting quickly at room temperature. The situation is analogous as the one described in section 12.3.5 (see that section for explanation).

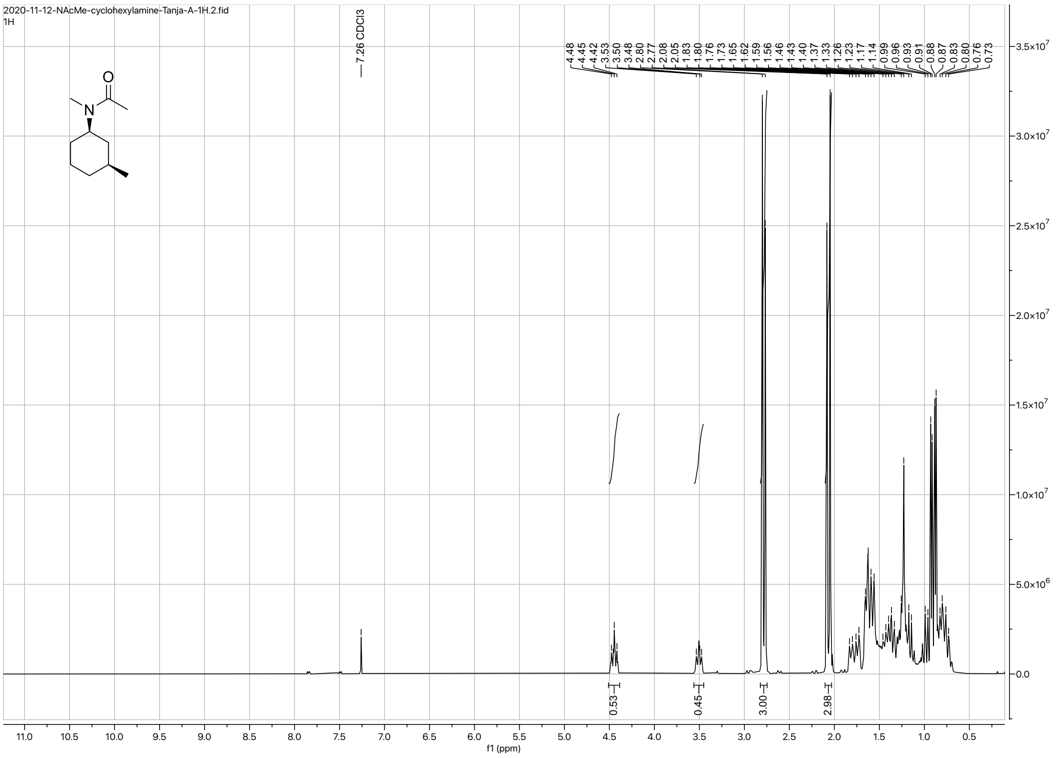

¹H COSY NMR (solvent $CDCl_3$)

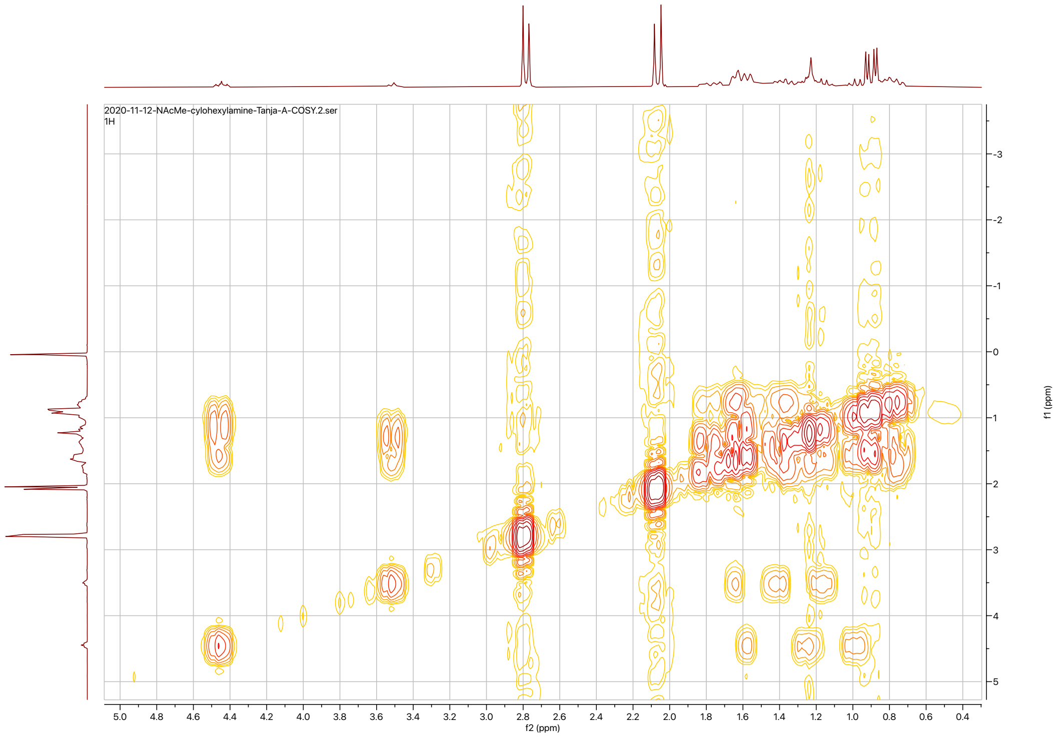

^{13}C-NMR (solvent $CDCl_3$)

Comment: All the peaks are split in two. The reason for that is the possibility to have two conformations for the acetyl group of the amide that are not converting quickly at room temperature. The situation is analogous as the one described in section 12.3.5.

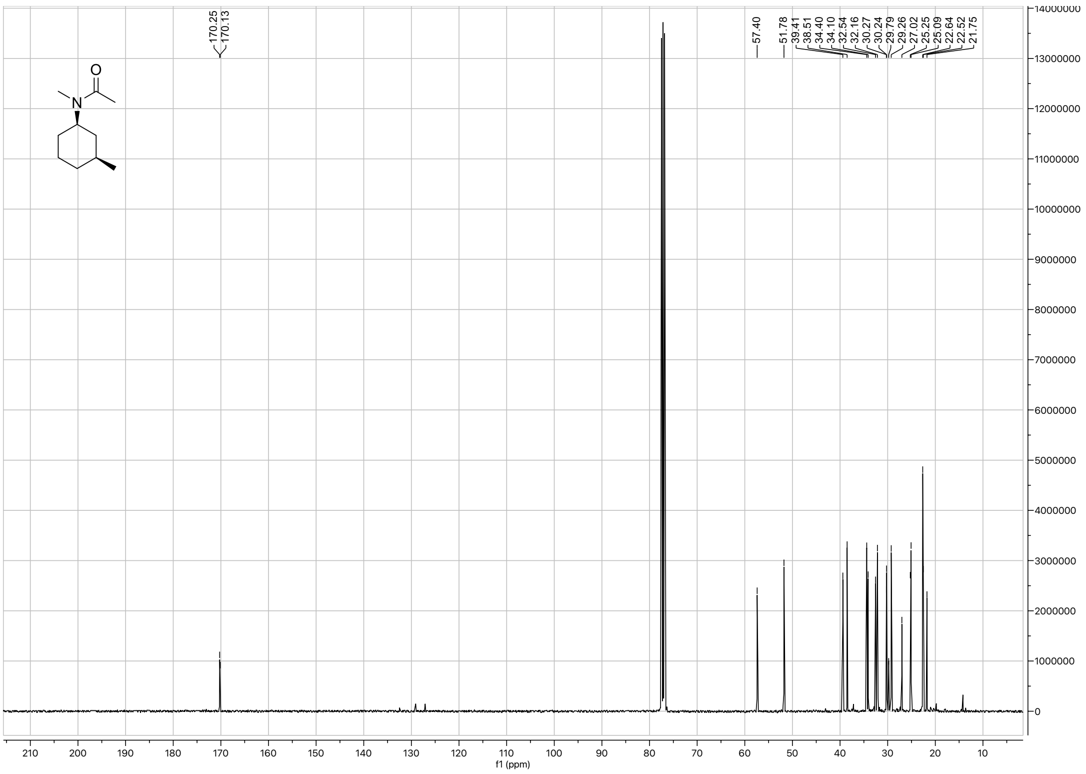

Reaction B

^{1}H-NMR (solvent $CDCl_3$)

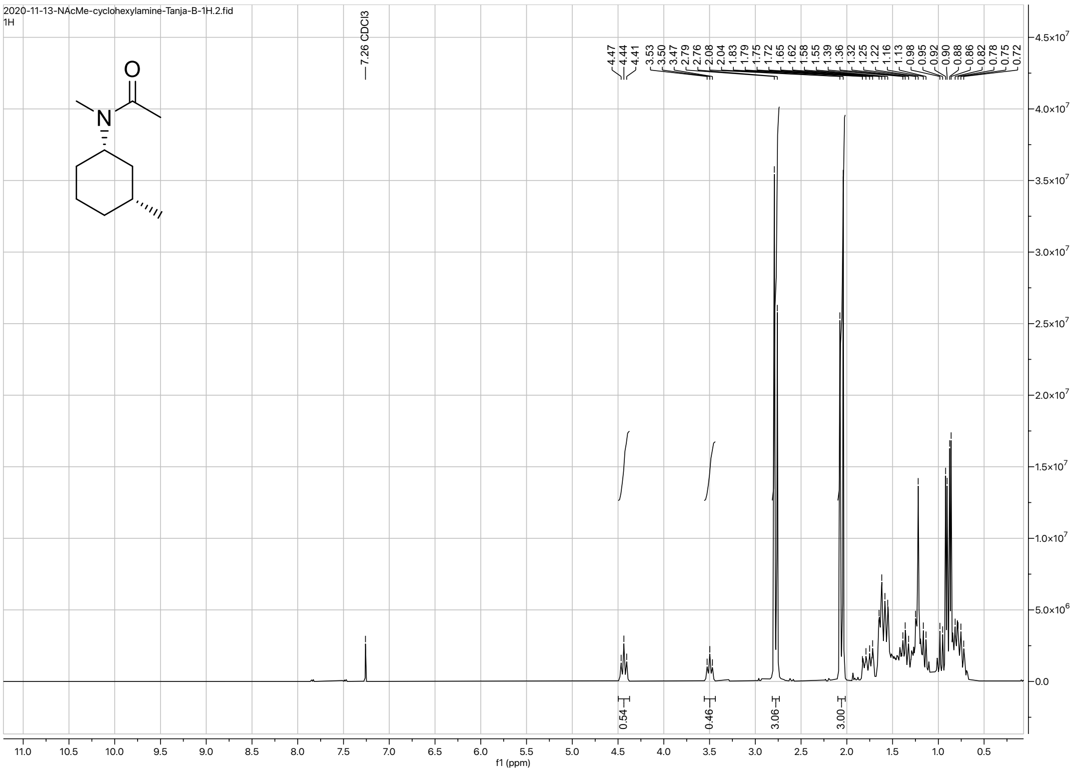

^{1}H COSY NMR (solvent $CDCl_3$)

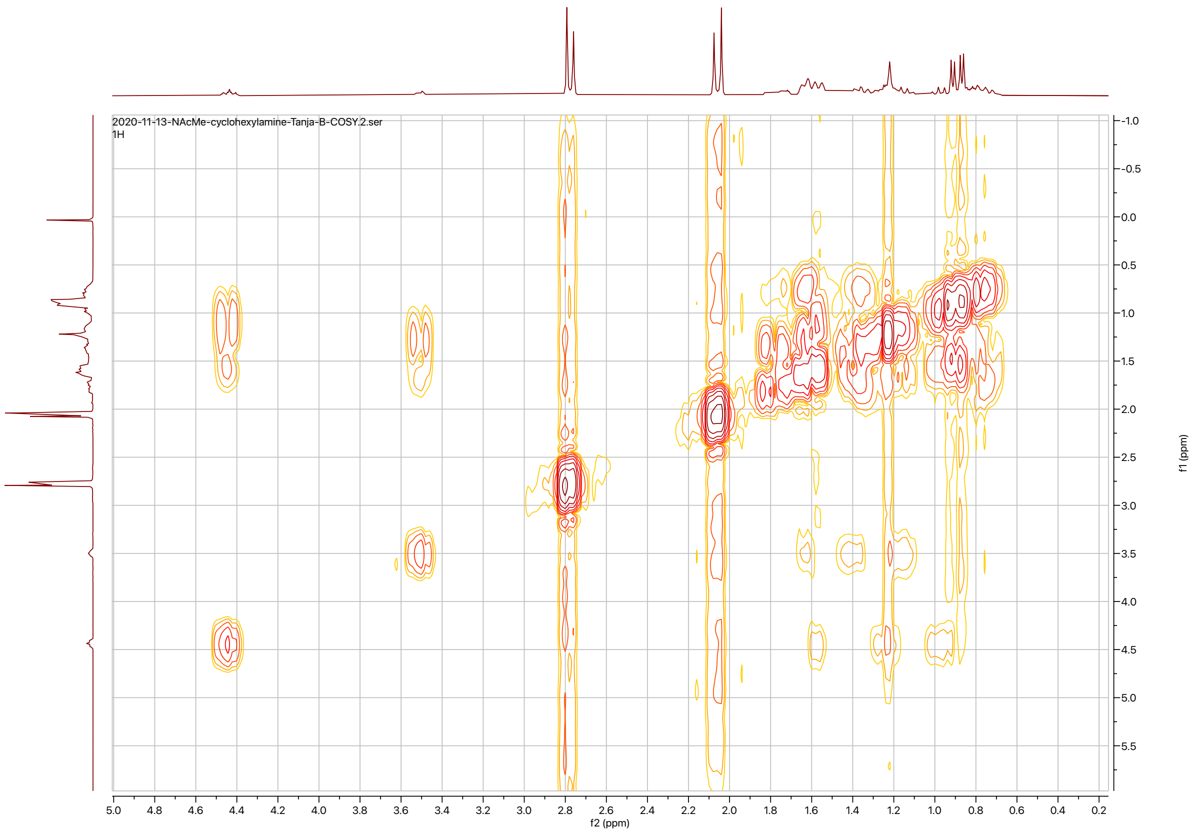

^{13}C-NMR (solvent $CDCl_3$)

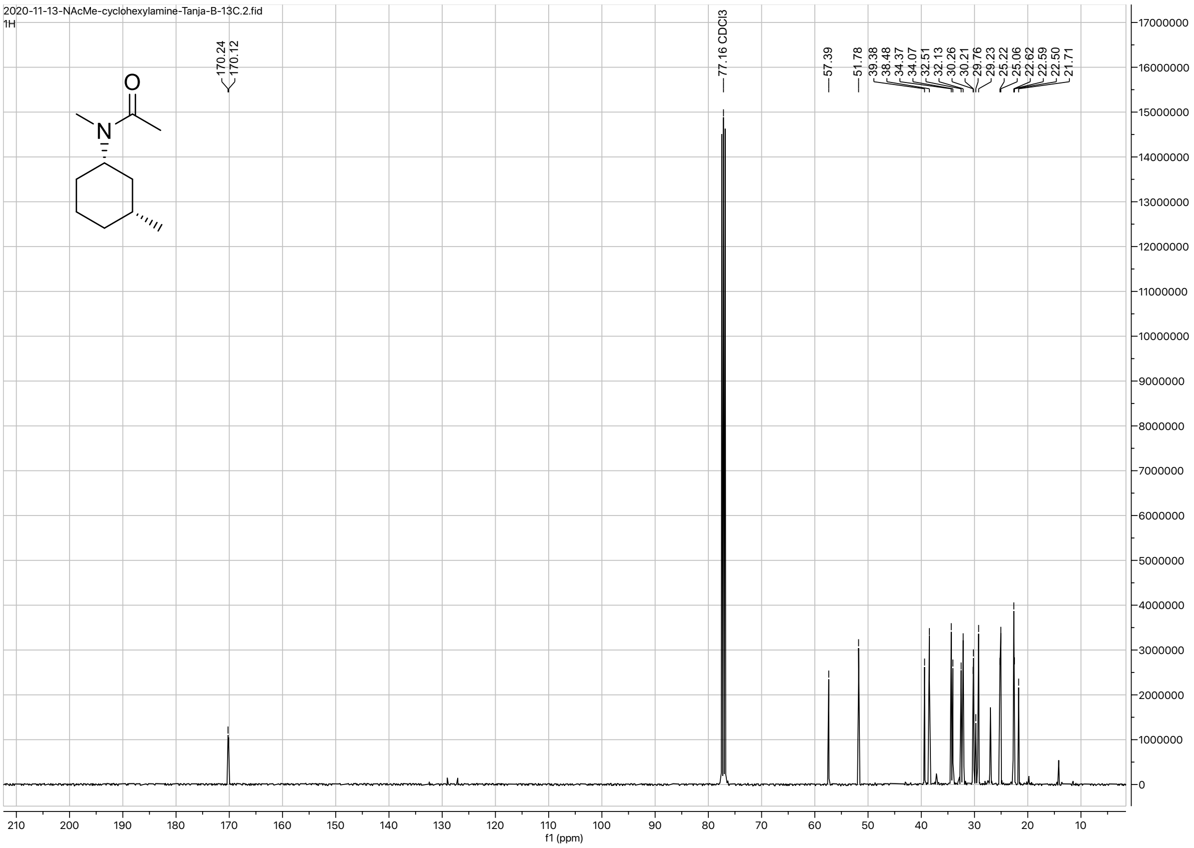

12.2. Substrates 2a-d

12.2.1. References for **2c** using ωTAs on analytical scale

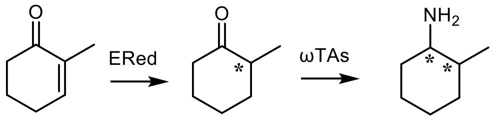

2a **2b** **2c**

Table S51. Conversion (%) for substrate **2a** or *rac*-**2b** using ωTAs or an ERed in combination with ωTAs for obtaining the four stereoisomers of the primary amine **2c**. (GC measurement with an achiral column)

substrate	ERed	ωTA	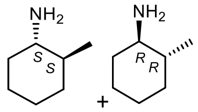	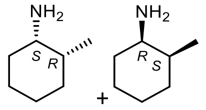	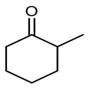	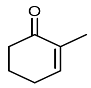
			(1*S*,2*S*)-**2c** + (1*R*,2*R*)-**2c**	(1*S*,2*R*)-**2c** + (1*R*,2*S*)-**2c**	*rac*-**2b**	**2a**
**2a**	TOYE	-			82	18
rac-**2b**	-	Vf-ωTA (S)	21	23	56	n.d.
rac-**2b**	-	As(R)-ωTA (R)	n.d.	n.d.	>99	n.d.
rac-**2b**	-	Cv-ωTA (S)	38	33	29	n.d.
**2a**	TOYE	Vf-ωTA (S)	6	23	71	n.d.
**2a**	TOYE	As(R)-ωTA (R)	n.d.	n.d.	>99	<1
**2a**	TOYE	Cv-ωTA (S)	8	48	43	1

Table S52. Diastereomeric and enantiomeric composition (%) for **2c** (GC measurement with a chiral column) after derivatization to the acetamide. Peak annotation was done according to the literature. [30]

substrate	ERed	ωTA	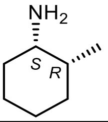	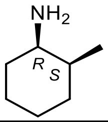	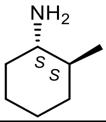	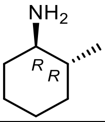
			**2c**-peak1 [%]	**2c**-peak2 [%]	**2c**-peak3 [%]	**2c**-peak4 [%]
rac-**2b**	-	Vf-ωTA (S)	51.6	n.d.	47.6	0.8
rac-**2b**	-	Cv-ωTA (S)	45.9	0.5	52.6	1.0
**2a**	TOYE	Vf-ωTA (S)	78.8	n.d.	20.7	0.5
**2a**	TOYE	Cv-ωTA (S)	85.1	n.d.	13.5	1.4

Comments: the TOYE reference reaction showed an *e.r.* of >95:<5 (*R*)-**2b** (reaction was done at 30 °C). Moreover, the only (*R*)-selective ωTA that was tested did not show any conversion. Therefore, further ωTAs were tested (see Table S53). The *e.r.* of the remaining (i.e., unreacted) saturated intermediate was also measured and reported in Table S54.

Table S53. Conversion (%) for substrate **2a** or *rac*-**2b** using ωTAs or an ERed in combination with ωTAs for obtaining the four stereoisomers of the primary amine **2c** (GC measurement with an achiral column). Moreover, the *e.r.* for the remaining saturated intermediate **2b** was also measured (GC measurement with a chiral column).

substrate	ERed	T (°C)	ωTA	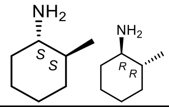	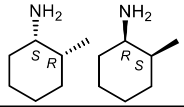	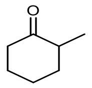	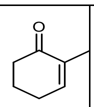	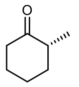
				(1*S*,2*S*)-**2c** + (1*R*,2*R*)-**2c**	(1*S*,2*R*)-**2c** + (1*R*,2*S*)-**2c**	*rac*-**2b**	**2a**	*e.r.* (*R*)-**2b**
**2a**	TOYE	30	-			98	2	95:5
**2a**	TOYE	20	-			94	6	96:4
**2a**	TOYE	30	Cv (S)	16	35	49	n.d.	*rac*
**2a**	TOYE	20	Cv (S)	13	44	42	<1	*rac*
**2a**	TOYE	30	At (R)	2	n.d.	98	n.d.	*rac*
**2a**	TOYE	20	At (R)	2	n.d.	98	<1	*rac*
**2a**	TOYE	30	Hn (R)	n.d.	n.d.	>99	n.d.	*rac*
**2a**	TOYE	20	Hn (R)	n.d.	n.d.	>99	n.d.	*rac*
**2a**	TOYE	30	As(R)mut11	31	56	9	4	*rac*
**2a**	TOYE	20	As(R)mut11	25	47	10	18	*rac*
rac-**2b**	-	30	Cv (S)	44	25	31	n.d.	*rac*
rac-**2b**	-	30	At	4	n.d.	96	n.d.	*rac*
rac-**2b**	-	30	Hn	1	<1	98	n.d.	*rac*
rac-**2b**	-	30	As(R)mut11	32	62	6	n.d.	*rac*

The single TOYE reaction gives an *e.r.* of 95:5 (*R*). However, when TOYE was combined with a ωTA, the *e.r.* of the remaining intermediate **2b** in solution is racemic. Therefore, the remaining (unreacted) **2b** racemizes when it is in solution during the ωTA-catalyzed reaction.

Table S54. Diastereomeric and enantiomeric composition (%) for **2c** (GC measurement with a chiral column) after derivatization to the acetamide. Peak annotation was done according to the literature. [30]

substrate	ERed	T (°C)	ωTA	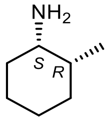	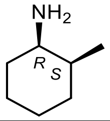	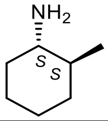	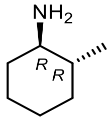
				(1*S*,2*R*)-**2c**	(1*R*,2*S*)-**2c**	(1*S*,2*S*)-**2c**	(1*R*,2*R*)-**2c**
**2a**	TOYE	30	Cv (S)	67	n.d.	29	4
**2a**	TOYE	20	Cv (S)	76	n.d.	21	3
**2a**	TOYE	30	As(R)mut11	<1	63	2	34
**2a**	TOYE	20	As(R)mut11	<1	63	2	34
rac-**2b**	-	30	Cv (S)	37	n.d.	61	2
rac-**2b**	-	30	As(R)mut11	<1	65	2	33

A co-injection of the Cv(S)-ωTA-catalyzed and As(R)mut11-ωTA-catalyzed reactions also clearly shows that these enzymes produced amines with opposite configuration at C-1 (see GC chromatograms on next page):

GC-FID chromatograms measured with chiral column for the **2c** isomers from ωTA reactions. Peak annotation was done according to the literature. [30]

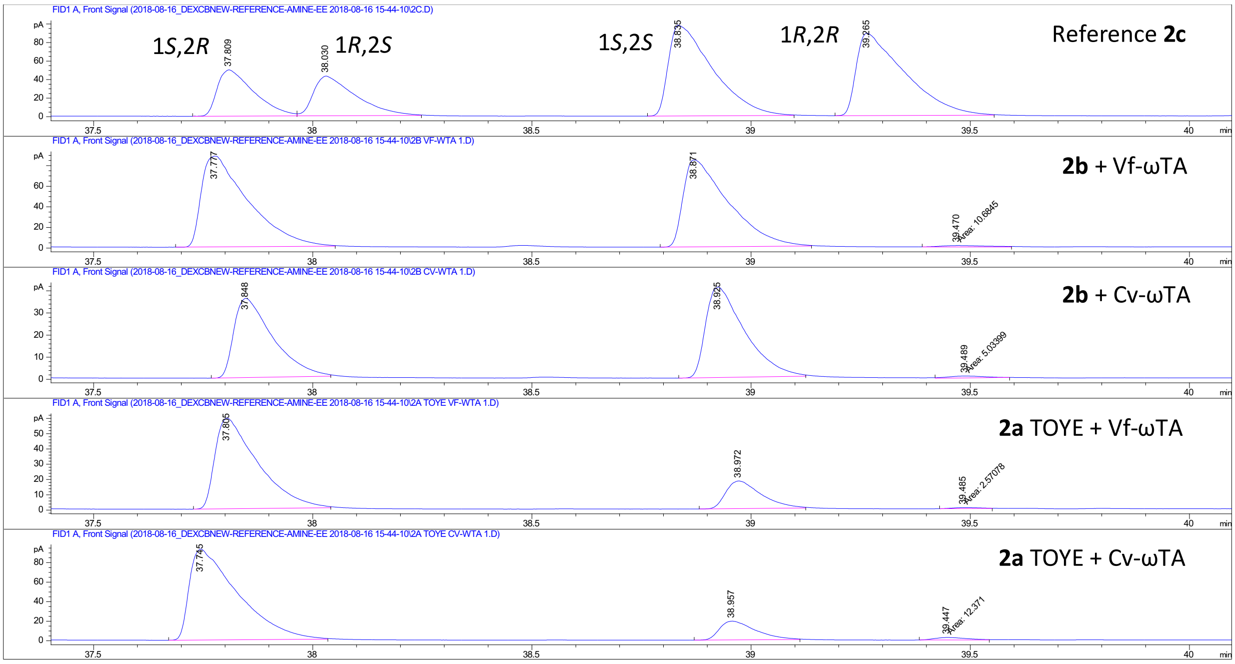

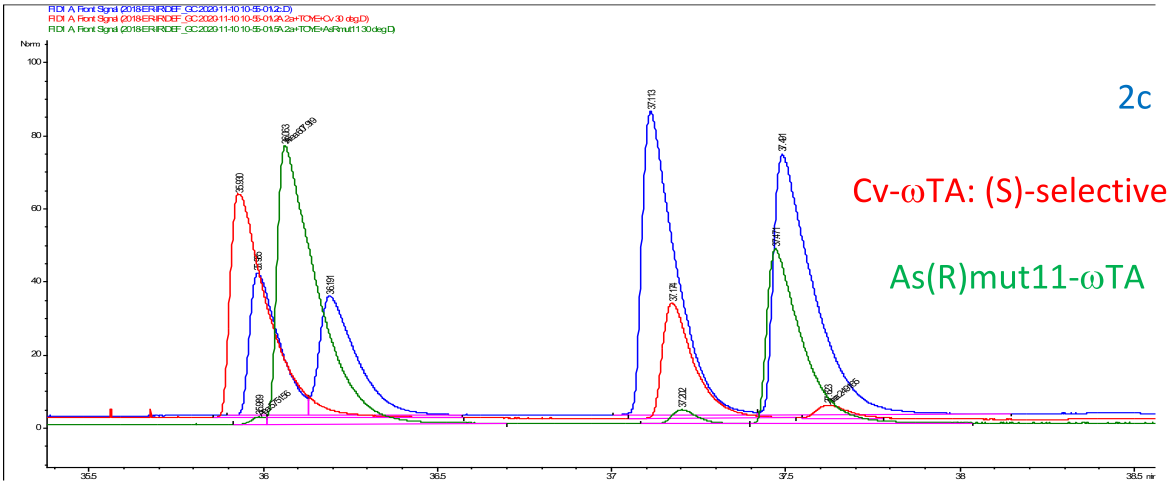

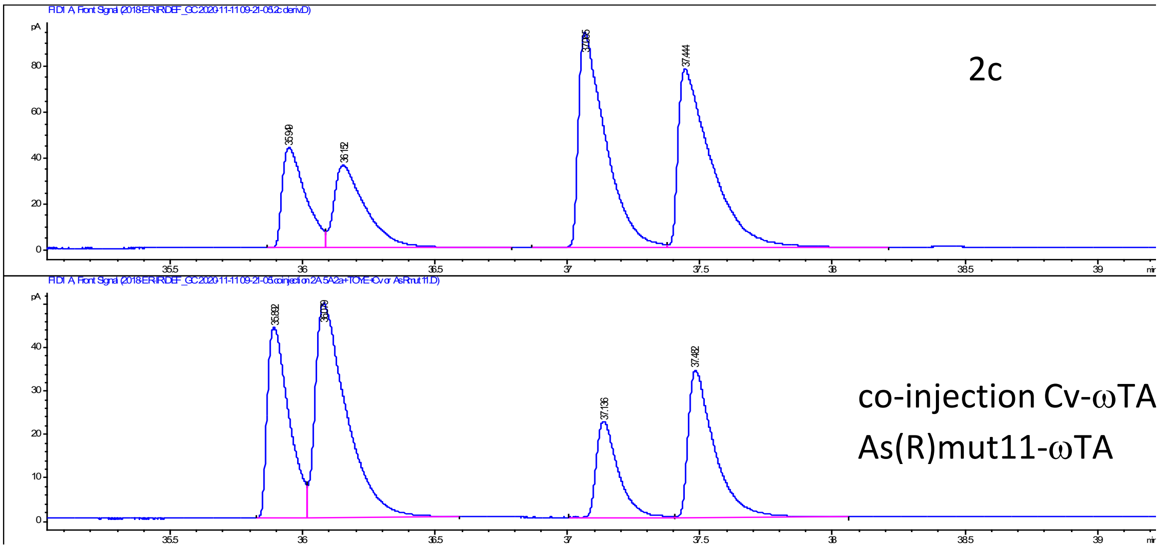

12.2.2. *Chemical synthesis of rac-**2d***

Step 1: carbamate formation: The synthesis of the carbamate compound **8** was performed by reacting the amine *rac*-**2c** (4.558 mmol, 516 mg, 0.6 mL) with ethyl-chloroformate (**7**, 11.395 mmol, 1.233 g, 1.1 mL, 2.5 eq.) in the presence of pyridine (2.934 g, 11.395 mmol, 3 mL) as catalyst in a mixture of EtOH/H_2O (12/23 mL v/v). The ratio between pyridine/EtOH/H_2O was 8:32:60. The reaction was complete after 2.5 h at room temperature leading to the formation of compound **8** as a mixture of diastereomers according to GC-MS analysis. The isolated product was obtained in 52% yield (440 mg) as a yellow liquid and used in the next step without any further purification.

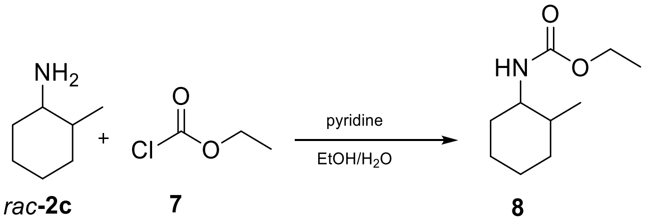

Step 2: carbamate reduction: The crude product **8** (2.375 mmol, 440 mg) was directly used in the reduction to the corresponding methylated amine *rac*-**2d** by $LiAlH_4$ (2 M stock in THF; 5.938 mmol, 225 mg, 2.5 eq., 6 mL) in THF (16 mL). The reaction was complete after 3.5 h leading to the formation of compound *rac*-**2d** as a mixture of diastereomers according to GC-MS analysis. The isolated product was obtained in 42% yield (128 mg) as a yellow liquid. The final product *rac*-**2d** was also analyzed by ^{1}H-NMR (a complex spectrum due to the presence of stereoisomers).

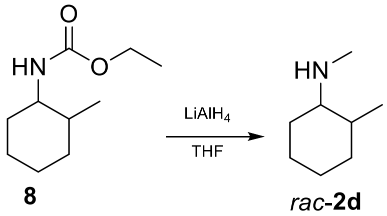

GC-MS analysis isolated carbamate **8**

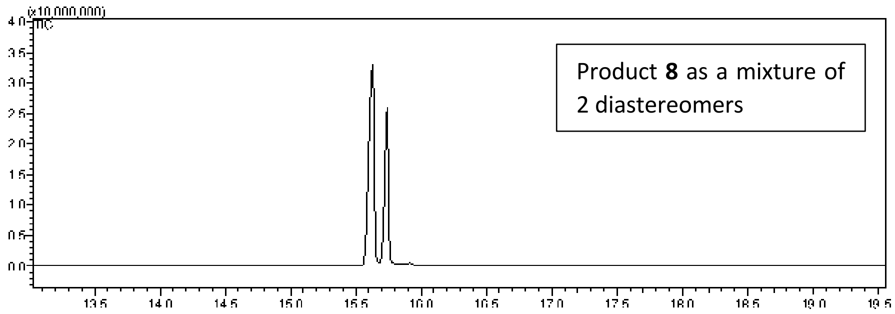

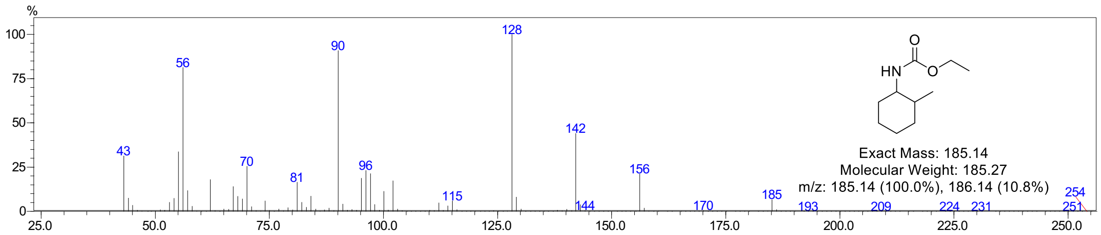

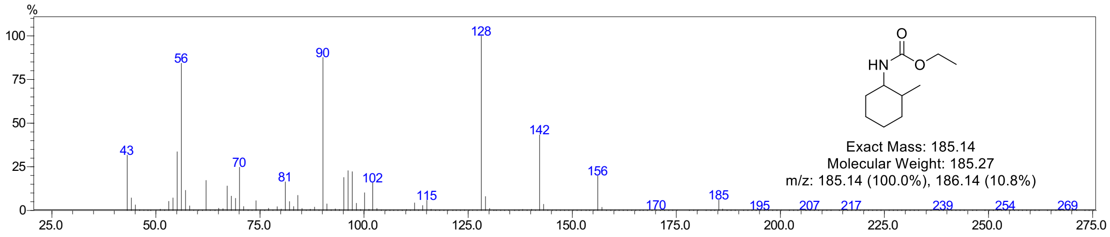

GC-MS analysis isolated compound *rac*-**2d**

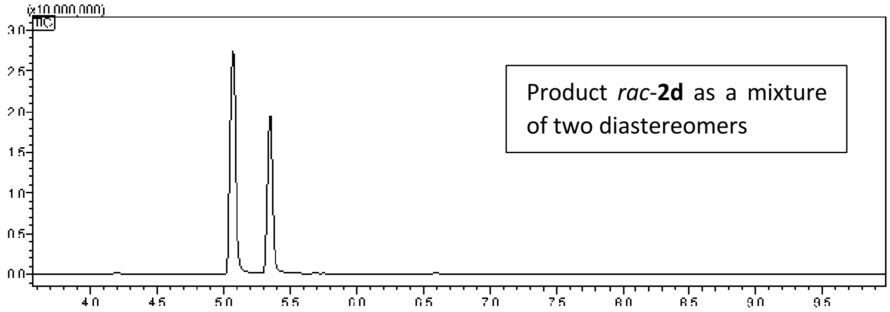

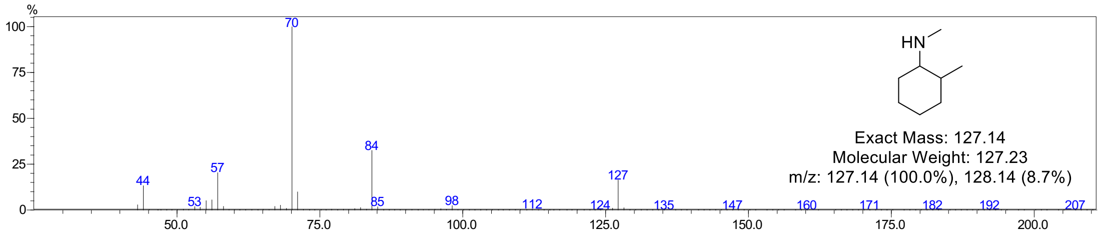

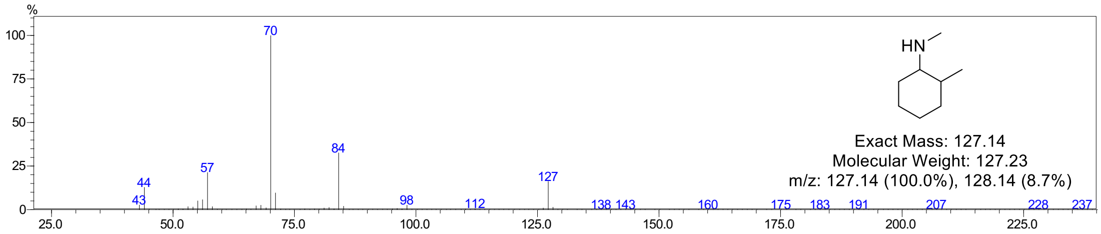

^{1}H-NMR isolated compound *rac*-**2d** (solvent $CDCl_3$)

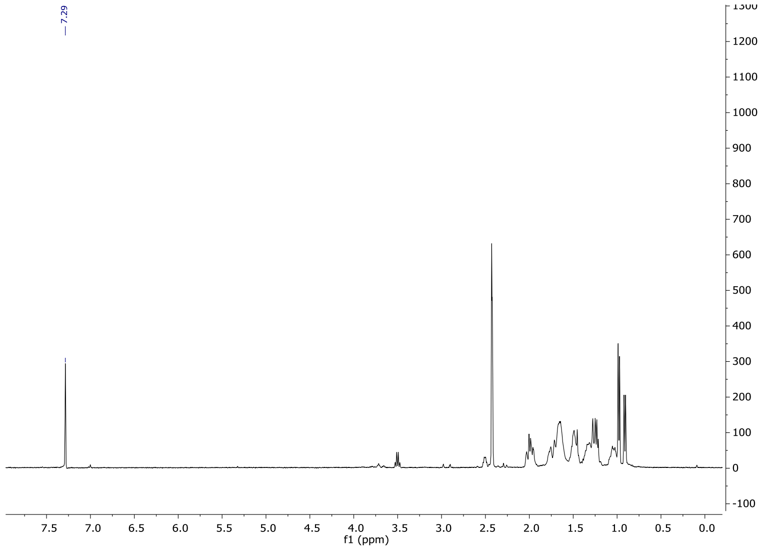

12.3. Substrate 3a-d

12.3.1. References for **3c** with ωTAs on analytical scale

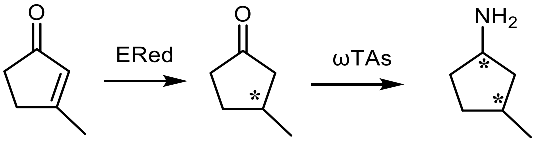

3a **3b** **3c**

Table S55. Conversion (%) for substrate **3a** or *rac*-**3b** or (*R*)-**3b** using ωTAs and an ERED in combination with ωTAs for obtaining the four diastereomers of the primary amine **3c**. (GC measurement with an achiral column)

substrate	ERed	ωTA	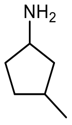	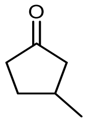	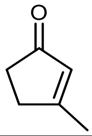
			**3c**	**3b**	**3a**
**3a**	PETNR	-		24	76
**3b**	-	Vf-ωTA (S)	33	67	n.d.
**3b**	-	As(R)-ωTA (R)	7	93	n.d.
**3b**	-	Cv-ωTA (S)	67	33	n.d.
(*R*)-**3b**	-	Vf-ωTA (S)	29	68	3
(*R*)-**3b**	-	As(R)-ωTA (R)	2	98	n.d.
(*R*)-**3b**	-	Cv-ωTA (S)	59	41	n.d.
**3a**	PETNR	Vf-ωTA (S)	81	11	8
**3a**	PETNR	As(R)-ωTA (R)	3	47	50
**3a**	PETNR	Cv-ωTA (S)	21	13	66

With any of the tested achiral (Agilent HP1, HP5, DB200, DB1701), only one peak for the amine products was observed. Therefore, diastereomers are not separated in the case of **3c** with typical achiral columns in contrast to the case of compounds **1c**, **2c** and **4c**.

Table S56. Diastereomeric and enantiomeric composition (%) for **3c** (GC measurement with a chiral column) after derivatization to the acetamide.

			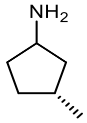	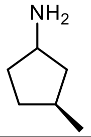	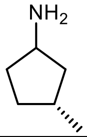	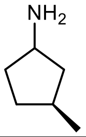
substrate	ER	ωTA	**3c**-peak1 [%]	**3c**-peak2 [%]	**3c**-peak3 [%]	**3c**-peak4 [%]
**3b**	-	Vf-ωTA (S)	25	27	25	23
**3b**	-	As(R)-ωTA (R)	n.d.	5	5	90
**3b**	-	Cv-ωTA (S)	35	27	13	25
(R)-**3b**	-	Vf-ωTA (S)	48	n.d.	52	n.d.
(R)-**3b**	-	Cv-ωTA (S)	70	n.d.	30	n.d.
**3a**	PETNR	Vf-ωTA (S)	n.d.	44	n.d.	56
**3a**	PETNR	Cv-ωTA (S)	n.d.	47	n.d.	53

note: The PETNR blank reaction yields an *e.r.* >99.8:<0.2 ((*S*)-enantiomer)

Comment: The tested ωTAs do not show a clear stereoselectivity for the reduction of the carbonyl moiety of substrates (*R*)-**3b** or (*S*)-**2b** (the latter is generated in situ by stereoselective reduction of **3a** with PETNR).

Furthermore, by performing GC analytics with chiral columns (i.e., the best performing is Agilent Chirasil DEX-CB column as reported below), peak 1+2 and peak 3+4 are not completely separated.

The amination of (*R*)-**3b** affords the diastereomers that eluted as peak 1 and 3. The amination of the in situ generated (*S*)-**3b** (99.7:0.3 *e.r.*, obtained starting from **3a** by reduction with PETNR), affords the diastereomers that eluted as peak 2 and 4.)

GC-FID chromatograms for the separation of the **3c** isomers from ωTA reactions:

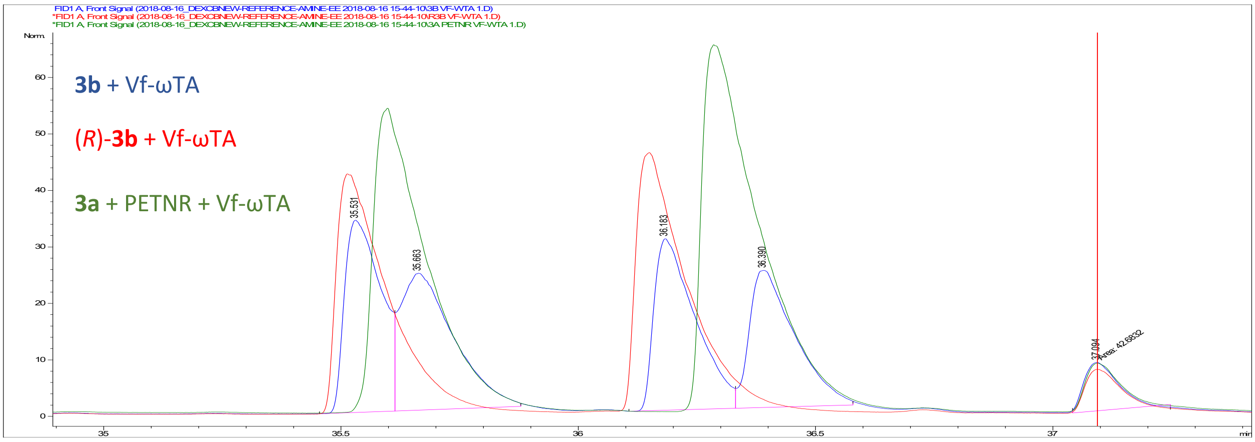

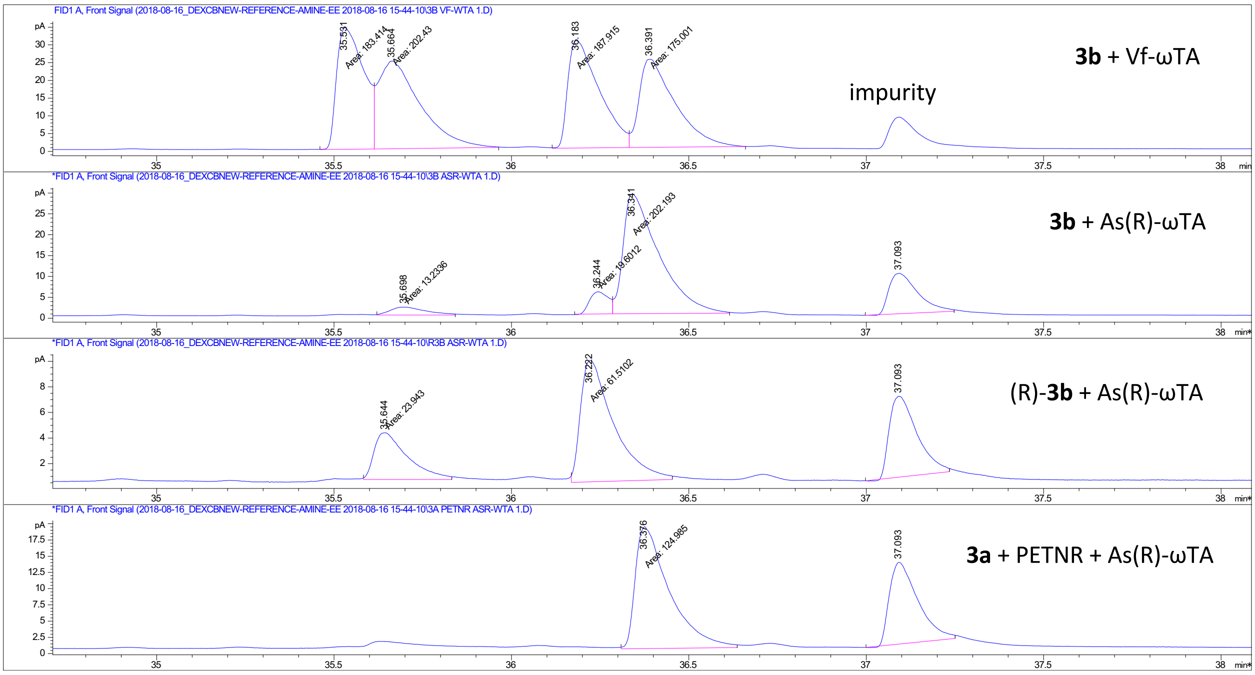

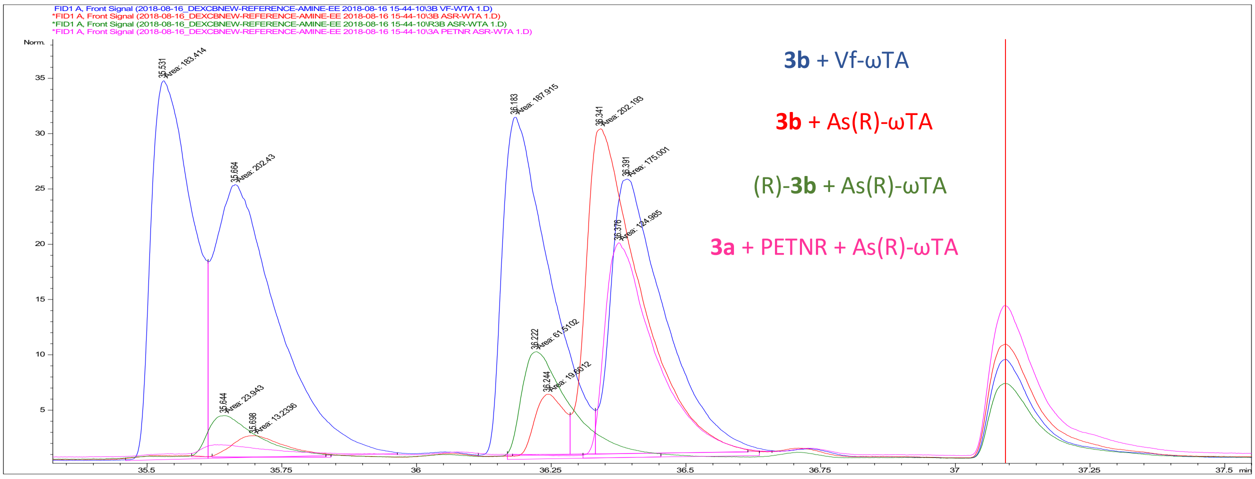

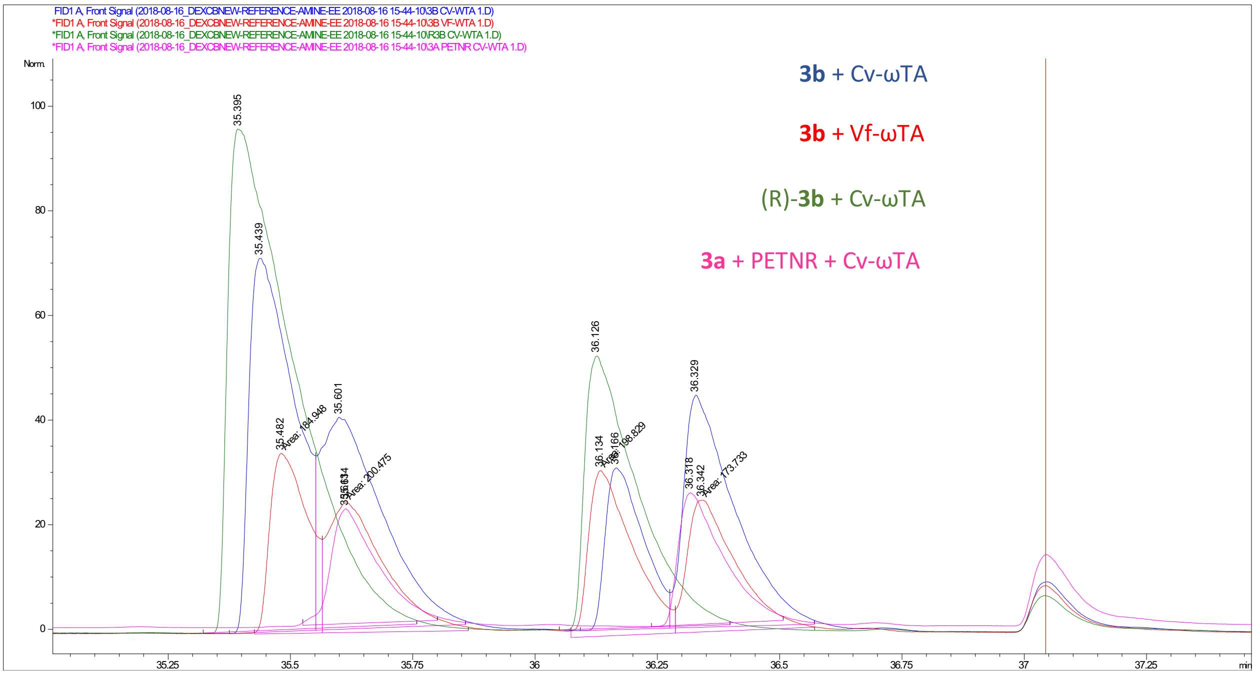

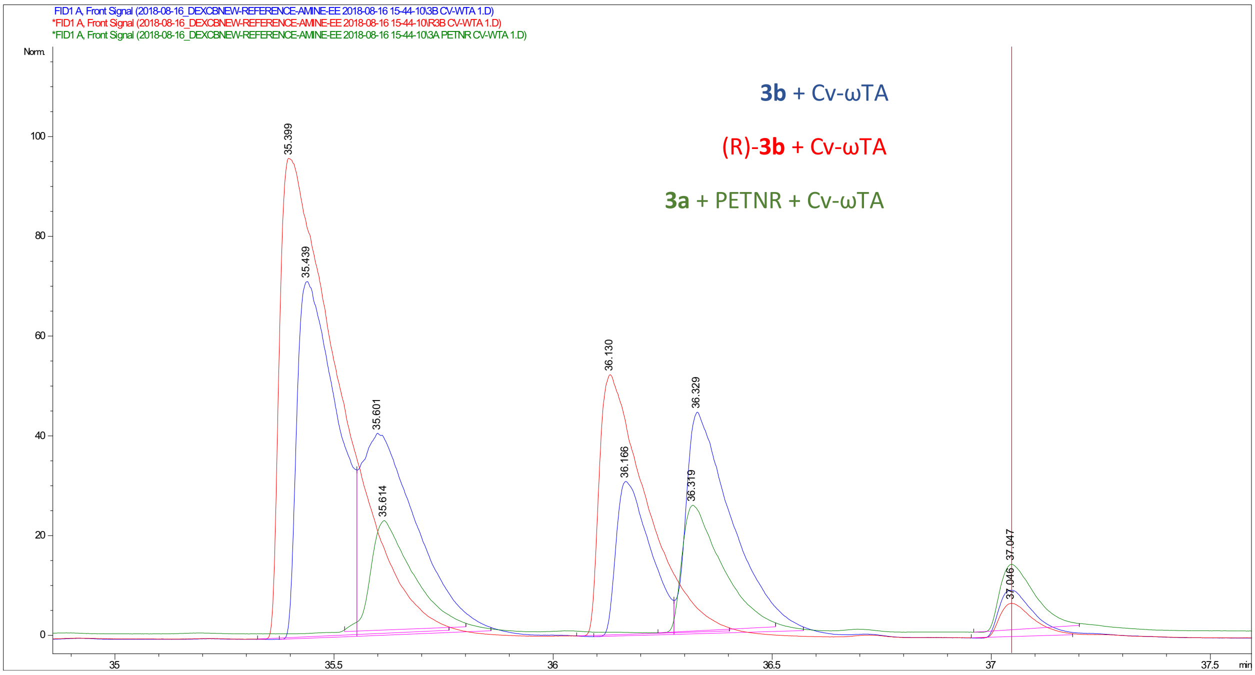

Finally, the conversion to the amine was measured with an Agilent DB1701-30 m. Total conversion of the two amines and the d.r. was also measured with an achiral column (Agilent DB1704-60m-3d). We observed two peaks.

Derivatization was performed using benzoic anhydride/DMAP as follows: 57.5 mg of BA and 15.3 mg of DMAP were dissolved in 250 µL of THF. 20 µL of derivatization solution were added into 1 mL of reactions containing **3c** and shaken at 30 °C for 30 min. Next, 1mL of $NaHCO_3$ (10%) was added and the mixture was shaken for 30 min at 30 °C. Derivatization was confirmed by analysis with GC-MS.

12.3.2. Biocatalytic reaction to synthesize enantiopure **3d** on semi-preparative scale by combining ERed and IRed.

The final configuration of the amines that are produced with the IRED-20 and IRED-15 (methylammonium formate/methylamine buffer) were determined by NMR. Therefore, a biocatalytic reaction on semi-preparative scale was performed combining **3a** with OYE2 and either IRED-20 or IRED15, respectively.

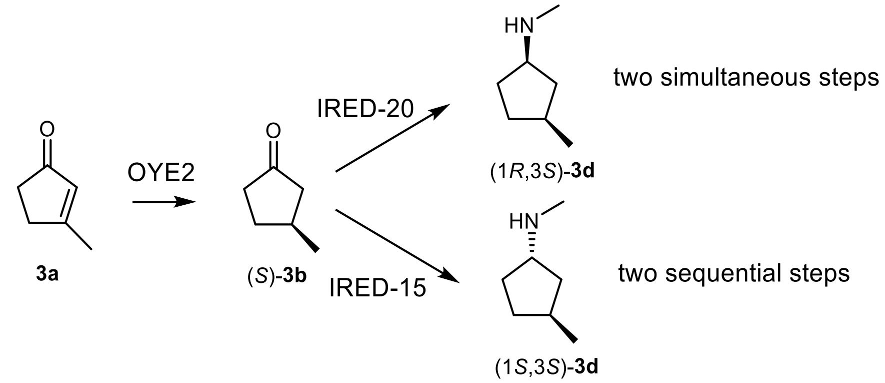

(1) Biocatalytic reactions

Reaction (A) – cascade reaction in one step: In a 50 mL falcon tube, a total volume of 50 mL consisted of: buffer (methylammonium formate/methylamine, 1M, pH 8; i.e., filled up to 50 mL after all the other reagents have been added), OYE2 (25 µM, 2.4 mL of 521 µM stock), IRED-20 (28 µM, 1.9 mL of 742 µM stock), FDH-QRN (10 µM, 509 µL of 983 µM stock), $NADP^+$ (0.5 mM, 20 mg) and substrate **3a** (52.6 mg, 10 mM, 96.13 g/mol, 0.971 mg/mL). The reaction was run at 30 °C for 24 h under horizontal shaking. A 50 µL sample was extracted after basifying with KOH (10M) and the conversion measured by GC with an achiral column (98% conversion).

Reaction (B) – cascade reaction in two steps: (i) step 1: In a 50 mL falcon tube, a total volume of 50 mL consisted of: buffer (methylammonium formate/methylamine, 1M, pH 8; i.e., filled up to 50 mL after all the other reagents have been added), OYE2 (25 µM, 2.4 mL of 521 µM stock), FDH-QRN (10 µM, 509 µL of 983 µM stock), $NADP^+$ (0.5 mM, 20 mg) and substrate **3a** (53.7 mg, 10 mM, 96.13 g/mol, 0.971 mg/mL). The reaction was run at 30 °C for 22 h. Then, a small sample was extracted, measured via GC, yielding full conversion and an *e.r.* of 99.6:0.4 for (*S*)-**3b**. (ii) step 2: IRED-15 (50 µM, 2.5 mL of 1000 µM), FDH-QRN (10 µM, 509 µL of 983 µM stock) and $NADP^+$ (0.5 mM, 20 mg) were added and the reaction incubated for further 22 h at 30 °C. A 50 µL sample was extracted after basifying with KOH (10M) and the conversion measured by achiral GC (>99 conversion).

Work-up of biocatalytic reactions: The reactions were basified with KOH (2 mL, 10 M), NaCl was added (big spatula tip), and the reactions were split in two falcon tubes and the organic compounds extracted with MTBE (each 2 x 15 mL and 1 x 5 mL). In between each extraction step, centrifugation was done for 10 min. The organic phases were combined and dried over anhydrous $MgSO_4$, filtered over cotton and kept at -20 °C until they were further acetylated.

A small sample was derivatized with benzoic anhydride and the d.r. measured with achiral GC-MS

GC: Reaction A: DB1701-60m-3d: amine peak 1 (51.53 min) 1.1%, amine peak 2 (51.65 min) 98.9%

GC: Reaction B: DB1701-60m-3d: amine peak 1 (51.47 min) 95.0% amine peak 2 (51.54 min) 5.0%

(2) Acetylation:

Reaction A: The acetylation was carried out using the same procedure as described in paragraph 12.1.4 using following reagents: **3c** (~61 mg in 60 mL MTBE, 0.54 mmol, 113.1 g/mol, ca. 9 mM), DMAP (10 mol%, 6.6 mg, 122.17 g/mol, 0.054 mmol), acetic anhydride (2.5 mL, 27 mmol, 102.1 g/mol) and triethylamine (ca. 5 eq., 376 µL, 2.7 mmol, 101.19 g/mol). GC-MS analysis showed that only 1 peak with a m/z of 155 was formed. After work-up, an orange solid was obtained (102 mg,) that was stored at -20 °C until further use.

Reaction B: The acetylation was carried out using the same procedure as described in paragraph 12.1.4 using following reagents: **3c** (~63 mg in 60 mL MTBE, 0.56 mmol, 113.1 g/mol, ca. 9 mM), DMAP (10 mol%, 6.6 mg, 122.17 g/mol, 0.054 mmol), acetic anhydride (2.5 mL, 27 mmol, 102.1 g/mol) and triethylamine (ca. 5 eq., 376 µL, 2.7 mmol, 101.19

g/mol). GC-MS analysis showed that only 1 peak with a m/z of 155 was formed. After work-up, a reddish solid was obtained (86 mg) that was stored at -20 °C until further use.

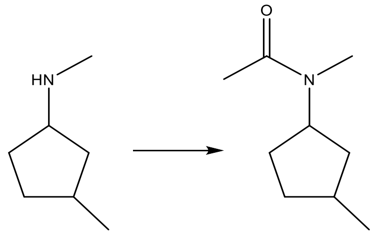

113.1 mg/mmol 155.1 mg/mmol

GC-FID analysis of both reactions showed the presence of traces of acetic anhydride (6% for reaction A and 10% for reaction B). This was confirmed with GC-MS.

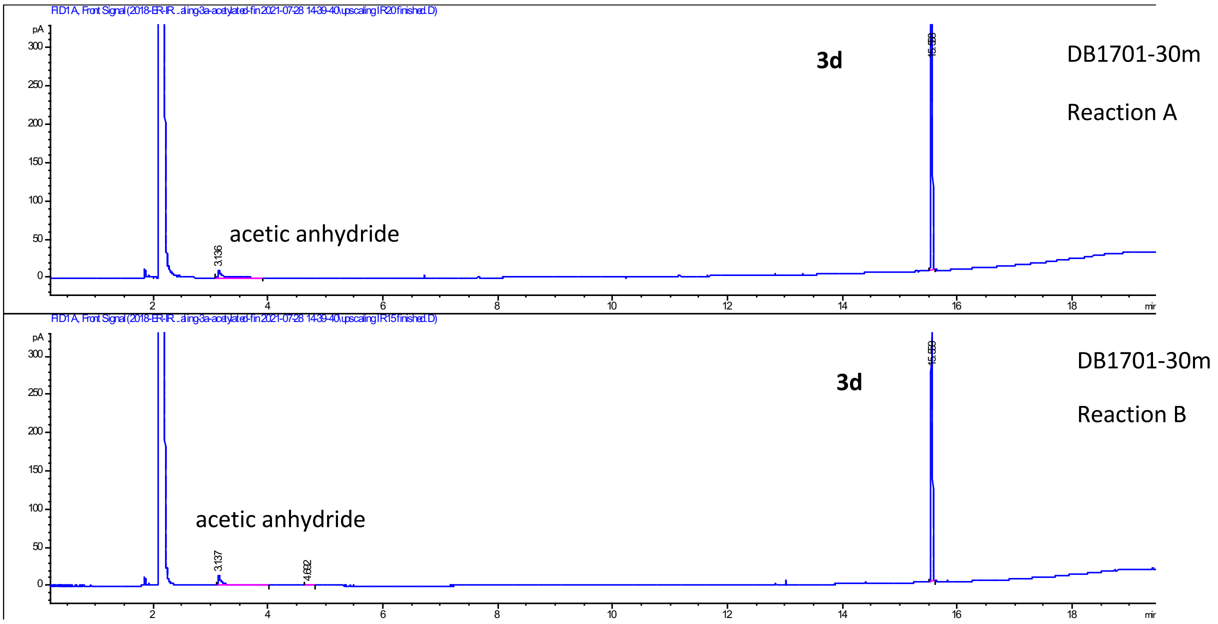

(3) Products purification by column chromatography:

A preliminary TLC plate analysis (eluent pure DCM on a silica plate, Figure S7A) showed that the desired product is more retained by the silica than the impurity acetic anhydride.

Column conditions: Column diameter: 1 cm; Packing: SiO_2 60 for column chromatography with pure DCM; SiO_2 bed height: 10 cm; Fraction collections: ca. 8 mL each.

Reaction A (IRED-20): The sample was dissolved in a small volume of DCM and loaded onto the column. Initial elution was done with DCM until 15 fractions were collected. TCL analysis showed that the compound hasn't been eluting. Thus, elution was continued with DCM/MeOH 9:1 for further 5 fractions (fraction 17 had a yellowish colour) and TLC analysis revealed that the compound was eluting in fraction 17 (Figure S7B). GC-FID identified the product with a ratio product/acetic anhydride ca. 98.5/1.5. After removing of the solvent under reduced pressure, a mass of 67.1 mg was obtained (80%).

Reaction B (IRED20): The same procedure as for reaction A was repeated. The product eluted again in fraction 17 and was obtained with 41.6 mg (48%) with an apparent area ratio product/acetic anhydride 98/2.

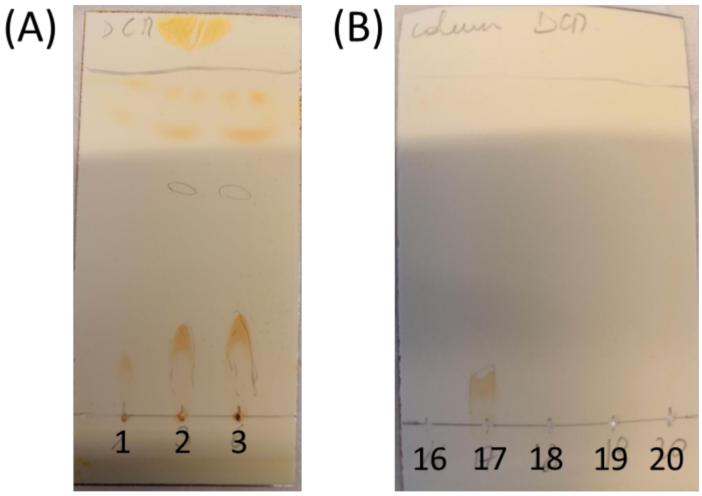

Figure S7. TCL analysis of Reaction A during column chromatography. Eluent pure DCM on a silica plate. (A) Product before column chromatography. (B) Elution fractions during column chromatography (fractions 16 to 20 were spotted).

*12.3.3. NMR measurements of enantiopure **3d** produced by biocatalytic reaction on semi-preparative scale*

(1) The synthetic strategy and conclusions of NOESY analysis

First, the two diastereomers of the **3d** product were synthesized as *N*-acetylated derivatives following the same chemo-enzymatic strategy illustrated in Fig. S6 by starting from **3a** (see SI 12.3.2 for details) The initial enzymatic conversions of **3a** (53 and 54 mg-scale, respectively) catalyzed by OYE2 and either IRED-20 or IRED-15 led to enantiomerically pure **3d** products in 98% and >99% conversion, respectively. Follow-up chemical acetylation and methylation yielded the desired final products in 67 mg (79% overall yield from **3a**) and 42 mg (49% overall yield from **3a**), respectively. NOESY experiments conducted on the enantiomerically pure *N*-acetylated **3d** products revealed a detectable NOE signal between the methyl group of CH3-N and the methyl group substituent at C-3 for the enantiomer product obtained in the cascade with OYE2 and IRED-20 (Fig. S8A). Therefore, this indicates that CH_3-N and CH_3-C substituents are in *cis*-configuration and the product was assigned to be (1*R*,3*S*)-**3d**. In contrast, the NOE signal was not detected for the diastereomer product obtained in the cascade with OYE2 and IRED-15, thus indicating that the two methyl substituents are too far away (Fig. S8B). This enantiomer (a *trans*) was assigned to be (1*S*,3*S*)-**3d**.

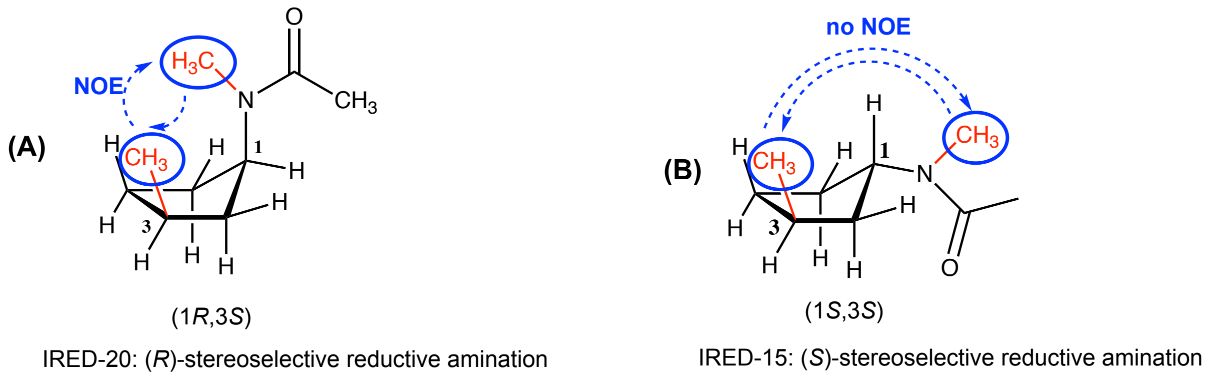

Figure S8. Schematic depiction of the NOE signals analysis for the determination of the absolute configuration of the diastereomer products of **3d** obtained from the cascade reaction combining OYE2 with either IRED-20 or IRED-15. (A) Observed NOE signal for the product obtained with IRED-20. (B) Lack of NOE signal for the product obtained with IRED-15.

(2) Analysis of 2D NMRs, COSY and HSQC

NMRs (^{1}H and ^{13}C) measured in $CDCl_3$ at room temperature (27 °C) looked complex because all the signals are split in two patterns. The reason for that is the possibility to have two conformations for the acetyl group of the amide that are not converting quickly at room temperature.

In fact, the NMRs spectra became simpler when the sample was dissolved in DMSO and were recorded at higher temperatures. At the temperature of 120 °C, one signal for each ^{1}H or ^{13}C was observed, thus demonstrating that the system was dynamic and that the two possible conformations quickly convert above 100 °C.

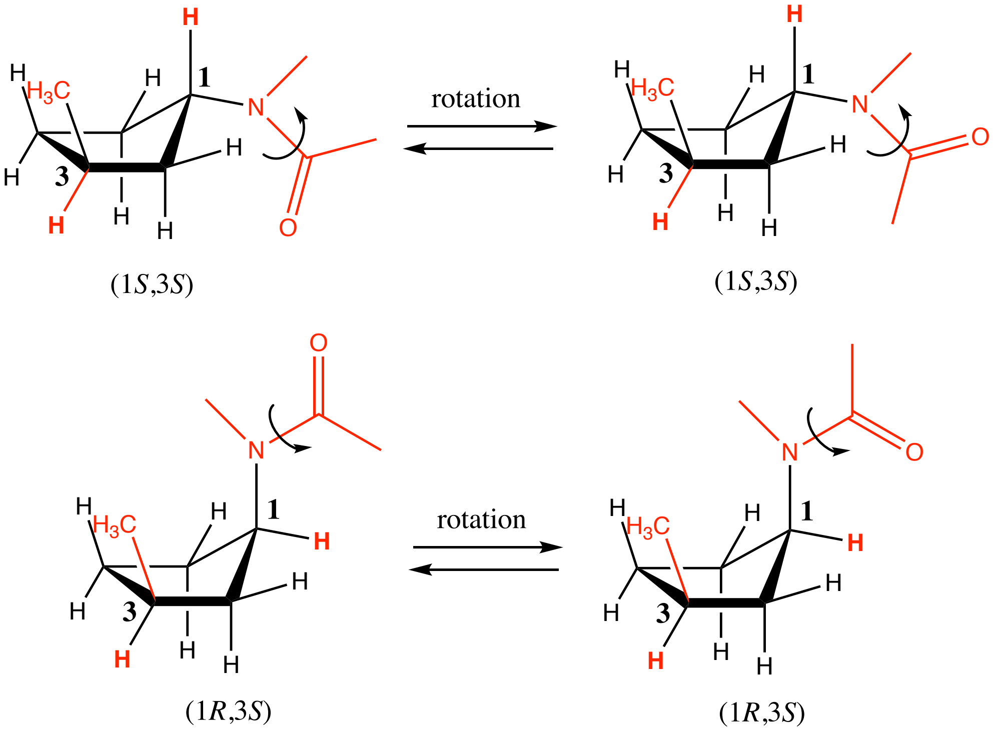

Reaction A (with IRED-20)

^{1}H-NMR (solvent $CDCl_3$) at 27 °C

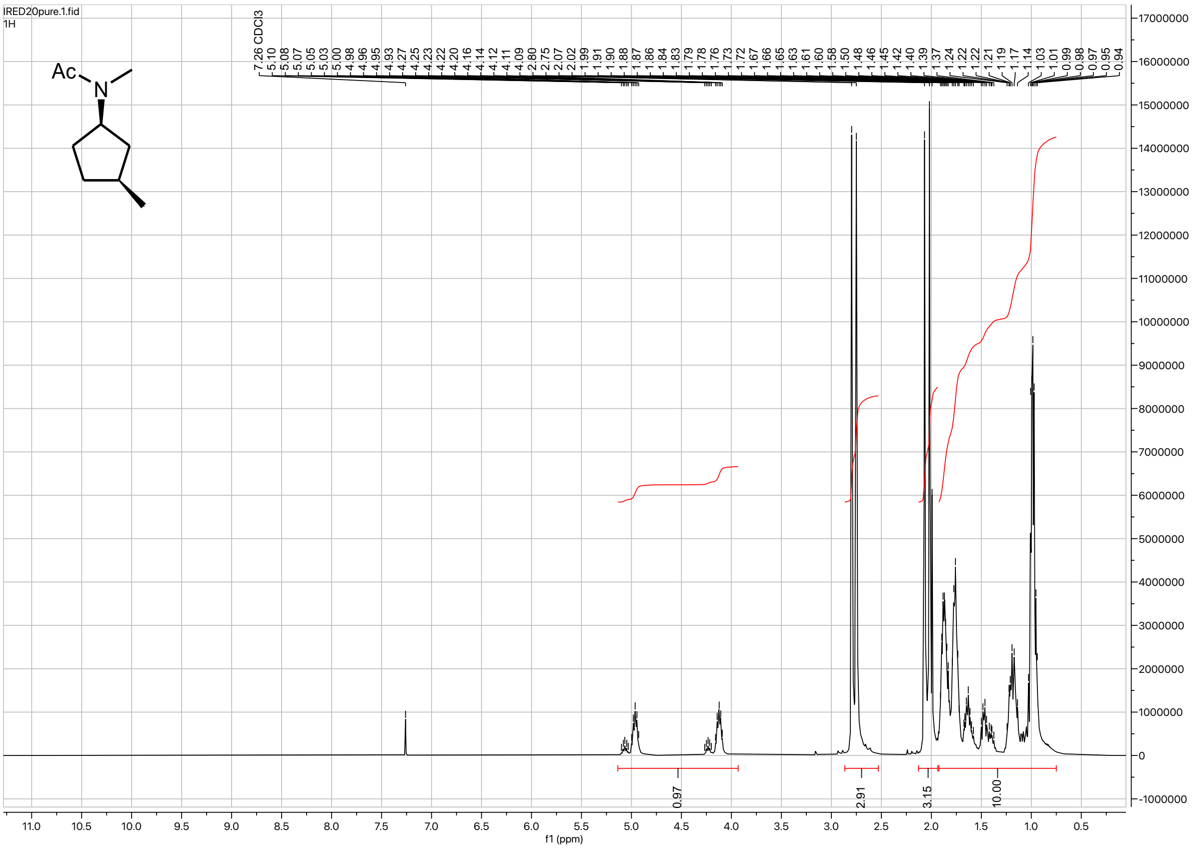

^{1}H-NMR (solvent DMSO-d6) at 27 °C

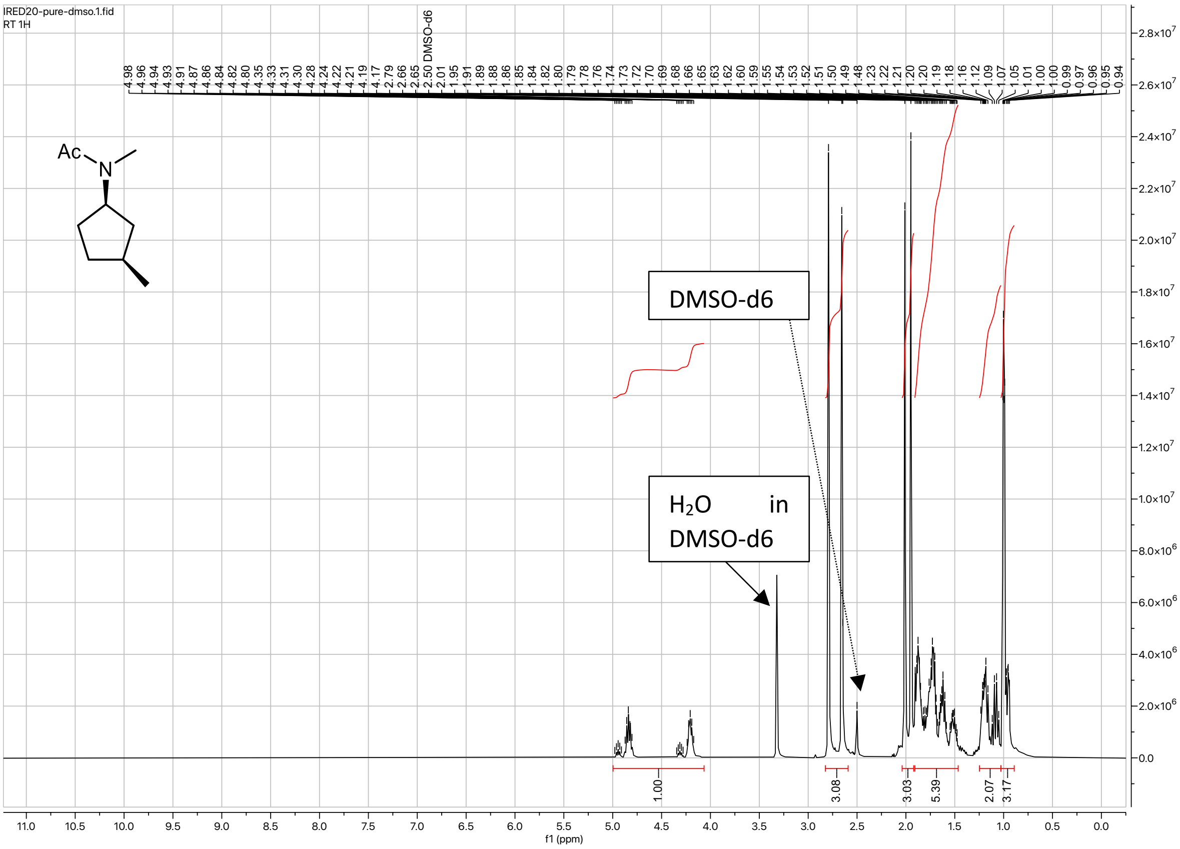

^{1}**H-NMR** (solvent DMSO-d6) at 120 °C

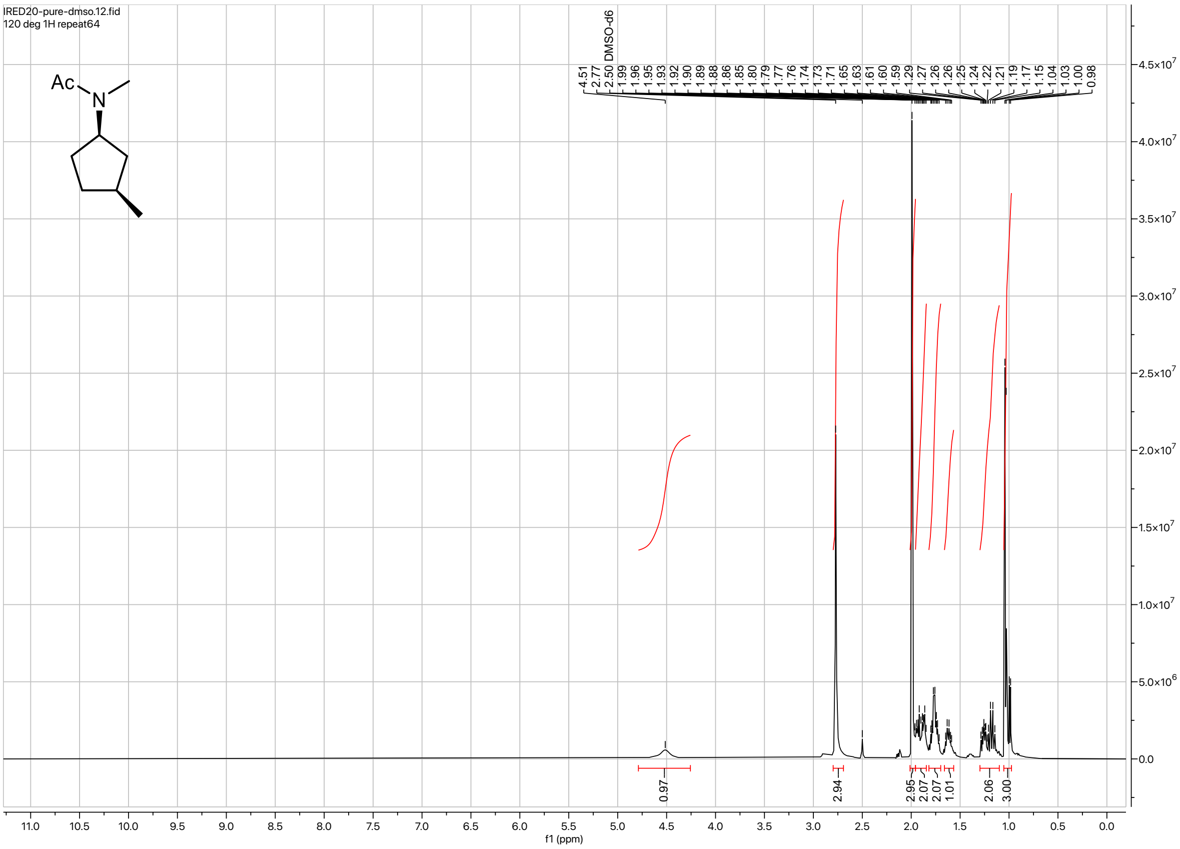

Expansion 0.5–5 ppm of ^{1}**H-NMR** (solvent DMSO-d6) at 120 °C

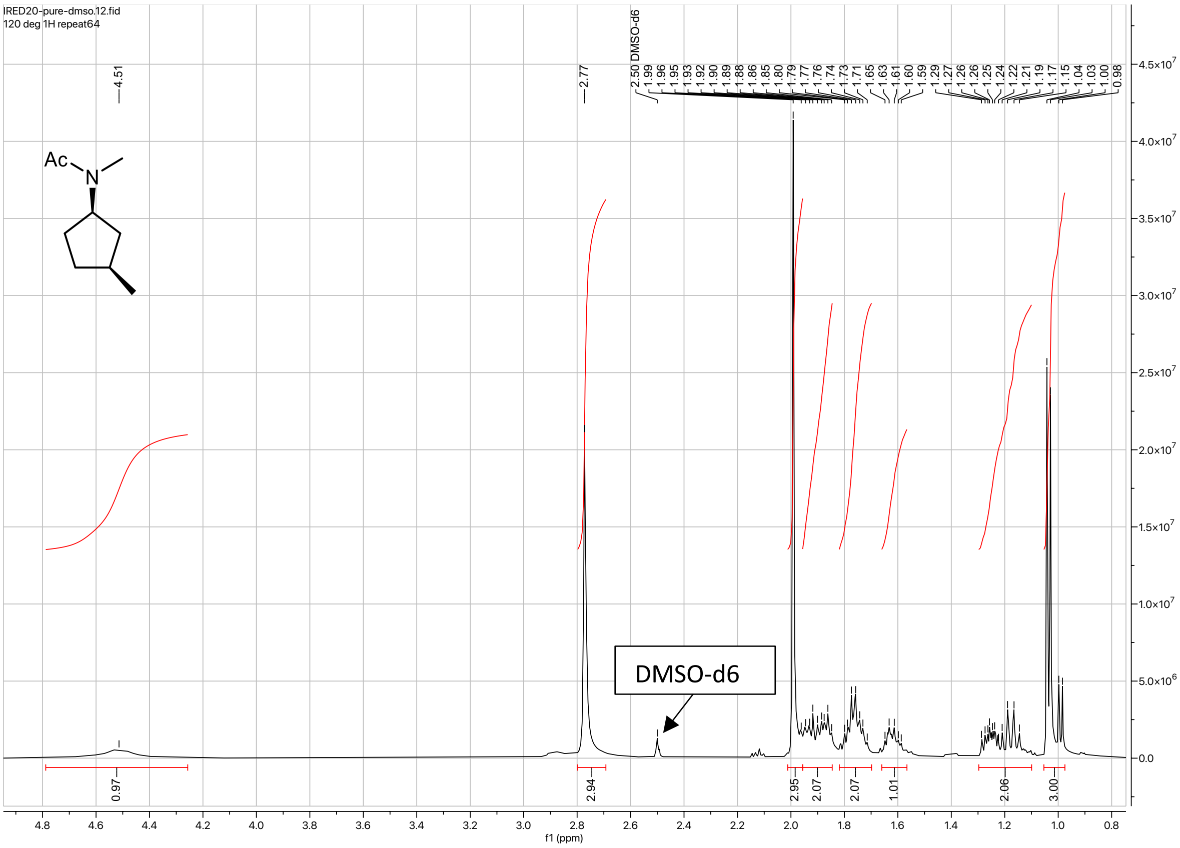

^{13}C-NMR (solvent DMSO-d6) at 120 °C

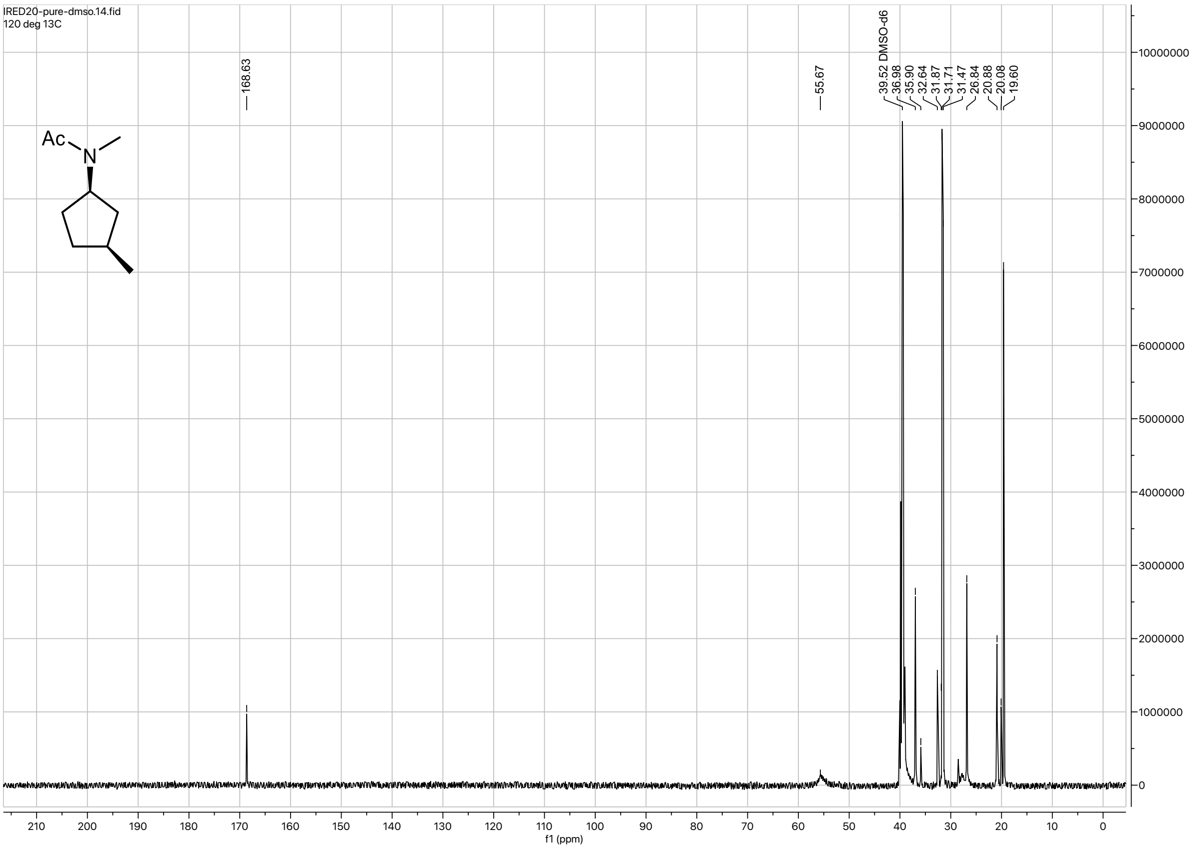

Reaction B (with IRED-15)

^{1}H-NMR (solvent $CDCl_3$) at 27 °C

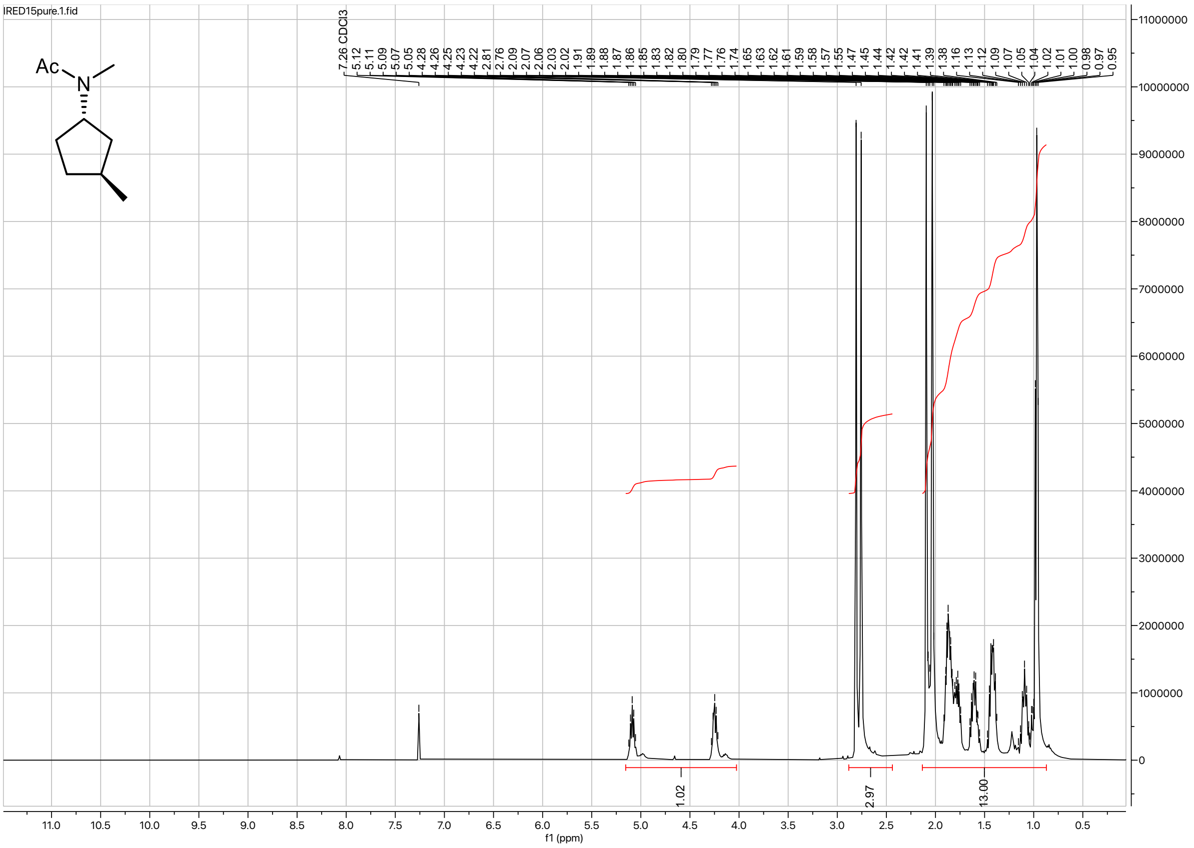

^{1}**H-NMR** (solvent DMSO-d6) at 27 °C

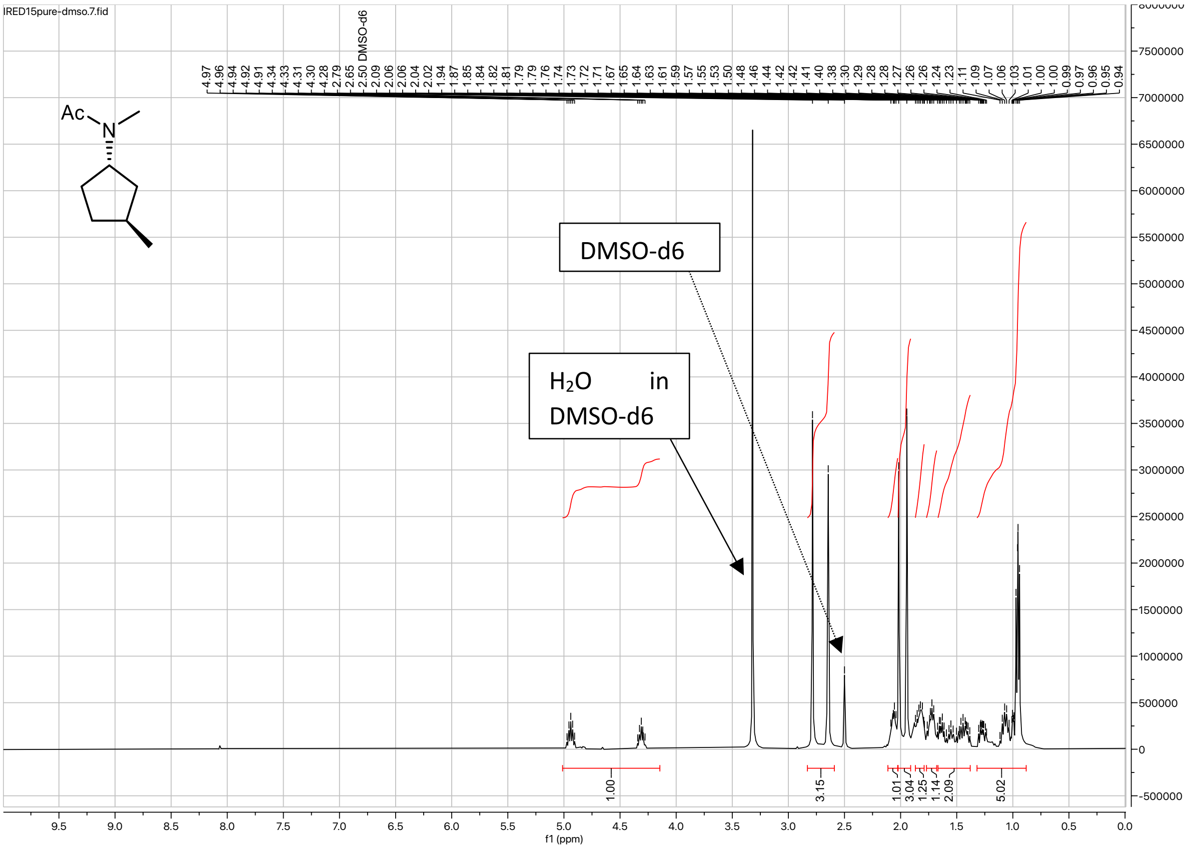

^{1}**H-NMR** (solvent DMSO-d6) at 120 °C

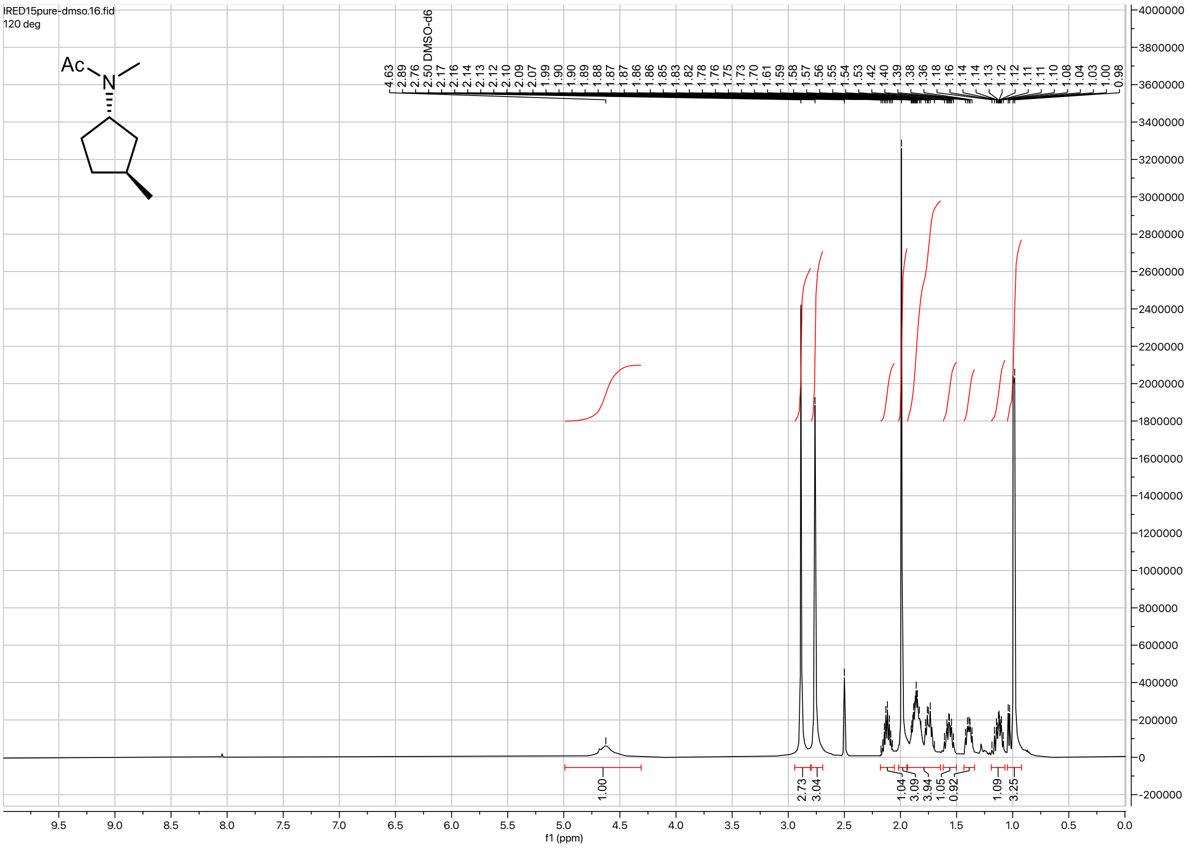

Expansion 0.5–5 ppm of ^{1}**H-NMR** (solvent DMSO-d6) at 120 °C

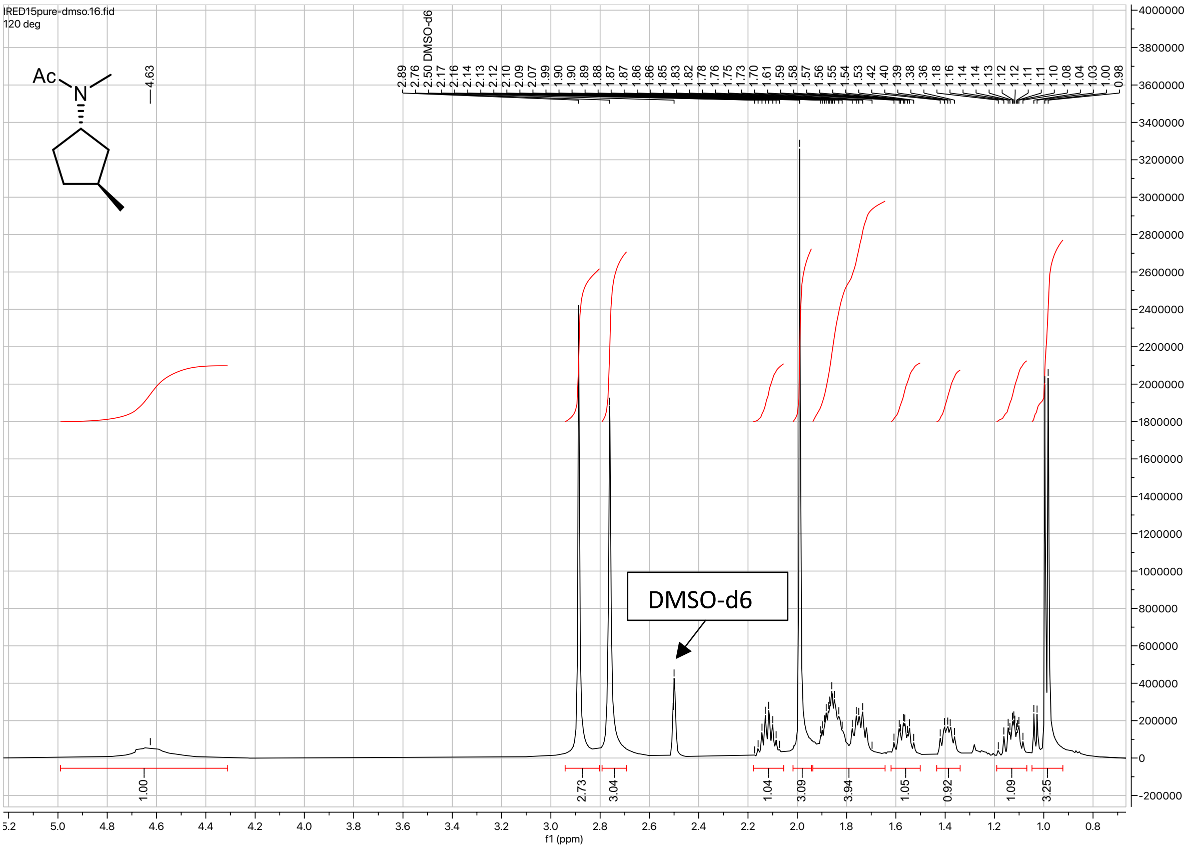

^{13}**C-NMR** (solvent DMSO-d6) at 120 °C

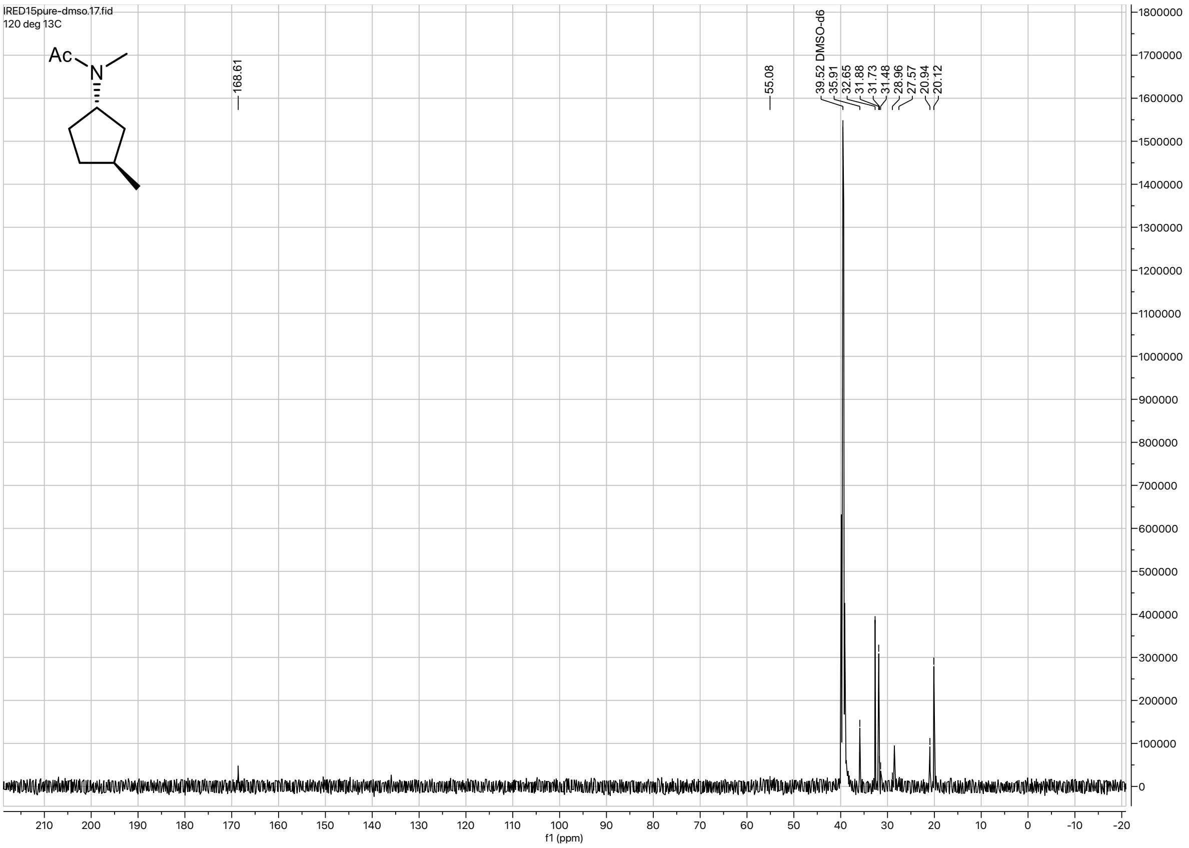

(3) NOESY experiments to assign the absolute configuration

General comment: it is known that OYE2 installs a stereogenic center at carbon-3 in (*S*) absolute configuration, after the asymmetric C=C double bond reaction.

After the subsequent reductive amination at the carbonyl of carbon-1, it is reasonable to assume that the *cis* diastereomer (i.e., (1*S*,3*S*)) will assume a more favorable conformation in which both the CH_3- and the bulky (CH_3NCOCH_3)- groups will be in the position as depicted below.

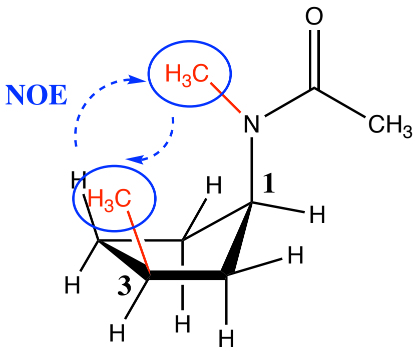

(1*R*,3*S*)

By doing NOESY NMR experiments using the samples in $CDCl_3$ at room temperature, the sample obtained after column chromatography from Reaction A (IRED-20, paragraph 12.3.2) gave a strong NOE signal between the two methyl groups highlighted in red. Notably, if one irradiates the CH_3-C of the ring, a NOE is observed on the CH_3-N. Vice versa, irradiating the CH_3-N, a NOE is observed on the CH_3-C. Such strong NOE signals tell us that the two methyl groups are close in the space. Such a space proximity is only possible if the two methyl groups are located on the same face of the 5-membered ring. **Therefore, IRED-20 produced the (*cis*) stereoisomer (1*R*,3*S*)-3d.**

In the case of the *trans* diastereomer (i.e., (1*R*, 2*S*)), the following structure can be drawn:

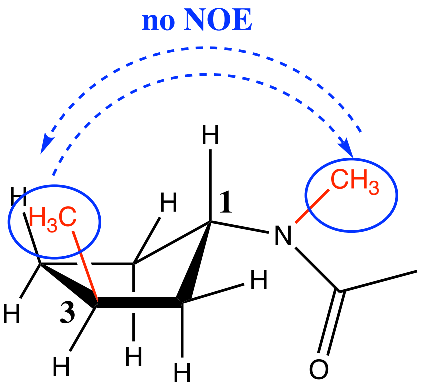

(1*S*,3*S*)

The two methyl groups are located on opposite faces of the 5-membered ring; therefore, they are far from each other. By doing NOESY NMR experiments using the samples in $CDCl_3$ at room temperature, the sample obtained after column chromatography from Reaction B (IRED15, paragraph 12.3.2) gave a very weak NOE signal between the two methyl groups highlighted in red (order of magnitudes lower than in the previous case). **Therefore, IRED-15 produced the (*trans*) stereoisomer (1*S*,3*S*)-3d.**

Additional note: It is interesting to note that both samples produced by either IRED-20 or IRED-15 gave a measurable NOE signal between the CH_3-C of the ring and the CH_3-CO of the acetyl group. Such NOEs can be explained as below:

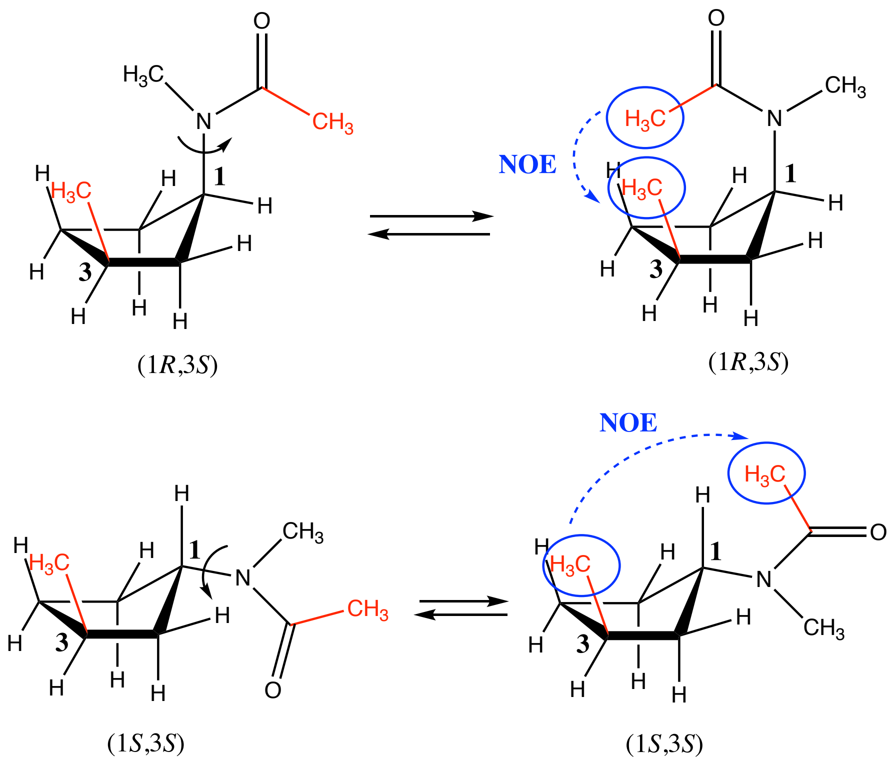

^{1}H-NOESY (1D selective gradient NOESY frequency 2.8 ppm)

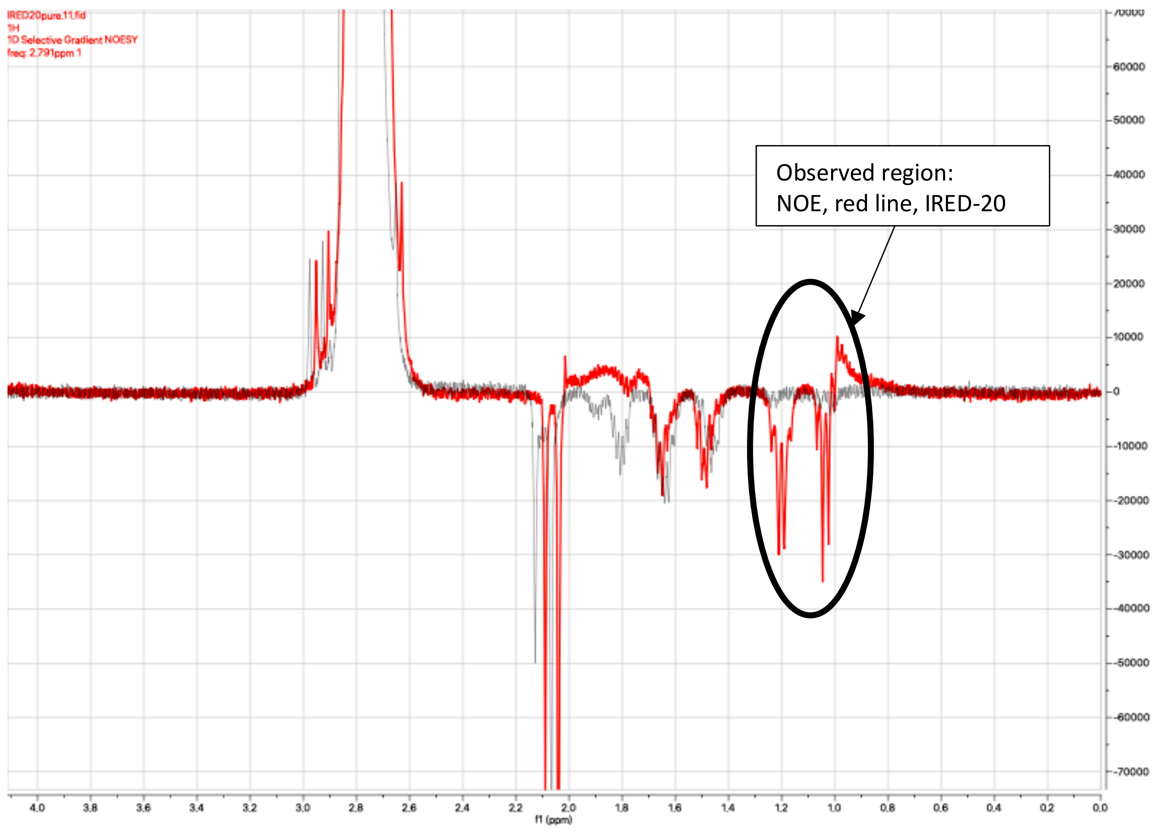

Legend: Red line (selective NOESY NMR of the product made with IRED-20)

Grey line (selective NOESY NMR of the product made with IRED-15)

^{1}H-NOESY (1D selective gradient NOESY frequency 1.0 ppm)

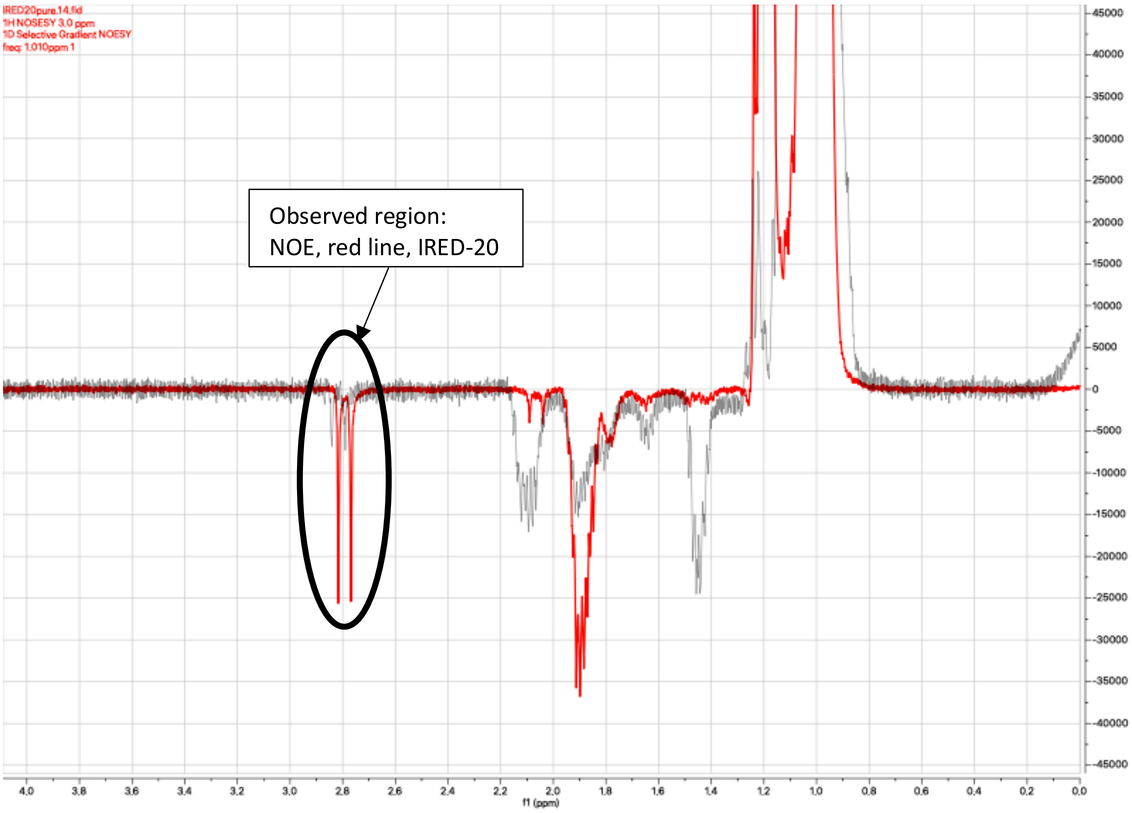

Legend: Red line (selective NOESY NMR of the product made with IRED-20)

Grey line (selective NOESY NMR of the product made with IRED-15)

12.4. *Substrate 4a-d*

12.4.1. *Reference for **4b** - Identification of the enantiomers using optical rotation*

The publication from Baker et al, [31] describes the optical rotation for (*S*)-**4b** (+6.5° c 1.5) and (*R*)-**4b** (-5.9° c 1.4). Therefore, a biocatalytic reaction combining **4a** and YqjM was made on semi-preparative scale.

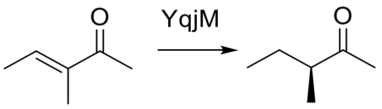

4a 4b

(*S*)-**4b** (*e.r.* = >97:<3)

(1) Biocatalytic reaction: In a 50 mL falcon tube, following reagents were pipetted (total volume 40 mL): buffer (KPi, 50 mM, pH 8.0, 38 mL), YqjM (13 µM, 950 µL of 574 µM stock solution), FDH-QRN (7 µM, 300 µL of 958 µM), $NADP^+$ (0.25 mM, 10 mg, 765.5 g/mol), sodium formate (38 mM, 103 mg, 68.01 g/mol) and substrate **4a** (~10 mM, 61 mg, 98.15 g/mol). The reaction was incubated at 30 °C for 21 h on a horizontal shaker. After that, the conversion of a small sample was verified by GC-FID (>99% conversion, 97% yield, *e.r.* >97:<3).

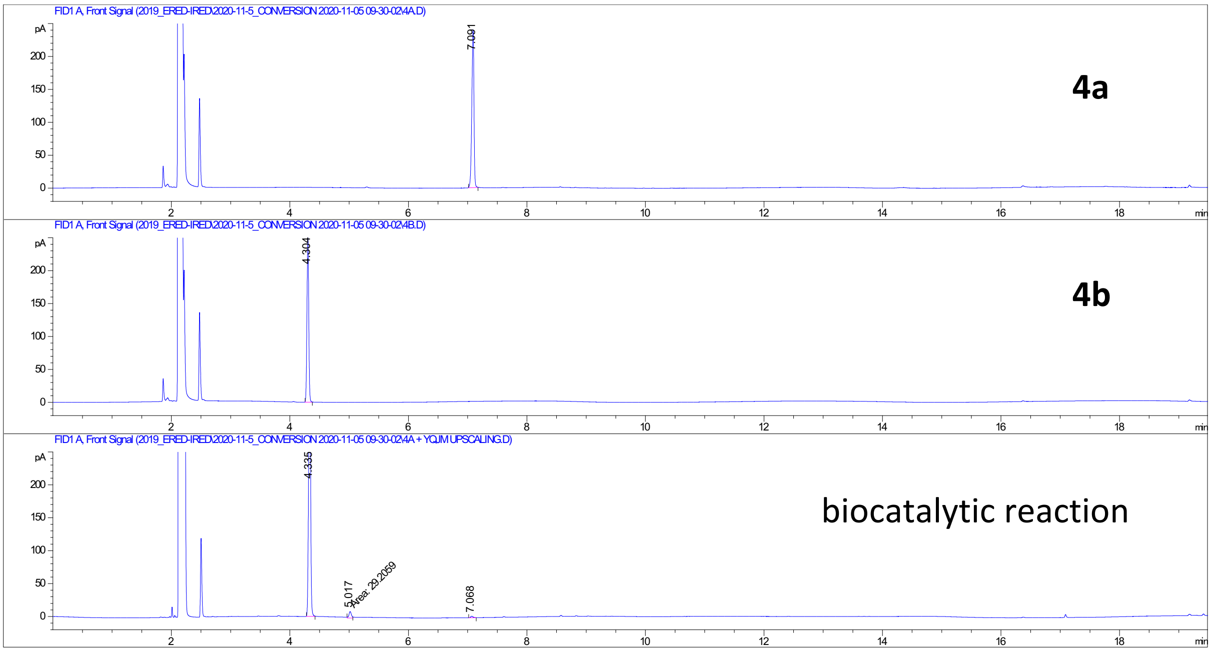

(2) Work-up: Due to the volatility of **4b**, removal of the solvent is challenging. Therefore, extraction was performed once with a minimum amount of DCM (4 mL). A spatula tip of NaCl was also added. The reaction was stirred for 10 min, then centrifuged for 10 min. Approximately 3 mL of the organic phase could be recovered which was dried over $MgSO_4$.The concentration of **4b** was measured via GC-FID using toluene as internal standard and resulted in approximately 16.1 mg/mL (48 mg/mL, 78% yield, *e.r.* >97:<3)

Chiral GC-FID chromatograms:

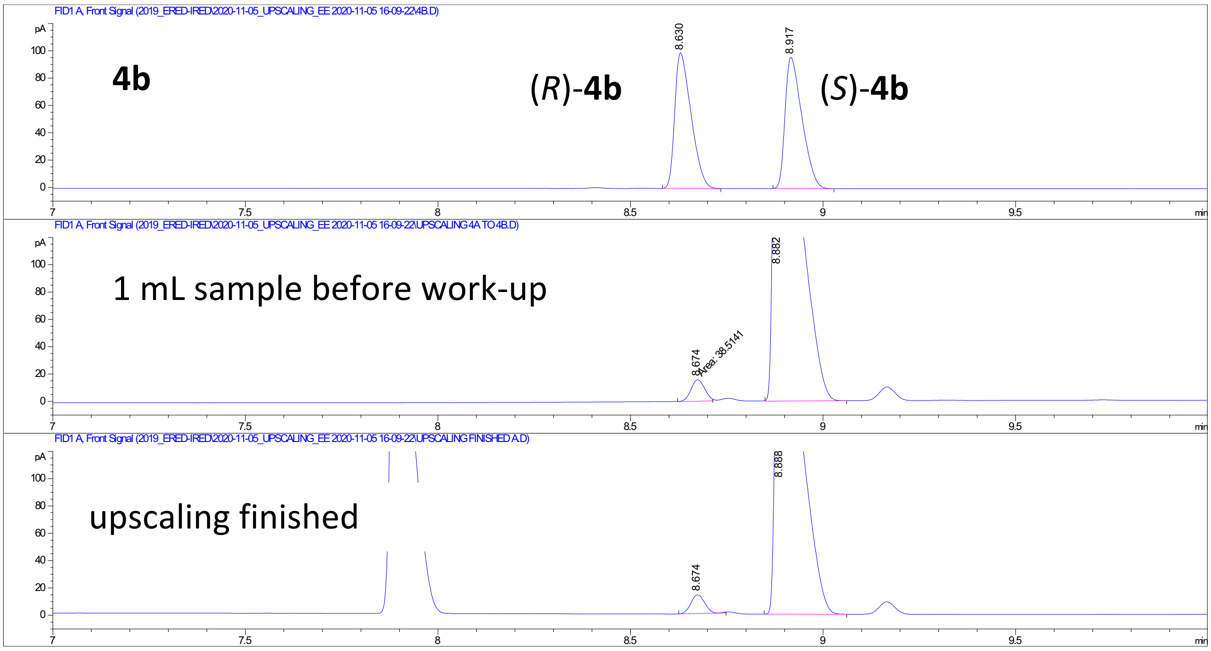

(3) Optical rotation: The optical rotation was measured directly after the work-up in a 5 x 50 mm glass/PTFE tube on a WZZ-2S polarimeter (W&J Instrument) and a α_{obs} value of 0.216 was measured. By using

Equation 1, an $[\alpha]^{20}_{D}$ = +26.8 measured at the concentration of 1.6 g dL^{-1} was calculated which means YqjM produces (*S*)-**4b**.

Equation 1 $$[\alpha]_D^T = \frac{\alpha_{obs}}{c * l}$$

α_{obs} observed optical rotation (0.216
c the concentration of the solution in grams per milliliter (0.0161)
l the length of the tube in decimeters (0.5)
T Temperature of measurement

12.4.2. References for **4c** with ωTAs on analytical scale

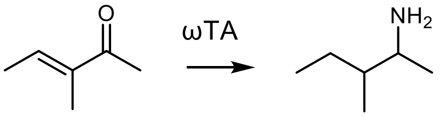

rac-**4b** **4c**

Table S57. Conversion (%) for substrate **4a** or **4b** using ωTAs and an ERED in combination with ωTAs for obtaining the four stereoisomers of the primary amine **4c**. (GC measurement with an achiral column)

substrate	ERed	ωTA	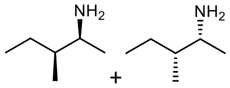	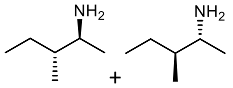	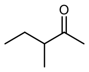	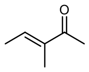
			(2*S*,3*S*)-**4c** + (2*R*,3*R*)-**4c**	(2*S*,3*R*)-**4c** + (2*R*,3*S*)-**4c**	**4b**	**4a**
**4a**	XenA[(a)]	-			>99	n.d.
**4b**	-	Vf-ωTA (S)	9	14	77	n.d.
**4b**	-	As(R)-ωTA (R)	14	14	72	n.d.
**4b**	-	Cv-ωTA (S)	11	26	63	n.d.
**4a**	XenA	Vf-ωTA (S)	15	n.d.	85	n.d.
**4a**	XenA	As(R)-ωTA (R)	<1	22	78	n.d.
**4a**	XenA	Cv-ωTA (S)	10.5	n.d.	90	n.d.

(a) *e.r.* >99:<1 (*S*)-**4b**

Table S58. Diastereomeric and enantiomeric composition (%) for **4c** (GC measurement with chiral column) after derivatization to the acetamide.

substrate	ERED	ωTA	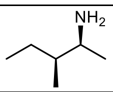	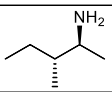	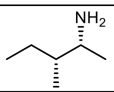	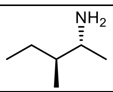
			(2*S*,3*S*)-**4c**	(2*S*,3*R*)-**4c**	(2*R*,3*R*)-**4c**	(2*R*,3*S*)-**4c**
**4b**	-	Vf-ωTA (S)	40.4	58.6	n.d.	1.0
**4b**		As(R)-ωTA (R)	n.d.	n.d.	53.5	46.5
**4b**	-	Cv-ωTA (S)	29.8	69.6	n.d.	0.6
**4a**	XenA	Vf-ωTA (S)	98.7	n.d.	n.d.	1.3
**4a**	XenA	As(R)-ωTA (R)	n.d.	n.d.	1.5	98.5
**4a**	XenA	Cv-ωTA (S)	97.3	n.d.	n.d.	2.7

By using the known stereoselectivity of the transaminases on this type of substrate, the peak annotation could be performed.

GC-FID chromatograms for the separation of the **4c** isomers from ωTA reactions:

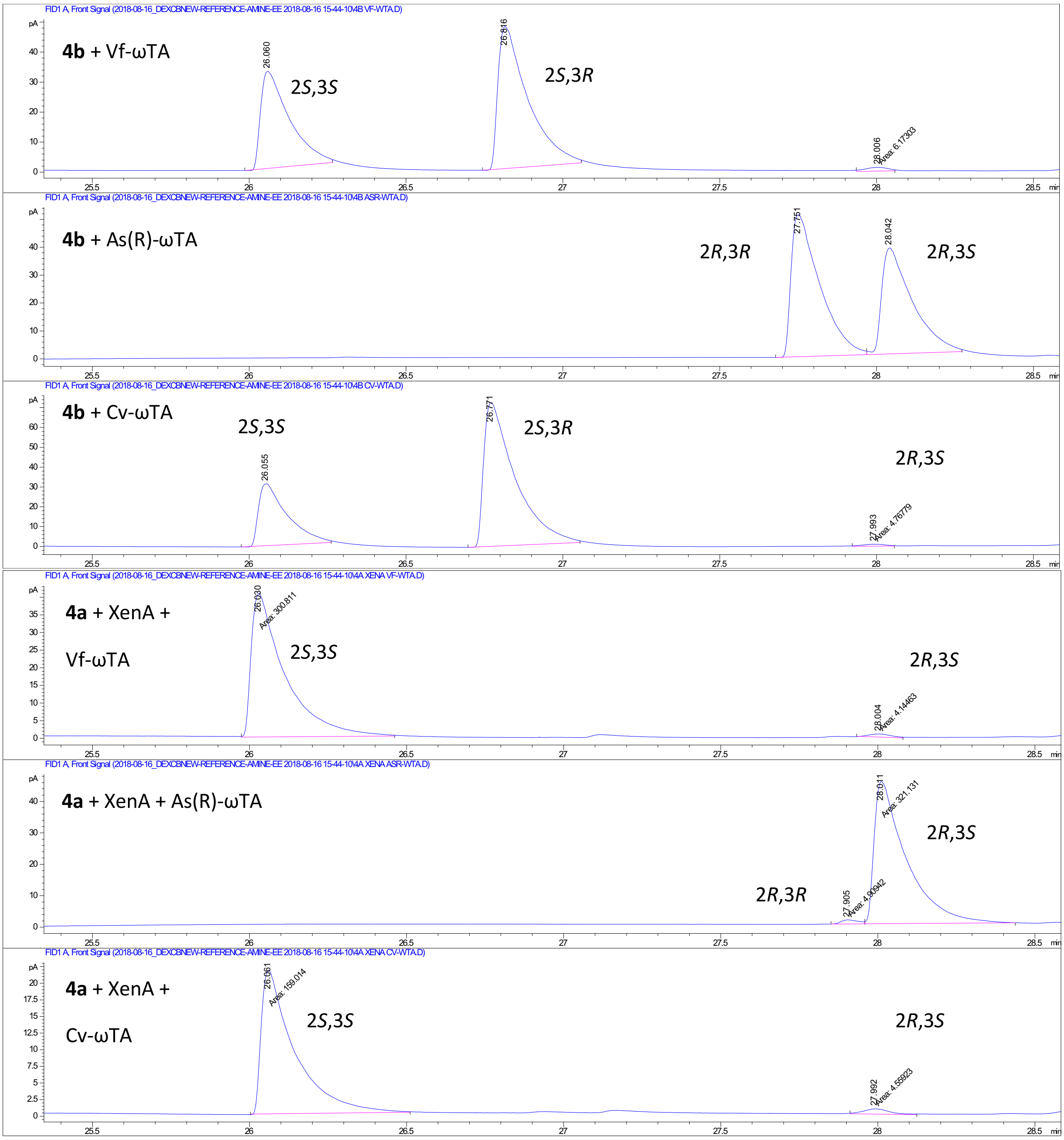

12.4.3. References for **4c** with ωTAs on semi-preparative scale

Commercially available references for **4c** and **4d** are not available. To keep the *e.r.* of the saturated intermediate high, the reaction will be performed in two sequential steps when ERed and ωTA are combined.

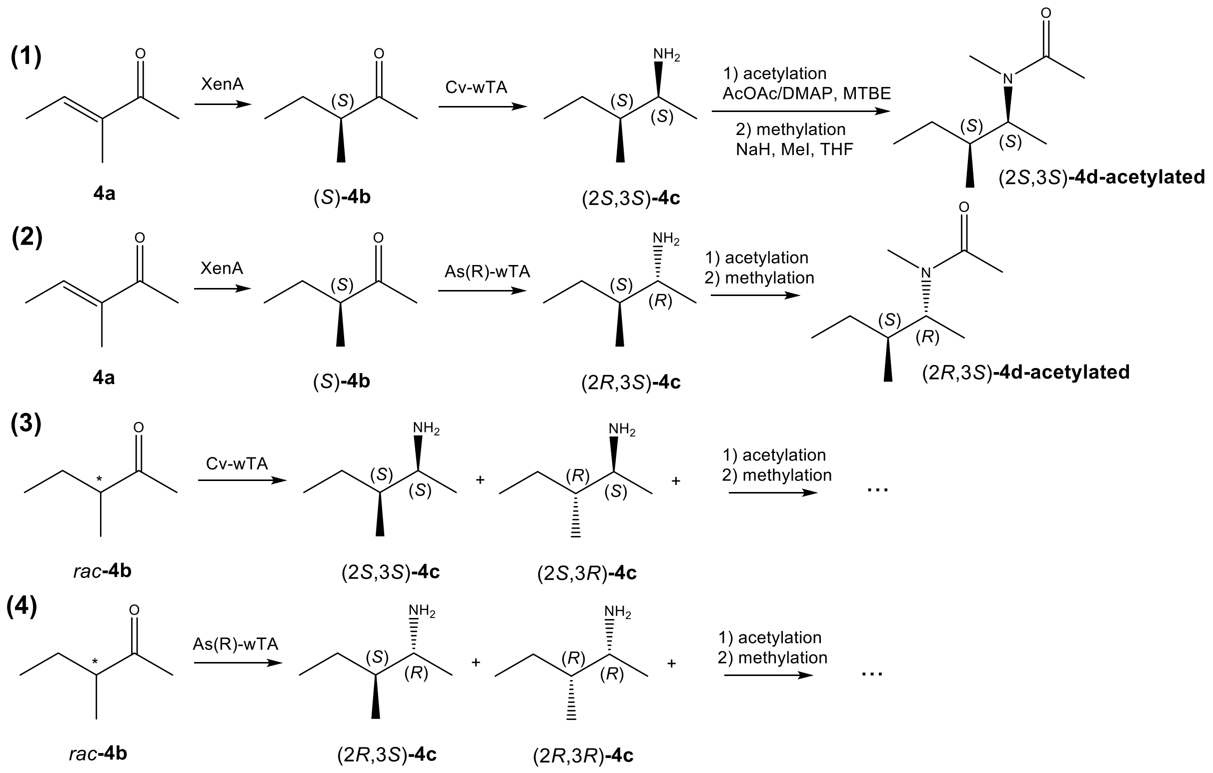

(1a) Biocatalytic reaction combining ERed and ωTA: (i) ERed reaction: In a 15 mL falcon tube, a total volume of 10 mL consisted of: buffer (KPi, 9.53 mL, 50 mM, pH 7), XenA (12 µM, 200 µL of 574 µM stock), Cb-FDH (5 µM, 56 µL of 909 µM stock), NAD^+ (0.25 mM, 100 µL of 25 mM stock), sodium formate (3 equiv., 30 mM, 100 µL of 3 M stock, 68 g/mol) and substrate **4a** (10 mM, 10 mg, 98.15 g/mol, 0.88 g/mL). The reaction was incubated at 30 °C on a horizontal shaker for 22 h. After measuring conversion (>99%) and *e.r.* (99:1 for (*S*)-**4b**), the reagents for the ωTA reaction were added. (ii) amination: Cv-ωTA or As(R)-ωTA (120 mg, as lyophilized cells), D- or L-alanine (5 equiv., 50 mM, 2 mL of 250 mM stock), PLP (1 mM, 265.16 g/mol), LDH-101 (1 mg/mL, 10 mg), Cb-FDH (10 µM, 110 µL of 909 µM stock), NAD^+ (1 mM, 400 µL of 25 mM stock), sodium formate (3 equiv., 30 mM, 100 µL of 3 M stock, 68 g/mol). The reaction mixture was incubated for further 22 h at 30 °C. A small sample was taken for measuring the conversion and the d.r. (after derivatization with DMAP/acetic anhydride), before the work-up was done.

(1b) Biocatalytic reaction with *rac*-4b and ωTA: In a 15 mL falcon tube, a total volume of 4 mL consisted of: buffer (KPi, 1.17 mL, 50 mM, pH 7), Cv-ωTA or As(R)-ωTA (30 mg/mL, 120 mg, as lyophilized cells), D- or L-alanine (5 equiv., 250 mM, 2 mL of 500 mM stock), PLP (1 mM, 320 µL of 12.5 mM stock, 265.16 g/mol), LDH-101 (1 mg/mL, 4 mg), Cb-FDH (10 µM, 44 µL of 909 µM stock), NAD^+ (1 mM, 160 µL of 25 mM stock), sodium formate (3 equiv., 150 mM, 200 µL of 3 M stock, 68 g/mol) and substrate *rac*-**4b** (50 mM, 20 mg, 100.16 g/mol; 0.815 mg/mL). The reaction mixture was incubated for 22 h at 30 °C. A small sample was taken for measuring the conversion and the d.r. (after derivatization with DMAP/acetic anhydride), before the work-up was done.

(2) Work-up of biocatalytic reactions: The tubes were centrifuged for 15 min and the supernatant transferred into new falcon tubes. After acidic extraction (addition of HCl, 3 N, 1.5 mL) with MTBE (1 x 10 mL for the ERed-ωTA reaction and 1 x 5 mL for the ωTA reaction), the organic layer was basified with KOH (10 M, 1 mL) and the organic compounds extracted with MTBE (1 x 5 mL for the ERED-ωTA reaction and 1x3 mL for the ωTA reaction). After drying the combined organic phases over anhydrous $MgSO_4$, it was kept at -20 °C without removing the solvent. Comment: after work-up, there was still intermediate detected.

Table S59. Conversion (%) for substrate **4a** and **4b** using ωTAs and an ERed in combination with ωTAs for obtaining the four stereoisomers of the primary amine **4c**. (GC measurement with an achiral column)

	substrate	ERed	ωTA	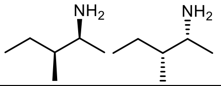	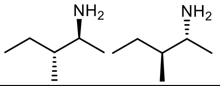	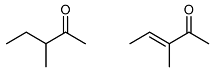	
				(2*S*,3*S*)-**4c** + (2*R*,3*R*)-**4c**	(2*S*,3*R*)-**4c** + (2*R*,3*S*)-**4c**	**4b**	**4a**
before work-up	**4a**	XenA	Cv-ωTA (S)	36	5	59	n.d.
	**4a**	XenA	As(R)-ωTA (S)	1	59	40	n.d.
	**4b**	-	Cv-ωTA (S)	35	41	24	n.d.
	**4b**	-	As(R)-ωTA (S)	41	45	14	n.d.
after work-up	**4a**	XenA	Cv-ωTA (S)	56	7	37	n.d.
	**4a**	XenA	As(R)-ωTA (S)	2	81	17	n.d.
	**4b**	-	Cv-ωTA (S)	45	51	4	n.d.
	**4b**	-	As(R)-ωTA (S)	47	51	2	n.d.

Table S60. Diastereomeric and enantiomeric composition (%) for **4c** (GC measurement with a chiral column) after derivatization to the acetamide.

substrate	ERed	ωTA	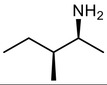	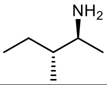	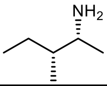	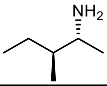
			(2*S*,3*S*)-**4c**	(2*S*,3*R*)-**4c**	(2*R*,3*R*)-**4c**	(2*R*,3*S*)-**4c**
**4a**	XenA	Cv-ωTA (S)	96.5	3.5	n.d.	n.d.
**4a**	XenA	As(R)-ωTA (S)	n.d.	n.d.	1.7	98.3
**4b**	-	Cv-ωTA (S)	48.3	51.5	0.1	n.d.
**4b**	-	As(R)-ωTA (S)	0.3	n.d.	48.5	51.2

(3) Acetylation: To the reactions obtained after the work-up, a solution of DMAP in acetic anhydride (50 mg/mL, 1 equiv. DMAP) was added; the reaction was incubated at 30 °C at room temperature under stirring. A small aliquot was taken, and acetylation was verified via GC-MS to be quantitative.

(4) Work-up acetylation: The organic phase was acidified (HCl, 3 N, 7.5 mL and 4 mL for the two-enzyme and one-enzyme reaction, respectively) and stirred for 10 min. After centrifugation for 10 min, the organic phase was then basified (K_2CO_3, saturated solution, 7.5 mL and 4 mL for the two-enzyme and one-enzyme reaction, respectively), stirred for 10 min and centrifuged. After drying the combined organic phases over anhydrous $MgSO_4$, the solvent was removed under reduced pressure.

Chiral GC-FID chromatograms for the separation of the **4d** isomers from ωTA reactions:

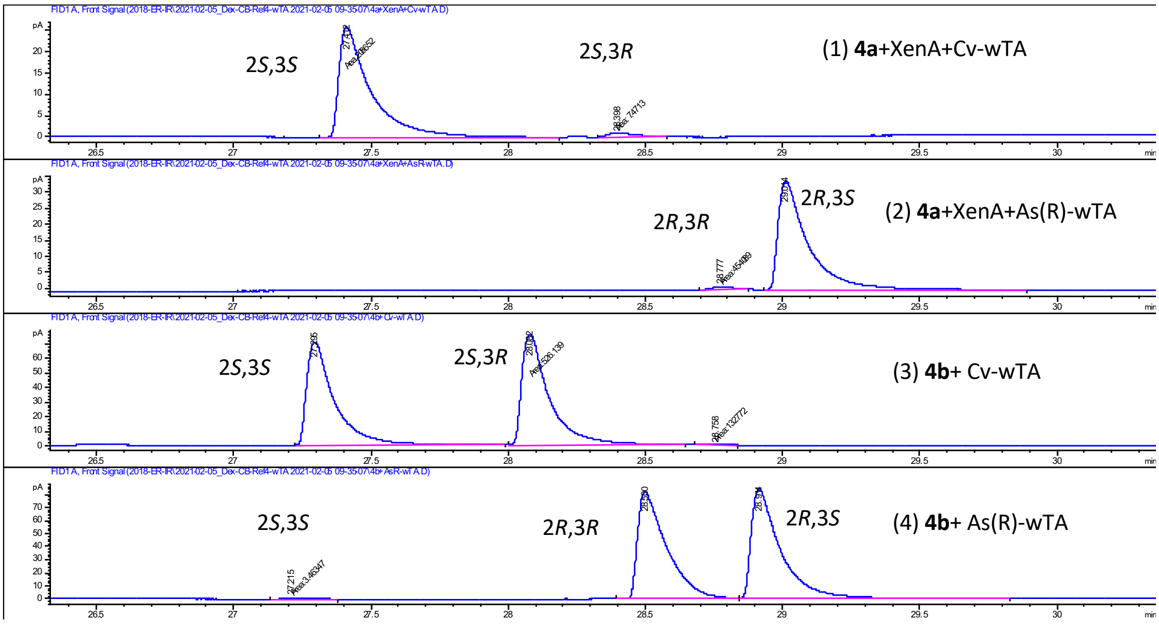

*12.4.4. References for **4d** - chemo-enzymatic synthesis*

(5) Methylation: NaH (10 equiv.) was added into a glass vial and washed with *n*-hexane to remove the oil. The glass vial was cooled down with an acetone/N_2 bath to 0 °C. The acetylated compound obtained in the previous step (paragraph 12.4.3) was dissolved in THF (2 mL) and added to the NaH containing glass vial. The reaction mixture was stirred at 0 °C for almost 2 h. Then, MeI was added (5 equiv.) and the reaction was further kept at 0 °C for some minutes; next, the acetone/N_2 bath was removed, and the reaction was stirred overnight at room temperature. Methylation was confirmed by GC-MS and work-up was performed.

(6) Work-up methylation: The organic compound was twice extracted with DCM (2x10 mL), the organic phases dried over anhydrous $MgSO_4$, and the solvent was removed under reduced pressure (30 mg were obtained).

GC-MS chromatograms of the final products obtained (all the peaks had a m/z of the desired mass of the product):

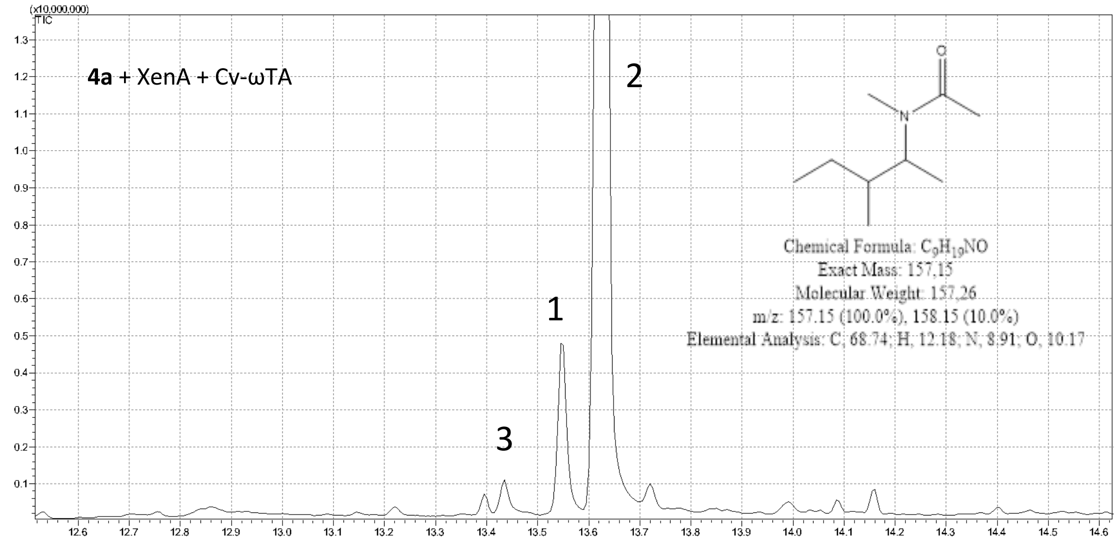

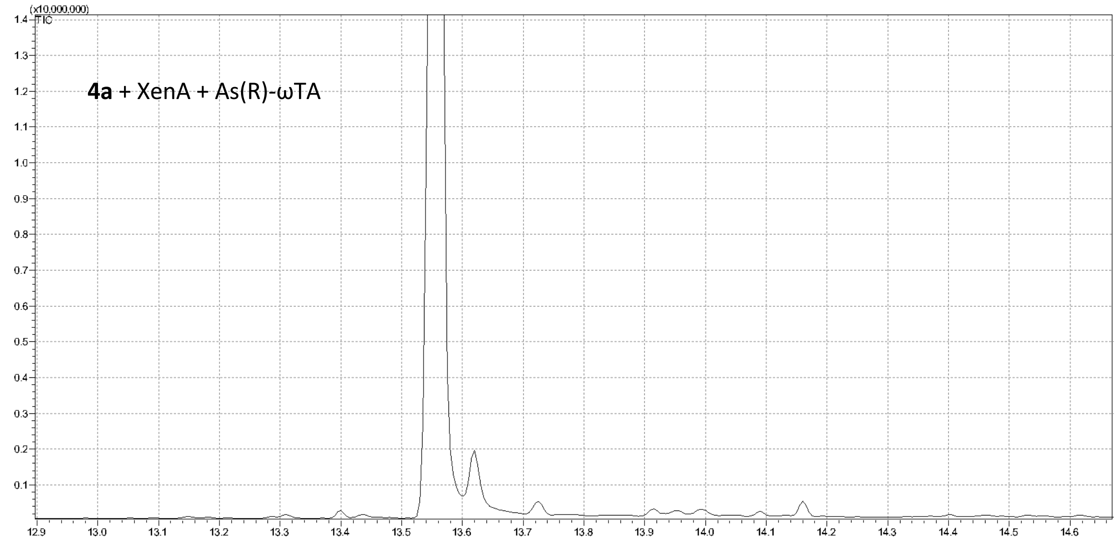

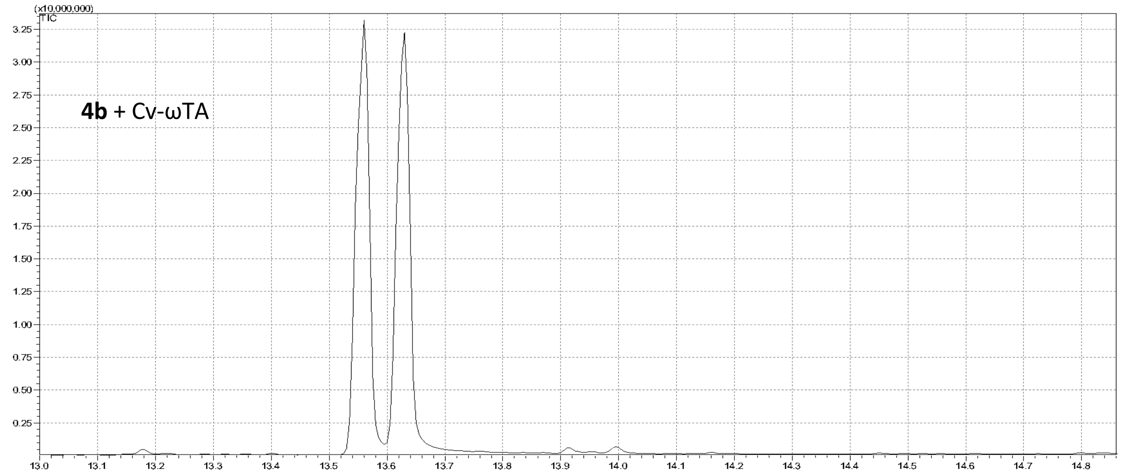

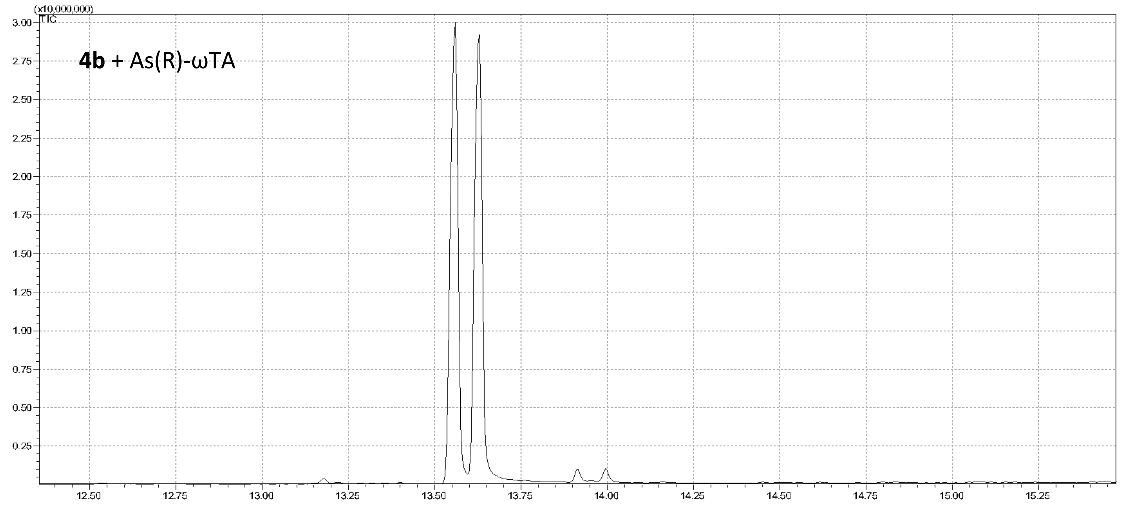

Chiral GC-FID chromatograms for the separation of the **4d** isomers from ωTA reactions with subsequent chemical acetylation and methylation:

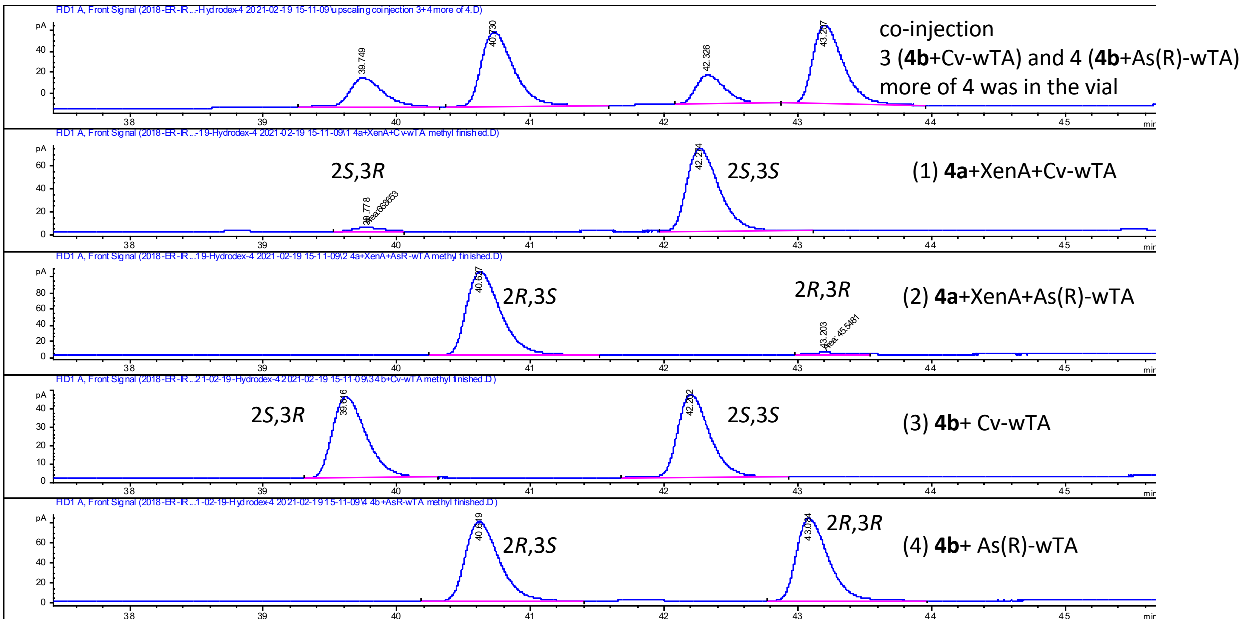

13. Analytical methods

13.1. Achiral GC-FID measurement

Column: DB1701 30m x 250 µm x 0.25 µm, Agilent. Carrier gas: H_2

Method: DB1701-method B: constant pressure 6.9 psi; 60 °C, hold 6.5 min; 20 °C min^{-1} to 100 °C, hold 1 min; 20 °C min^{-1} to 280 °C, hold 1 min.

substrate 1		min	substrate 2		min
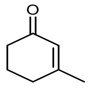 1a	**1a**	12.5	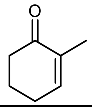	**2a**	11.0
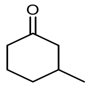 1b	**1b**	10.2	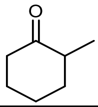	**2b**	9.9
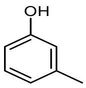		13.5	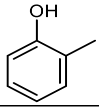		13.1
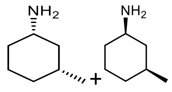	(2*S*,3*R*)-**1c** + (2*R*,3*S*)-**1c**	8.1	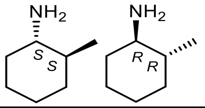	(1*S*,2*S*)-**2c** + (1*R*,2*R*)-**2c**	8.1
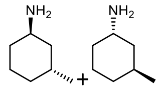	(2*R*,3*R*)-**1c** + (2*S*,3*S*)-**1c**	8.3	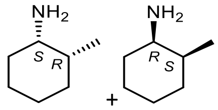	(1*S*,2*R*)-**2c** + (1*R*,2*S*)-**2c**	8.4
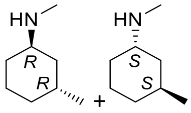	(1*R*,3*R*)-**1d** + (1*S*,3*S*)-**1d**	9.0	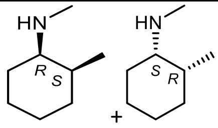	(1*R*,2*S*)-**2d** + (1*S*,2*R*)-**2d**	9.0
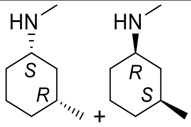	(1*S*,3*R*)-**1d** + (1*R*,3*S*)-**1d**	9.4	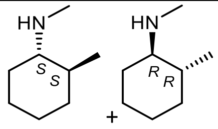	(1*S*,2*S*)-**2d** + (1*R*,2*R*)-**2d**	9.3

substrate 3		min	substrate 4		min
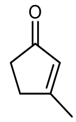	**3a**	11.1	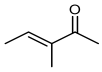	**4a**	7.3
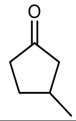	**3b**	7.7	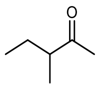	**4b**	4.5
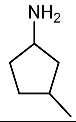	**3c** only 1 peak	5.1	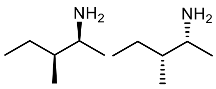	(2*S*,3*S*)-**4c** + (2*R*,3*R*)-**4c**	4.1
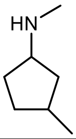	**3d** only 1 peak	6.6	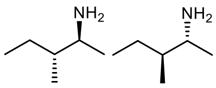	(2*S*,3*R*)-**4c** + (2*R*,3*S*)-**4c**	4.2

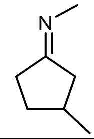		9.2	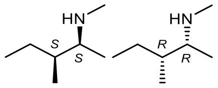	(2*S*,3*S*)-**4d** + (2*R*,3*R*)-**4d**	4.7
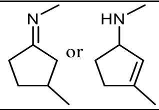		9.1	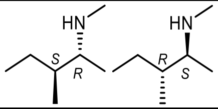	(2*S*,3*R*)-**4d** + (2*R*,3*S*)-**4d**	4.9

13.2. Chiral GC-FID measurements for the separation of the saturated intermediates 1b-4b

Columns: Rt-bDEXsm, 30m x 250 µm x 0.25 µm, Restek. Carrier gas: H_2

Rt-bDEXsa, 30m x 250 µm x 0.25 µm, Restek. Carrier gas: H_2

Methods: Restek-SM-A (with column Rt-bDEXsm): constant flow 0.9 mL min^{-1}; 60 °C, hold 6.5 min; 10 °C min^{-1} to 230 °C, hold 1 min.

Restek-SM-F (with column Rt-bDEXsm): constant flow 0.9 mL min^{-1}; 60 °C, hold 6.5 min; 5 °C min^{-1} to 110 °C, hold 10 min; 10 °C min^{-1} to 230 °C, hold 1 min.

Restek-SM-E (with column Rt-bDEXsm): constant flow 0.9 mL min^{-1}; 60 °C, hold 6.5 min; 5 °C min^{-1} to 130 °C, hold 5 min; 10 °C min^{-1} to 230 °C, hold 1 min.

Restek-SA-A (with column Rt-bDEXsa): constant flow 0.9 mL min^{-1}; 80 °C, hold 6.5 min; 10 °C min^{-1} to 230 °C, hold 1 min.

method used	enantiomer 1	enantiomer 2
Restek-SM-F	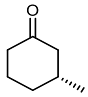 (*R*)-**1b** 19.44 min	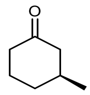 (*S*)-**1b** 19.70 min
Restek-SA-A	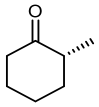 (*R*)-**2b** 13.50 min	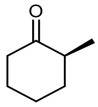 (*S*)-**2b** 13.68 min
Restek-SM-E	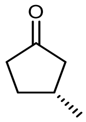 (*R*)-**3b** 15.16 min	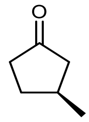 (*S*)-**3b** 15.30 min
Restek-SM-A	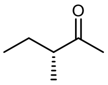 (*R*)-(-)-**4b** 8.53 min	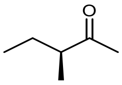 (*S*)-(+)-**4b** 8.82 min

13.1. Chiral GC measurements for the separation of the diastereomers

Columns: CP-Chirasil (Dex-CB, CP7503) 25m x 0.32 mm x 0.25 µm, Agilent. Carrier gas: H_2
Hydrodex ß-TBDAc 50 m x 0.25 mm, Macherey Nagel. Carrier gas: H_2
DB1701 60m x 250 µm x 0.25 µm, Agilent. Carrier gas: H_2

Methods: Dex-CB-amid: constant flow 1.5 ml min^{-1}; 60 °C, hold 1 min; 2 °C min^{-1} to 150 °C, hold 0 min; 10 °C min^{-1} to 180 °C, hold 1 min.
Hydrodex-ß-TBDAC-4D-short: constant flow 1.1 ml min^{-1}; 60 °C, hold 1 min; 2 °C min^{-1} to 148 °C, hold 0 min; 10 °C min^{-1} to 220 °C, hold 1 min.
DB1701-60m-3d: constant pressure 14.11 psi; 80 °C, hold 13 min; 5 °C min^{-1} to 200 °C, hold 10 min; 20 °C min^{-1} to 280 °C, hold 3 min.

DEX-CB-amid<br>OLD column [a]<br>after derivatization<br>(DMAP/acetic anhydride)	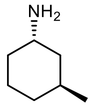<br>(1*S*,2*S*)-**1c**<br>39.2 min	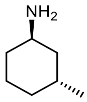<br>(1*R*,2*R*)-**1c**<br>39.4 min	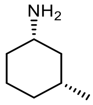<br>(1*S*,2*R*)-**1c**<br>39.7 min	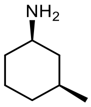<br>(1*R*,2*S*)-**1c**<br>40.0 min
Dex-CB-amid<br>NEW column<br>after derivatization<br>(DMAP/acetic anhydride)	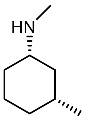<br>(1*S*,3*R*)-**1d**<br>39.0 min	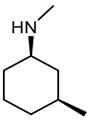<br>(1*R*,3*S*)-**1d**<br>39.6 min	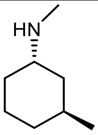<br>1*S*,3*S*-**1d**<br>41.0 min	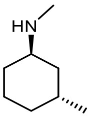<br>1*R*,3*R*-**1d**<br>41.5 min
Dex-CB-amid<br>NEWcolumn<br>after derivatization<br>(DMAP/acetic anhydride)	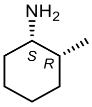<br>(1*S*,2*R*)-**2c**<br>39.6 min	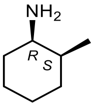<br>(1*R*,2*S*)-**2c**<br>39.8 min	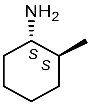<br>(1*S*,2*S*)-**2c**<br>40.6 min	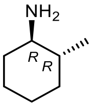<br>(1*R*,2*R*)-**2c**<br>3941.0 min
Dex-CB-amid<br>NEW column<br>after derivatization<br>(DMAP/acetic anhydride)	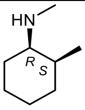<br>(1*R*,2*S*)-**2d**<br>39.9 min	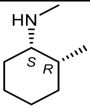<br>(1*S*,2*R*)-**2d**<br>40.3 min	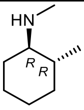<br>(1*R*,2*R*)-**2d**<br>43.4 min	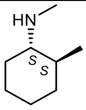<br>(1*S*,2*S*)-**2d**<br>43.9 min
DB1701-60m-3d<br>after derivatization<br>(DMAP/benzoic anhydride)Y	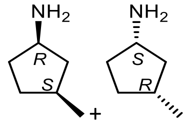<br>(1*R*,3*S*)-**3c**+(1*S*,3*R*)-**3c**; 52.0 min		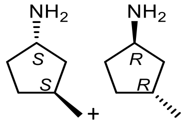<br>(1*S*,3*S*)-**3c**+(1*R*,3*R*)-**3c**; 52.2 min	
DB1701-60m-3d<br>after derivatization<br>(DMAP/benzoic anhydride)	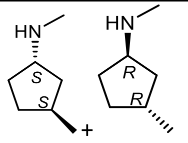<br>(1*S*,3*S*)-**3d**+(1*R*,3*R*)-**3d**; 51.3 min		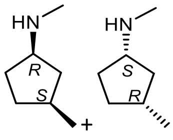<br>(1*R*,3*S*)-**3d**+(1*S*,3*R*)-**3d**; 51.5 min	
Dex-CB-amid<br>NEW column<br>after derivatization<br>(DMAP/acetic anhydride)	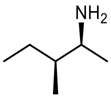<br>(2*S*,3*S*)-**4c**<br>30.6 min	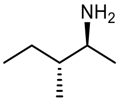<br>(2*S*,3*R*)-**4c**<br>31.3 min	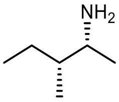<br>(2*R*,3*R*)-**4c**<br>31.6 min	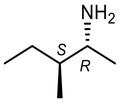<br>(2*R*,3*S*)-**4c**<br>32.1 min
Hydrodex-B-TBDAC-4D-short<br>after derivatization<br>(DMAP/acetic anhydride)	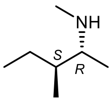<br>(2*S*,3*R*)-**4d**<br>39.8 min	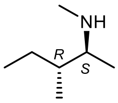<br>(2*R*,3*S*)-**4d**<br>40.7 min	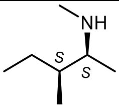<br>(2*S*,3*S*)-**4d**<br>42.3 min	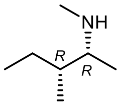<br>(2*R*,3*R*)-**4d**<br>43.2 min

[a] 4 peaks get only separated with our old column, not with the new one. (Comment: with the new Dex-CB column peak 2 and 3 do not get resolved (38.66 + 39.78 + 39.87 + 41.20 min retention time)

14. Representative GC-chromatograms

14.1. Representative achiral GC-chromatograms

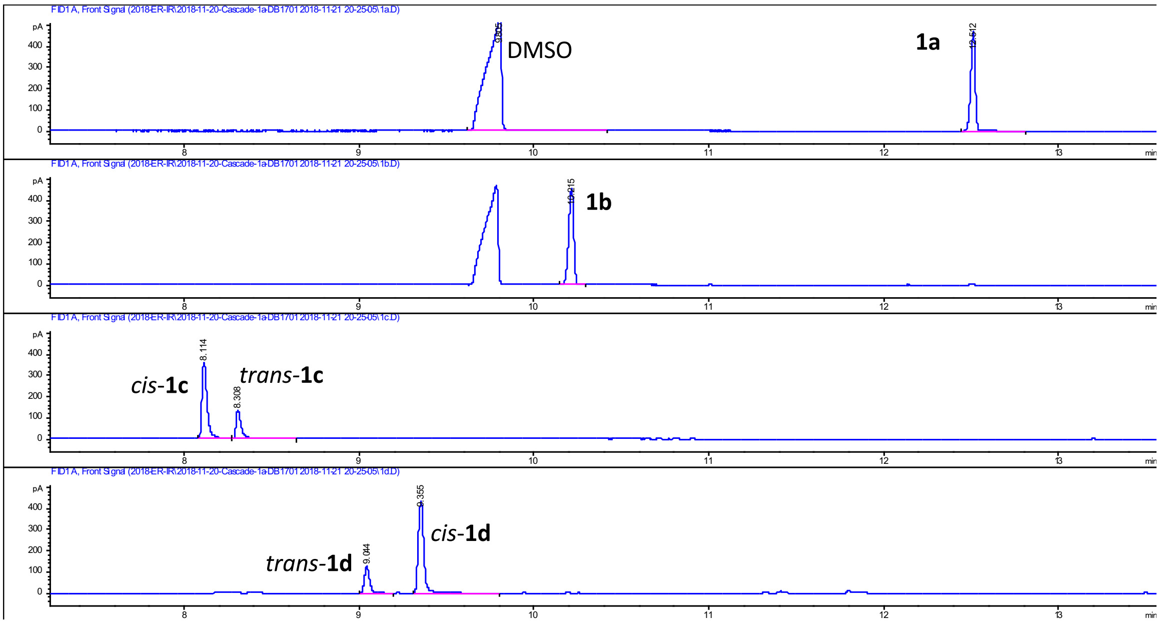

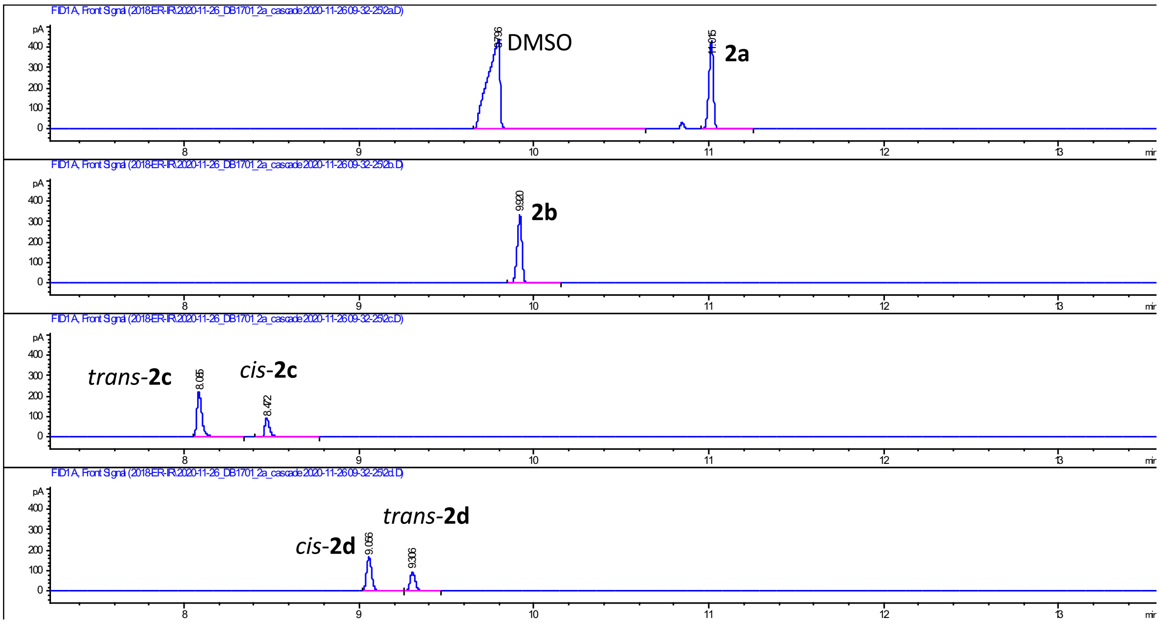

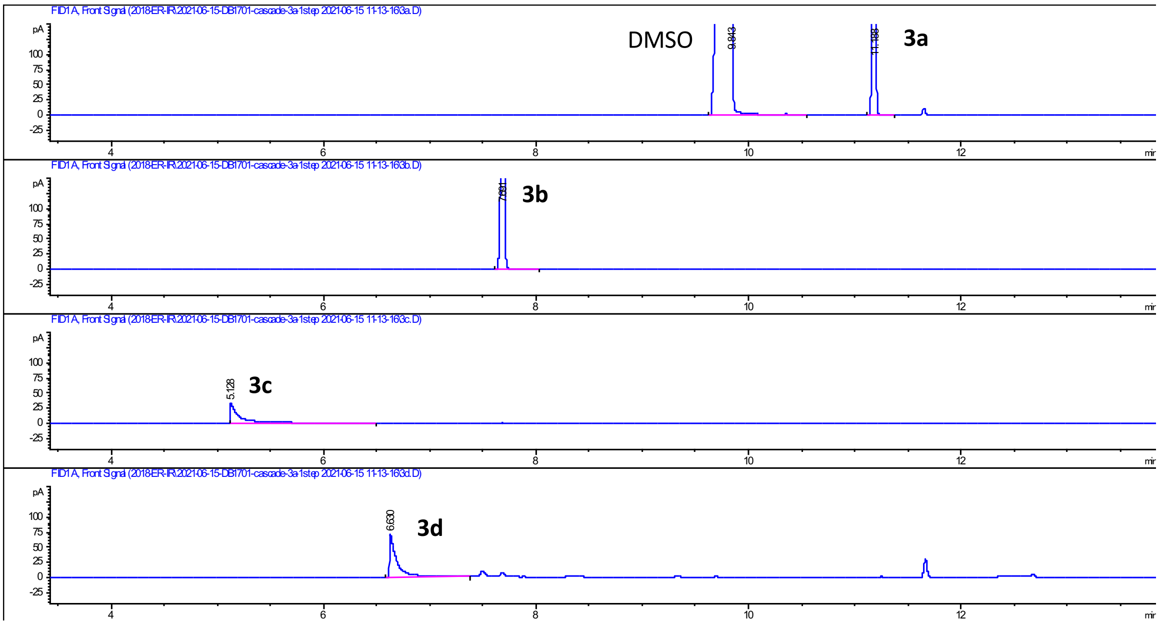
DMSO
3a
3b
3c
5.128
3d
6.630

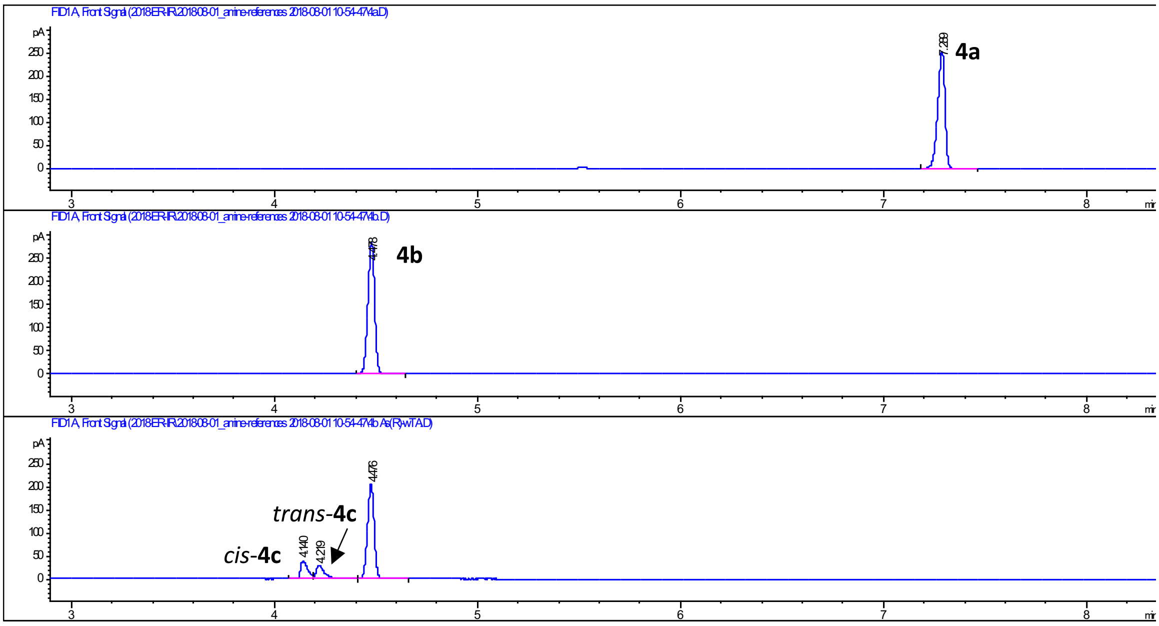
4a
4b
trans-4c
cis-4c
4.140
4.219
4.476

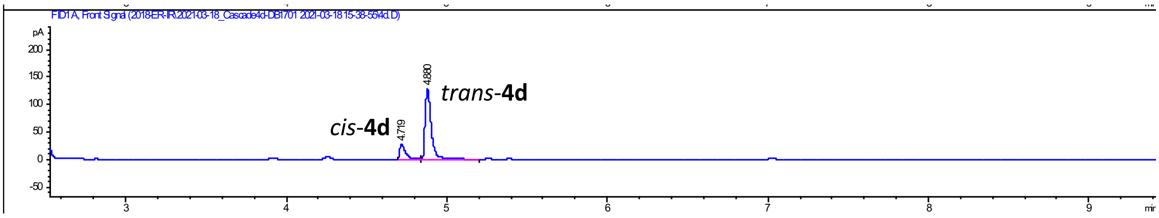
cis-4d
trans-4d
4.719
4.880

14.2. Representative chiral GC-FID chromatograms for the best performing reactions

14.2.1. Chiral primary amines

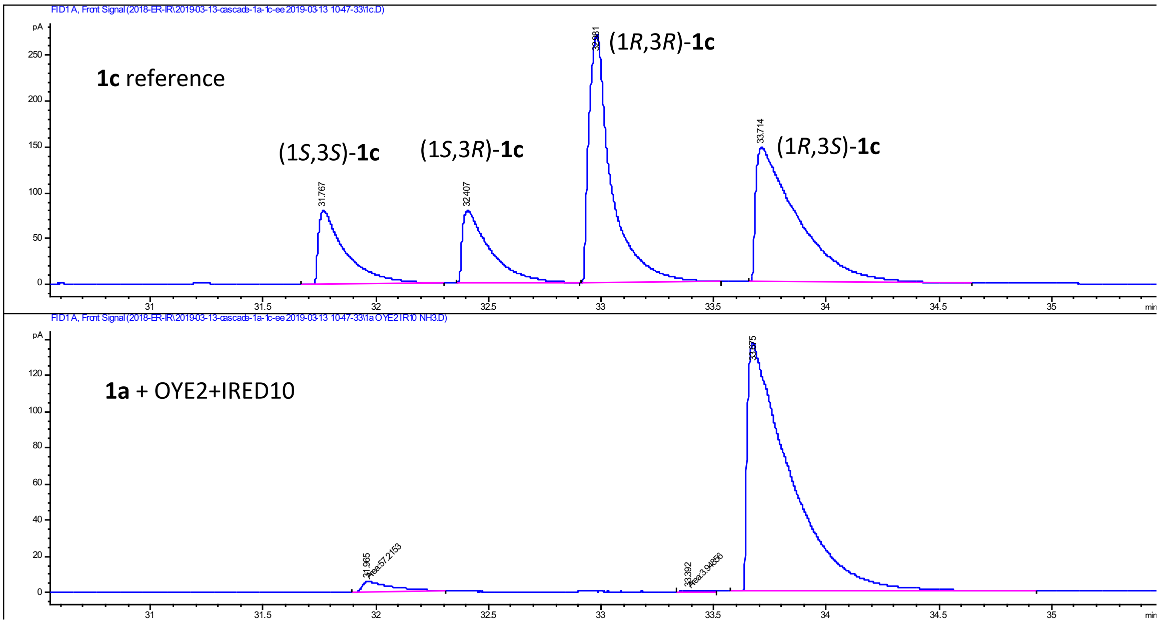

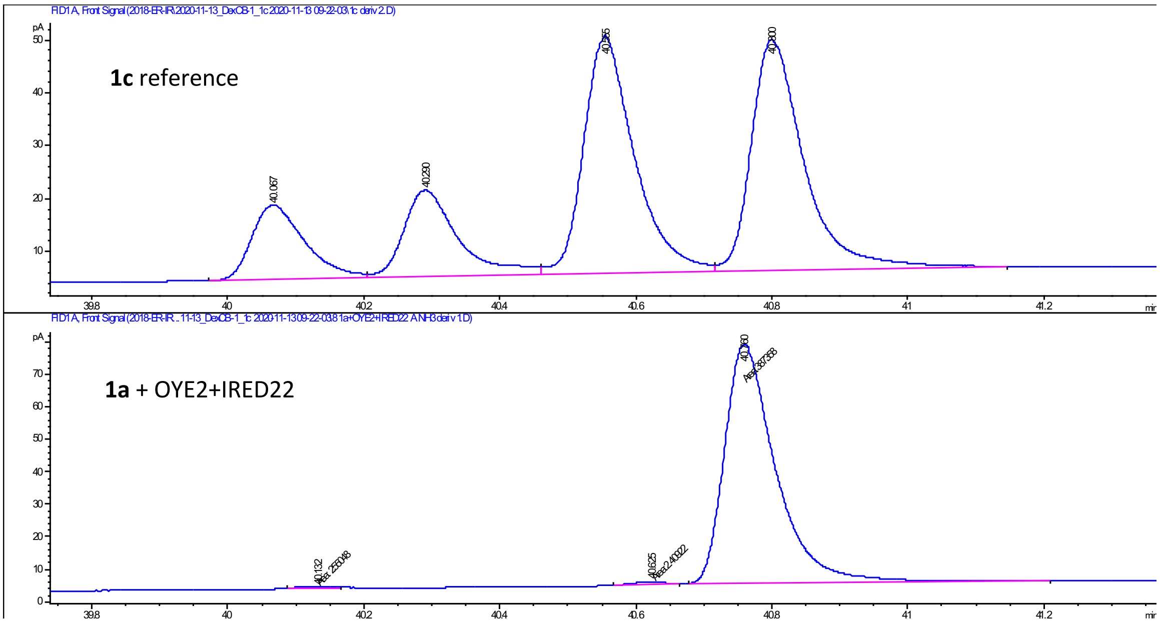

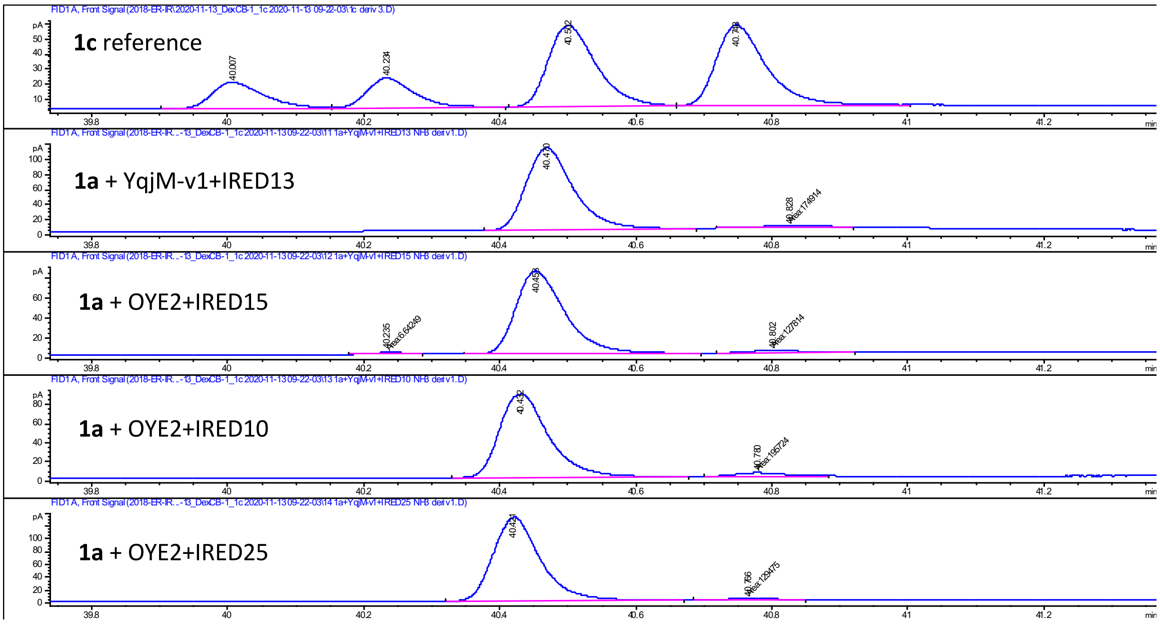
1c reference
1a + YqjM-v1+IRED13
1a + OYE2+IRED15
1a + OYE2+IRED10
1a + OYE2+IRED25

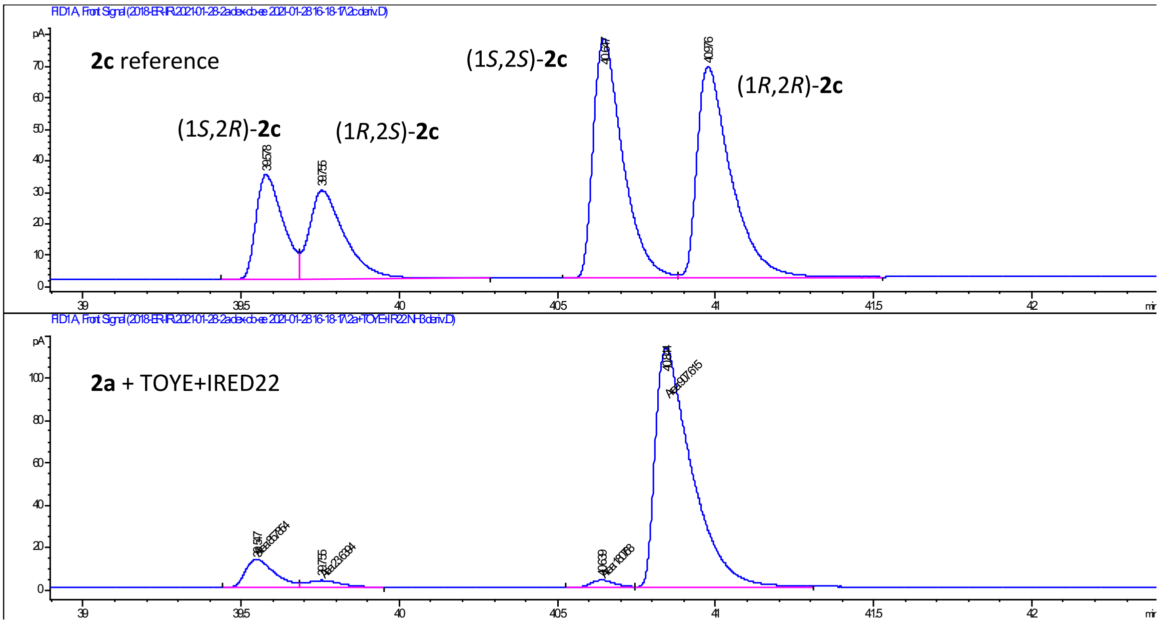
2c reference
(1S,2R)-2c
(1R,2S)-2c
(1S,2S)-2c
(1R,2R)-2c
2a + TOYE+IRED22

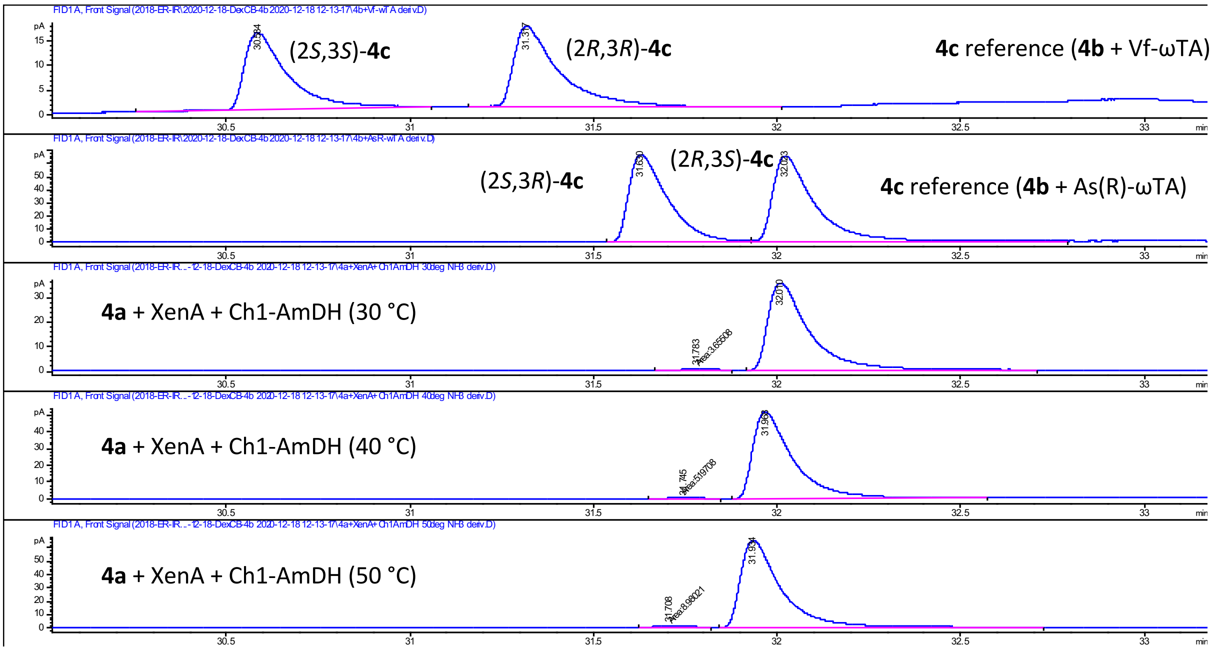
(2*S*,3*S*)-**4c**
(2*R*,3*R*)-**4c**
4c reference (**4b** + Vf-ωTA)
(2*S*,3*R*)-**4c**
(2*R*,3*S*)-**4c**
4c reference (**4b** + As(R)-ωTA)
4a + XenA + Ch1-AmDH (30 °C)
4a + XenA + Ch1-AmDH (40 °C)
4a + XenA + Ch1-AmDH (50 °C)

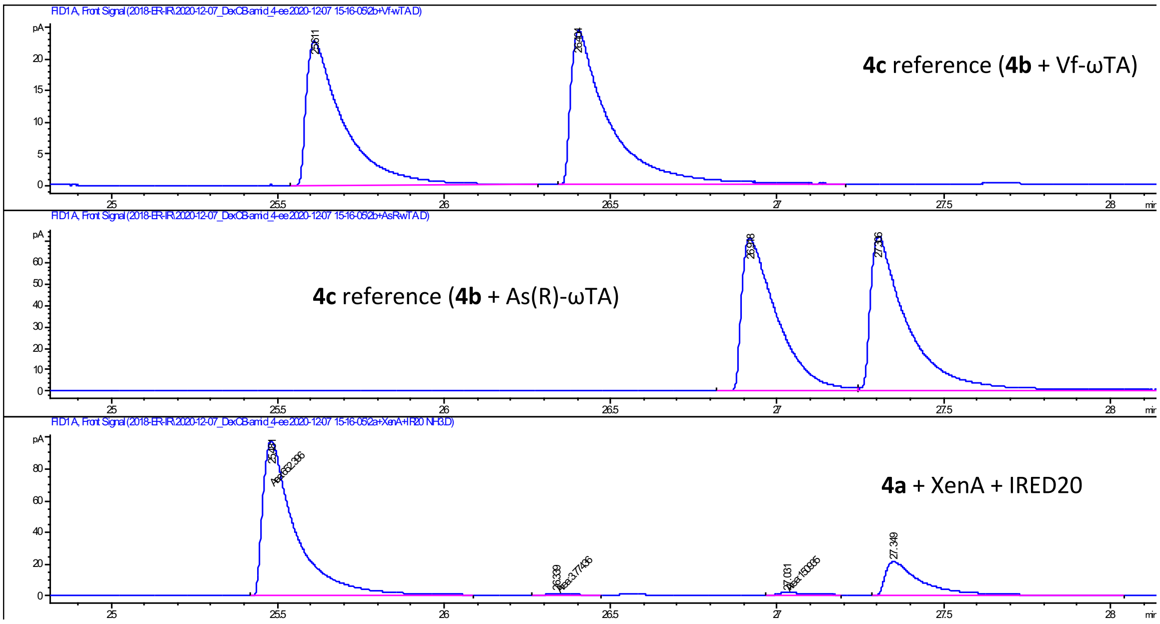
4c reference (**4b** + Vf-ωTA)
4c reference (**4b** + As(R)-ωTA)
4a + XenA + IRED20

14.2.2. Chiral secondary (N-methyl)amines

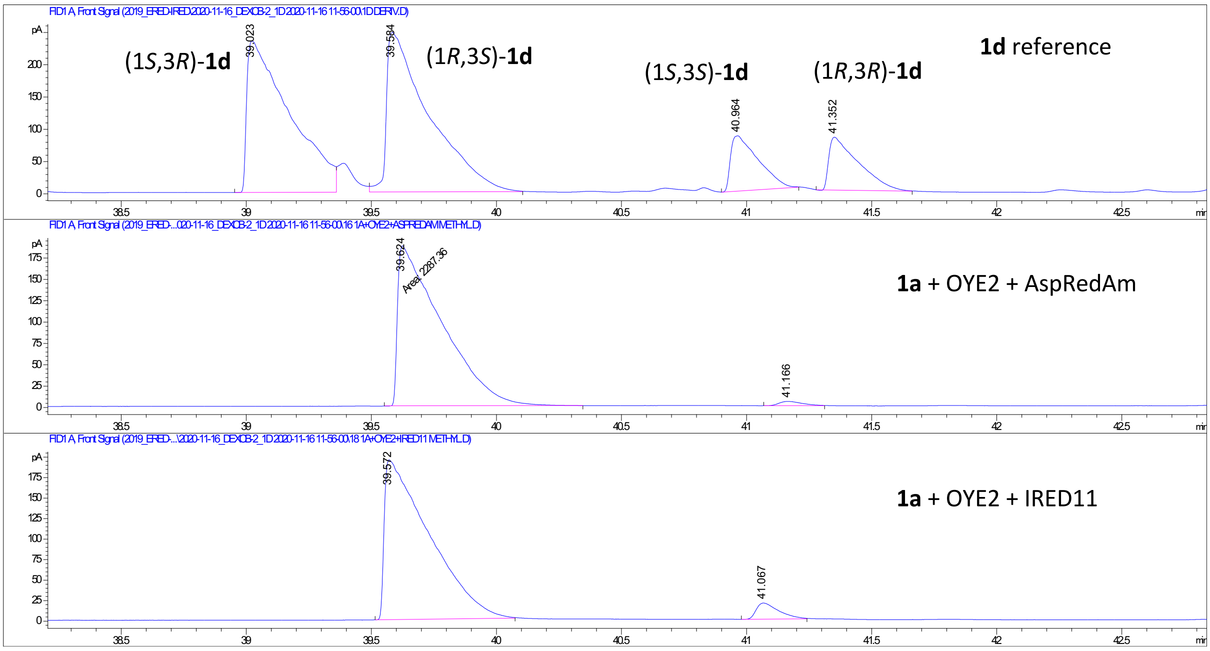

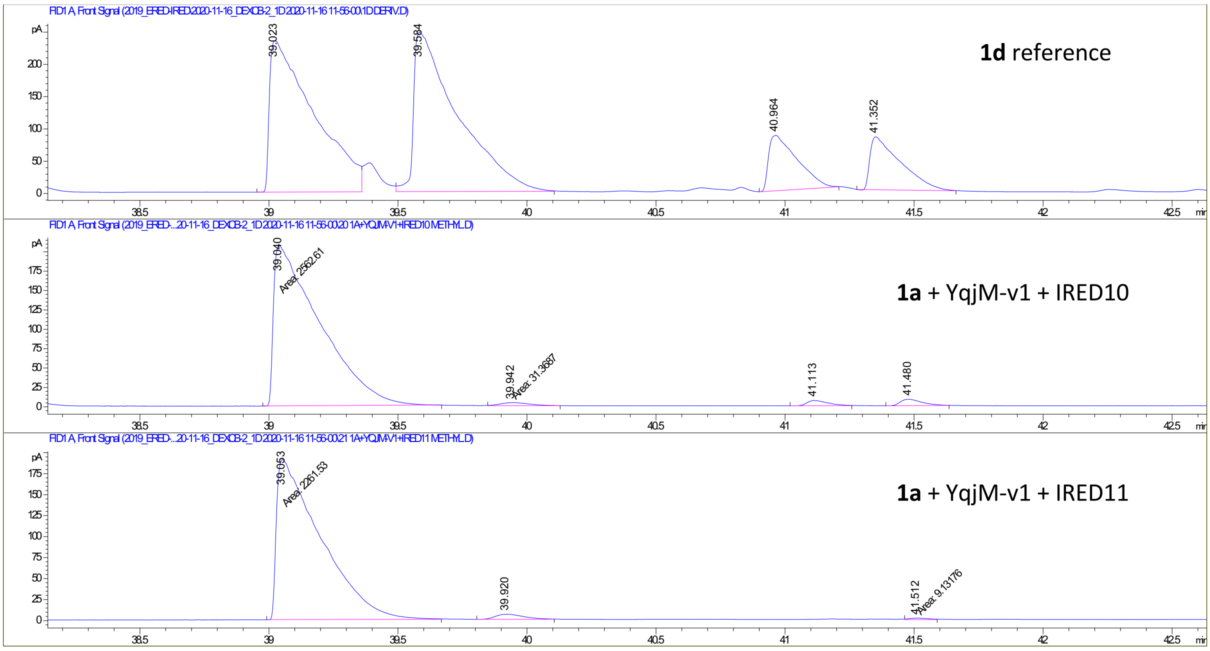

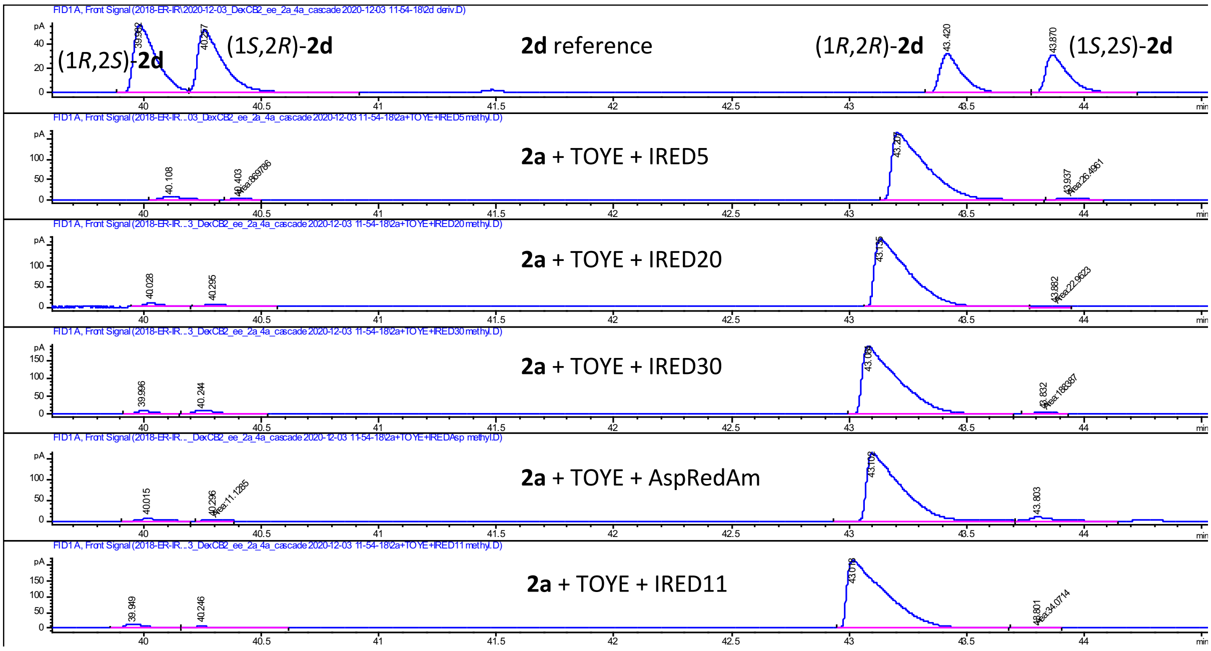
(1R,2S)-2d
(1S,2R)-2d
2d reference
(1R,2R)-2d
(1S,2S)-2d
2a + TOYE + IRED5
2a + TOYE + IRED20
2a + TOYE + IRED30
2a + TOYE + AspRedAm
2a + TOYE + IRED11

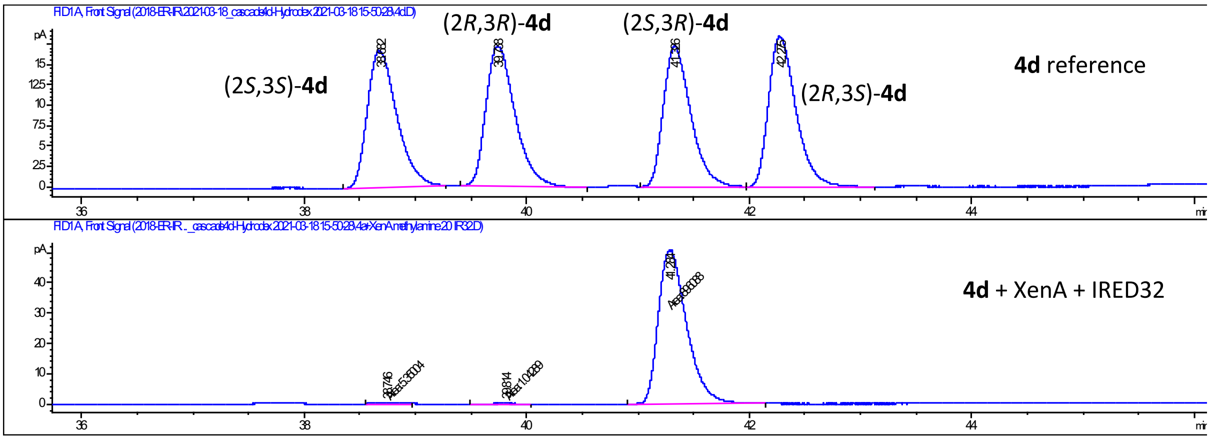
(2R,3R)-4d
(2S,3R)-4d
(2S,3S)-4d
(2R,3S)-4d
4d reference
4d + XenA + IRED32

14.3. GC-FID chromatograms for substrate 3

14.3.1. Cascade reaction combining *3a* with OYE2 and IRED22 (ammonium formate buffer)

e.r. of **3b**: GC-FID (Restek-SM-E)

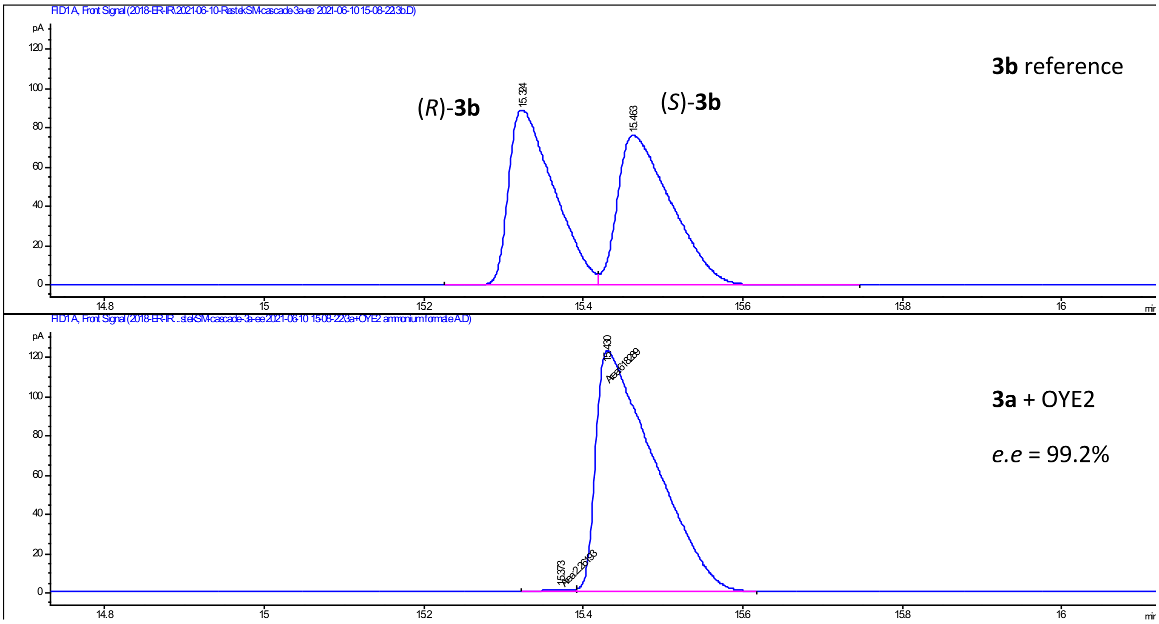

conversion for biocatalytic cascade reaction: DB1701-30m column

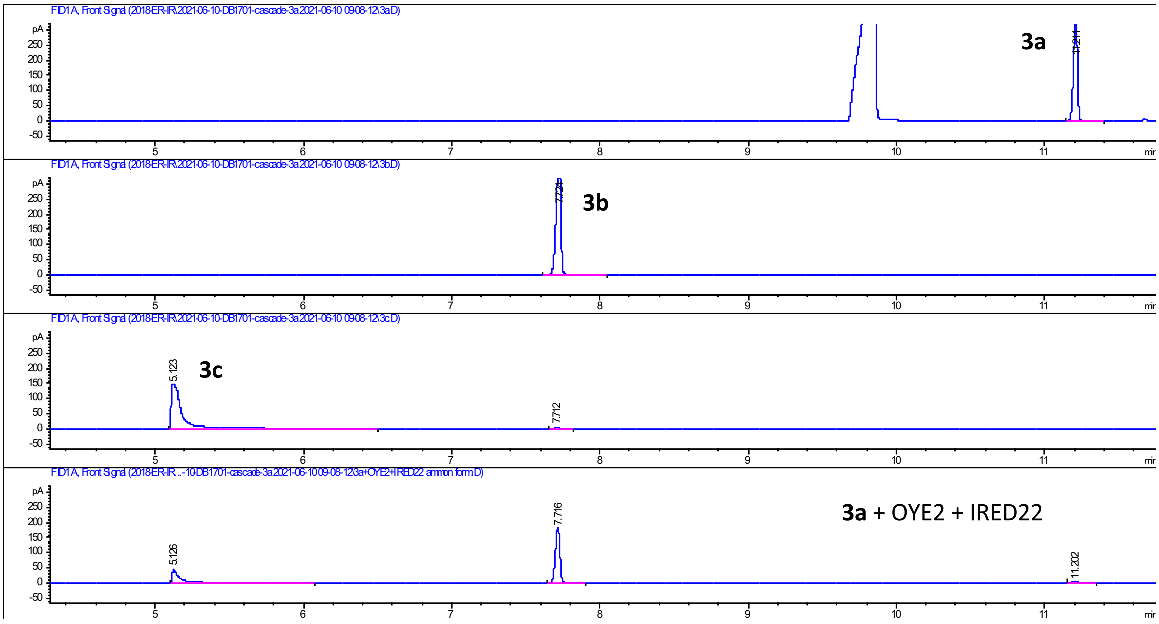

d.r. for cascade reaction after derivatization with benzoic acid/DMAP: DB1701-60m column

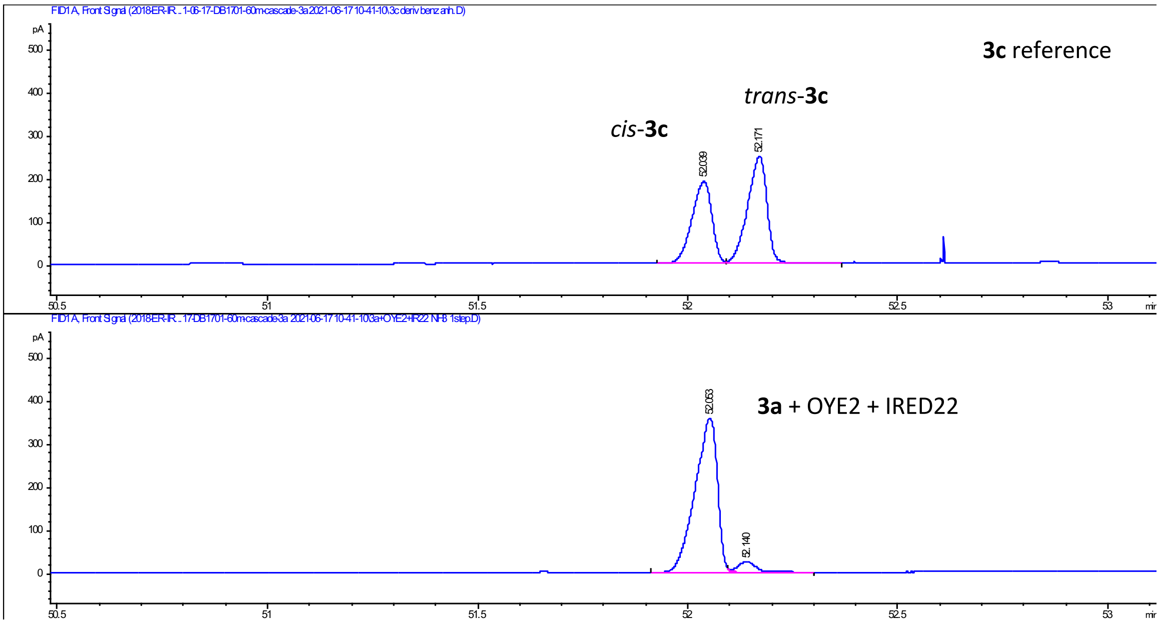

*14.3.1. Cascade reaction combining **3a** with OYE2 and IRED20 and IRED15 (methylammonium formate buffer)*

e.r. of **3b**: GC-FID (Restek-SM-E)

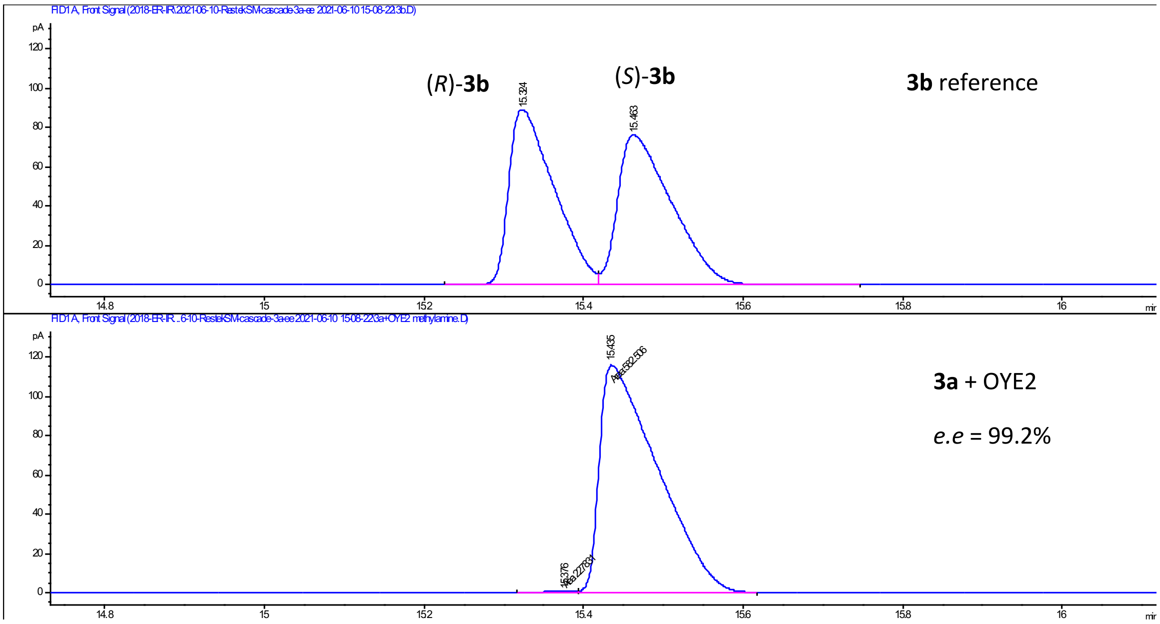

conversion for biocatalytic cascade reaction (concurrent): DB1701-30m column

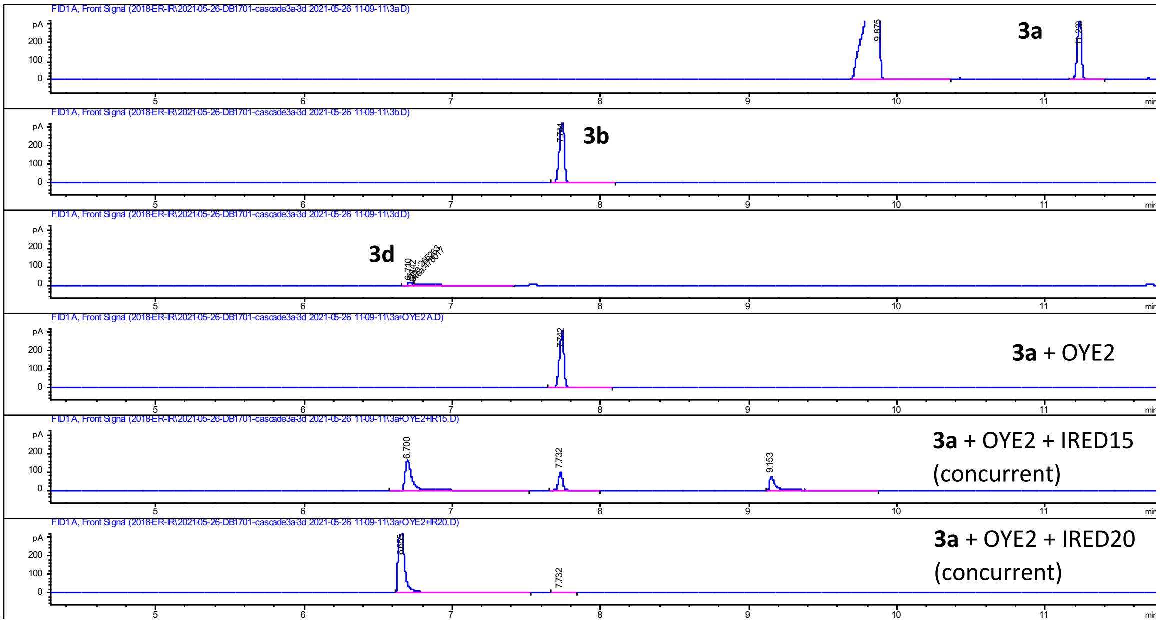

d.r. for cascade reaction (concurrent) after derivatization with benzoic acid/DMAP: DB1701-60m column

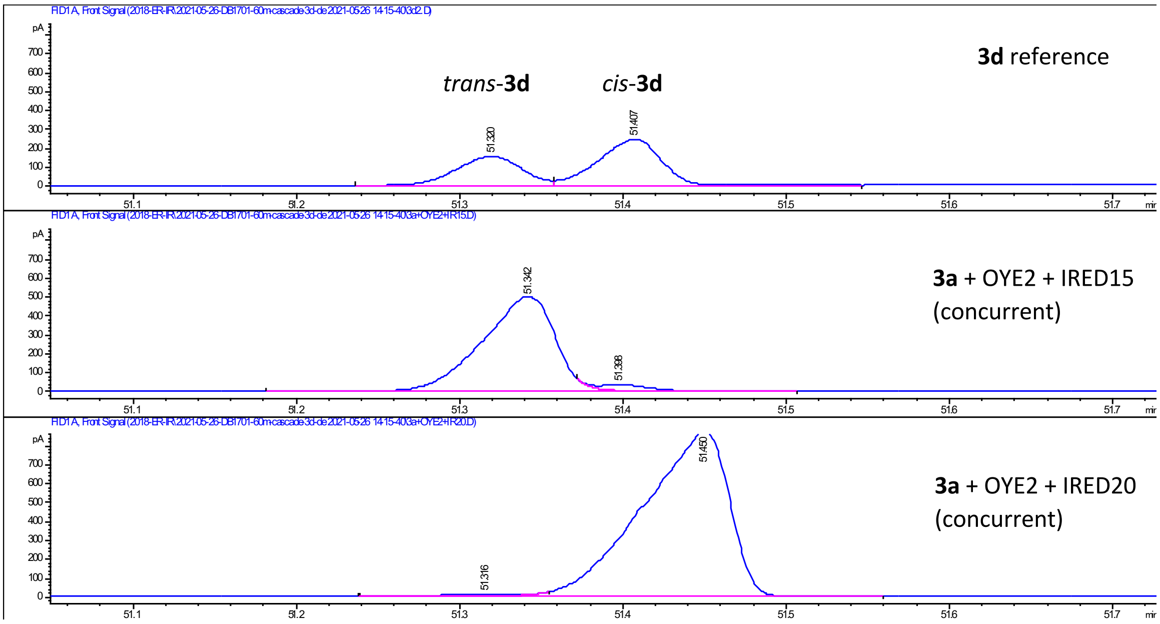

conversion for biocatalytic cascade reaction (sequential): DB1701-30m column

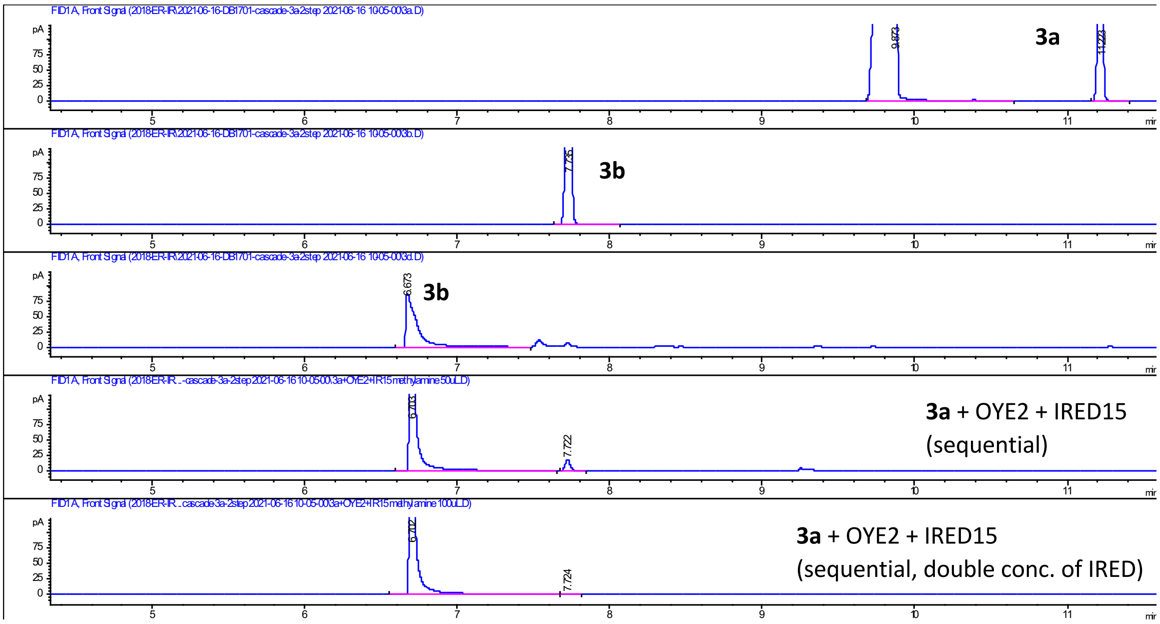

d.r. for cascade reaction (sequential) after derivatization with benzoic acid/DMAP: DB1701-60m column

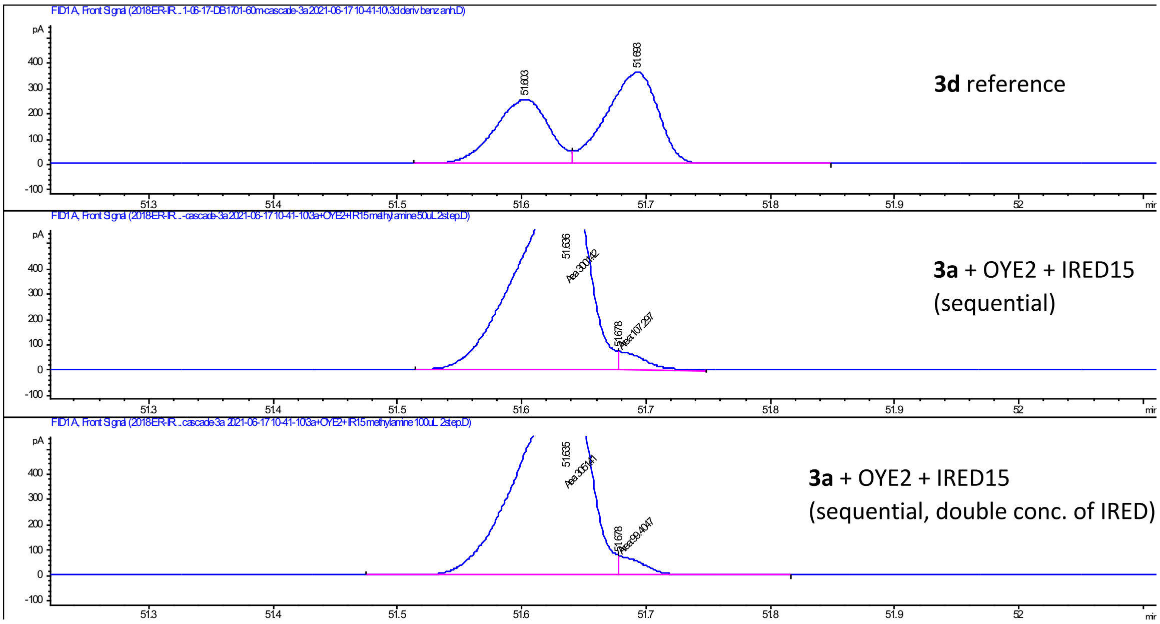

15. Literature

1. Sheldon, R. A.; Arends, I.; Hanefeld, U., *Green Chemistry and Catalysis*. Wiley-VCH Verlag GmbH & Co. KGa: **2007**, 2-5.
2. Knaus, T.; Mutti, F. G.; Humphreys, L. D.; Turner, N. J.; Scrutton, N. S., Systematic methodology for the development of biocatalytic hydrogen-borrowing cascades: application to the synthesis of chiral α-substituted carboxylic acids from α-substituted α,β-unsaturated aldehydes. *Org. Biomol. Chem.* **2015,** *13*, 223-233.
3. Skalden, L.; Peters, C.; Ratz, L.; Bornscheuer, U. T., Synthesis of (1*R*,3*R*)-1-amino-3-methylcyclohexane by an enzyme cascade reaction. *Tetrahedron* **2016,** *72* (46), 7207-7211.
4. Leipold, F.; Hussain, S.; Ghislieri, D.; Turner, N. J., Asymmetric Reduction of Cyclic Imines Catalyzed by a Whole-Cell Biocatalyst Containing an (*S*)-Imine Reductase. *ChemCatChem* **2013,** *5* (12), 3505-3508.
5. Wetzl, D.; Berrera, M.; Sandon, N.; Fishlock, D.; Ebeling, M.; Muller, M.; Hanlon, S.; Wirz, B.; Iding, H., Expanding the Imine Reductase Toolbox by Exploring the Bacterial Protein-Sequence Space. *ChemBioChem* **2015,** *16* (12), 1749-56.
6. Wetzl, D.; Gand, M.; Ross, A.; Müller, H.; Matzel, P.; Hanlon, S. P.; Müller, M.; Wirz, B.; Höhne, M.; Iding, H., Asymmetric Reductive Amination of Ketones Catalyzed by Imine Reductases. *ChemCatChem* **2016,** *8* (12), 2023-2026.
7. Aleku, G. A.; France, S. P.; Man, H.; Mangas-Sanchez, J.; Montgomery, S. L.; Sharma, M.; Leipold, F.; Hussain, S.; Grogan, G.; Turner, N. J., A reductive aminase from *Aspergillus oryzae*. *Nat. Chem.* **2017,** *9* (10), 961-969.
8. Mutti, F. G.; Knaus, T.; Scrutton, N. S.; Breuer, M.; Turner, N. J., Conversion of alcohols to enantiopure amines through dual-enzyme hydrogen-borrowing cascades. *Science* **2015,** *349* (6255), 1525-1529.
9. Knaus, T.; Böhmer, W.; Mutti, F. G., Amine dehydrogenases: efficient biocatalysts for the reductive amination of carbonyl compounds. *Green Chem.* **2017,** *19*, 453-463.
10. Tseliou, V.; Knaus, T.; Masman, M. F.; Corrado, M. L.; Mutti, F. G., Generation of amine dehydrogenases with increased catalytic performance and substrate scope from ε-deaminating L-Lysine dehydrogenase. *Nature Communications* **2019,** *10* (1), 3717.
11. Ihara, M.; Kawano, Y.; Urano, M.; Okabe, A., Light driven CO2 fixation by using cyanobacterial photosystem I and NADPH-dependent formate dehydrogenase. *PloS one* **2013,** *8* (8), e71581.
12. French, C. E.; Nicklin, S.; Bruce, N. C., Sequence and properties of pentaerythritol tetranitrate reductase from *Enterobacter cloacae* PB2. *J. Bacteriol.* **1996,** *178* (22), 6623-7.
13. Adalbjornsson, B. V.; Toogood, H. S.; Fryszkowska, A.; Pudney, C. R.; Jowitt, T. A.; Leys, D.; Scrutton, N. S., Biocatalysis with thermostable enzymes: structure and properties of a thermophilic 'ene'-reductase related to old yellow enzyme. *ChemBiochem* **2010,** *11* (2), 197-207.
14. Karplus, P. A.; Fox, K. M.; Massey, V., Flavoprotein structure and mechanism. 8. Structure-function relations for old yellow enzyme. *The FASEB Journal* **1995,** *9* (15), 1518-26.
15. Blehert, D. S.; Fox, B. G.; Chambliss, G. H., Cloning and Sequence Analysis of Two *Pseudomonas* Flavoprotein Xenobiotic Reductases. *J. Bacteriol.* **1999,** *181* (20), 6254-6263.
16. Straßner, J.; Fürholz, A.; Macheroux, P.; Amrhein, N.; Schaller, A., A Homolog of Old Yellow Enzyme in Tomato: Spectral Properties and Substrate Specificity of the Recombinant Protein. *J. Biol. Chem.* **1999,** *274* (49), 35067-35073.
17. Snape, J. R.; Walkley, N. A.; Morby, A. P.; Nicklin, S.; White, G. F., Purification, properties, and sequence of glycerol trinitrate reductase from *Agrobacterium radiobacter*. *J. Bacteriol.* **1997,** *179* (24), 7796-802.
18. Richter, N.; Groger, H.; Hummel, W., Asymmetric reduction of activated alkenes using an enoate reductase from *Gluconobacter oxydans*. *Appl. Microbiol. Biotechnol.* **2011,** *89* (1), 79-89.
19. Fitzpatrick, T. B.; Amrhein, N.; Macheroux, P., Characterization of YqjM, an Old Yellow Enzyme homolog from *Bacillus subtilis* involved in the oxidative stress response. *J. Biol. Chem.* **2003,** *278* (22), 19891-7.
20. French, C. E.; Bruce, N. C., Purification and characterization of morphinone reductase from *Pseudomonas putida* M10. *Biochem. J* **1994,** *301* (1), 97-103.
21. Bommarius, B. R.; Schurmann, M.; Bommarius, A. S., A novel chimeric amine dehydrogenase shows altered substrate specificity compared to its parent enzymes. *Chem. Commun.* **2014,** *50* (95), 14953-14955.
22. Abrahamson, M. J.; Vázquez-Figueroa, E.; Woodall, N. B.; Moore, J. C.; Bommarius, A. S., Development of an Amine Dehydrogenase for Synthesis of Chiral Amines. *Angew. Chem. Int. Ed.* **2012,** *51* (16), 3969-3972.
23. Schutte, H.; Flossdorf, J.; Sahm, H.; Kula, M.-R., Purification and Properties of Formaldehyde Dehydrogenase and Formate Dehydrogenase from *Candida boidinii*. *Eur. J. Biochem.* **1976,** *62* (1), 151-160.

24. Mutti, F. G.; Fuchs, C. S.; Pressnitz, D.; Turrini, N. G.; Sattler, J. H.; Lerchner, A.; Skerra, A.; Kroutil, W., Amination of Ketones by Employing Two New (*S*)-Selective ω-Transaminases and the His-Tagged ω-TA from *Vibrio fluvialis*. *Eur. J. Org. Chem.* **2012,** *2012* (5), 1003-1007.
25. Koszelewski, D.; Göritzer, M.; Clay, D.; Seisser, B.; Kroutil, W., Synthesis of Optically Active Amines Employing Recombinant ω-Transaminases in *E. coli* Cells. *ChemCatChem* **2010,** *2* (1), 73-77.
26. Mutti, F. G.; Fuchs, C. S.; Pressnitz, D.; Sattler, J. H.; Kroutil, W., Stereoselectivity of Four (*R*)-Selective Transaminases for the Asymmetric Amination of Ketones. *Adv. Synth. Catal.* **2011,** *353* (17), 3227-3233.
27. Hall, M.; Stueckler, C.; Hauer, B.; Stuermer, R.; Friedrich, T.; Breuer, M.; Kroutil, W.; Faber, K., Asymmetric Bioreduction of Activated C=C Bonds Using *Zymomonas mobilis* NCR Enoate Reductase and Old Yellow Enzymes OYE 1–3 from Yeasts. *Eur. J. Org. Chem.* **2008,** *2008* (9), 1511-1516.
28. Hall, M.; Stueckler, C.; Ehammer, H.; Pointner, E.; Oberdorfer, G.; Gruber, K.; Hauer, B.; Stuermer, R.; Kroutil, W.; Macheroux, P.; Faber, K., Asymmetric Bioreduction of C=C Bonds using Enoate Reductases OPR1, OPR3 and YqjM: Enzyme-Based Stereocontrol. *Adv. Synth. Catal.* **2008,** *350* (3), 411-418.
29. Skalden, L.; Peters, C.; Dickerhoff, J.; Nobili, A.; Joosten, H. J.; Weisz, K.; Hohne, M.; Bornscheuer, U. T., Two subtle amino Acid changes in a transaminase substantially enhance or invert enantiopreference in cascade syntheses. *ChemBioChem* **2015,** *16* (7), 1041-5.
30. Richter, N.; Simon, R. C.; Lechner, H.; Kroutil, W.; Ward, J. M.; Hailes, H. C., Omega-Transaminases for the amination of functionalised cyclic ketones. *Org. Biomol. Chem.* **2015,** *13* (33), 8843-51.
31. Baker, R.; Boyes, R. H. O.; Broom, D. M. P.; O'Mahony, M. J.; Swain, C. J., Preparation of chiral lactone from laevoglucosan; a key intermediate for synthesis of the spiroacetal moieties of the avermectins and milbemycins. *J. Chem. Soc., Perkin Trans. 1* **1987,** (0), 1613-1621.
